# Safety, Immunogenicity, and Efficacy of COVID-19 Vaccines in Adolescents, Children, and Infants: A Systematic Review and Meta-Analysis

**DOI:** 10.3389/fpubh.2022.829176

**Published:** 2022-04-14

**Authors:** Yuxuan Du, Long Chen, Yuan Shi

**Affiliations:** ^1^Department of Neonatology, Children's Hospital of Chongqing Medical University, Chongqing, China; ^2^National Clinical Research Center for Child Health and Disorders, Chongqing, China; ^3^Ministry of Education Key Laboratory of Child Development and Disorders, Chongqing, China; ^4^China International Science and Technology Cooperation Base of Child Development and Critical Disorders, Chongqing, China; ^5^Chongqing Key Laboratory of Pediatrics, Chongqing, China

**Keywords:** COVID-19 vaccine, adolescents, child, infant, randomized controlled trial, meta-analysis

## Abstract

**Background:**

As the epidemic progresses, universal vaccination against COVID-19 has been the trend, but there are still some doubts about the efficacy and safety of COVID-19 vaccines in adolescents, children, and even infants.

**Purpose:**

To evaluate the safety, immunogenicity, and efficacy of COVID-19 vaccines in the population aged 0–17 years.

**Method:**

A comprehensive search for relevant randomized controlled trials (RCTs) was conducted in PubMed, Embase, and the Cochrane Library from inception to November 9, 2021. All data were pooled by RevMan 5.3 statistical software, with risk ratio (RR) and its 95% confidence interval as the effect measure. This study protocol was registered on PROSPERO (CRD42021290205).

**Results:**

There was a total of six randomized controlled trials included in this systematic review and meta-analysis, enrolling participants in the age range of 3–17 years, and containing three types of COVID-19 vaccines. Compared with mRNA vaccines and adenovirus vector vaccines, inactivated vaccines have a more satisfactory safety profile, both after initial (RR 1.40, 95% CI 1.04–1.90, *P* = 0.03) and booster (RR 1.84, 95% CI 1.20–2.81, *P* = 0.005) vaccination. The risk of adverse reactions was significantly increased after the first and second doses, but there was no significant difference between the first two doses (RR 1.00, 95%CI 0.99–1.02, *P* = 0.60). Nevertheless, the two-dose regimen is obviously superior to the single-dose schedule for immunogenicity and efficacy. After booster vaccination, both neutralizing antibodies (RR 144.80, 95%CI 44.97–466.24, *P* < 0.00001) and RBD-binding antibodies (RR 101.50, 95%CI 6.44–1,600.76, *P* = 0.001) reach optimal levels, but the cellular immune response seemed not to be further enhanced. In addition, compared with younger children, older children and adolescents were at significantly increased risk of adverse reactions after vaccination, with either mRNA or inactivated vaccines, accompanied by a stronger immune response.

**Conclusion:**

The available evidence suggests that the safety, immunogenicity and efficacy of COVID-19 vaccines are acceptable in people aged 3–17 years. However, there is an urgent need for additional multicenter, large-sample studies, especially in younger children under 3 years of age and even in infants, with long-term follow-up data.

**Systematic Review Registration:**

https://www.crd.york.ac.uk/prospero/display_record.php?ID=CRD42021290205, identifier: CRD42021290205.

## Introduction

It is the epidemic of coronavirus disease 2019 (COVID-19) that has placed a heavy burden on people worldwide, both physically and mentally (1, 2). In order to control the epidemic, various types of COVID-19 vaccines have sprung up around the world, but the vast majority have only been approved for adults (3). However, with the prevalence of the Omicron variant, a highly divergent variant of syndrome coronavirus 2 (SARS-CoV-2), immune protection for adolescents, children and even infants seems to be imminent. Following the approval of CoronaVac for children aged 3–17 years, the BNT162b2-mRNA vaccine was urgently approved for children aged 5 years and older on November 2, 2021 (4). The sequential authorization of two different vaccines announces that the focus of vaccination is gradually shifting to younger children, as the fight against the epidemic progresses, which not only helps protect children's health and interrupt community epidemics but also promotes educational equity and economic recovery (5).

Compared with adults, teenagers and children infected with SARS-CoV-2 generally present with milder symptoms (6, 7). Therefore, the benefits of COVID-19 vaccines may not be as pronounced in this group as in adults (8). However, the possibility of critical illnesses, such as multisystemic inflammatory syndrome in children (MIS-C) (9), cannot be ruled out in this population, especially in those with underlying disease (10). Moreover, if left unchecked, this population has the potential to become a transit reservoir for SARS-CoV-2, leading to widespread community epidemics (11–13). Furthermore, vaccination helps promote regular back-to-school education (14), which not only prevents online instructions from becoming a barrier to education for poor students, but also removes the worry of working parents (5). In addition, maintaining good social activities also contributes to good psychological growth and sound character building in young children (15).

Advancing the childhood vaccination process should begin by eliminating parent's vaccination hesitancy. However, it is parental doubts about the safety, efficacy, and necessity of vaccinations that are holding back the process (16–19). After all, although a large number of vaccines have been shown to be safe and effective in adults, including the elderly (20–27), there is still a gap in research data for people under the age of 18. Considering the limited available clinical evidence and the urgency of advancing the vaccination process, we plan to conduct a meta-analysis based on existing randomized controlled trials (RCTs), to comprehensively evaluate the safety, immunogenicity, and efficacy of various COVID-19 vaccines in adolescents, children, and even infants.

## Methods

The systematic review and meta-analysis was conducted following the Preferred Reporting Items for Systematic Reviews and Meta-Analyses (PRISMA) guidelines (28), with a study protocol registered in the International Prospective Register of Systematic Evaluations database (CRD42021290205).

### Search Strategy

We conducted a comprehensive search in Pubmed, Embase, and Cochrane Library databases from inception to November 9, 2021, using “COVID-19 Vaccines,” “SARS-CoV-2,” “COVID-19,” “Adolescent,” “Child,” “Infant” and “Randomized controlled trial” as medical subject headings (MeSH) terms. The search details can be found in the Supplementary Material. The search in the clinical trials registers (Clinical Trials.gov, an ongoing NIH trial registry) was also performed to find potentially available studies. The electronic database search was additionally supplemented by a manual search of the reference lists of relevant systematic reviews and key articles.

### Study Selection

Only randomized controlled trials were eligible, with no restrictions on language or publication status; cohort studies, case-control studies, single-arm studies, cross-sectional studies, case reports, reviews, comments, and letters were all excluded. These RCTs were conducted in healthy humans aged 0–17 years, with various types of COVID-19 vaccines as interventions, and placebo, adjuvant, or other vaccines as controls. The following statistical information should be provided as outcome indicators: (1) the incidence of adverse events after vaccination, including total adverse reactions, local adverse reactions, systemic adverse reactions, and any specific adverse reactions, (2) humoral immune responses, including the seroconversion after vaccination, (3) cellular immune responses, such as IFN-γ enzyme-linked immunospot, (4) incidence of confirmed COVID-19 post-vaccination. After removing duplicate records, two review authors (YD and LC) independently assessed the titles and abstracts of all records, and then conducted a full-text review with predetermined criteria. Disagreements were resolved by consulting a third author (YS).

### Data Extraction

Specific bibliographic software EndNote X9 was used to manage the literature. Using a pre-developed data extraction form in Microsoft Excel, two authors independently extracted the following data: name of the first author, date of publication, study protocol, baseline characteristics of participants, sample size, intervention details, and outcome indicators. The seroconversion was defined as at least a fourfold increase in geometric mean titres (GMT) from baseline after vaccination. A secondary case definition of COVID-19 was also adopted, according to which patients were diagnosed with COVID-19 as long as they were positive for SARS-CoV-2 by RT-PCR and accompanied by one or more associated symptoms. In order to avoid missing data as much as possible, we carefully read the original text and supplementary materials of the included studies. If the original article grouped vaccinees according to age, dose of vaccination, etc., we would combine the data for each subgroup. If the original article did not provide the data in the form we expected, the required data would be calculated manually based on the information provided. When the required dichotomous variables were provided in the form of totals and percentages, we would obtain the available data by calculating the product. When the original text did not provide the information we needed, we attempted to obtain the corresponding information from the supplementary material. Considering the limited time, we did not contact the corresponding author to obtain the original data. In case of any disagreement, consensus would be reached through discussion or consultation with a third authors (YS).

### Risk of Bias Assessment and Evidence Quality Assessment

To evaluate the methodological quality of the studies, two reviewer (YD and LC) independently assessed the risk of each study according to the Cochrane collaboration tool for assessing the risk of bias (Rob) (29). In order to appraise the quality and certainty of the evidence, these two authors (YD and LC) also assessed the reliability of the primary results by Gradepro 3.6 software, according to the Grades of Recommendation, Assessment, Development, and Evaluation (GRADE) standard. Any differences in the assessment process would be resolved by consulting the third reviewer (YS). Considering that the currently available literature may be limited, we would pool all studies regardless of quality.

### Data Synthesis and Analysis

We used RevMan 5.3 statistical software to pool dichotomous outcomes, with the risk ratio (RR) and its 95% confidence interval (CI) as the effect measures. RR > 1 implies a higher risk in the observation group, and *P* < 0.05 indicates that this difference is statistically significant. The *I*^2^ statistic was used to estimate the level of heterogeneity, and significant heterogeneity was considered when the *I*^2^ value was >50% (30). Following the recommendations of the Cochrane Handbook (29) and taking into account the different characteristics of the included studies (31), all data would be pooled by using random-effects models, regardless of the heterogeneity. However, if there were <5 studies available, the random-effects model would no longer be applicable. In this case, the fixed-effects model would be chosen to pool the data. To trace the source of heterogeneity, we performed sensitivity analyses by excluding pooled studies one by one. Furthermore, subgroup analyses were conducted according to the number of vaccinations, type of vaccines, age of the recipients, and specific adverse reactions. When appropriate, direct comparisons were also conducted between prime and boost vaccinations, as well as among different ages. In addition, if ten or more RCT studies were eventually included, the funnel plot analysis of the primary outcome was planned to assess publication bias (32).

## Results

### Characteristics of the Included Studies

**Figure 1:**
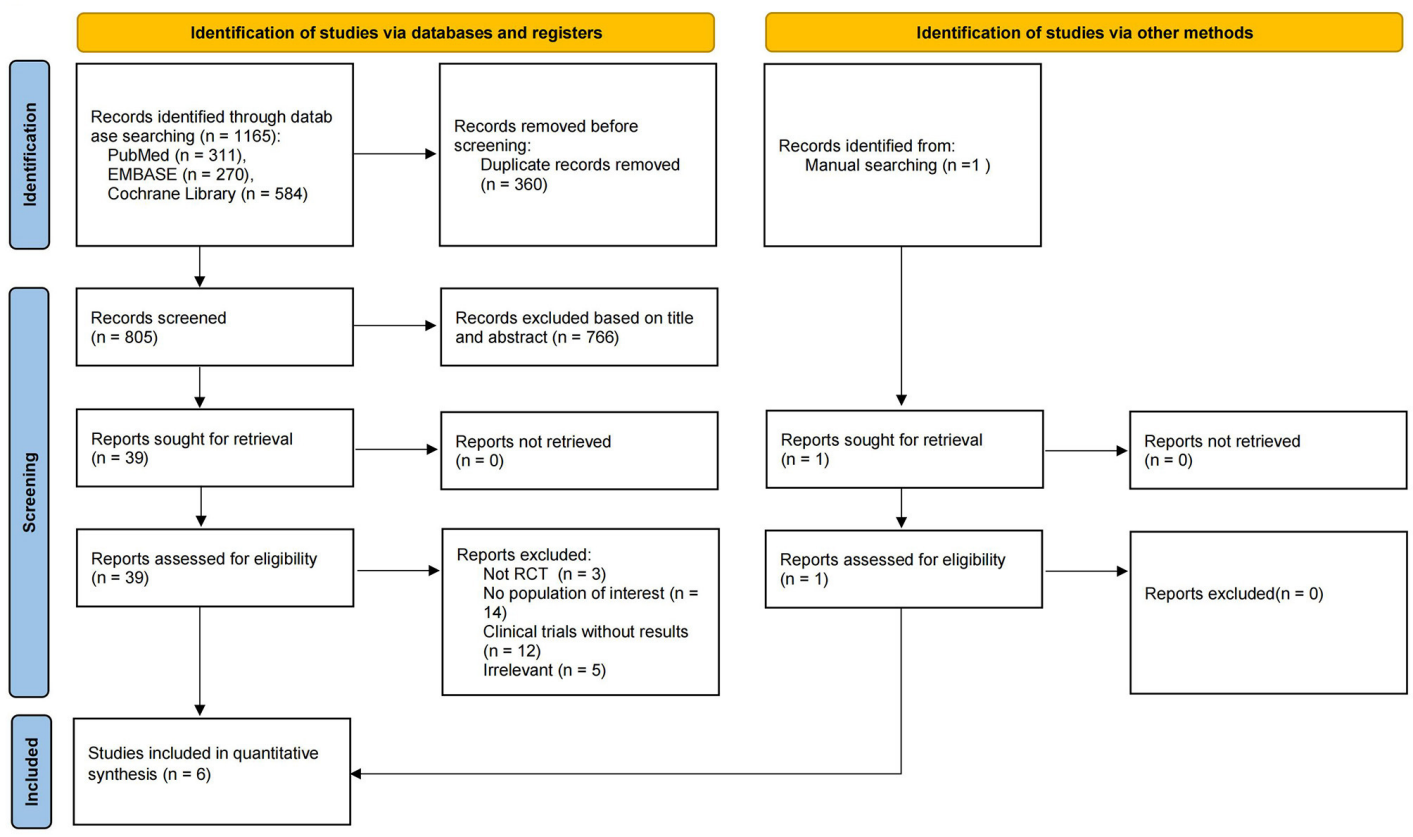
Flow chart of study identification and selection.

**Figure 2:**
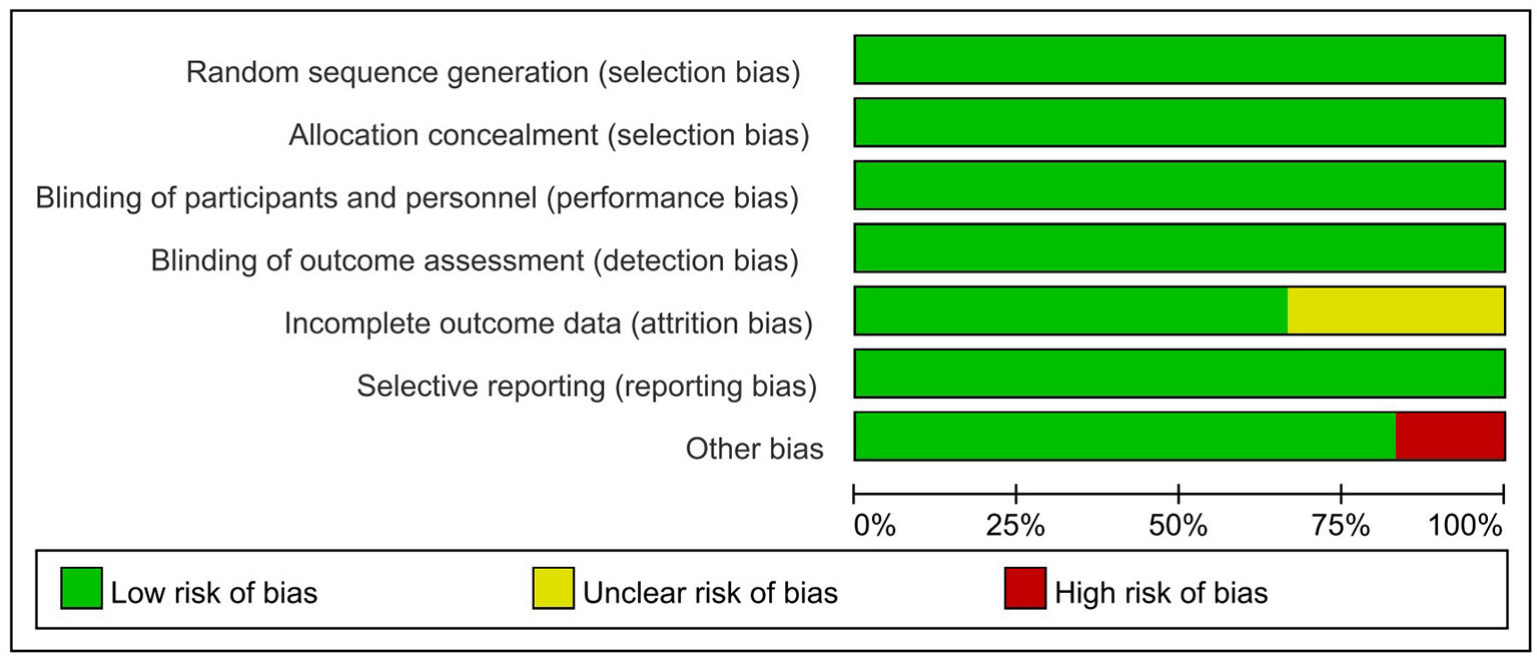
Risk of bias graph for included RCTs.

**Figure 3:**
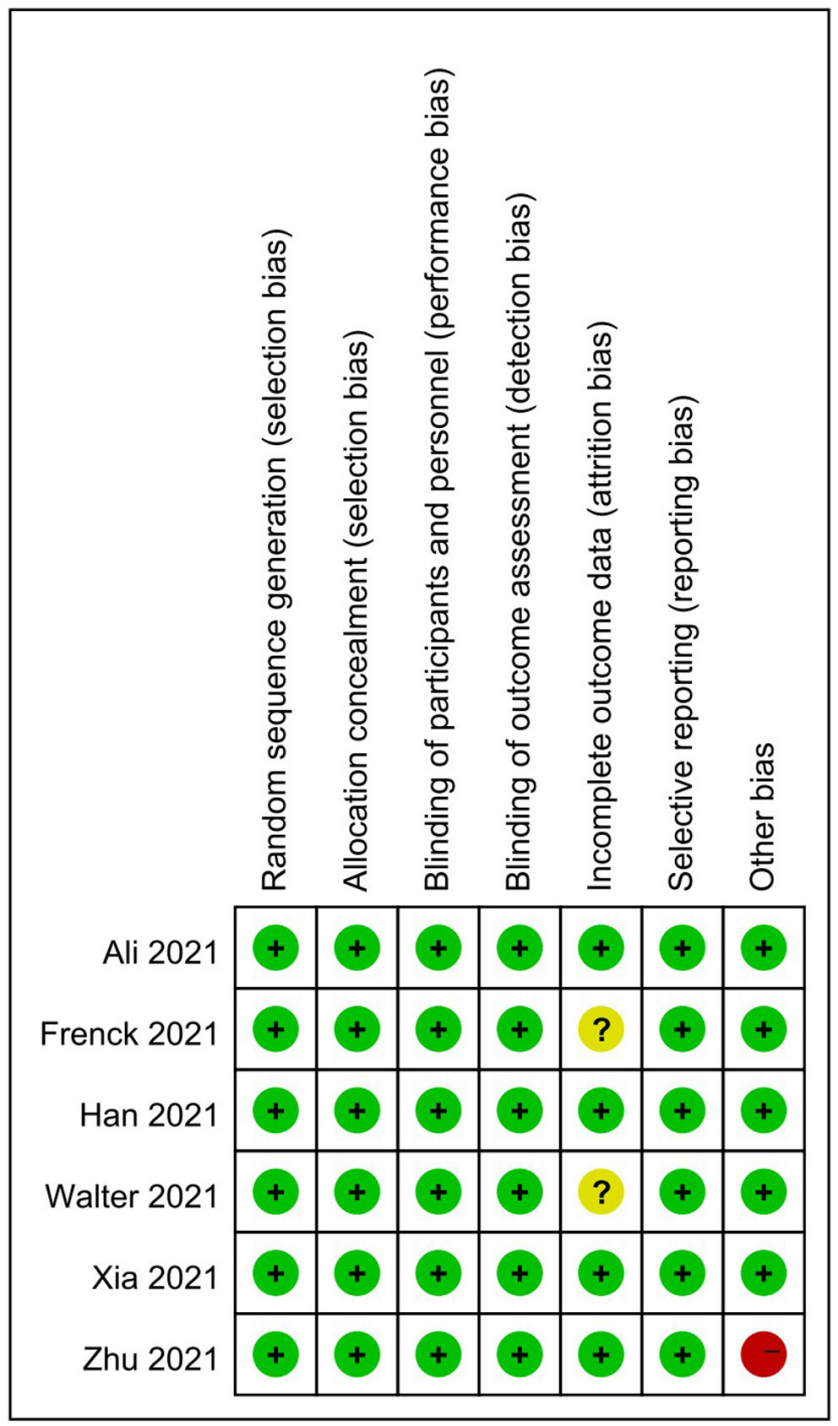
Risk of bias summary for included RCTs.

In this meta-analysis, a total of 1,166 citations were retrieved, and after removing duplicates, we screened 805 records based on title and abstract, of which 766 were determined to be irrelevan. The remaining 40 articles were evaluated in full text and 34 of them were excluded for various reasons. Finally, a total of 6 studies were included (33–38), of which 1 trial was identified by manual search. No relevant trials providing available outcome indicators were found on ClinicalTrials.gov. The flow chart for identifying and selecting the studies was presented in Figure 1. These six RCTs included three types of COVID-19 vaccines, with mRNA vaccines being the most studied (60%) (33–35), followed by inactivated vaccines (40%) (35, 36) and adenoviral vector vaccines (20%) (38), all with saline or aluminum hydroxide adjuvants as controls. A total of 9,962 participants were enrolled, ranging in age from 3 to 17 years old. All participants received a two-dose injection, except for the vaccinees in one RCT (37), who received a three-dose regimen. In the two RCTs studying inactivated vaccines (36, 37), investigators grouped subjects according to age and the dose of vaccine administered. The characteristics of the included studies were summarized in Table 1. Overall, the risk of bias in these studies was low, with the main risk factors being incomplete outcome data and other biases, as shown in detail in Figures 2, 3.

**Table 1:** The characteristics of the included studies.

**References**	**Clinical trials registration**	**Phase**	**Age range**	**Type of vaccine**	**Dose of administration**	**Number of scheduled doses (time of inoculations)**	**Control**	**Number of observation group**	**Number of control group**
Ali et al. (33)	NCT04649151	Phase 2/3	12–17	mRNA-1273 vaccine (mRNA vaccine)	100 μg/dose	Prime and boost inoculation (0, 28 days)	Saline	2,486	1,240
Frenck et al. (34)	NCT04368728	Phase 3	12–15	BNT162b2 Covid-19 Vaccine (mRNA vaccine)	30 μg/dose	Prime and boost inoculation (0, 21 days)	Saline	1,131	1,129
Walter et al. (35)	NCT04816643	Phase 2/3	5–11	BNT162b2 Covid-19 Vaccine (mRNA vaccine)	10 μg/dose	Prime and boost inoculation (0, 21 days)	Saline	1,518	750
Han et al. (36)	NCT04551547	Phase 1/2	3–17 **(**3–5; 6–11; 12–17)	CoronaVac (Inactivated vaccine)	1.5 or 3 μg/dose	Prime and boost inoculation (0, 28 days)	Alum	436	114
Xia et al., (37)	ChiCTR2000032459	Phase 1/2	3–17 (3–5; 6–12; 13–17)	BBIBP-COV (Inactivated vaccine)	2 ug, 4 ug or 8 μg/dose	Three doses (0, 28, and 56 days)	Saline and aluminum hydroxide adjuvant	756	252
Zhu et al. (38)	NCT04566770	Phase 2	6–17	Ad5-vectored COVID-19 vaccine (Adenovirus vaccine)	0.3 ml/dose	Prime and boost inoculation (0, 56 days)	Placebo containing the same excipients as the vaccine, without viral particles	100	50

### Safety of COVID-19 Vaccines

A total of six RCTs (33–38) evaluated possible adverse reactions after the first and second doses. Only four RCTs (33, 36–38) provided data on total adverse reactions, while all six RCTs reported the occurrence of specific adverse reactions after vaccination. Walter et al. did not provide the exact number of participants in the placebo group in the safety analysis. By reading the original article (35), we only know that there were 748 or 749 children in the placebo group after the first dose, and 740 or 741 children in the placebo group after the second dose. Nevertheless, after data analysis, it was found that the effect of small changes in this data on the results was largely negligible. Therefore, we still included this RCT.

The data showed that the risk of unsolicited (RR 1.21, 95%CI 1.07–1.36, *P* = 0.002; Supplementary Figure 1, Table 2) adverse reactions was significantly higher in the vaccine group than in the control group, within 28 or 30 days after the whole vaccination procedure. However, for severe (RR 2.35, 95%CI 0.78–7.03, *P* = 0.13), and even life-threatening (RR 1.00, 95%CI 0.06–15.94, *P* = 1.00) unsolicited adverse reactions, there was no significant difference between the two groups. No case reports of death, multisystem inflammatory syndrome in children (MIS-C), myocarditis, or pericarditis disease were found in any individual RCT.

**Table 2:** Overall adverse reactions and unsolicited adverse reactions within 28 or 30 days after whole vaccination procedure in inactivated vaccine group vs. control group.

	**No. of studies**	**RR (95% CI)**	**I2**	***P*** **-value**
Overall adverse reactions within 28 or 30 days after whole vaccination procedure	2	1.59 [1.26, 2.01]	77	<0.05^*^
**Unsolicited adverse reactions within 28 or 30 days after whole vaccination procedure**				
Overall	4	1.21 [1.07, 1.36]	14	<0.05^*^
Related to study vaccination	3	1.96 [1.59, 2.41]	20	<0.05^*^
Severe	3	2.35 [0.78, 7.03]	0	>0.05
Life-threatening	3	1.00 [0.06, 15.94]	Not applicable	>0.05
Serious	3	1.63 [0.45, 5.88]	0	>0.05
Medically-attended	1	0.96 [0.74, 1.25]	Not applicable	>0.05
Leading to discontinuation	3	2.99 [0.36, 24.93]	0	>0.05

* *P < 0.05*.

#### Adverse Reactions to Different Inoculation Doses

Subgroup analyses of adverse reactions after different number of inoculations were performed. The data showed that the risk of adverse events was statistically higher in the vaccine group than in the control group after the first (RR 1.49, 95%CI 1.43–1.55, *P* < 0.00001; Supplementary Figure 2, Table 3) and second doses (RR 1.76, 95%CI 1.67–1.85, *P* < 0.00001; Supplementary Figure 2, Table 3), but no significant differences were found between the first and second dose groups (RR 1.00, 95%CI 0.99–1.02, *P* = 0.60; Supplementary Figure 3, Table 4). Only one RCT (37) assessed possible local (RR 1.86, 95%CI 0.55–6.30, *P* = 0.32; Supplementary Figure 2, Table 3) and systemic (RR 2.30, 95%CI 0.69–7.64, *P* = 0.17; Supplementary Figure 2, Table 3) adverse reactions after the third dose, and showed no significant difference between the two groups.

**Table 3:** Total adverse reactions in vaccination group vs. control group.

		**No. of studies**	**RR (95% CI)**	**I2**	***P*** **-value**
After dose 1	Total adverse reactions	3	1.49 [1.43, 1.55]	70	<0.05^*^
	Local adverse reactions	3	2.60 [2.42, 2.80]	47	<0.05^*^
	Systemic adverse reactions	3	1.26 [1.19, 1.33]	68	<0.05^*^
After dose 2	Total adverse reactions	3	1.76 [1.67, 1.85]	60	<0.05^*^
	Local adverse reactions	3	2.89 [2.67, 3.14]	8	<0.05^*^
	Systemic adverse reactions	3	1.88 [1.77, 2.01]	29	<0.05^*^
After dose 3	Total adverse reactions	0	/	/	/
	Local adverse reactions	1	1.86 [0.55, 6.30]	Not applicable	>0.05
	Systemic adverse reactions	1	2.30 [0.69, 7.64]	Not applicable	>0.05

* *P < 0.05*.

**Table 4:** Total and specific reactions in vaccination group after dose 1 vs. after dose 2.

	**No. of studies**	**RR (95% CI)**	**I2**	***P*** **-value**
**Overall**				
Total adverse reactions	3	1.00 [0.99, 1.02]	90	>0.05
Local adverse reactions	3	1.02 [1.00, 1.04]	82	<0.05^*^
Systemic adverse reactions	3	0.83 [0.81, 0.86]	96	<0.05^*^
**Overall**	6	0.73 [0.71, 0.74]	97	>0.05
Local pain	6	1.02 [1.00, 1.04]	73	*P* = 0.05
Erythema/ Redness	5	0.70 [0.62, 0.79]	0	<0.05^*^
Induration	1	2.00 [0.18, 21.71]	Not applicable	>0.05
Pruritus/ Itch	3	1.15 [0.39, 3.41]	0	>0.05
Swelling	6	0.79 [0.70, 0.89]	0	<0.05^*^
Axillary Swelling	1	1.11 [1.00, 1.23]	Not applicable	*P* = 0.05
Fever	6	0.44 [0.37, 0.53]	95	<0.05^*^
Cough	3	1.76 [0.99, 3.12]	0	*P* = 0.05
Oropharyngeal pain	1	3.00 [0.32, 28.35]	Not applicable	>0.05
Headache	6	0.65 [0.62, 0.69]	65	<0.05^*^
Fatigue	6	0.72 [0.69, 0.76]	39	<0.05^*^
Myalgia	6	0.59 [0.55, 0.64]	39	<0.05^*^
Arthralgia	4	0.52 [0.47, 0.58]	0	<0.05^*^
Nausea/ vomiting	1	0.47 [0.42, 0.54]	Not applicable	<0.05^*^
Nausea	3	1.24 [0.49, 3.11]	0	>0.05
Vomiting	5	1.26 [0.58, 2.78]	0	>0.05
Diarrhea	4	1.45 [0.72, 2.94]	0	>0.05
Anorexia	2	1.81 [0.68, 4.83]	32	>0.05
Chills	3	0.44 [0.40, 0.48]	41	<0.05^*^
Pruritus (systemic adverse reaction)	1	3.00 [0.12, 72.77]	Not applicable	>0.05
Acute allergic reaction/ Hypersensitivity	1	0.33 [0.01, 8.13]	Not applicable	>0.05
Abnormal skin and mucosa	1	2.92 [0.31, 28.00]	Not applicable	>0.05
Dysphagia	1	0.33 [0.01, 8.09]	Not applicable	>0.05

* *P < 0.05*.

#### Adverse Reactions to Different COVID-19 Vaccines

Considering the relatively high statistical heterogeneity in the above analysis (*I*^2^ from 8 to 70%), we further performed subgroup analyses according to different vaccine types. The data showed a significantly increased risk of total, local, and systemic adverse reactions after vaccination both in the mRNA vaccine group and in the adenovirus vector vaccine group, however, in the inactivated vaccine group, only the risk of local reactions after initial vaccination was significantly higher than in the control group (RR 6.34, 95%CI 1.54–26.10, *P* = 0.01; Supplementary Figure 4, Table 5).

**Table 5:** Adverse reactions among vaccination group vs. control group.

		**No. of studies**	**RR (95% CI)**	**I2**	***P*** **-value**
**Total adverse reactions**					
After dose 1	Overall	3	1.49 [1.43, 1.55]	70	<0.05^*^
	mRNA vaccine	1	1.47 [1.41, 1.54]	Not applicable	<0.05^*^
	Inactivated vaccine	1	1.27 [0.76, 2.13]	Not applicable	>0.05
	Vectored vaccine	1	3.44 [1.78, 6.65]	Not applicable	<0.05^*^
After dose 2	Overall	3	1.76 [1.67, 1.85]	60	<0.05^*^
	mRNA vaccine	1	1.74 [1.66, 1.83]	Not applicable	<0.05^*^
	Inactivated vaccine	1	1.83 [0.90, 3.72]	Not applicable	>0.05
	Vectored vaccine	1	8.25 [2.06, 33.00]	Not applicable	<0.05^*^
After dose 3	Overall	0	/	/	/
	mRNA vaccine	0	/	/	/
	Inactivated vaccine	0	/	/	/
	Vectored vaccine	0	/	/	/
**Local adverse reactions**					
After dose 1	Overall	3	2.60 [2.42, 2.80]	47	<0.05^*^
	mRNA vaccine	1	2.56 [2.38, 2.76]	Not applicable	<0.05^*^
	Inactivated vaccine	1	6.34 [1.54, 26.10]	Not applicable	<0.05^*^
	Vectored vaccine	1	6.00 [1.94, 18.53]	Not applicable	<0.05^*^
After dose 2	Overall	3	2.89 [2.67, 3.14]	8	<0.05^*^
	mRNA vaccine	1	2.86 [2.64, 3.10]	Not applicable	<0.05^*^
	Inactivated vaccine	1	4.29 [1.03, 17.96]	Not applicable	P=0.05
	Vectored vaccine	1	19.69 [1.21, 319.62]	Not applicable	<0.05^*^
After dose 3	Overall	1	1.86 [0.55, 6.30]	Not applicable	>0.05
	mRNA vaccine	0	/	/	/
	Inactivated vaccine	1	1.86 [0.55, 6.30]	Not applicable	>0.05
	Vectored vaccine	0	/	/	/
**Systemic adverse reactions**					
After dose 1	Overall	3	1.26 [1.19, 1.33]	68	<0.05^*^
	mRNA vaccine	1	1.23 [1.17, 1.31]	Not applicable	<0.05^*^
	Inactivated vaccine	1	1.32 [0.87, 2.00]	Not applicable	>0.05
	Vectored vaccine	1	3.70 [1.55, 8.83]	Not applicable	<0.05^*^
After dose 2	Overall	3	1.88 [1.77, 2.01]	29	<0.05^*^
	mRNA vaccine	1	1.87 [1.76, 1.99]	Not applicable	<0.05^*^
	Inactivated vaccine	1	1.61 [0.76, 3.40]	Not applicable	>0.05
	Vectored vaccine	1	6.00 [1.48, 24.38]	Not applicable	<0.05^*^
After dose 3	Overall	1	2.30 [0.69, 7.64]	Not applicable	>0.05
	mRNA vaccine	0	/	/	/
	Inactivated vaccine	1	2.30 [0.69, 7.64]	Not applicable	>0.05
	Vectored vaccine	0	/	/	/

* *P < 0.05*.

Detailed analyses were conducted for specific adverse events after vaccination. In the mRNA vaccine group, the risk of adverse reactions such as pain, swelling, and fever were significantly higher, both after initial vaccination and booster vaccination (Supplementary Figure 5, Supplementary Table 1). In the inactivated vaccine group, only the risk of local pain was significantly higher, and the risk of all other known adverse reactions was not significantly different compared with the control group (Supplementary Figure 6, Supplementary Table 1). For the adenovirus vector vaccine, there was no significant difference in the risk of adverse reactions compared with placebo, except for a significantly higher risk of local pain (RR 5.67, 95%CI 1.83–17.55, *P* = 0.003; Supplementary Figure 7, Supplementary Table 1) and fever after (RR 7.00, 95%CI 1.74–28.21, *P* = 0.006; Supplementary Figure 7, Supplementary Table 1) the first dose. After pooling all available data on specific reactions, the risk was significantly higher in all vaccine groups than in the control group, but relatively lower in the inactivated vaccine group, both after initial vaccination (RR 1.40, 95% CI 1.04–1.90, *p* = 0.03) and after booster vaccination (RR 1.84, 95% CI 1.20–2.81, *p* = 0.005) (Supplementary Table 1).

#### Adverse Reactions in Different Age Groups

When subgroup analysis was performed according to different vaccine types, the data showed that heterogeneity remained generally high in the mRNA vaccine group, but lower heterogeneity could be found in most subgroups after removing the RCT study by Walter et al. (35). Considering that the RCT by Walter et al. targeted younger children aged 5–11 years, whereas the other two RCTs studying mRNA vaccines (33, 34) were conducted in children and adolescents aged 12 years and older, we decided to perform further subgroup analyses for specific adverse reactions depending on the different ages of mRNA vaccine recipients (Supplementary Figure 8, Supplementary Table 2). For older children aged 12–17 years, the risk of all adverse reactions after vaccination was significantly higher, except for systemic reactions such as vomiting and diarrhea. As for younger children aged 5–11 years, the risk of headache (RR 0.45, 95%CI 0.26–0.80, *P* = 0.007) and fatigue (RR 0.54, 95%CI 0.34–0.88, *P* = 0.01) after the first dose as well as the risk of diarrhea (RR 0.10, 95%CI 0.03–0.36, *P* = 0.0003) after booster vaccination were even significantly lower; but for other adverse reactions, there was no statistical difference between the two groups.

Overall, the risk of various adverse reactions after mRNA vaccination appears to be higher in older children aged 12–17 years than in younger children aged 5–11 years. Considering that both Frenck et al. (34) and Walter et al. (35) chose the mRNA-1273 vaccine as the intervention, we decided to directly compare the occurrence of various adverse reactions following mRNA-1273 vaccination in older and younger children (Supplementary Figure 9, Table 6). The data showed a significantly higher risk of various adverse reactions in participants aged 12–15 years, both after the initial (RR 1.40, 95%CI 1.21–1.62, *P* < 0.00001) and the booster (RR 2.04, 95%CI 1.75–2.38, *P* < 0.00001) vaccination, suggesting that the mRNA-1273 vaccine may have a greater safety profile in young children aged 5–11 years.

**Table 6:** Specific adverse reactions in mRNA vaccine recipients aged ≥12 years vs. <12 years.

		**No. of studies**	**RR (95% CI)**	**I2**	***P*** **-value**
**After dose 1**	Overall	2	1.40 [1.21, 1.62]	71	<0.05^*^
	Local pain	2	2.09 [1.56, 2.81]	93	<0.05^*^
	Erythema or Redness	2	1.77 [0.77, 4.03]	45	>0.05
	Swelling	2	2.72 [0.95, 7.74]	26	>0.05
	Fever	2	5.12 [1.25, 21.01]	36	<0.05^*^
	Headache	2	1.04 [0.75, 1.43]	92	>0.05
	Fatigue	2	1.00 [0.75, 1.34]	90	>0.05
	Myalgia	2	1.34 [0.78, 2.29]	67	>0.05
	Arthralgia	2	0.87 [0.41, 1.85]	69	>0.05
	Vomiting	2	1.85 [0.38, 9.07]	0	>0.05
	Diarrhea	2	0.97 [0.44, 2.12]	0	>0.05
	Chills	2	1.87 [1.05, 3.36]	82	<0.05^*^
**After dose 2**	Overall	2	2.04 [1.75, 2.38]	77	<0.05^*^
	Local pain	2	2.21 [1.62, 3.02]	93	<0.05^*^
	Erythema or Redness	2	2.28 [0.95, 5.48]	0	>0.05
	Swelling	2	2.97 [1.03, 8.57]	0	<0.05^*^
	Fever	2	10.52 [2.68, 41.29]	32	<0.05^*^
	Headache	2	1.69 [1.20, 2.38]	92	<0.05^*^
	Fatigue	2	1.60 [1.16, 2.22]	92	<0.05^*^
	Myalgia	2	2.30 [1.31, 4.01]	84	<0.05^*^
	Arthralgia	2	1.86 [0.88, 3.92]	74	>0.05
	Vomiting	2	1.85 [0.38, 9.05]	0	>0.05
	Diarrhea	2	0.53 [0.25, 1.13]	89	>0.05
	Chills	2	3.93 [2.11, 7.33]	80	<0.05^*^

* *P < 0.05*.

Two RCTs on inactivated vaccines (CoronaVac (36), BBIBP-COV (37)) both reported total adverse reactions in children of different ages within 28 days after the whole vaccination procedure, so subgroup analysis was performed according to the age of the participants (Supplementary Figure 10, Table 7). The data showed that the risk of adverse reactions was higher in all inactivated vaccine subgroups than in all control groups, especially in the 6-11/12 age group (RR 2.41, 95%CI 1.37–4.23, *P* = 0.002); however, the difference was not statistically significant in the 3–5 age group (RR 1.15, 95%CI 0.81–1.64, *P* = 0.43). Notably, participants in one RCT study (36) received a total of 2 doses of vaccine, whereas participants in the other RCT study (37) received a total of 3 doses of vaccine. However, it was not possible to specifically analyze the safety of inactivated vaccines after a single dose, because Han et al. (36) did not provide information on adverse reactions within 28 days after a single dose. In addition, there were minor differences in the grouping methods of the two RCTs, with one (36) grouping vaccinees into age groups of 3–5, 6–11, and 12–17 years, while the other (37) grouping participants into age groups of 3–5, 6–12, and 13–17 years. Overall, the risk of adverse reactions following inactivated vaccination was more noteworthy in older children than in younger children, which is generally consistent with the results of subgroup analyses of mRNA vaccines.

**Table 7:** Overall adverse reactions within 28 days after whole vaccination procedure in inactivated vaccine group of different ages vs. control group.

	**No. of studies**	**RR (95% CI)**	**I2**	***P*** **-value**
Overall adverse reactions within 28 days after whole vaccination procedure	2	1.60 [1.27, 2.01]	57	<0.05^*^
3–5 years old	2	1.15 [0.81, 1.64]	28	>0.05
6–11/12 years old	2	2.41 [1.37, 4.23]	83	<0.05^*^
12/13–17 years old	2	1.71 [1.19, 2.46]	0	<0.05^*^

* *P < 0.05*.

Since only one RCT (38) chose the adenovirus vector vaccine as an intervention, and no data were available for different age groups, further subgroup analysis could not be performed for the adenovirus vector vaccine.

#### Adverse Reactions in Different Doses of Vaccines

Two RCTs (36, 37) provided information on recipients aged 3–17 years after receiving different doses of inactivated vaccine, but subgroup analyses failed to be performed on this basis because the vaccine doses differed in the two RCTs. However, data from both RCTs suggested acceptable safety and tolerability profiles for various doses of inactivated vaccines.

### Immunogenicity

#### Humoral Immune Responses

Three RCTs (36–38) provided data on seroconversion, and the data showed that the seroconversion after inoculation was significant, especially after the second dose (RR 144.80, 95%CI 44.97–466.24, *P* < 0.00001; Supplementary Figure 11, Table 8). Notably, although participants reported by Xia et al. (37) received a total of three doses of adenoviral vector vaccine, their serological response rate had reached 100% at day 56 (28 days after the second dose).

**Table 8:** Seroconversion rate in vaccine group vs. control group.

	**No. of studies**	**RR (95% CI)**	**I2**	***P*** **-value**
**Pseudovirus neutralizing antibody**				
28 days after Dose 1	3	77.99 [28.40, 214.14]	82	<0.05^*^
28 days after Dose 2	3	144.80 [44.97, 466.24]	73	<0.05^*^
Neutralizing antibody 28 days after Dose 2	2	118.74 [38.67, 364.63]	0	<0.05^*^
3-5 years old	2	110.57 [15.87, 770.57]	0	<0.05^*^
6–11/12 years old	2	124.37 [17.79, 869.21]	0	<0.05^*^
12/ 13–17 years old	2	121.28 [17.36, 847.06]	0	<0.05^*^
				
**RBD–binding enzyme-linked immunosorbent assay antibody**				
28 days after Dose 1	1	99.48 [6.31, 1569.12]	Not applicable	<0.05^*^
56 days after Dose 1 (Before Dose 2)	1	98.47 [6.24, 1553.30]	Not applicable	<0.05^*^
28 days after Dose 2	1	101.50 [6.44, 1600.76]	Not applicable	<0.05^*^

* *P < 0.05*.

In addition, given that Han et al. (36) and Xia et al. (37) both provided seroconversions for each age group at 28 days post-vaccination, a subgroup analysis was performed accordingly. The data showed a significant humoral immune response to SARS-CoV-2 after inactivated vaccination in all age groups, but the response appears to be relatively low in children aged 3–5 years (RR 110.57, 95%CI 15.87–770.57, *P* < 0.00001; Supplementary Figure 11, Table 8). Moreover, Han et al. (36) and Xia et al. (37) also provided data for different doses, which may suggest dose-dependent immunogenicity. Han et al. (36) indicated that the neutralizing antibody titer induced by the 3.0 μg dose group was obviously higher than that of the 1.5 μg dose group after boost vaccination (*P* < 0.05). Similarly, it was reported by Xia et al. (37) that the 4 and 8 μg dose groups elicited significantly higher antibody responses compared with the 2 μg dose group (*P* < 0.05).

Three other RCTs (33–35) with mRNA vaccine as the intervention compared immune responses 1 month after booster vaccination in vaccinees and young adults (16 or 18 years of age and older), and assessed non-inferiority by calculating the geometric mean ratio (GMR) with its 95% confidence interval. Ali et al. (33) reported the GMT of 1401.7 (95% CI: 1276.3, 1539.4) in adolescents aged 12–17 years, with a neutralizing antibody GMR of 1.08 (95% CI: 0.94 to 1.24) relative to young adults aged 18 to 25 years, meeting the non-inferiority criterion (i.e., lower limit of the two-sided 95% confidence interval > 0.67). As reported by Frenck et al. (34) and Walter et al. (35), the GMRs of neutralizing antibodies in adolescents aged 12 to 15 years and children aged 5–11 years to young adults aged 16 to 25 years were respectively 1.76 (95% CI: 1.47–2.10), and 1.04 (95% CI: 0.93–1.18), which met the criteria of non-inferiority as well. In particular, the immune response to BNT162b2 Covid-19 vaccine might be greater in adolescents aged 12 to 15 years than in young adults aged 16 to 25 years, because the lower limit of the two-sided 95% confidence interval for the GMR is >1.

There were also two RCT studies (33, 38) evaluating the receptor binding domain (RBD)-binding ELISA antibody. The results of Ali et al. (33) showed a GMR of 1.09 (95% CI: 0.94–1.26) for RBD-binding ELISA antibodies in adolescents aged 12–17 years relative to young adults aged 16–25 years, while in the trial of Zhu et al. (38), the seroconversion rate of RBD-binding antibodies in the vaccine group reached 98%(RR 99.48, 95%CI 6.31–1,569.12, *P* = 0.001) and 100%(RR 101.50, 95%CI 6.44–1,600.76, *P* = 0.001) at day 28 after initial and booster vaccination, respectively (Supplementary Figure 11, Table 8).

#### Cellular Immune Responses

There was only one RCT (38) evaluating the potential of vaccines to induce specific cellular responses. It was reported that significant specific T-cell responses, particularly Th 1 cell responses, were induced after initial adenoviral vector vaccination, but the intensity of immunity appeared to diminish after booster vaccination.

### Efficacy

Three RCTs (33–35) with mRNA vaccine as an intervention assessed vaccine efficacy, which was at 100.0% (95% CI: 28.9%-NE%), 100% (95% CI: 75.3%–100%), and 90.7% (95% CI: 67.4%–98.3%), respectively. Both types of mRNA vaccines provided satisfactory prevention against COVID-19, especially the BNT162b2 Covid-19 vaccine for adolescents aged 12 years and older (RR 0.03, 95%CI 0.00–0.44; Supplementary Figure 12, Table 9) (34). Other RCT studies (36–38) with inactivated vaccine or adenovirus vector vaccine as interventions did not evaluate the vaccine efficacy.

**Table 9:** COVID-19 diagnosed after vaccination in vaccine group vs. control group.

	**No. of studies**	**RR (95% CI)**	**I2**	***P*** **-value**
**Covid-19 after the vaccination**	3	0.10 [0.05, 0.21]	0	<0.05^*^
After dose 1 to before dose 2	1	0.25 [0.07, 0.88]	Not applicable	<0.05^*^
Within 7 days after the second dose	1	0.09 [0.01, 1.64]	Not applicable	>0.05
7 days after second dose	2	0.06 [0.02, 0.20]	0	<0.05^*^
14 days after second dose	1	0.07 [0.01, 0.56]	Not applicable	<0.05^*^
**Covid-19 after dose** 2	3	0.06 [0.02, 0.18]	0	<0.05^*^
mRNA-1273 vaccine	1	0.07 [0.01, 0.56]	Not applicable	<0.05^*^
BNT162b2 Covid-19 Vaccine	2	0.06 [0.02, 0.20]	0	<0.05^*^

* *P < 0.05*.

### Sensitivity Analysis and Publication Bias

Through detailed subgroup analysis, we have tried to minimize the effect of heterogeneity on our results. However, when performing sensitivity analyses, we still found that the heterogeneity of pooled effects for certain outcomes may change substantially after removing individual RCT. Although the changes barely affect our conclusions, it still suggests that the results are not robust enough and need to be viewed with caution. As suggested by the Cochrane Handbook (29), it is well known that assessing publication bias with funnel plots is not reliable when fewer than 10 studies were included (32). It was only a total of 6 RCTs that were included in this meta-analysis, and there were essentially only 3 or fewer papers available for specific outcome indicators. Therefore, given the limited number of available literature, we did not assess the publication bias.

### Grading of Evidence Quality

As shown in the Supplementary Tables 3–6, we assessed the quality of the primary outcomes. Overall, the quality of evidence for most outcomes was moderate and high, with inconsistency as the main downgrading factor.

## Discussion

The risk of various adverse reactions, mainly including local pain, swelling and fever, was increased to varying degrees after different types of vaccination, but they were generally mild and not fatal. There was insufficient evidence to attribute the reported severe adverse events exclusively to vaccination. It was inactivated vaccines that had a higher safety profile compared with mRNA vaccines and adenoviral vector vaccines, and data are available to support the safety and tolerability of inactivated vaccines at different doses. Besides, the risk of adverse reactions occurring after the first two doses was significantly increased, but no significant differences were found between the prime and boost vaccination groups. Relatively speaking, the third dose of vaccine might be safer for vaccinees. Moreover, there were subtle differences in the risk of adverse reactions among different age groups. For older vaccine recipients, adverse reactions caused by mRNA vaccine and inactivated vaccine warrant further attention.

In addition, good immunogenicity could be observed for all vaccine types and, in particular, dose-level-dependent immunogenicity was found in the inactivated vaccine group. The immunogenicity of vaccines varies slightly among age groups. Older children over 12 years of age would develop a stronger immune response after vaccination, especially after BNT162b2 Covid-19 vaccine. This difference may be related to the fact that immune function is not yet well developed in young children. Furthermore, although there was no significant difference in the risk of adverse reactions between single-dose and double-dose vaccines, the double-dose regimen was significantly superior to the single-dose schedule in terms of humoral immunogenicity and prophylactic efficacy. However, data from Zhu et al. (38) showed no further enhancement in the intensity of T-cell immune response after booster vaccination. This result should be viewed with caution due to the limited data on the cellular immune response. What's more, both types of mRNA vaccines have shown satisfactory efficacy in preventing COVID-19, especially the BNT162b2 Covid-19 vaccine applied in adolescents aged 12 years and older.

In general, in this meta-analysis based on RCTs, the safety, immunogenicity, and efficacy of the COVID-19 vaccines were confirmed to some extent in children and teenagers aged 3 to 17 years, but analyses in younger children under 3 years of age and even in infants were lacking. For different vaccine types, inactivated vaccines had better safety profiles significantly; for different injection regimens, double-dose vaccination induced a stronger humoral immune response and produced better prophylactic effects; for different age groups of vaccinees, the vaccine has better immunogenicity in older children, accompanied by a higher risk of adverse reactions; for different doses of inactivated vaccine, there were no significant differences in adverse reactions among different dose groups, but the humoral immune response was more pronounced in the high dose group. If possible, individualized vaccination programs can be considered. Countries can administer the most appropriate COVID-19 vaccine to children and adolescents of different ages in a variety of health conditions, depending on local circumstances.

In addition to six included RCTs, a comprehensive search identified three relevant trials (20, 39, 40) that included adolescents, all of which confirmed good safety and immunogenicity of the vaccine in this age group but were not included in the review because no information was specifically provided for specific age group. Notably, Thomas et al. (40) followed the subjects for 6 months and confirmed that the immune efficacy of the BNT162b2 Covid-19 vaccine, although gradually decreasing over time, could still be maintained at a good level.

To our knowledge, this is the first meta-analysis specifically targeting COVID-19 vaccine recipients under the age of 18 years, which has comprehensively assessed the safety, immunogenicity, and efficacy of COVID-19 vaccines in the population. Previously, Liu et al. published a systematic review (41) evaluating COVID-19 vaccination in children and adolescents, but that review included only two RCTs and did not perform a quantitative analysis. Moreover, those included in this review are all recently published, high-quality randomized controlled trials, that can provide the strongest evidence to date. In addition, to reduce the effect of heterogeneity, we performed a rigorous subgroup analysis to figure more precise and detailed results. However, there are some limitations as well. First of all, we only included a limited number of RCTs, including only three types of COVID-19 vaccines (the mRNA vaccine, inactivated vaccine, and adenovirus vector vaccine), and lacked data on younger children under 3 years of age or even infants, as well as long-term follow-up data. Besides, the RCT (38) with adenoviral vector vaccine as an intervention was a small-sample study, so the data provided may be overridden by other large-sample studies. Although this possibility has been substantially reduced by detailed subgroup analysis, the small sample size may still limit the statistical validity of this trial. Furthermore, for the cellular immune response after vaccination, only one RCT (38) provided relevant data. In addition, although methodological heterogeneity and clinical heterogeneity were well controlled, statistical heterogeneity could not be ignored. Despite the implementation of careful subgroup analyses, high statistical heterogeneity could still be found in some subgroups, which may be related to potential factors such as geographic region, population ethnicity, and vaccine dose.

Regarding vaccination of people under 18 years of age, the following issues remain to be urgently addressed.

To begin with, there is an urgent need to fill the gaps in long-term follow-up data, to assess the duration of immune response after vaccination, and whether vaccines cause long-term adverse outcomes, such as myocarditis. Although the available data (42) suggested that the incidence and long-term risk of myocarditis caused by the virus itself appeared to be more threatening than that of vaccine-associated myocarditis, which might be self-limiting, we still need stronger evidence to dispel this concern. Besides, recent data (43) indicates that inactivated vaccination may cause pathophysiological changes in vaccine recipients similar to those in infected individuals, suggesting that careful consideration is needed when vaccinating children, even with inactivated vaccines that appear to be safer, especially for children with underlying disease. What's more, given that MIS-C may be an immune disease associated with SARS-CoV-2 infection, we cannot exclude the possibility that this complication is instead induced after COVID-19 vaccination (11). Relevant studies are urgently needed to elucidate the mechanism underlying this rare but severe disease (44).

Moreover, assessment of children under 3 years of age and even infants is urgently needed on the agenda. As reported (45), Pfizer may respectively release the results of vaccination trials for children aged 2 to 5 years by the end of 2021, and for children aged 6 months to 2 years in the first quarter of 2022, which, if positive, will greatly facilitate the vaccination process for younger children. Besides, immune protection for this specific group of newborns could be considered starting with pregnant women. Recent studies (46–48) have shown that antibodies can be detected in the placenta or breast milk after vaccination of pregnant or lactating women without a significant increase in adverse fetal or neonatal outcomes, which may suggest an alternative route of immune protection for the fetus or newborn. Higher-level randomized controlled trials are needed to validate this idea in order to ensure maternal and infant safety.

Furthermore, considering the overall benefits to society, we have to assess whether the benefits of vaccinating children outweigh the burden on overall local epidemic control (49). In a situation where vaccines are in short supply, it seems more ethical to give priority to immunocompromised populations such as the elderly (50). Local tailoring may be the solution to this dilemma. However, it was the emergence of the Omicron variant that has reminded us the only a comprehensive vaccination program, including for low-risk populations, will allow us to achieve victory against the epidemic.

In addition, given the urgency of advancing childhood vaccination, there is a need for a comprehensive assessment of the factors influencing vaccination, particularly those affecting parental intentions. Surveys around the world (16, 17, 51) have shown that distrust in the safety and efficacy of vaccines is an important reason why parents are reluctant to have their children vaccinated, and that most parents are willing to vaccinate their children when the vaccine is safe and reliable. Therefore, high-quality studies assessing the safety and efficacy of the COVID-19 vaccine in younger children appear to be essential to eliminate childhood vaccine hesitancy. Moreover, parental fear of COVID-19 is an important influencing factor in the decision to vaccinate children (51, 52), stemming not only from the health risks children may face, but also from the risk of family transmission due to children's infection, which may have a negative impact on the family's economic income as well as social activities. Therefore, in order to enhance parents' perception of COVID-19, local governments should proactively provide a platform for scientific communication and share valid data in a timely manner. Furthermore, race, religious affiliation, trust in government agencies, willingness to get vaccinated for themselves, education level, annual income, work environment, mother tongue, and age may all be important factors influencing parent's willingness (18, 51). As the epidemic progressed, surveys from various countries spurted out, but most were single-center surveys. Surveys may be contradictory from country to country (18, 19), and parental attitudes may change as the epidemic evolves. Therefore, in addition to continuing to advance research on vaccines, a systematic review that brings together various influencing factors is highly desirable (41) and will help us assess the influencing factors that affect parental willingness in different contexts, thus guiding us to take various effective measures to advance the childhood vaccination process for various populations in different regions.

## Conclusions

In conclusion, our meta-analysis pooled the available randomized controlled trials and confirmed the favorable safety, immunogenicity, and efficacy of COVID-19 vaccines (mRNA-1273 vaccine, BNT162b2 Covid-19 Vaccine, CoronaVac, BBIBP-COV, and Ad5-vectored COVID-19 vaccine) in adolescents and children aged 3–17 years. Nevertheless, there is still a large gap in trials to confirm the safety and efficacy of different COVID-19 vaccines in people under 18 years of age, especially in younger children under 3 years old and even infants. There is an urgent need to conduct multicenter, large-sample clinical studies of COVID-19 vaccine in younger children with a wider range of vaccine types and longer follow-up periods, to promote global universalization and standardization of childhood vaccination. Given the rapidly changing epidemiological situation and the advancing vaccine research process, this meta-analysis should be updated in time when more data are available.

## Data Availability Statement

The original contributions presented in the study are included in the article/Supplementary Materials, further inquiries can be directed to the corresponding author.

## Author Contributions

YD and YS conceived and designed the study, performed the data analysis, and prepared the figures and the tables. YD and LC conducted the database search and extracted the data. YD wrote the manuscript. LC and YS revised it critically for important intellectual content. YS is the guarantor. All authors contributed to the article and approved the submitted version.

## Conflict of Interest

The authors declare that the research was conducted in the absence of any commercial or financial relationships that could be construed as a potential conflict of interest.

## Publisher's Note

All claims expressed in this article are solely those of the authors and do not necessarily represent those of their affiliated organizations, or those of the publisher, the editors and the reviewers. Any product that may be evaluated in this article, or claim that may be made by its manufacturer, is not guaranteed or endorsed by the publisher.
