# Supplementary material for: Safety, Immunogenicity, and Efficacy of COVID-19 Vaccines in Adolescents, Children, and Infants: A Systematic Review and Meta-Analysis
Source: Front Public Health. 2022 Apr 14;10:829176. doi: 10.3389/fpubh.2022.829176 (PMC9046659; doi:10.3389/fpubh.2022.829176)
Supplement: Supplementary file 1 [file Data_Sheet_1.ZIP › Supplementary Material/Search formula.docx]

**Search formula**

**1. pubmed**

**1.1 Search formula:**

**(randomized controlled trial[Publication Type] OR randomized[Title/Abstract] OR placebo[Title/Abstract]) AND ((((((COVID 19 Vaccines[Title/Abstract] OR Vaccines, COVID-19[Title/Abstract] OR COVID-19 Virus Vaccines[Title/Abstract] OR COVID 19 Virus Vaccines[Title/Abstract] OR Vaccines, COVID-19 Virus[Title/Abstract] OR Virus Vaccines, COVID-19[Title/Abstract] OR COVID-19 Virus Vaccine[Title/Abstract] OR COVID 19 Virus Vaccine[Title/Abstract] OR Vaccine, COVID-19 Virus[Title/Abstract] OR Virus Vaccine, COVID-19[Title/Abstract] OR COVID19 Virus Vaccines[Title/Abstract] OR Vaccines, COVID19 Virus[Title/Abstract] OR Virus Vaccines, COVID19[Title/Abstract] OR COVID19 Virus Vaccine[Title/Abstract] OR Vaccine, COVID19 Virus[Title/Abstract] OR Virus Vaccine, COVID19[Title/Abstract] OR COVID19 Vaccines[Title/Abstract] OR Vaccines, COVID19[Title/Abstract] OR COVID19 Vaccine[Title/Abstract] OR Vaccine, COVID19[Title/Abstract] OR SARS-CoV-2 Vaccines[Title/Abstract] OR SARS CoV 2 Vaccines[Title/Abstract] OR Vaccines, SARS-CoV-2[Title/Abstract] OR SARS-CoV-2 Vaccine[Title/Abstract] OR SARS CoV 2 Vaccine[Title/Abstract] OR Vaccine, SARS-CoV-2[Title/Abstract] OR SARS2 Vaccines[Title/Abstract] OR Vaccines, SARS2[Title/Abstract] OR SARS2 Vaccine[Title/Abstract] OR Vaccine, SARS2[Title/Abstract] OR Coronavirus Disease 2019 Vaccines[Title/Abstract] OR Coronavirus Disease 2019 Vaccine[Title/Abstract] OR Coronavirus Disease 2019 Virus Vaccine[Title/Abstract] OR Coronavirus Disease 2019 Virus Vaccines[Title/Abstract] OR Coronavirus Disease-19 Vaccines[Title/Abstract] OR Coronavirus Disease 19 Vaccines[Title/Abstract] OR Vaccines, Coronavirus Disease-19[Title/Abstract] OR Coronavirus Disease-19 Vaccine[Title/Abstract] OR Coronavirus Disease 19 Vaccine[Title/Abstract] OR Vaccine, Coronavirus Disease-19[Title/Abstract] OR COVID 19 Vaccine[Title/Abstract] OR Vaccine, COVID 19[Title/Abstract] OR 2019-nCoV Vaccine[Title/Abstract] OR 2019 nCoV Vaccine[Title/Abstract] OR Vaccine, 2019-nCoV[Title/Abstract] OR 2019 Novel Coronavirus Vaccines[Title/Abstract] OR 2019 Novel Coronavirus Vaccine[Title/Abstract] OR 2019-nCoV Vaccines[Title/Abstract] OR 2019 nCoV Vaccines[Title/Abstract] OR Vaccines, 2019-nCoV[Title/Abstract] OR COVID-19 Vaccine[Title/Abstract] OR Vaccine, COVID-19[Title/Abstract] OR SARS Coronavirus 2 Vaccines[Title/Abstract])) OR ((COVID 19[Title/Abstract] OR COVID-19 Virus Disease[Title/Abstract] OR COVID 19 Virus Disease[Title/Abstract] OR COVID-19 Virus Diseases[Title/Abstract] OR Disease, COVID-19 Virus[Title/Abstract] OR Virus Disease, COVID-19[Title/Abstract] OR COVID-19 Virus Infection[Title/Abstract] OR COVID 19 Virus Infection[Title/Abstract] OR COVID-19 Virus Infections[Title/Abstract] OR Infection, COVID-19 Virus[Title/Abstract] OR Virus Infection, COVID-19[Title/Abstract] OR 2019-nCoV Infection[Title/Abstract] OR 2019 nCoV Infection[Title/Abstract] OR 2019-nCoV Infections[Title/Abstract] OR Infection, 2019-nCoV[Title/Abstract] OR Coronavirus Disease-19[Title/Abstract] OR Coronavirus Disease 19[Title/Abstract] OR 2019 Novel Coronavirus Disease[Title/Abstract] OR 2019 Novel Coronavirus Infection[Title/Abstract] OR 2019-nCoV Disease[Title/Abstract] OR 2019 nCoV Disease[Title/Abstract] OR 2019-nCoV Diseases[Title/Abstract] OR Disease, 2019-nCoV[Title/Abstract] OR COVID19[Title/Abstract] OR Coronavirus Disease 2019[Title/Abstract] OR Disease 2019, Coronavirus[Title/Abstract] OR SARS Coronavirus 2 Infection[Title/Abstract] OR SARS-CoV-2 Infection[Title/Abstract] OR Infection, SARS-CoV-2[Title/Abstract] OR SARS CoV 2 Infection[Title/Abstract] OR SARS-CoV-2 Infections[Title/Abstract] OR COVID-19 Pandemic[Title/Abstract] OR COVID 19 Pandemic[Title/Abstract] OR COVID-19 Pandemics[Title/Abstract] OR Pandemic, COVID-19[Title/Abstract]))) OR ((Coronavirus Disease 2019 Virus[Title/Abstract] OR 2019 Novel Coronavirus[Title/Abstract] OR 2019 Novel Coronaviruses[Title/Abstract] OR Coronavirus, 2019 Novel[Title/Abstract] OR Novel Coronavirus, 2019[Title/Abstract] OR Wuhan Seafood Market Pneumonia Virus[Title/Abstract] OR SARS-CoV-2 Virus[Title/Abstract] OR SARS CoV 2 Virus[Title/Abstract] OR SARS-CoV-2 Viruses[Title/Abstract] OR Virus, SARS-CoV-2[Title/Abstract] OR 2019-nCoV[Title/Abstract] OR COVID-19 Virus[Title/Abstract] OR COVID 19 Virus[Title/Abstract] OR COVID-19 Viruses[Title/Abstract] OR Virus, COVID-19[Title/Abstract] OR Wuhan Coronavirus[Title/Abstract] OR Coronavirus, Wuhan[Title/Abstract] OR SARS Coronavirus 2[Title/Abstract] OR Coronavirus 2, SARS[Title/Abstract] OR Severe Acute Respiratory Syndrome Coronavirus 2[Title/Abstract]))) OR ((("SARS-CoV-2"[Mesh]) OR "COVID-19"[Mesh]) OR "COVID-19 Vaccines"[Mesh])) AND (((((("Infant"[Mesh]) OR "Child"[Mesh]) OR "Adolescent"[Mesh]) OR ((Adolescents[Title/Abstract] OR Adolescence[Title/Abstract] OR Teens[Title/Abstract] OR Teen[Title/Abstract] OR Teenagers[Title/Abstract] OR Teenager[Title/Abstract] OR Youth[Title/Abstract] OR Youths[Title/Abstract] OR Adolescents, Female[Title/Abstract] OR Adolescent, Female[Title/Abstract] OR Female Adolescent[Title/Abstract] OR Female Adolescents[Title/Abstract] OR Adolescents, Male[Title/Abstract] OR Adolescent, Male[Title/Abstract] OR Male Adolescent[Title/Abstract] OR Male Adolescents[Title/Abstract]))) OR (Children[Title/Abstract])) OR (Infants[Title/Abstract])))** Sort by: **Most Recent**

**1.2 The part of the database that we used:** Advanced search.

**1.3 The searching period:** From inception to November 9, 2021.

**2. EMBASE**

**2.1 Search formula:**

.......................................................

No. Query Results Results Date

#14. #5 AND #12 AND #13 270 9 Nov 2021

#13. 'randomized controlled trial':ab,ti OR 1,005,789 9 Nov 2021

'randomized':ab,ti OR 'placebo':ab,ti

#12. #6 OR #7 OR #8 OR #9 OR #10 OR #11 203,562 9 Nov 2021

#11. 'covid-19':ab,ti OR 'covid 19':ab,ti OR 174,001 9 Nov 2021

'covid-19 virus disease':ab,ti OR 'covid 19 virus

disease':ab,ti OR 'covid-19 virus diseases':ab,ti

OR 'disease, covid-19 virus':ab,ti OR 'virus

disease, covid-19':ab,ti OR 'covid-19 virus

infection':ab,ti OR 'covid 19 virus

infection':ab,ti OR 'covid-19 virus

infections':ab,ti OR

'infection, covid-19 virus':ab,ti OR 'virus

infection, covid-19':ab,ti OR

'2019-ncov infection':ab,ti OR '2019

ncov infection':ab,ti OR

'2019-ncov infections':ab,ti OR

'infection, 2019-ncov':ab,ti OR 'coronavirus

disease-19':ab,ti OR 'coronavirus disease

19':ab,ti OR '2019 novel coronavirus

disease':ab,ti OR '2019 novel coronavirus

infection':ab,ti OR '2019-ncov disease':ab,ti OR

'2019 ncov disease':ab,ti OR

'2019-ncov diseases':ab,ti OR

'disease, 2019-ncov':ab,ti OR 'covid19':ab,ti OR

'coronavirus disease 2019':ab,ti OR 'disease

2019, coronavirus':ab,ti OR 'sars coronavirus 2

infection':ab,ti OR 'sars-cov-2 infection':ab,ti

OR 'infection, sars-cov-2':ab,ti OR 'sars cov

2 infection':ab,ti OR

'sars-cov-2 infections':ab,ti OR

'covid-19 pandemic':ab,ti OR 'covid

19 pandemic':ab,ti OR 'covid-19 pandemics':ab,ti

OR 'pandemic, covid-19':ab,ti

#10. 'coronavirus disease 2019'/exp 160,317 9 Nov 2021

#9. 'sars-cov-2':ab,ti OR 'coronavirus disease 2019 59,746 9 Nov 2021

virus':ab,ti OR '2019 novel coronavirus':ab,ti OR

'2019 novel coronaviruses':ab,ti OR 'coronavirus,

2019 novel':ab,ti OR 'novel coronavirus,

2019':ab,ti OR 'wuhan seafood market pneumonia

virus':ab,ti OR 'sars-cov-2 virus':ab,ti OR 'sars

cov 2 virus':ab,ti OR 'sars-cov-2 viruses':ab,ti

OR 'virus, sars-cov-2':ab,ti OR '2019-ncov':ab,ti

OR 'covid-19 virus':ab,ti OR 'covid

19 virus':ab,ti OR 'covid-19 viruses':ab,ti OR

'virus, covid-19':ab,ti OR 'wuhan

coronavirus':ab,ti OR 'coronavirus, wuhan':ab,ti

OR 'sars coronavirus 2':ab,ti OR 'coronavirus 2,

sars':ab,ti OR 'severe acute respiratory syndrome

coronavirus 2':ab,ti

#8. 'severe acute respiratory syndrome coronavirus 47,538 9 Nov 2021

2'/exp

#7. 'covid-19 vaccines':ab,ti OR 'covid 19 5,279 9 Nov 2021

vaccines':ab,ti OR 'vaccines, covid-19':ab,ti OR

'covid-19 virus vaccines':ab,ti OR 'covid 19

virus vaccines':ab,ti OR 'vaccines, covid-19

virus':ab,ti OR 'virus vaccines, covid-19':ab,ti

OR 'covid-19 virus vaccine':ab,ti OR 'covid 19

virus vaccine':ab,ti OR 'vaccine, covid-19

virus':ab,ti OR 'virus vaccine, covid-19':ab,ti

OR 'covid19 virus vaccines':ab,ti OR 'vaccines,

covid19 virus':ab,ti OR 'virus vaccines,

covid19':ab,ti OR 'covid19 virus vaccine':ab,ti

OR 'vaccine, covid19 virus':ab,ti OR 'virus

vaccine, covid19':ab,ti OR 'covid19

vaccines':ab,ti OR 'vaccines, covid19':ab,ti OR

'covid19 vaccine':ab,ti OR 'vaccine,

covid19':ab,ti OR 'sars-cov-2 vaccines':ab,ti OR

'sars cov 2 vaccines':ab,ti OR 'vaccines,

sars-cov-2':ab,ti OR 'sars-cov-2 vaccine':ab,ti

OR 'sars cov 2 vaccine':ab,ti OR 'vaccine,

sars-cov-2':ab,ti OR 'sars2 vaccines':ab,ti OR

'vaccines, sars2':ab,ti OR 'sars2 vaccine':ab,ti

OR 'vaccine, sars2':ab,ti OR 'coronavirus disease

2019 vaccines':ab,ti OR 'coronavirus disease 2019

vaccine':ab,ti OR 'coronavirus disease 2019 virus

vaccine':ab,ti OR 'coronavirus disease 2019 virus

vaccines':ab,ti OR 'coronavirus disease-19

vaccines':ab,ti OR 'coronavirus disease 19

vaccines':ab,ti OR 'vaccines, coronavirus

disease-19':ab,ti OR 'coronavirus disease-19

vaccine':ab,ti OR 'coronavirus disease 19

vaccine':ab,ti OR 'vaccine, coronavirus

disease-19':ab,ti OR 'covid 19 vaccine':ab,ti OR

'vaccine, covid 19':ab,ti OR '2019-ncov

vaccine':ab,ti OR '2019 ncov vaccine':ab,ti OR

'vaccine, 2019-ncov':ab,ti OR '2019 novel

coronavirus vaccines':ab,ti OR '2019 novel

coronavirus vaccine':ab,ti OR '2019-ncov

vaccines':ab,ti OR '2019 ncov vaccines':ab,ti OR

'vaccines, 2019-ncov':ab,ti OR 'covid-19

vaccine':ab,ti OR 'vaccine, covid-19':ab,ti OR

'sars coronavirus 2 vaccines':ab,ti

#6. 'sars-cov-2 vaccine'/exp 8,475 9 Nov 2021

#5. #1 OR #2 OR #3 OR #4 4,362,680 9 Nov 2021

#4. 'infants':ab,ti OR 'children':ab,ti OR 2,008,581 9 Nov 2021

'adolescents':ab,ti OR 'adolescence':ab,ti OR

'teens':ab,ti OR 'teen':ab,ti OR

'teenagers':ab,ti OR 'teenager':ab,ti OR

'youth':ab,ti OR 'youths':ab,ti OR 'adolescents,

female':ab,ti OR 'adolescent, female':ab,ti OR

'female adolescent':ab,ti OR

'female adolescents':ab,ti OR 'adolescents,

male':ab,ti OR 'adolescent, male':ab,ti OR

'male adolescent':ab,ti OR

'male adolescents':ab,ti

#3. 'infant'/exp 1,202,045 9 Nov 2021

#2. 'child'/exp 3,096,179 9 Nov 2021

#1. 'adolescent'/exp 1,778,075 9 Nov 2021

.......................................................

**2.2 The part of the database that we used:** Emtree and Advanced search.

**2.3 The searching period:** From inception to November 9, 2021.

**3. The cochrane library**

**3.1 Search formula:**

#1. MeSH descriptor: [Child] explode all trees

#2. MeSH descriptor: [Infant] explode all trees

#3. MeSH descriptor: [Adolescent] explode all trees

#4. (Infants):ti,ab,kw OR (Children):ti,ab,kw OR (Adolescents):ti,ab,kw OR (Adolescence):ti,ab,kw OR (Teens):ti,ab,kw OR (Teen):ti,ab,kw OR (Teenagers):ti,ab,kw OR (Teenager):ti,ab,kw OR (Youth):ti,ab,kw OR (Youths):ti,ab,kw OR (Adolescents, Female):ti,ab,kw OR (Adolescent, Female):ti,ab,kw OR (Female Adolescent):ti,ab,kw OR (Female Adolescents):ti,ab,kw OR (Adolescents, Male):ti,ab,kw OR (Adolescent, Male):ti,ab,kw OR (Male Adolescent):ti,ab,kw OR (Male Adolescents):ti,ab,kw

#5. #1 OR #2 OR #3 OR #4

#6. (COVID 19 Vaccines):ti,ab,kw OR (COVID 19 Virus Vaccines):ti,ab,kw OR (COVID 19 Virus Vaccine):ti,ab,kw OR (COVID19 Virus Vaccines):ti,ab,kw OR (Vaccines, COVID19 Virus):ti,ab,kw OR (Virus Vaccines, COVID19):ti,ab,kw OR (COVID19 Virus Vaccine):ti,ab,kw OR (Vaccine, COVID19 Virus):ti,ab,kw OR (Virus Vaccine, COVID19):ti,ab,kw OR (COVID19 Vaccines):ti,ab,kw OR (Vaccines, COVID19):ti,ab,kw OR (COVID19 Vaccine):ti,ab,kw OR (Vaccine, COVID19):ti,ab,kw OR (SARS CoV 2 Vaccines):ti,ab,kw OR (SARS CoV 2 Vaccine):ti,ab,kw OR (SARS2 Vaccines):ti,ab,kw OR (Vaccines, SARS2):ti,ab,kw OR (SARS2 Vaccine):ti,ab,kw OR (Vaccine, SARS2):ti,ab,kw OR (Coronavirus Disease 2019 Vaccines):ti,ab,kw OR (Coronavirus Disease 2019 Vaccine):ti,ab,kw OR (Coronavirus Disease 2019 Virus Vaccine):ti,ab,kw OR (Coronavirus Disease 2019 Virus Vaccines):ti,ab,kw OR (Coronavirus Disease 19 Vaccines):ti,ab,kw OR (Coronavirus Disease 19 Vaccine):ti,ab,kw OR (COVID 19 Vaccine):ti,ab,kw OR (Vaccine, COVID 19):ti,ab,kw OR (2019 nCoV Vaccine):ti,ab,kw OR (2019 Novel Coronavirus Vaccines):ti,ab,kw OR (2019 Novel Coronavirus Vaccine):ti,ab,kw OR (2019 nCoV Vaccines):ti,ab,kw OR (SARS Coronavirus 2 Vaccines):ti,ab,kw

#7. MeSH descriptor: [COVID-19 Vaccines] explode all trees

#8. MeSH descriptor: [SARS-CoV-2] explode all trees

#9. (Coronavirus Disease 2019 Virus):ti,ab,kw OR (2019 Novel Coronavirus):ti,ab,kw OR (2019 Novel Coronaviruses):ti,ab,kw OR (Coronavirus, 2019 Novel):ti,ab,kw OR (Novel Coronavirus, 2019):ti,ab,kw OR (Wuhan Seafood Market Pneumonia Virus):ti,ab,kw OR (SARS CoV 2 Virus):ti,ab,kw OR (COVID 19 Virus):ti,ab,kw OR (Wuhan Coronavirus):ti,ab,kw OR (Coronavirus, Wuhan):ti,ab,kw OR (SARS Coronavirus 2):ti,ab,kw OR (Coronavirus 2, SARS):ti,ab,kw OR (Severe Acute Respiratory Syndrome Coronavirus 2):ti,ab,kw

#10. MeSH descriptor: [COVID-19] explode all trees

#11. (COVID 19):ti,ab,kw OR (COVID 19 Virus Disease):ti,ab,kw OR (COVID 19 Virus Infection):ti,ab,kw OR (2019 nCoV Infection):ti,ab,kw OR (Coronavirus Disease 19):ti,ab,kw OR (2019 Novel Coronavirus Disease):ti,ab,kw OR (2019 Novel Coronavirus Infection):ti,ab,kw OR (2019 nCoV Disease):ti,ab,kw OR (COVID19):ti,ab,kw OR (Coronavirus Disease 2019):ti,ab,kw OR (Disease 2019, Coronavirus):ti,ab,kw OR (SARS Coronavirus 2 Infection):ti,ab,kw OR (SARS CoV 2 Infection):ti,ab,kw OR (COVID 19 Pandemic):ti,ab,kw

#12. #6 OR #7 OR #8 OR #9 OR #10 OR #11

#13. #5 OR #12

**3.2 The part of the database that we used:** Advanced search.

**3.3 The searching period:** From inception to November 9, 2021.
