# Supplementary material for: Safety, Immunogenicity, and Efficacy of COVID-19 Vaccines in Adolescents, Children, and Infants: A Systematic Review and Meta-Analysis
Source: Front Public Health. 2022 Apr 14;10:829176. doi: 10.3389/fpubh.2022.829176 (PMC9046659; doi:10.3389/fpubh.2022.829176)

Supplementary Figure 1. Unsolicited adverse reactions within 28 or 30 days after whole vaccination procedure

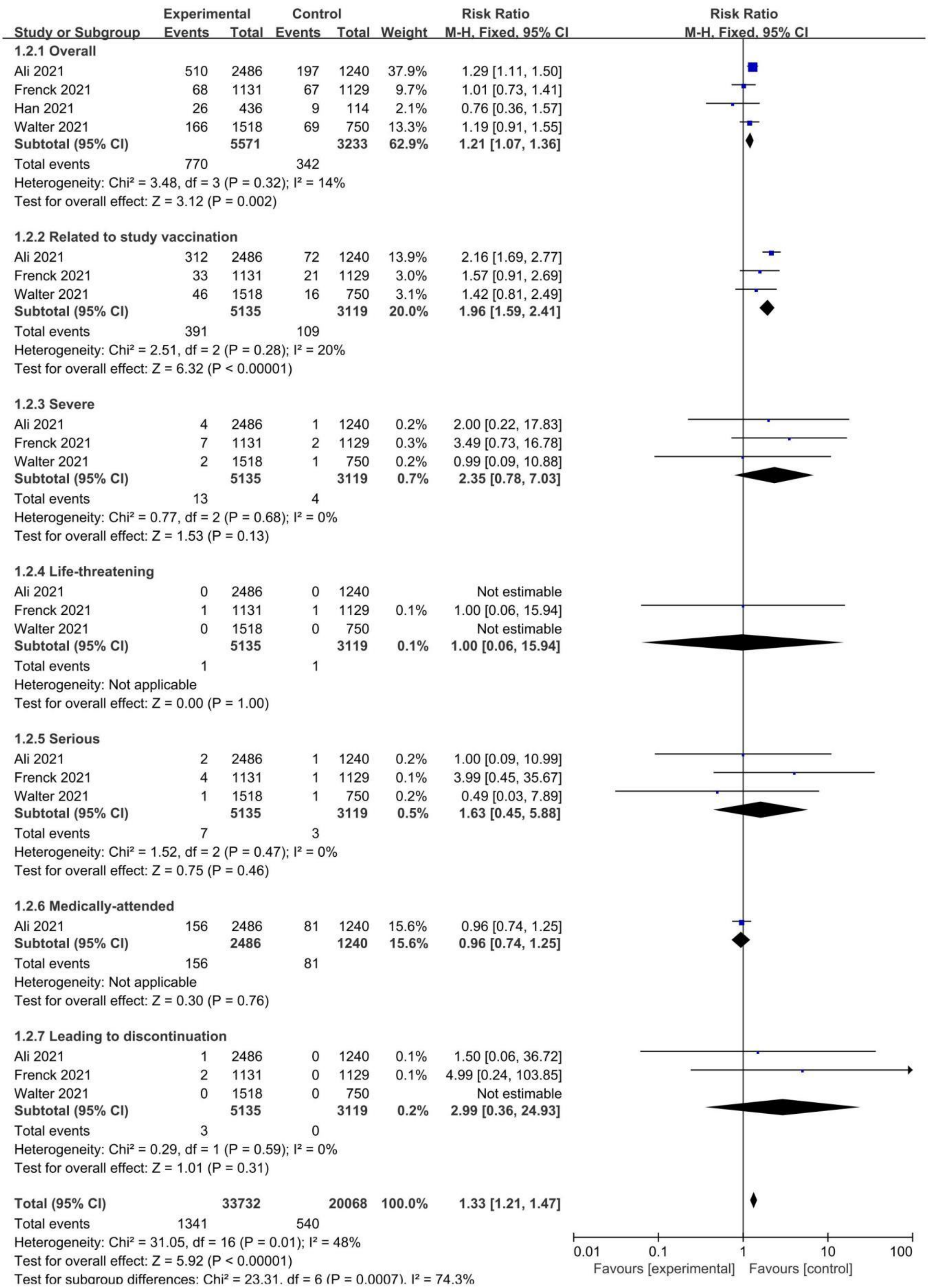

Supplementary Figure 2. Total adverse reactions in vaccination group versus control group

Study or Subgroup	Experimental Events	Experimental Total	Control Events	Control Total	Weight	Risk Ratio M-H, Fixed, 95% CI
**1.3.1 Solicited adverse reactions after the first vaccination**						
Ali 2021	2381	2482	806	1238	21.8%	1.47 [1.41, 1.54]
Han 2021	73	436	15	114	0.5%	1.27 [0.76, 2.13]
Zhu 2021	55	100	8	50	0.2%	3.44 [1.78, 6.65]
**Subtotal (95% CI)**		**3018**		**1402**	**22.5%**	**1.49 [1.43, 1.55]**
Total events	2509		829			
Heterogeneity: Chi² = 6.76, df = 2 (P = 0.03); I² = 70%						
Test for overall effect: Z = 18.34 (P < 0.00001)						
**1.3.2 Solicited local adverse reactions after the first vaccination**						
Ali 2021	2339	2482	455	1238	12.3%	2.56 [2.38, 2.76]
Xia 2021	38	755	2	252	0.1%	6.34 [1.54, 26.10]
Zhu 2021	36	100	3	50	0.1%	6.00 [1.94, 18.53]
**Subtotal (95% CI)**		**3337**		**1540**	**12.5%**	**2.60 [2.42, 2.80]**
Total events	2413		460			
Heterogeneity: Chi² = 3.80, df = 2 (P = 0.15); I² = 47%						
Test for overall effect: Z = 25.38 (P < 0.00001)						
**1.3.3 Solicited systemic adverse reactions after the first vaccination**						
Ali 2021	1701	2482	687	1238	18.6%	1.23 [1.17, 1.31]
Xia 2021	99	755	25	252	0.8%	1.32 [0.87, 2.00]
Zhu 2021	37	100	5	50	0.1%	3.70 [1.55, 8.83]
**Subtotal (95% CI)**		**3337**		**1540**	**19.5%**	**1.26 [1.19, 1.33]**
Total events	1837		717			
Heterogeneity: Chi² = 6.31, df = 2 (P = 0.04); I² = 68%						
Test for overall effect: Z = 7.82 (P < 0.00001)						
**1.3.4 Solicited adverse reactions after the second vaccination**						
Ali 2021	2405	2478	680	1220	18.5%	1.74 [1.66, 1.83]
Han 2021	56	425	8	111	0.3%	1.83 [0.90, 3.72]
Zhu 2021	33	100	2	50	0.1%	8.25 [2.06, 33.00]
**Subtotal (95% CI)**		**3003**		**1381**	**18.8%**	**1.76 [1.67, 1.85]**
Total events	2494		690			
Heterogeneity: Chi² = 4.97, df = 2 (P = 0.08); I² = 60%						
Test for overall effect: Z = 21.72 (P < 0.00001)						
**1.3.5 Solicited local adverse reactions after the second vaccination**						
Ali 2021	2314	2478	398	1220	10.8%	2.86 [2.64, 3.10]
Xia 2021	26	751	2	248	0.1%	4.29 [1.03, 17.96]
Zhu 2021	19	100	0	50	0.0%	19.69 [1.21, 319.62]
**Subtotal (95% CI)**		**3329**		**1518**	**10.9%**	**2.89 [2.67, 3.14]**
Total events	2359		400			
Heterogeneity: Chi² = 2.17, df = 2 (P = 0.34); I² = 8%						
Test for overall effect: Z = 25.55 (P < 0.00001)						
**1.3.6 Solicited systemic adverse reactions after the second vaccination**						
Ali 2021	2134	2478	561	1220	15.3%	1.87 [1.76, 1.99]
Xia 2021	39	751	8	248	0.2%	1.61 [0.76, 3.40]
Zhu 2021	24	100	2	50	0.1%	6.00 [1.48, 24.38]
**Subtotal (95% CI)**		**3329**		**1518**	**15.6%**	**1.88 [1.77, 2.01]**
Total events	2197		571			
Heterogeneity: Chi² = 2.82, df = 2 (P = 0.24); I² = 29%						
Test for overall effect: Z = 19.68 (P < 0.00001)						
**1.3.8 Solicited local adverse reactions after the third vaccination**						
Xia 2021	17	749	3	246	0.1%	1.86 [0.55, 6.30]
**Subtotal (95% CI)**		**749**		**246**	**0.1%**	**1.86 [0.55, 6.30]**
Total events	17		3			
Heterogeneity: Not applicable						
Test for overall effect: Z = 1.00 (P = 0.32)						
**1.3.9 Solicited systemic adverse reactions after the third vaccination**						
Xia 2021	21	749	3	246	0.1%	2.30 [0.69, 7.64]
**Subtotal (95% CI)**		**749**		**246**	**0.1%**	**2.30 [0.69, 7.64]**
Total events	21		3			
Heterogeneity: Not applicable						
Test for overall effect: Z = 1.36 (P = 0.17)						
**Total (95% CI)**		**20851**		**9391**	**100.0%**	**1.85 [1.80, 1.89]**
Total events	13847		3673			
Heterogeneity: Chi² = 531.19, df = 19 (P < 0.00001); I² = 96%						
Test for overall effect: Z = 49.61 (P < 0.00001)						
Test for subgroup differences: Chi² = 447.95, df = 7 (P < 0.00001), I² = 98.4%						

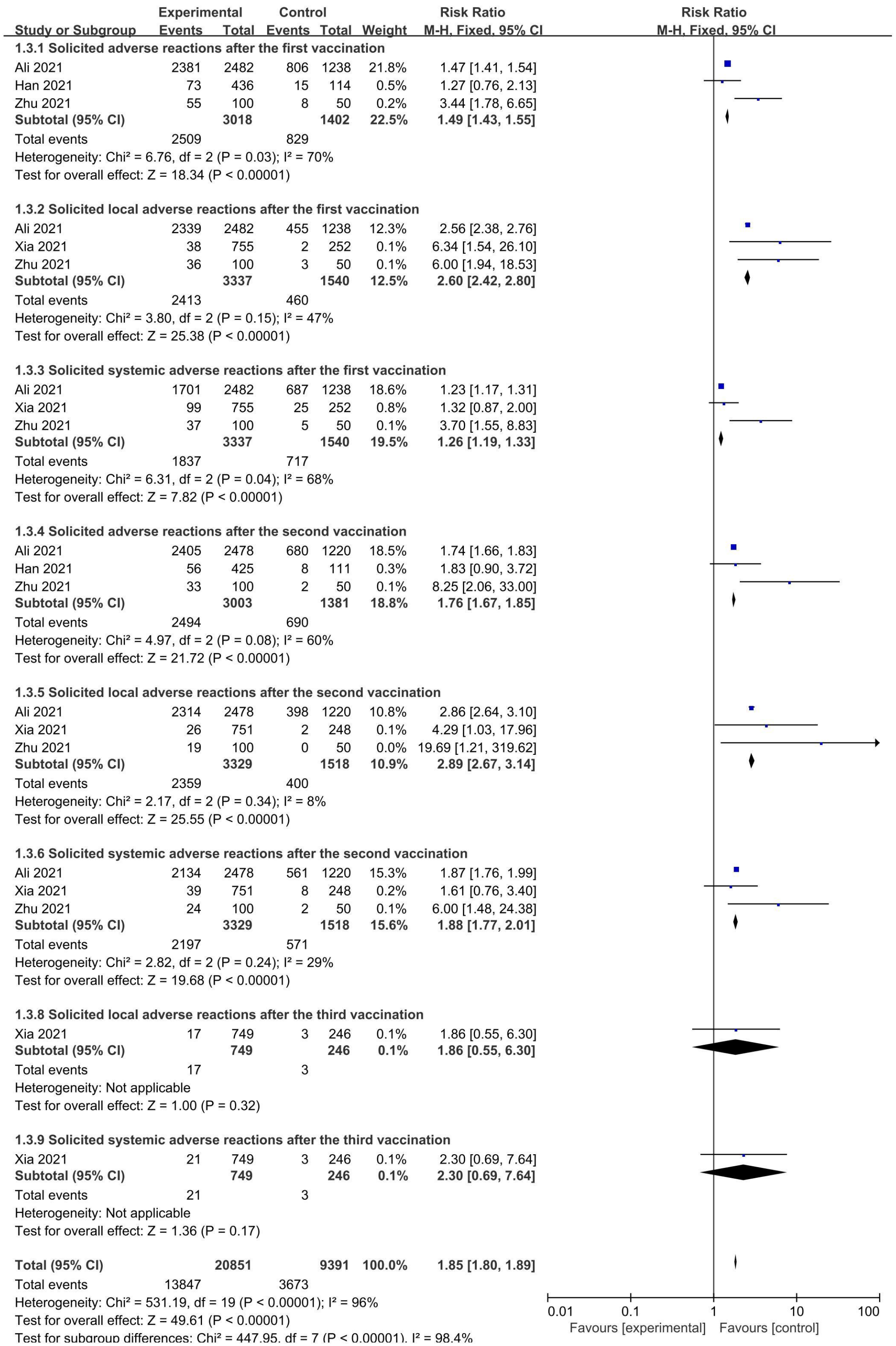

Supplementary Figure 3. Total adverse reactions in vaccination group after dose 1 versus after dose 2

	Dose 1		Dose 2			Risk Ratio
Study or Subgroup	Events	Total	Events	Total	Weight	M-H, Fixed, 95% CI
**2.1.1 Total adverse reactions**						
Ali 2021	2381	2482	2405	2478	34.1%	0.99 [0.98, 1.00]
Han 2021	73	436	56	425	0.8%	1.27 [0.92, 1.75]
Zhu 2021	55	100	33	100	0.5%	1.67 [1.20, 2.32]
**Subtotal (95% CI)**		**3018**		**3003**	**35.4%**	**1.00 [0.99, 1.02]**
Total events	2509		2494			
Heterogeneity: Chi² = 19.21, df = 2 (P < 0.0001); I² = 90%						
Test for overall effect: Z = 0.52 (P = 0.60)						
**2.1.2 Local adverse reactions**						
Ali 2021	2339	2482	2314	2478	32.8%	1.01 [0.99, 1.02]
Xia 2021	38	755	26	751	0.4%	1.45 [0.89, 2.37]
Zhu 2021	36	100	19	100	0.3%	1.89 [1.17, 3.07]
**Subtotal (95% CI)**		**3337**		**3329**	**33.5%**	**1.02 [1.00, 1.04]**
Total events	2413		2359			
Heterogeneity: Chi² = 10.97, df = 2 (P = 0.004); I² = 82%						
Test for overall effect: Z = 2.53 (P = 0.01)						
**2.1.3 Systemic adverse reactions**						
Ali 2021	1701	2482	2134	2478	30.3%	0.80 [0.77, 0.82]
Xia 2021	99	755	39	751	0.6%	2.53 [1.77, 3.61]
Zhu 2021	37	100	24	100	0.3%	1.54 [1.00, 2.38]
**Subtotal (95% CI)**		**3337**		**3329**	**31.2%**	**0.83 [0.81, 0.86]**
Total events	1837		2197			
Heterogeneity: Chi² = 53.87, df = 2 (P < 0.00001); I² = 96%						
Test for overall effect: Z = 11.05 (P < 0.00001)						
**Total (95% CI)**		**9692**		**9661**	**100.0%**	**0.96 [0.95, 0.97]**
Total events	6759		7050			
Heterogeneity: Chi² = 282.22, df = 8 (P < 0.00001); I² = 97%						
Test for overall effect: Z = 7.16 (P < 0.00001)						
Test for subgroup differences: Chi² = 126.45, df = 2 (P < 0.00001), I² = 98.4%						

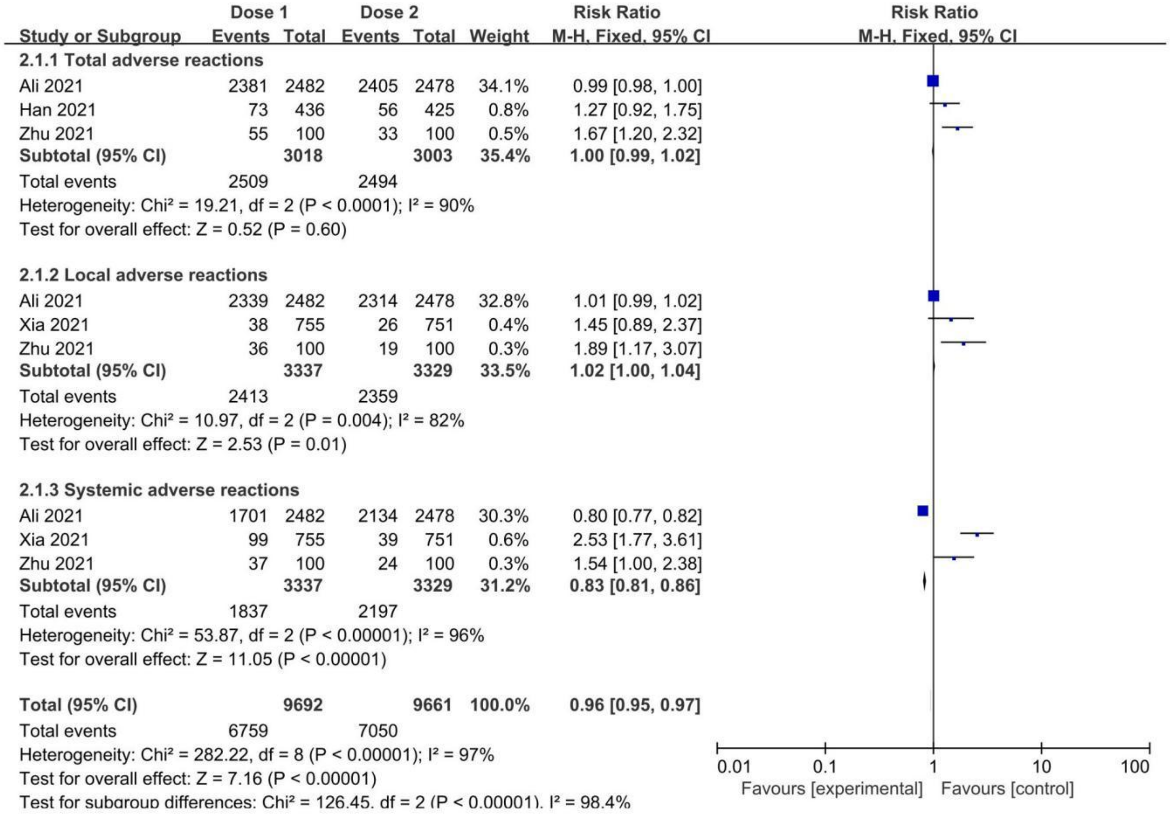

Supplementary Figure 4. Adverse reactions among vaccination group versus control group: A) Total adverse reactions after dose 1; B) Total adverse reactions after dose 2; C)Local adverse reactions after dose 1; D) Local adverse reactions after dose 2; E) Systemic adverse reactions after dose 1; F) Systemic adverse reactions after dose 2

A) Total adverse reactions after dose 1

	Experimental		Control			Risk Ratio
Study or Subgroup	Events	Total	Events	Total	Weight	M-H, Fixed, 95% CI
**3.1.1 mRNA vaccine**						
Ali 2021	2381	2482	806	1238	96.9%	1.47 [1.41, 1.54]
**Subtotal (95% CI)**		**2482**		**1238**	**96.9%**	**1.47 [1.41, 1.54]**
Total events	2381		806			
Heterogeneity: Not applicable						
Test for overall effect: Z = 18.27 (P < 0.00001)						
**3.1.2 Inactivated vaccine**						
Han 2021	73	436	15	114	2.1%	1.27 [0.76, 2.13]
**Subtotal (95% CI)**		**436**		**114**	**2.1%**	**1.27 [0.76, 2.13]**
Total events	73		15			
Heterogeneity: Not applicable						
Test for overall effect: Z = 0.92 (P = 0.36)						
**3.1.3 Virus-vectored vaccine**						
Zhu 2021	55	100	8	50	1.0%	3.44 [1.78, 6.65]
**Subtotal (95% CI)**		**100**		**50**	**1.0%**	**3.44 [1.78, 6.65]**
Total events	55		8			
Heterogeneity: Not applicable						
Test for overall effect: Z = 3.67 (P = 0.0002)						
**Total (95% CI)**		**3018**		**1402**	**100.0%**	**1.49 [1.43, 1.55]**
Total events	2509		829			
Heterogeneity: Chi² = 6.76, df = 2 (P = 0.03); I² = 70%						
Test for overall effect: Z = 18.34 (P < 0.00001)						
Test for subgroup differences: Chi² = 6.64, df = 2 (P = 0.04), I² = 69.9%						

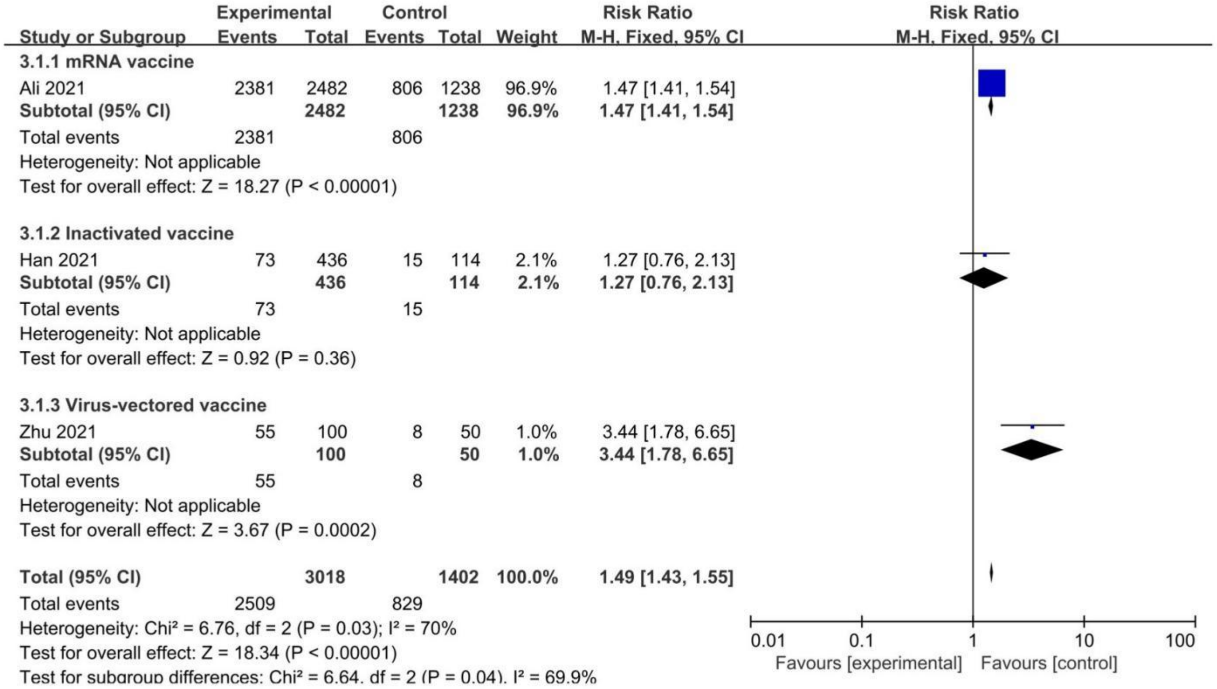

B) Total adverse reactions after dose 2

	Experimental		Control			Risk Ratio
Study or Subgroup	Events	Total	Events	Total	Weight	M-H, Fixed, 95% CI
**3.2.1 mRNA vaccine**						
Ali 2021	2405	2478	680	1220	98.3%	1.74 [1.66, 1.83]
**Subtotal (95% CI)**		**2478**		**1220**	**98.3%**	**1.74 [1.66, 1.83]**
Total events	2405		680			
Heterogeneity: Not applicable						
Test for overall effect: Z = 21.54 (P < 0.00001)						
**3.2.2 Inactivated vaccine**						
Han 2021	56	425	8	111	1.4%	1.83 [0.90, 3.72]
**Subtotal (95% CI)**		**425**		**111**	**1.4%**	**1.83 [0.90, 3.72]**
Total events	56		8			
Heterogeneity: Not applicable						
Test for overall effect: Z = 1.66 (P = 0.10)						
**3.2.3 Virus-vectored vaccine**						
Zhu 2021	33	100	2	50	0.3%	8.25 [2.06, 33.00]
**Subtotal (95% CI)**		**100**		**50**	**0.3%**	**8.25 [2.06, 33.00]**
Total events	33		2			
Heterogeneity: Not applicable						
Test for overall effect: Z = 2.98 (P = 0.003)						
**Total (95% CI)**		**3003**		**1381**	**100.0%**	**1.76 [1.67, 1.85]**
Total events	2494		690			
Heterogeneity: Chi² = 4.97, df = 2 (P = 0.08); I² = 60%						
Test for overall effect: Z = 21.72 (P < 0.00001)						
Test for subgroup differences: Chi² = 4.85, df = 2 (P = 0.09), I² = 58.7%						

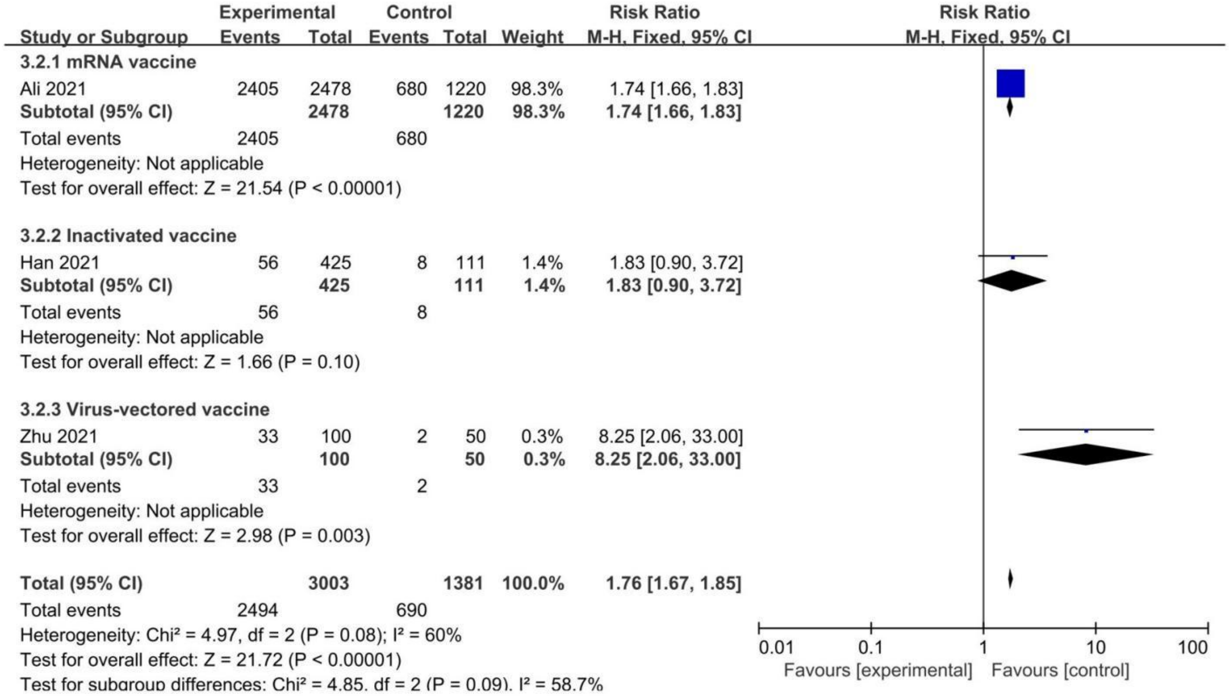

C)Local adverse reactions after dose 1

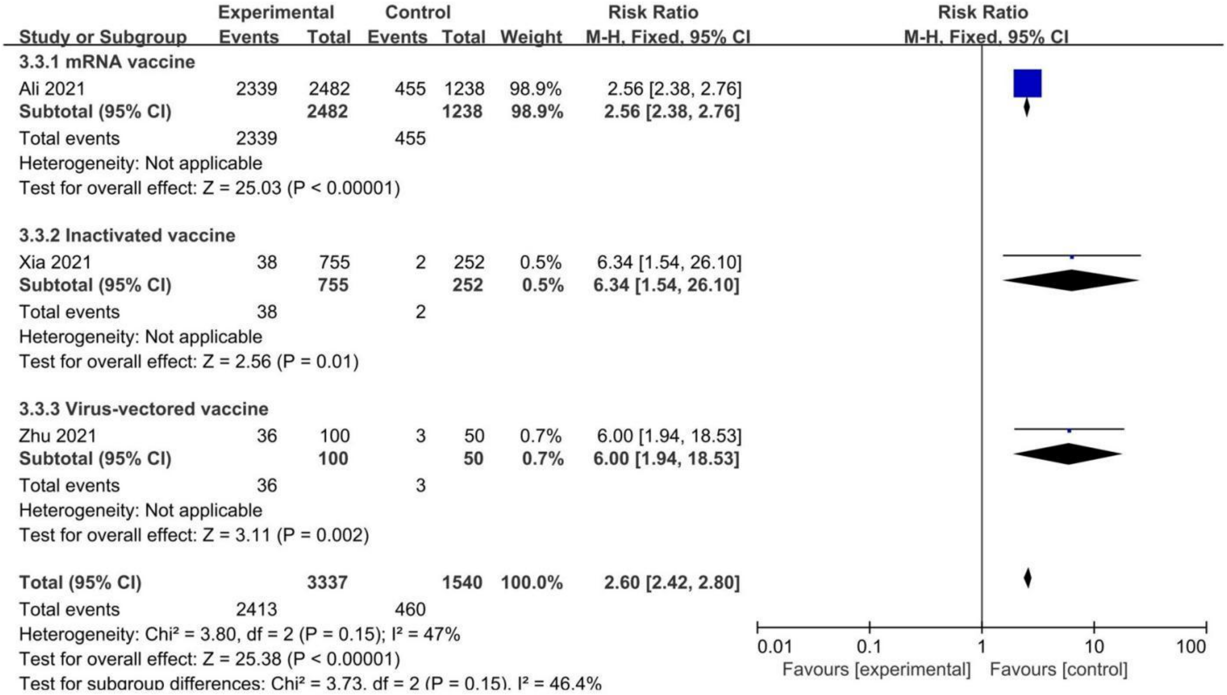

D) Local adverse reactions after dose 2

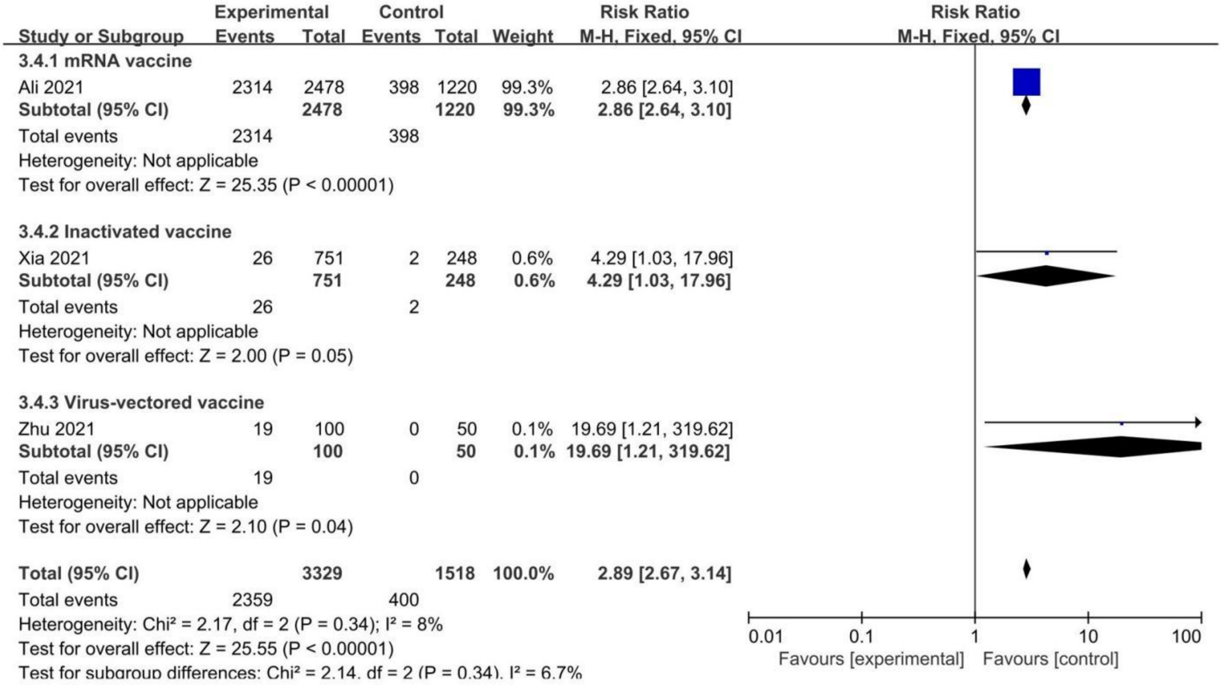

E) Systemic adverse reactions after dose 1

Study or Subgroup	Experimental Events	Experimental Total	Control Events	Control Total	Weight	Risk Ratio M-H, Fixed, 95% CI
**3.5.1 mRNA vaccine**						
Ali 2021	1701	2482	687	1238	95.4%	1.23 [1.17, 1.31]
**Subtotal (95% CI)**		**2482**		**1238**	**95.4%**	**1.23 [1.17, 1.31]**
Total events	1701		687			
Heterogeneity: Not applicable						
Test for overall effect: Z = 7.31 (P < 0.00001)						
**3.5.2 Inactivated vaccine**						
Xia 2021	99	755	25	252	3.9%	1.32 [0.87, 2.00]
**Subtotal (95% CI)**		**755**		**252**	**3.9%**	**1.32 [0.87, 2.00]**
Total events	99		25			
Heterogeneity: Not applicable						
Test for overall effect: Z = 1.32 (P = 0.19)						
**3.5.3 Virus-vectored vaccine**						
Zhu 2021	37	100	5	50	0.7%	3.70 [1.55, 8.83]
**Subtotal (95% CI)**		**100**		**50**	**0.7%**	**3.70 [1.55, 8.83]**
Total events	37		5			
Heterogeneity: Not applicable						
Test for overall effect: Z = 2.95 (P = 0.003)						
**Total (95% CI)**		**3337**		**1540**	**100.0%**	**1.26 [1.19, 1.33]**
Total events	1837		717			
Heterogeneity: Chi² = 6.31, df = 2 (P = 0.04); I² = 68%						
Test for overall effect: Z = 7.82 (P < 0.00001)						
Test for subgroup differences: Chi² = 6.17, df = 2 (P = 0.05), I² = 67.6%						

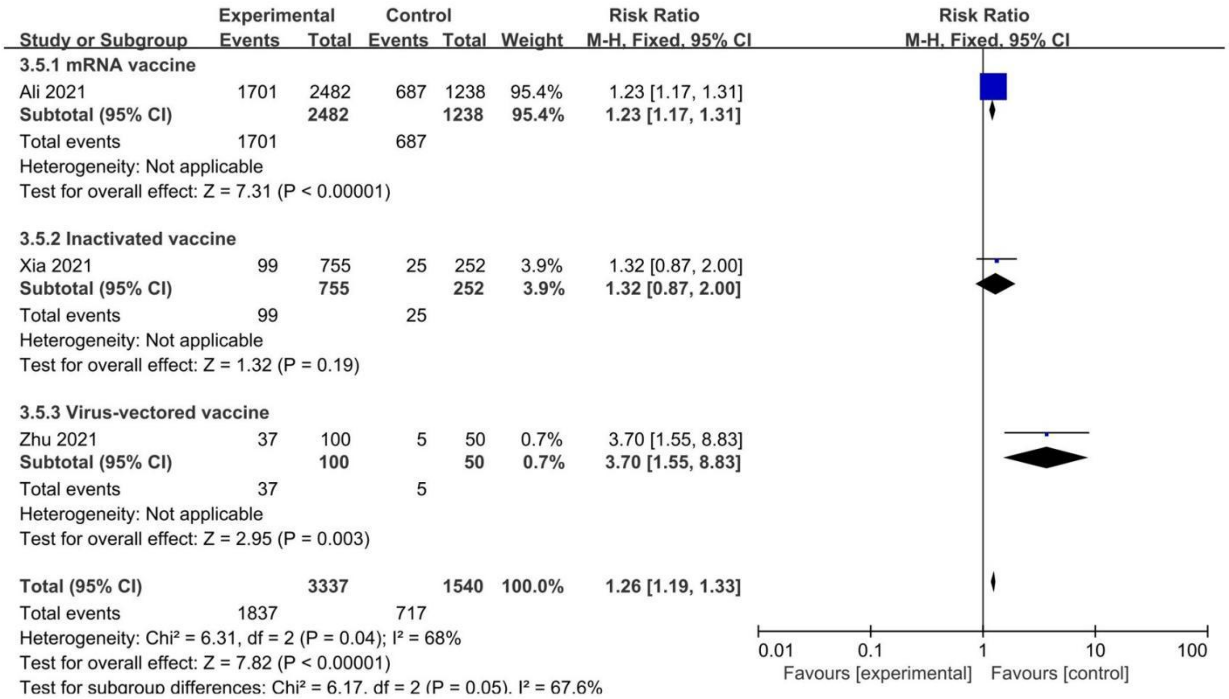

F) Systemic adverse reactions after dose 2

Study or Subgroup	Experimental Events	Experimental Total	Control Events	Control Total	Weight	Risk Ratio M-H, Fixed, 95% CI
**3.6.1 mRNA vaccine**						
Ali 2021	2134	2478	561	1220	98.1%	1.87 [1.76, 1.99]
**Subtotal (95% CI)**		**2478**		**1220**	**98.1%**	**1.87 [1.76, 1.99]**
Total events	2134		561			
Heterogeneity: Not applicable						
Test for overall effect: Z = 19.57 (P < 0.00001)						
**3.6.2 Inactivated vaccine**						
Xia 2021	39	751	8	248	1.6%	1.61 [0.76, 3.40]
**Subtotal (95% CI)**		**751**		**248**	**1.6%**	**1.61 [0.76, 3.40]**
Total events	39		8			
Heterogeneity: Not applicable						
Test for overall effect: Z = 1.25 (P = 0.21)						
**3.6.3 Virus-vectored vaccine**						
Zhu 2021	24	100	2	50	0.3%	6.00 [1.48, 24.38]
**Subtotal (95% CI)**		**100**		**50**	**0.3%**	**6.00 [1.48, 24.38]**
Total events	24		2			
Heterogeneity: Not applicable						
Test for overall effect: Z = 2.50 (P = 0.01)						
**Total (95% CI)**		**3329**		**1518**	**100.0%**	**1.88 [1.77, 2.01]**
Total events	2197		571			
Heterogeneity: Chi² = 2.82, df = 2 (P = 0.24); I² = 29%						
Test for overall effect: Z = 19.68 (P < 0.00001)						
Test for subgroup differences: Chi² = 2.81, df = 2 (P = 0.25), I² = 28.7%						

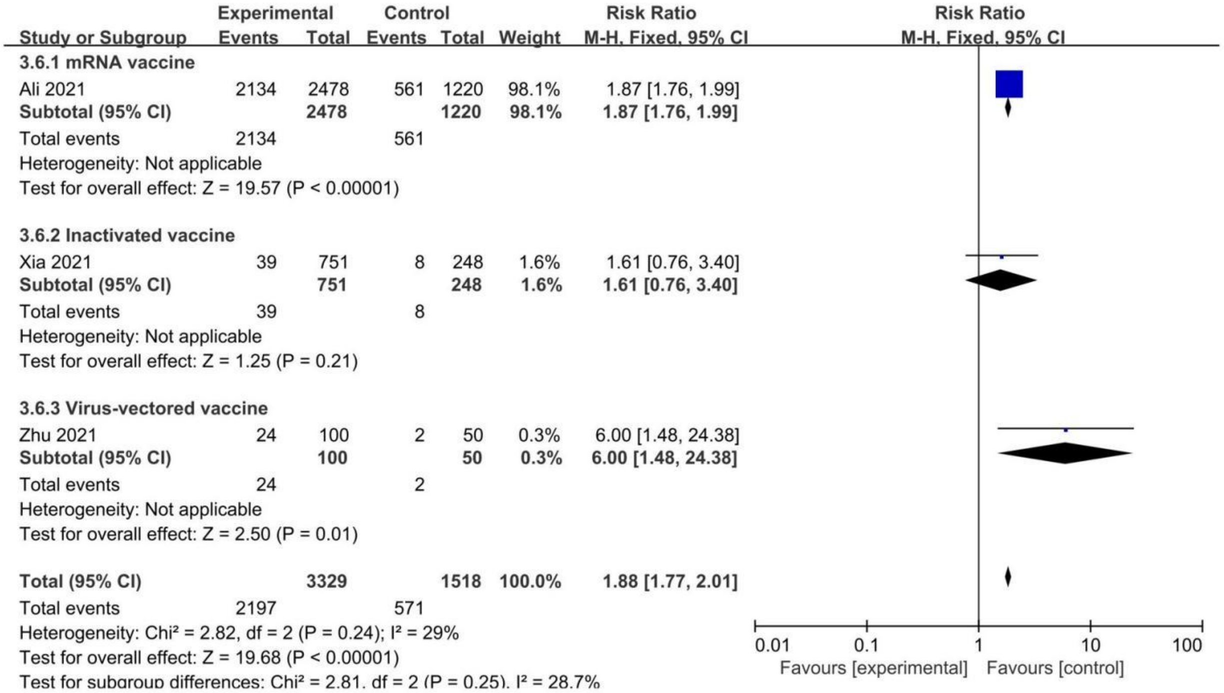

Supplementary Figure 5. Specific adverse reactions in mRNA vaccine group versus control group:

A) After dose 1

	Experimental		Control			Risk Ratio
Study or Subgroup	Events	Total	Events	Total	Weight	M-H, Fixed, 95% CI
**3.7.1 Local pain (local adverse reaction)**						
Ali 2021	2310	2482	431	1238	18.6%	2.67 [2.48, 2.89]
Frenck 2021	86	1131	23	1129	0.7%	3.73 [2.37, 5.87]
Walter 2021	74	1511	31	749	1.3%	1.18 [0.79, 1.78]
**Subtotal (95% CI)**		**5124**		**3116**	**20.7%**	**2.61 [2.43, 2.82]**
Total events	2470		485			
Heterogeneity: Chi^2 = 17.05, df = 2 (P = 0.0002); I^2 = 88%						
Test for overall effect: Z = 25.18 (P < 0.00001)						
**3.7.2 Erythema or Redness (local adverse reaction)**						
Ali 2021	334	2482	8	1238	0.3%	20.82 [10.36, 41.85]
Frenck 2021	6	1131	1	1129	0.0%	5.99 [0.72, 49.67]
Walter 2021	15	1511	6	749	0.3%	1.24 [0.48, 3.18]
**Subtotal (95% CI)**		**5124**		**3116**	**0.6%**	**12.09 [7.21, 20.28]**
Total events	355		15			
Heterogeneity: Chi^2 = 25.19, df = 2 (P < 0.00001); I^2 = 92%						
Test for overall effect: Z = 9.45 (P < 0.00001)						
**3.7.3 Swelling (local adverse reaction)**						
Ali 2021	403	2482	12	1238	0.5%	16.75 [9.47, 29.62]
Frenck 2021	7	1131	1	1129	0.0%	6.99 [0.86, 56.70]
Walter 2021	10	1511	3	749	0.1%	1.65 [0.46, 5.99]
**Subtotal (95% CI)**		**5124**		**3116**	**0.7%**	**13.41 [8.15, 22.06]**
Total events	420		16			
Heterogeneity: Chi^2 = 11.12, df = 2 (P = 0.004); I^2 = 82%						
Test for overall effect: Z = 10.21 (P < 0.00001)						
**3.7.4 Axillary Swelling (local adverse reaction)**						
Ali 2021	578	2482	101	1238	4.4%	2.85 [2.34, 3.49]
**Subtotal (95% CI)**		**2482**		**1238**	**4.4%**	**2.85 [2.34, 3.49]**
Total events	578		101			
Heterogeneity: Not applicable						
Test for overall effect: Z = 10.28 (P < 0.00001)						
**3.7.5 Fever (systemic adverse reaction)**						
Ali 2021	63	2480	12	1238	0.5%	2.62 [1.42, 4.84]
Frenck 2021	10	1131	1	1129	0.0%	9.98 [1.28, 77.85]
Walter 2021	3	1511	1	749	0.0%	1.49 [0.15, 14.27]
**Subtotal (95% CI)**		**5122**		**3116**	**0.6%**	**2.94 [1.68, 5.14]**
Total events	76		14			
Heterogeneity: Chi^2 = 1.84, df = 2 (P = 0.40); I^2 = 0%						
Test for overall effect: Z = 3.78 (P = 0.0002)						
**3.7.6 Headache (systemic adverse reaction)**						
Ali 2021	1106	2480	477	1238	20.6%	1.16 [1.07, 1.26]
Frenck 2021	55	1131	35	1129	1.1%	1.57 [1.04, 2.38]
Walter 2021	22	1511	24	749	1.0%	0.45 [0.26, 0.80]
**Subtotal (95% CI)**		**5122**		**3116**	**22.8%**	**1.15 [1.06, 1.24]**
Total events	1183		536			
Heterogeneity: Chi^2 = 12.30, df = 2 (P = 0.002); I^2 = 84%						
Test for overall effect: Z = 3.30 (P = 0.0010)						
**3.7.7 Fatigue (systemic adverse reaction)**						
Ali 2021	1188	2481	453	1238	19.6%	1.31 [1.20, 1.42]
Frenck 2021	60	1131	41	1129	1.3%	1.46 [0.99, 2.15]
Walter 2021	34	1511	31	749	1.3%	0.54 [0.34, 0.88]
**Subtotal (95% CI)**		**5123**		**3116**	**22.3%**	**1.27 [1.17, 1.38]**
Total events	1282		525			
Heterogeneity: Chi^2 = 13.04, df = 2 (P = 0.001); I^2 = 85%						
Test for overall effect: Z = 5.79 (P < 0.00001)						
**3.7.8 Myalgia (systemic adverse reaction)肌痛**						
Ali 2021	668	2480	205	1238	8.9%	1.63 [1.41, 1.87]
Frenck 2021	24	1131	13	1129	0.4%	1.84 [0.94, 3.60]
Walter 2021	9	1511	7	749	0.3%	0.64 [0.24, 1.70]
**Subtotal (95% CI)**		**5122**		**3116**	**9.6%**	**1.60 [1.40, 1.84]**
Total events	701		225			
Heterogeneity: Chi^2 = 3.58, df = 2 (P = 0.17); I^2 = 44%						
Test for overall effect: Z = 6.80 (P < 0.00001)						
**3.7.9 Arthralgia (systemic adverse reaction)**						
Ali 2021	371	2480	143	1238	6.2%	1.30 [1.08, 1.55]
Frenck 2021	10	1131	7	1129	0.2%	1.43 [0.54, 3.73]
Walter 2021	3	1511	5	749	0.2%	0.30 [0.07, 1.24]
**Subtotal (95% CI)**		**5122**		**3116**	**6.6%**	**1.27 [1.06, 1.51]**
Total events	384		155			
Heterogeneity: Chi^2 = 4.07, df = 2 (P = 0.13); I^2 = 51%						
Test for overall effect: Z = 2.65 (P = 0.008)						
**3.7.10 Nausea/vomiting (systemic adverse reaction)**						
Ali 2021	281	2480	110	1238	4.8%	1.28 [1.03, 1.57]
**Subtotal (95% CI)**		**2480**		**1238**	**4.8%**	**1.28 [1.03, 1.57]**
Total events	281		110			
Heterogeneity: Not applicable						
Test for overall effect: Z = 2.27 (P = 0.02)						
**3.7.11 Vomiting (systemic adverse reaction)**						
Frenck 2021	3	1131	1	1129	0.0%	2.99 [0.31, 28.75]
Walter 2021	2	1511	1	749	0.0%	0.99 [0.09, 10.92]
**Subtotal (95% CI)**		**2642**		**1878**	**0.1%**	**1.85 [0.38, 9.07]**
Total events	5		2			
Heterogeneity: Chi^2 = 0.43, df = 1 (P = 0.51); I^2 = 0%						
Test for overall effect: Z = 0.76 (P = 0.45)						
**3.7.12 Diarrhea (systemic adverse reaction)**						
Frenck 2021	8	1131	7	1129	0.2%	1.14 [0.42, 3.14]
Walter 2021	6	1511	4	749	0.2%	0.74 [0.21, 2.63]
**Subtotal (95% CI)**		**2642**		**1878**	**0.4%**	**0.97 [0.44, 2.12]**
Total events	14		11			
Heterogeneity: Chi^2 = 0.27, df = 1 (P = 0.60); I^2 = 0%						
Test for overall effect: Z = 0.08 (P = 0.94)						
**3.7.13 Chills (systemic adverse reaction)寒冷**						
Ali 2021	456	2480	138	1238	6.0%	1.65 [1.38, 1.97]
Frenck 2021	28	1131	10	1129	0.3%	2.80 [1.36, 5.73]
Walter 2021	5	1511	5	749	0.2%	0.50 [0.14, 1.71]
**Subtotal (95% CI)**		**5122**		**3116**	**6.5%**	**1.67 [1.41, 1.98]**
Total events	489		153			
Heterogeneity: Chi^2 = 5.70, df = 2 (P = 0.06); I^2 = 65%						
Test for overall effect: Z = 5.90 (P < 0.00001)						
**Total (95% CI)**		**56351**		**34276**	**100.0%**	**1.81 [1.74, 1.88]**
Total events	8238		2348			
Heterogeneity: Chi^2 = 509.23, df = 32 (P < 0.00001); I^2 = 94%						
Test for overall effect: Z = 29.75 (P < 0.00001)						
Test for subgroup differences: Chi^2 = 437.52, df = 12 (P < 0.00001), I^2 = 97.3%						

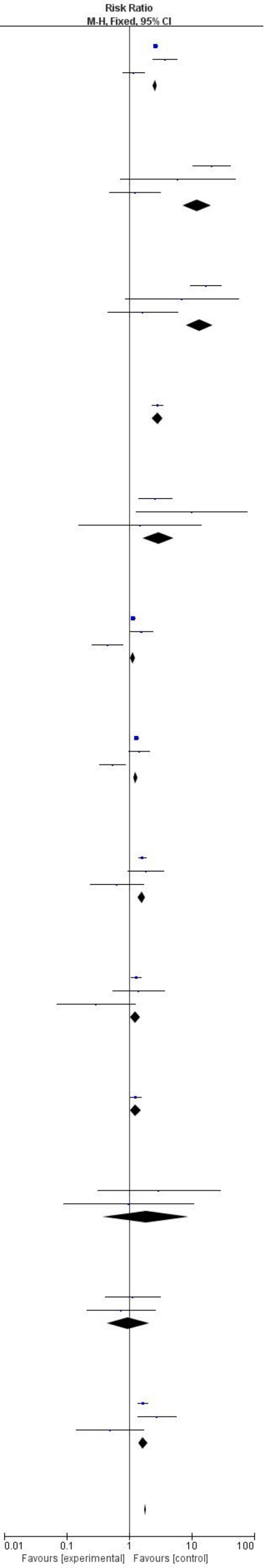

B) After dose 2

Study or Subgroup	Experimental Events	Experimental Total	Control Events	Control Total	Weight	Risk Ratio M-H, Fixed, 95% CI
**3.8.1 Local pain (local adverse reaction)**						
Ali 2021	2290	2478	370	1220	20.2%	3.05 [2.80, 3.32]
Frenck 2021	79	1131	18	1129	0.7%	4.38 [2.64, 7.26]
Walter 2021	71	1501	29	741	1.6%	1.21 [0.79, 1.84]
**Subtotal (95% CI)**		**5110**		**3090**	**22.5%**	**2.96 [2.73, 3.22]**
Total events	2440		417			
Heterogeneity: Chi² = 19.99, df = 2 (P < 0.0001); I² = 90%						
Test for overall effect: Z = 25.70 (P < 0.00001)						
**3.8.2 Erythema or Redness (local adverse reaction)**						
Ali 2021	484	2478	11	1220	0.6%	21.66 [11.96, 39.22]
Frenck 2021	5	1131	1	1129	0.0%	4.99 [0.58, 42.65]
Walter 2021	19	1501	5	741	0.3%	1.88 [0.70, 5.00]
**Subtotal (95% CI)**		**5110**		**3090**	**0.9%**	**15.02 [9.26, 24.34]**
Total events	508		17			
Heterogeneity: Chi² = 19.74, df = 2 (P < 0.0001); I² = 90%						
Test for overall effect: Z = 10.99 (P < 0.00001)						
**3.8.3 Swelling (local adverse reaction)**						
Ali 2021	509	2478	12	1220	0.7%	20.88 [11.83, 36.86]
Frenck 2021	5	1131	1	1129	0.0%	4.99 [0.58, 42.65]
Walter 2021	15	1501	3	741	0.2%	2.47 [0.72, 8.50]
**Subtotal (95% CI)**		**5110**		**3090**	**0.9%**	**16.62 [10.11, 27.33]**
Total events	529		16			
Heterogeneity: Chi² = 10.97, df = 2 (P = 0.004); I² = 82%						
Test for overall effect: Z = 11.08 (P < 0.00001)						
**3.8.4 Axillary Swelling (local adverse reaction)**						
Ali 2021	519	2477	61	1220	3.3%	4.19 [3.24, 5.41]
**Subtotal (95% CI)**		**2477**		**1220**	**3.3%**	**4.19 [3.24, 5.41]**
Total events	519		61			
Heterogeneity: Not applicable						
Test for overall effect: Z = 10.96 (P < 0.00001)						
**3.8.5 Fever (systemic adverse reaction)**						
Ali 2021	302	2477	12	1220	0.7%	12.40 [6.99, 21.98]
Frenck 2021	20	1131	1	1129	0.0%	19.96 [2.68, 148.51]
Walter 2021	7	1501	1	741	0.1%	3.46 [0.43, 28.04]
**Subtotal (95% CI)**		**5109**		**3090**	**0.8%**	**12.16 [7.15, 20.66]**
Total events	329		14			
Heterogeneity: Chi² = 1.63, df = 2 (P = 0.44); I² = 0%						
Test for overall effect: Z = 9.23 (P < 0.00001)						
**3.8.6 Headache (systemic adverse reaction)**						
Ali 2021	1739	2478	370	1220	20.2%	2.31 [2.12, 2.53]
Frenck 2021	65	1131	24	1129	1.0%	2.70 [1.71, 4.29]
Walter 2021	28	1501	19	741	1.0%	0.73 [0.41, 1.29]
**Subtotal (95% CI)**		**5110**		**3090**	**22.2%**	**2.26 [2.07, 2.46]**
Total events	1832		413			
Heterogeneity: Chi² = 15.74, df = 2 (P = 0.0004); I² = 87%						
Test for overall effect: Z = 18.53 (P < 0.00001)						
**3.8.7 Fatigue (systemic adverse reaction)**						
Ali 2021	1679	2478	353	1220	19.3%	2.34 [2.14, 2.57]
Frenck 2021	66	1131	25	1129	1.0%	2.64 [1.68, 4.14]
Walter 2021	39	1501	24	741	1.3%	0.80 [0.49, 1.32]
**Subtotal (95% CI)**		**5110**		**3090**	**21.6%**	**2.26 [2.07, 2.47]**
Total events	1784		402			
Heterogeneity: Chi² = 17.44, df = 2 (P = 0.0002); I² = 89%						
Test for overall effect: Z = 18.07 (P < 0.00001)						
**3.8.8 Myalgia (systemic adverse reaction)**						
Ali 2021	1154	2477	153	1220	8.3%	3.71 [3.18, 4.33]
Frenck 2021	32	1131	8	1129	0.3%	3.99 [1.85, 8.63]
Walter 2021	12	1501	7	741	0.4%	0.85 [0.33, 2.14]
**Subtotal (95% CI)**		**5109**		**3090**	**9.1%**	**3.60 [3.11, 4.18]**
Total events	1198		168			
Heterogeneity: Chi² = 9.58, df = 2 (P = 0.008); I² = 79%						
Test for overall effect: Z = 16.94 (P < 0.00001)						
**3.8.9 Arthralgia (systemic adverse reaction)**						
Ali 2021	716	2477	113	1220	6.2%	3.12 [2.59, 3.76]
Frenck 2021	16	1131	5	1129	0.2%	3.19 [1.17, 8.69]
Walter 2021	5	1501	4	741	0.2%	0.62 [0.17, 2.29]
**Subtotal (95% CI)**		**5109**		**3090**	**6.6%**	**3.04 [2.54, 3.64]**
Total events	737		122			
Heterogeneity: Chi² = 5.76, df = 2 (P = 0.06); I² = 65%						
Test for overall effect: Z = 12.08 (P < 0.00001)						
**3.8.10 Nausea/vomiting (systemic adverse reaction)**						
Ali 2021	591	2477	106	1220	5.8%	2.75 [2.26, 3.34]
**Subtotal (95% CI)**		**2477**		**1220**	**5.8%**	**2.75 [2.26, 3.34]**
Total events	591		106			
Heterogeneity: Not applicable						
Test for overall effect: Z = 10.15 (P < 0.00001)						
**3.8.11 Vomiting (systemic adverse reaction)**						
Frenck 2021	3	1131	1	1129	0.0%	2.99 [0.31, 28.75]
Walter 2021	2	1501	1	741	0.1%	0.99 [0.09, 10.87]
**Subtotal (95% CI)**		**2632**		**1870**	**0.1%**	**1.85 [0.38, 9.05]**
Total events	5		2			
Heterogeneity: Chi² = 0.44, df = 1 (P = 0.51); I² = 0%						
Test for overall effect: Z = 0.76 (P = 0.45)						
**3.8.12 Diarrhea (systemic adverse reaction)**						
Frenck 2021	6	1131	4	1129	0.2%	1.50 [0.42, 5.29]
Walter 2021	5	1501	5	156	0.4%	0.10 [0.03, 0.36]
**Subtotal (95% CI)**		**2632**		**1285**	**0.5%**	**0.53 [0.25, 1.13]**
Total events	11		9			
Heterogeneity: Chi² = 9.36, df = 1 (P = 0.002); I² = 89%						
Test for overall effect: Z = 1.65 (P = 0.10)						
**3.8.13 Chills (systemic adverse reaction)寒冷**						
Ali 2021	1066	2477	97	1220	5.3%	5.41 [4.45, 6.59]
Frenck 2021	42	1131	7	1129	0.3%	5.99 [2.70, 13.27]
Walter 2021	10	1501	4	741	0.2%	1.23 [0.39, 3.92]
**Subtotal (95% CI)**		**5109**		**3090**	**5.8%**	**5.28 [4.38, 6.37]**
Total events	1118		108			
Heterogeneity: Chi² = 6.23, df = 2 (P = 0.04); I² = 68%						
Test for overall effect: Z = 17.42 (P < 0.00001)						
**Total (95% CI)**		**56204**		**33405**	**100.0%**	**3.16 [3.03, 3.30]**
Total events	11601		1855			
Heterogeneity: Chi² = 365.73, df = 32 (P < 0.00001); I² = 91%						
Test for overall effect: Z = 52.54 (P < 0.00001)						
Test for subgroup differences: Chi² = 257.82, df = 12 (P < 0.00001), I² = 95.3%						

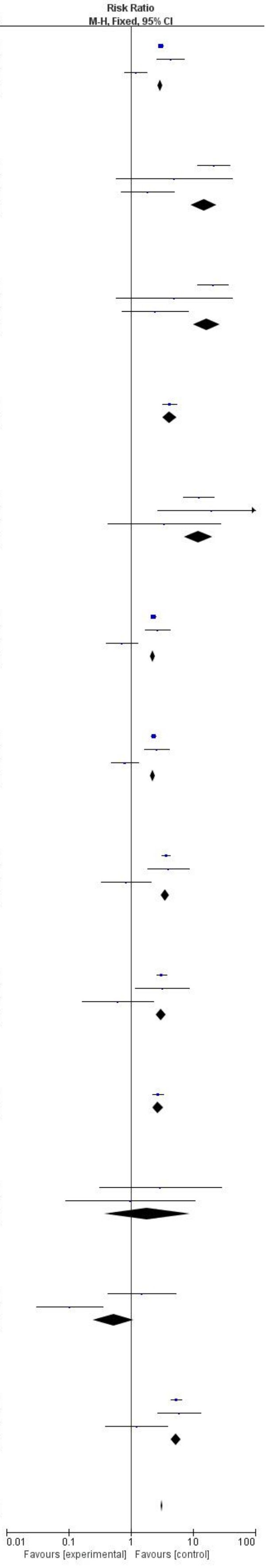

Supplementary Figure 6. Specific adverse reactions in inactivated vaccine group versus control group:

A) After dose 1

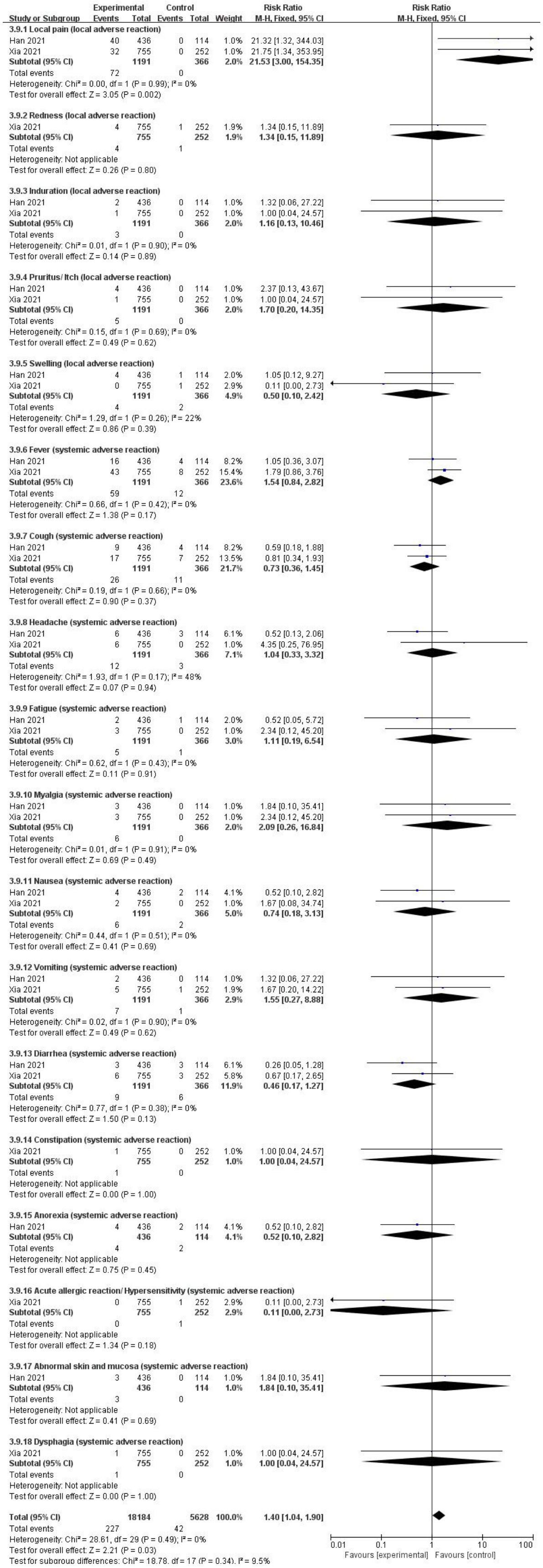

B) After dose 2

Study or Subgroup	Experimental Events	Experimental Total	Control Events	Control Total	Weight	Risk Ratio M-H, Fixed, 95% CI
**3.10.1 Local pain (local adverse reaction)**						
Han 2021	45	425	2	111	8.4%	5.88 [1.45, 23.85]
Xia 2021	16	751	0	248	2.0%	10.93 [0.66, 181.47]
**Subtotal (95% CI)**		**1176**		**359**	**10.4%**	**6.84 [1.96, 23.90]**
Total events	61		2			
Heterogeneity: Chi² = 0.15, df = 1 (P = 0.70); I² = 0%						
Test for overall effect: Z = 3.01 (P = 0.003)						
**3.10.2 Erythema or Redness (local adverse reaction)**						
Han 2021	1	425	0	111	2.1%	0.79 [0.03, 19.23]
Xia 2021	8	751	2	248	7.9%	1.32 [0.28, 6.18]
**Subtotal (95% CI)**		**1176**		**359**	**10.0%**	**1.21 [0.30, 4.83]**
Total events	9		2			
Heterogeneity: Chi² = 0.08, df = 1 (P = 0.78); I² = 0%						
Test for overall effect: Z = 0.27 (P = 0.79)						
**3.10.3 Pruritus/ Itch (local adverse reaction)**						
Han 2021	2	425	0	111	2.1%	1.31 [0.06, 27.19]
Xia 2021	1	751	0	248	2.0%	0.99 [0.04, 24.31]
**Subtotal (95% CI)**		**1176**		**359**	**4.1%**	**1.16 [0.13, 10.41]**
Total events	3		0			
Heterogeneity: Chi² = 0.02, df = 1 (P = 0.90); I² = 0%						
Test for overall effect: Z = 0.13 (P = 0.90)						
**3.10.4 Swelling (local adverse reaction)**						
Han 2021	6	425	0	111	2.1%	3.42 [0.19, 60.21]
Xia 2021	1	751	0	248	2.0%	0.99 [0.04, 24.31]
**Subtotal (95% CI)**		**1176**		**359**	**4.1%**	**2.24 [0.28, 18.17]**
Total events	7		0			
Heterogeneity: Chi² = 0.33, df = 1 (P = 0.56); I² = 0%						
Test for overall effect: Z = 0.75 (P = 0.45)						
**3.10.5 Fever (systemic adverse reaction)**						
Han 2021	5	425	1	111	4.2%	1.31 [0.15, 11.06]
Xia 2021	21	751	4	248	15.9%	1.73 [0.60, 5.00]
**Subtotal (95% CI)**		**1176**		**359**	**20.1%**	**1.64 [0.64, 4.24]**
Total events	26		5			
Heterogeneity: Chi² = 0.05, df = 1 (P = 0.82); I² = 0%						
Test for overall effect: Z = 1.03 (P = 0.30)						
**3.10.6 Cough (systemic adverse reaction)**						
Han 2021	5	425	1	111	4.2%	1.31 [0.15, 11.06]
Xia 2021	9	751	1	248	4.0%	2.97 [0.38, 23.34]
**Subtotal (95% CI)**		**1176**		**359**	**8.2%**	**2.12 [0.49, 9.19]**
Total events	14		2			
Heterogeneity: Chi² = 0.30, df = 1 (P = 0.58); I² = 0%						
Test for overall effect: Z = 1.00 (P = 0.32)						
**3.10.7 Headache (systemic adverse reaction)**						
Han 2021	5	425	0	111	2.1%	2.89 [0.16, 51.91]
Xia 2021	1	751	1	248	4.0%	0.33 [0.02, 5.26]
**Subtotal (95% CI)**		**1176**		**359**	**6.1%**	**1.21 [0.20, 7.38]**
Total events	6		1			
Heterogeneity: Chi² = 1.20, df = 1 (P = 0.27); I² = 16%						
Test for overall effect: Z = 0.21 (P = 0.83)						
**3.10.8 Fatigue (systemic adverse reaction)**						
Han 2021	1	425	0	111	2.1%	0.79 [0.03, 19.23]
Xia 2021	0	751	0	248		Not estimable
**Subtotal (95% CI)**		**1176**		**359**	**2.1%**	**0.79 [0.03, 19.23]**
Total events	1		0			
Heterogeneity: Not applicable						
Test for overall effect: Z = 0.15 (P = 0.88)						
**3.10.9 Myalgia (systemic adverse reaction)**						
Han 2021	1	425	0	111	2.1%	0.79 [0.03, 19.23]
Xia 2021	1	751	0	248	2.0%	0.99 [0.04, 24.31]
**Subtotal (95% CI)**		**1176**		**359**	**4.1%**	**0.89 [0.09, 8.49]**
Total events	2		0			
Heterogeneity: Chi² = 0.01, df = 1 (P = 0.92); I² = 0%						
Test for overall effect: Z = 0.10 (P = 0.92)						
**3.10.10 Nausea (systemic adverse reaction)**						
Han 2021	3	425	0	111	2.1%	1.84 [0.10, 35.37]
Xia 2021	2	751	0	248	2.0%	1.66 [0.08, 34.37]
**Subtotal (95% CI)**		**1176**		**359**	**4.1%**	**1.75 [0.21, 14.54]**
Total events	5		0			
Heterogeneity: Chi² = 0.00, df = 1 (P = 0.96); I² = 0%						
Test for overall effect: Z = 0.52 (P = 0.60)						
**3.10.11 Vomiting (systemic adverse reaction)**						
Han 2021	2	425	1	111	4.2%	0.52 [0.05, 5.71]
Xia 2021	2	751	0	248	2.0%	1.66 [0.08, 34.37]
**Subtotal (95% CI)**		**1176**		**359**	**6.2%**	**0.89 [0.14, 5.49]**
Total events	4		1			
Heterogeneity: Chi² = 0.35, df = 1 (P = 0.55); I² = 0%						
Test for overall effect: Z = 0.13 (P = 0.90)						
**3.10.12 Diarrhea (systemic adverse reaction)**						
Han 2021	1	425	1	111	4.2%	0.26 [0.02, 4.14]
**Subtotal (95% CI)**		**425**		**111**	**4.2%**	**0.26 [0.02, 4.14]**
Total events	1		1			
Heterogeneity: Not applicable						
Test for overall effect: Z = 0.95 (P = 0.34)						
**3.10.13 Anorexia (systemic adverse reaction)**						
Han 2021	4	425	0	111	2.1%	2.37 [0.13, 43.62]
**Subtotal (95% CI)**		**425**		**111**	**2.1%**	**2.37 [0.13, 43.62]**
Total events	4		0			
Heterogeneity: Not applicable						
Test for overall effect: Z = 0.58 (P = 0.56)						
**3.10.14 Acute allergic reaction/ Hypersensitivity (systemic adverse reaction)**						
Han 2021	0	425	1	111	6.3%	0.09 [0.00, 2.14]
Xia 2021	1	751	0	248	2.0%	0.99 [0.04, 24.31]
**Subtotal (95% CI)**		**1176**		**359**	**8.3%**	**0.31 [0.05, 2.04]**
Total events	1		1			
Heterogeneity: Chi² = 1.11, df = 1 (P = 0.29); I² = 10%						
Test for overall effect: Z = 1.22 (P = 0.22)						
**3.10.15 Abnormal skin and mucosa (systemic adverse reaction)**						
Han 2021	1	425	1	111	4.2%	0.26 [0.02, 4.14]
**Subtotal (95% CI)**		**425**		**111**	**4.2%**	**0.26 [0.02, 4.14]**
Total events	1		1			
Heterogeneity: Not applicable						
Test for overall effect: Z = 0.95 (P = 0.34)						
**3.10.16 Dyspnoea (systemic adverse reaction)**						
Xia 2021	1	751	0	248	2.0%	0.99 [0.04, 24.31]
**Subtotal (95% CI)**		**751**		**248**	**2.0%**	**0.99 [0.04, 24.31]**
Total events	1		0			
Heterogeneity: Not applicable						
Test for overall effect: Z = 0.00 (P = 1.00)						
**Total (95% CI)**		**16138**		**4889**	**100.0%**	**1.84 [1.20, 2.81]**
Total events	146		16			
Heterogeneity: Chi² = 16.53, df = 26 (P = 0.92); I² = 0%						
Test for overall effect: Z = 2.82 (P = 0.005)						
Test for subgroup differences: Chi² = 12.87, df = 15 (P = 0.61), I² = 0%						

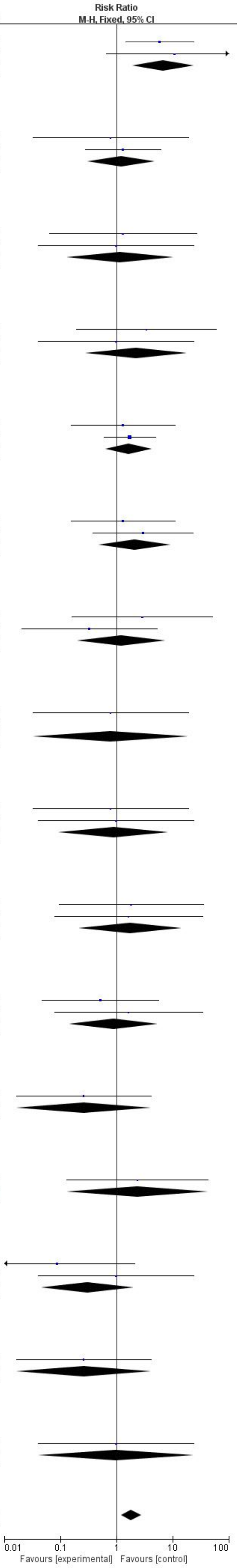

Supplementary Figure 7. Specific adverse reactions in vectored vaccine group versus control group:

A) After dose 1

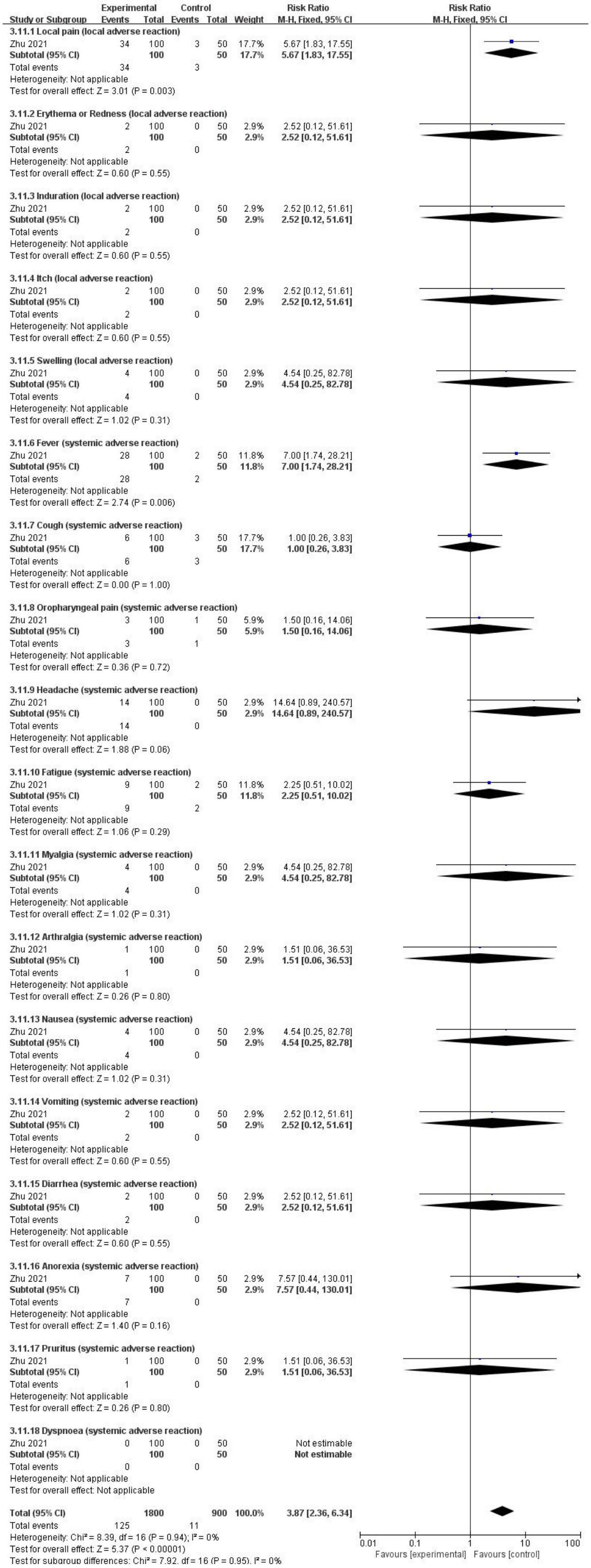

B) After dose 2

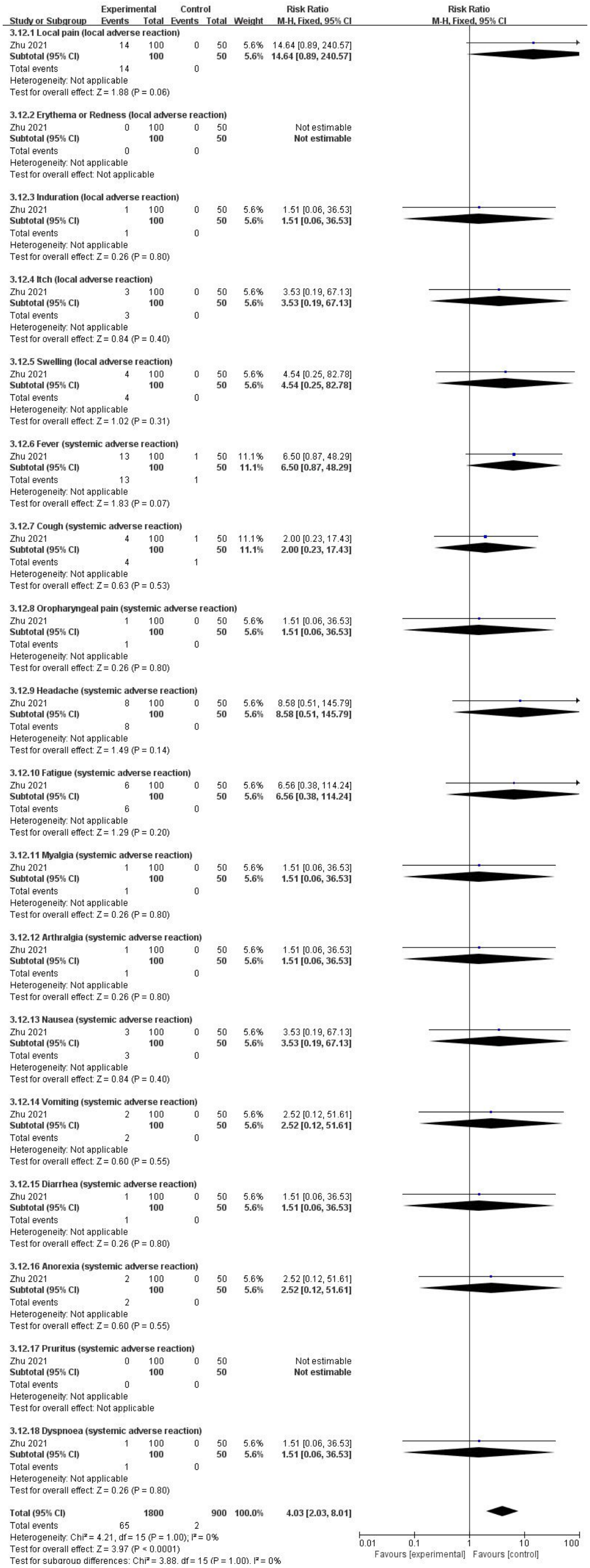

Supplementary Figure 8. Adverse reactions in mRNA vaccine group of different ages versus control group:

A) Local pain after the first vaccination

Study or Subgroup	Experimental Events	Experimental Total	Control Events	Control Total	Weight	Risk Ratio M-H, Fixed, 95% CI
**4.1.1 ⩾12 years old**						
Ali 2021	2310	2482	431	1238	89.9%	2.67 [2.48, 2.89]
Frenck 2021	86	1131	23	1129	3.6%	3.73 [2.37, 5.87]
**Subtotal (95% CI)**		**3613**		**2367**	**93.5%**	**2.71 [2.51, 2.93]**
Total events	2396		454			
Heterogeneity: Chi² = 2.05, df = 1 (P = 0.15); I² = 51%						
Test for overall effect: Z = 25.67 (P < 0.00001)						
**4.1.2 <12 years old**						
Walter 2021	74	1511	31	749	6.5%	1.18 [0.79, 1.78]
**Subtotal (95% CI)**		**1511**		**749**	**6.5%**	**1.18 [0.79, 1.78]**
Total events	74		31			
Heterogeneity: Not applicable						
Test for overall effect: Z = 0.80 (P = 0.42)						
**Total (95% CI)**		**5124**		**3116**	**100.0%**	**2.61 [2.43, 2.82]**
Total events	2470		485			
Heterogeneity: Chi² = 17.05, df = 2 (P = 0.0002); I² = 88%						
Test for overall effect: Z = 25.18 (P < 0.00001)						
Test for subgroup differences: Chi² = 15.22, df = 1 (P < 0.0001), I² = 93.4%						

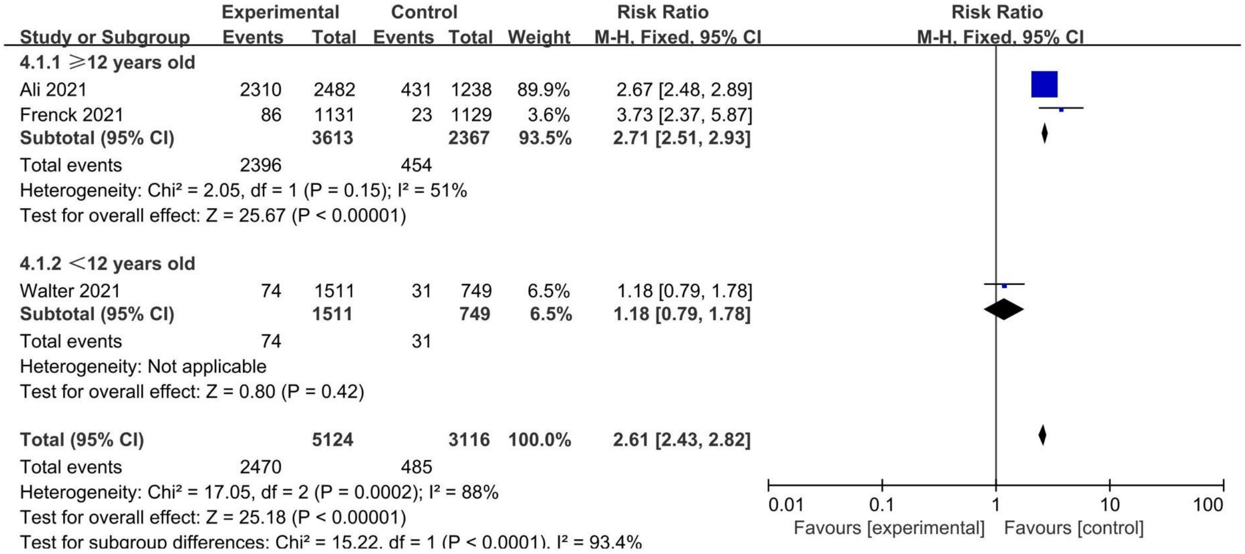

B) Local pain after the second vaccination

Study or Subgroup	Experimental Events	Experimental Total	Control Events	Control Total	Weight	Risk Ratio M-H, Fixed, 95% CI
**4.2.1 ⩾12 years old**						
Ali 2021	2290	2478	370	1220	89.7%	3.05 [2.80, 3.32]
Frenck 2021	79	1131	18	1129	3.3%	4.38 [2.64, 7.26]
**Subtotal (95% CI)**		**3609**		**2349**	**93.0%**	**3.09 [2.84, 3.37]**
Total events	2369		388			
Heterogeneity: Chi² = 1.94, df = 1 (P = 0.16); I² = 49%						
Test for overall effect: Z = 26.10 (P < 0.00001)						
**4.2.2 <12 years old**						
Walter 2021	71	1501	29	741	7.0%	1.21 [0.79, 1.84]
**Subtotal (95% CI)**		**1501**		**741**	**7.0%**	**1.21 [0.79, 1.84]**
Total events	71		29			
Heterogeneity: Not applicable						
Test for overall effect: Z = 0.88 (P = 0.38)						
**Total (95% CI)**		**5110**		**3090**	**100.0%**	**2.96 [2.73, 3.22]**
Total events	2440		417			
Heterogeneity: Chi² = 19.99, df = 2 (P < 0.0001); I² = 90%						
Test for overall effect: Z = 25.70 (P < 0.00001)						
Test for subgroup differences: Chi² = 18.24, df = 1 (P < 0.0001), I² = 94.5%						

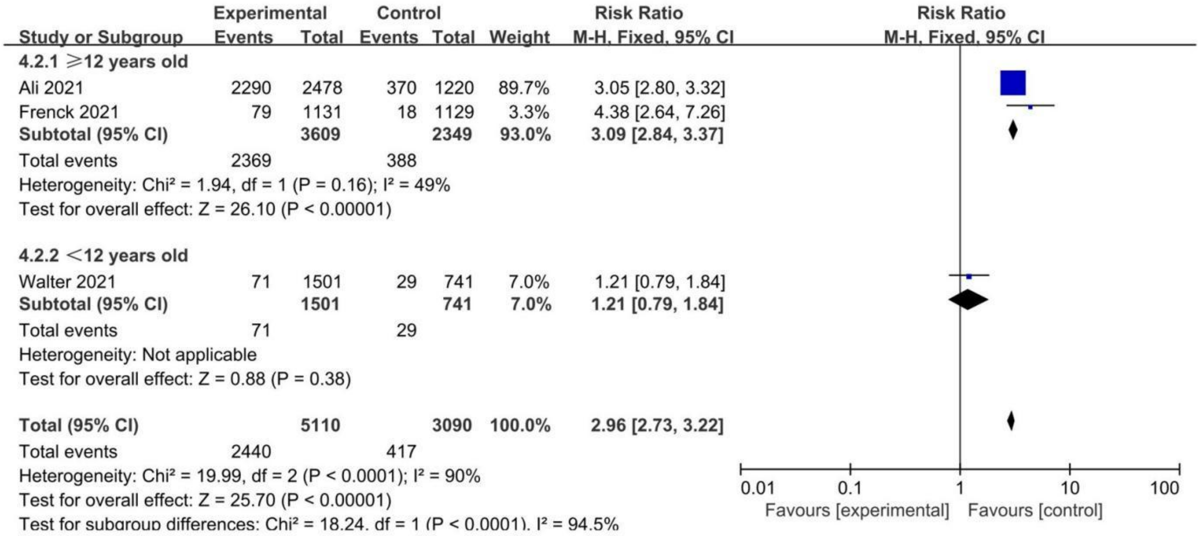

C) Erythema or Redness after the first vaccination

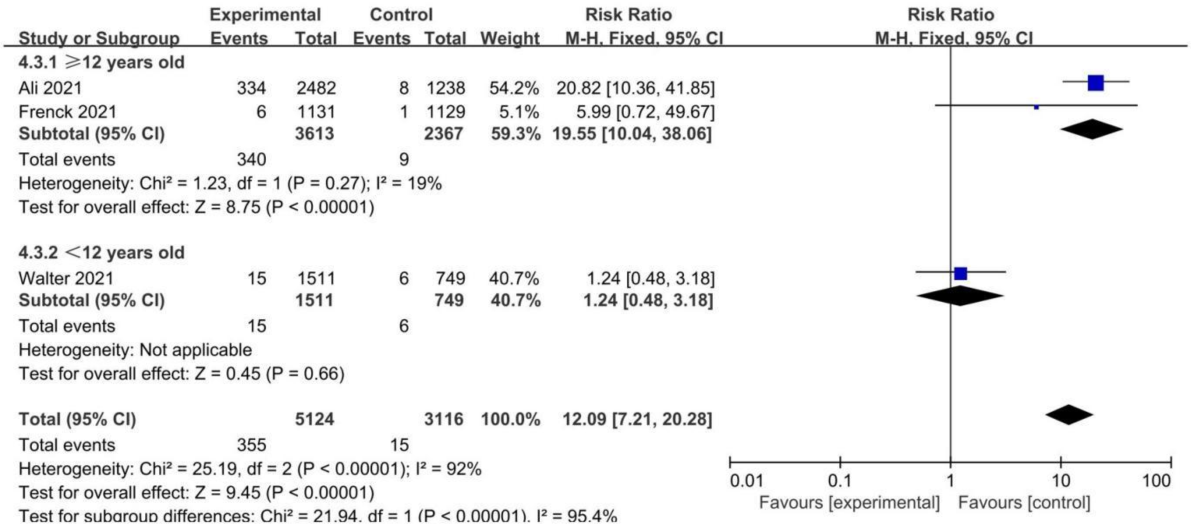

D) Erythema or Redness after the second vaccination

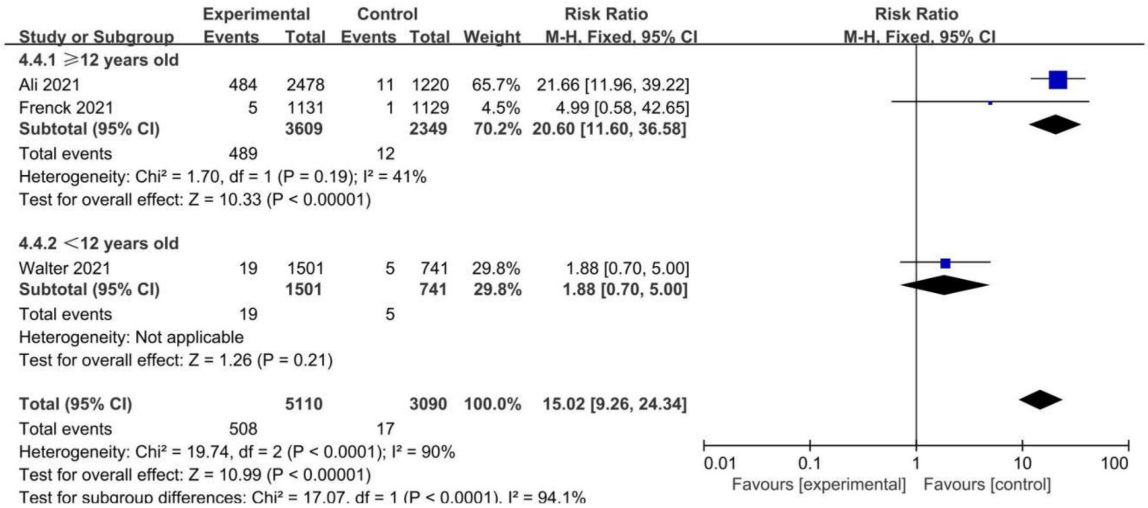

E) Swelling after the first vaccination

Study or Subgroup	Experimental Events	Experimental Total	Control Events	Control Total	Weight	Risk Ratio M-H, Fixed, 95% CI
**4.5.1 ≥12 years old**						
Ali 2021	403	2482	12	1238	76.2%	16.75 [9.47, 29.62]
Frenck 2021	7	1131	1	1129	4.8%	6.99 [0.86, 56.70]
**Subtotal (95% CI)**		**3613**		**2367**	**80.9%**	**16.18 [9.31, 28.09]**
Total events	410		13			
Heterogeneity: Chi² = 0.63, df = 1 (P = 0.43); I² = 0%						
Test for overall effect: Z = 9.88 (P < 0.00001)						
**4.5.2 <12 years old**						
Walter 2021	10	1511	3	749	19.1%	1.65 [0.46, 5.99]
**Subtotal (95% CI)**		**1511**		**749**	**19.1%**	**1.65 [0.46, 5.99]**
Total events	10		3			
Heterogeneity: Not applicable						
Test for overall effect: Z = 0.76 (P = 0.44)						
**Total (95% CI)**		**5124**		**3116**	**100.0%**	**13.41 [8.15, 22.06]**
Total events	420		16			
Heterogeneity: Chi² = 11.12, df = 2 (P = 0.004); I² = 82%						
Test for overall effect: Z = 10.21 (P < 0.00001)						
Test for subgroup differences: Chi² = 10.19, df = 1 (P = 0.001), I² = 90.2%						

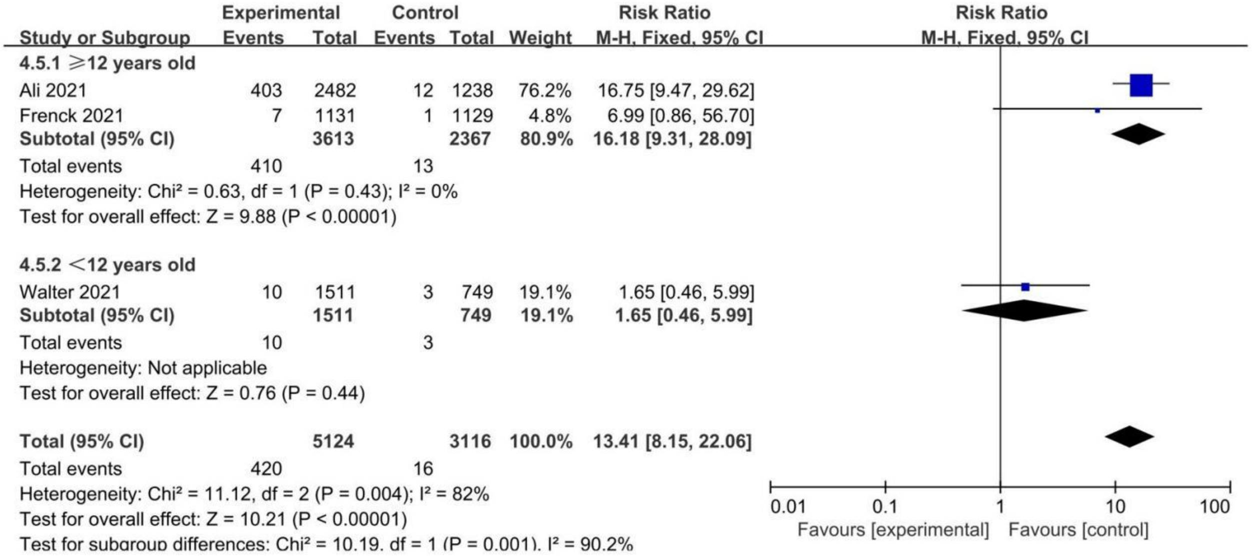

F) Swelling after the second vaccination

Study or Subgroup	Experimental Events	Experimental Total	Control Events	Control Total	Weight	Risk Ratio M-H, Fixed, 95% CI
**4.6.1 ≥12 years old**						
Ali 2021	509	2478	12	1220	76.2%	20.88 [11.83, 36.86]
Frenck 2021	5	1131	1	1129	4.7%	4.99 [0.58, 42.65]
**Subtotal (95% CI)**		**3609**		**2349**	**81.0%**	**19.95 [11.50, 34.61]**
Total events	514		13			
Heterogeneity: Chi² = 1.63, df = 1 (P = 0.20); I² = 39%						
Test for overall effect: Z = 10.65 (P < 0.00001)						
**4.6.2 <12 years old**						
Walter 2021	15	1501	3	741	19.0%	2.47 [0.72, 8.50]
**Subtotal (95% CI)**		**1501**		**741**	**19.0%**	**2.47 [0.72, 8.50]**
Total events	15		3			
Heterogeneity: Not applicable						
Test for overall effect: Z = 1.43 (P = 0.15)						
**Total (95% CI)**		**5110**		**3090**	**100.0%**	**16.62 [10.11, 27.33]**
Total events	529		16			
Heterogeneity: Chi² = 10.97, df = 2 (P = 0.004); I² = 82%						
Test for overall effect: Z = 11.08 (P < 0.00001)						
Test for subgroup differences: Chi² = 9.16, df = 1 (P = 0.002), I² = 89.1%						

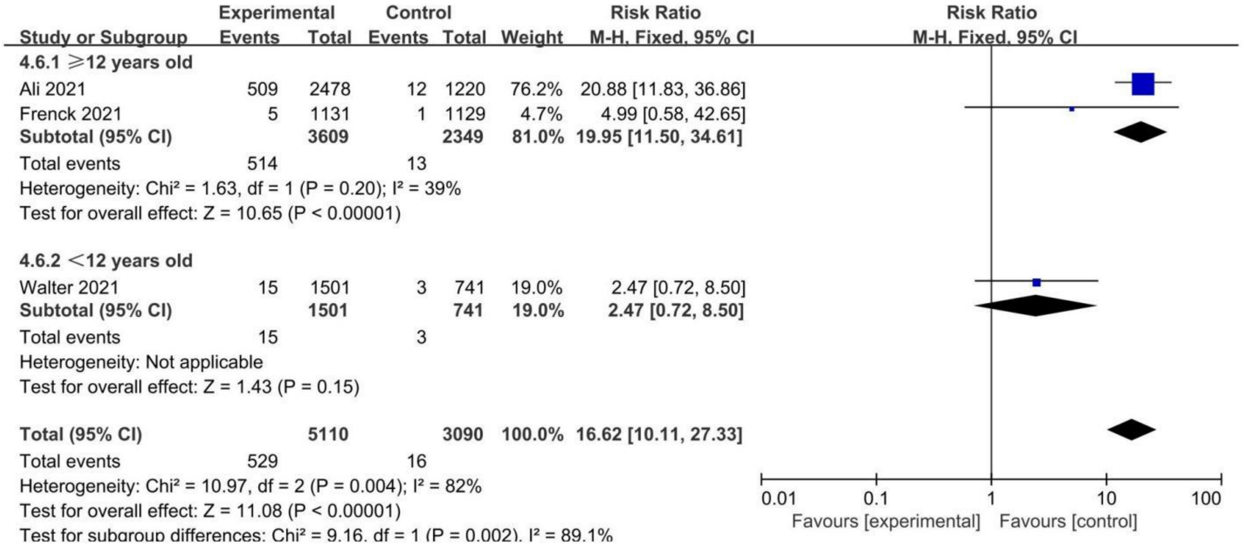

G) Fever after the first vaccination

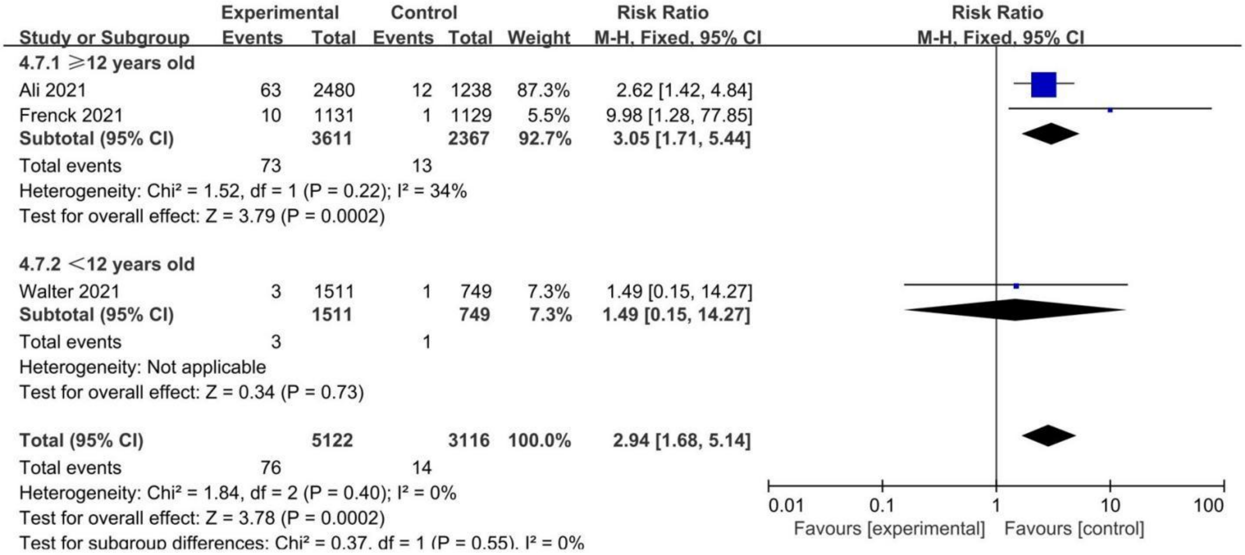

H) Fever after the second vaccination

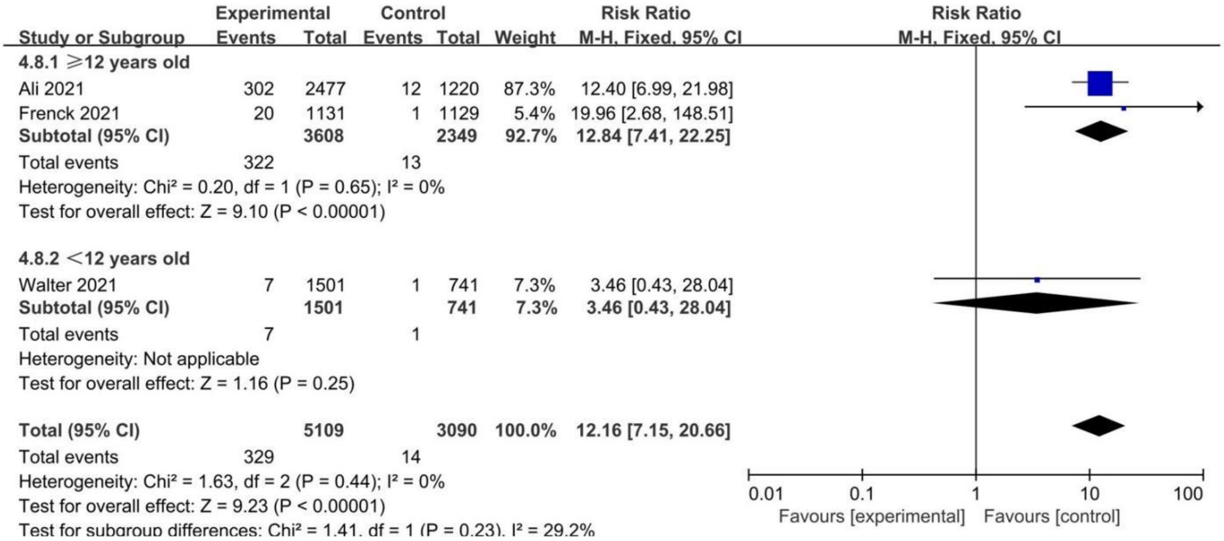

I) Headache after the first vaccination

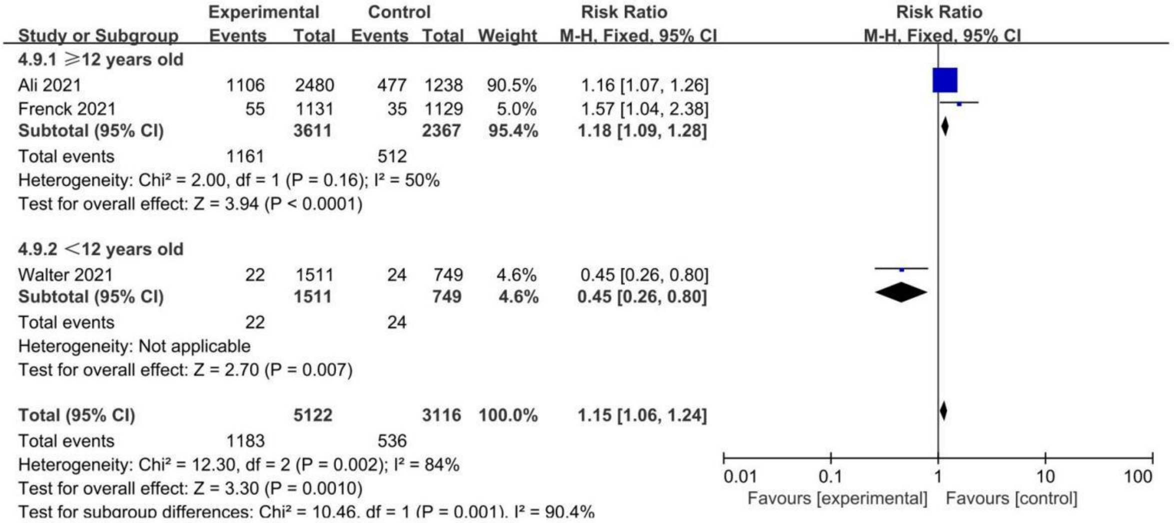

J) Headache after the second vaccination

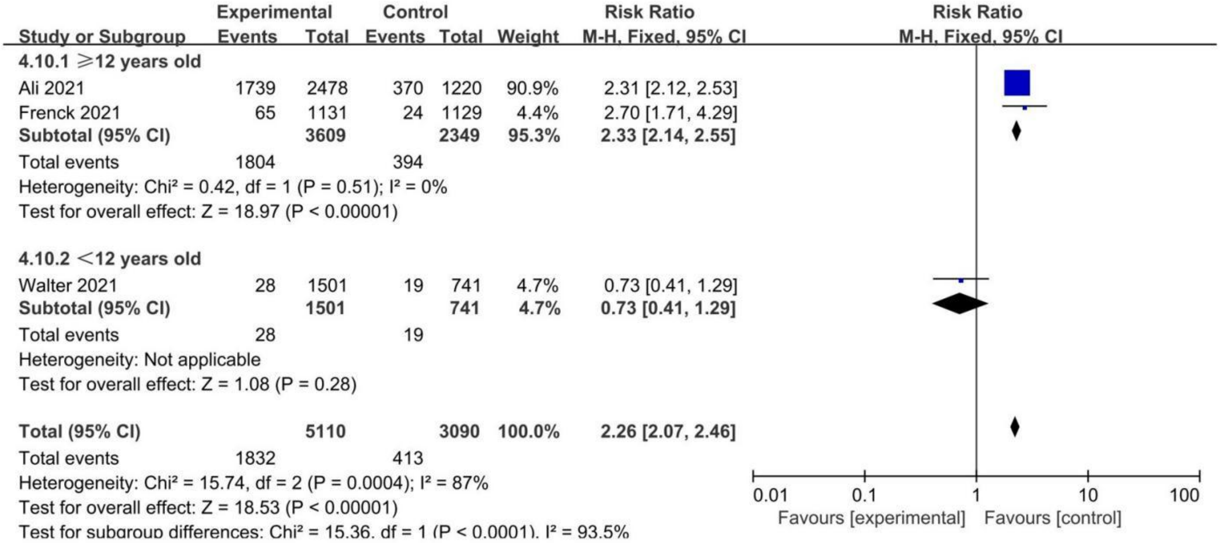

K) Fatigue after the first vaccination

Study or Subgroup	Experimental Events	Experimental Total	Control Events	Control Total	Weight	Risk Ratio M-H, Fixed, 95% CI
**4.11.1 ⩾12 years old**						
Ali 2021	1188	2481	453	1238	88.0%	1.31 [1.20, 1.42]
Frenck 2021	60	1131	41	1129	6.0%	1.46 [0.99, 2.15]
**Subtotal (95% CI)**		**3612**		**2367**	**94.0%**	**1.32 [1.21, 1.43]**
Total events	1248		494			
Heterogeneity: Chi² = 0.30, df = 1 (P = 0.59); I² = 0%						
Test for overall effect: Z = 6.56 (P < 0.00001)						
**4.11.2 <12 years old**						
Walter 2021	34	1511	31	749	6.0%	0.54 [0.34, 0.88]
**Subtotal (95% CI)**		**1511**		**749**	**6.0%**	**0.54 [0.34, 0.88]**
Total events	34		31			
Heterogeneity: Not applicable						
Test for overall effect: Z = 2.49 (P = 0.01)						
**Total (95% CI)**		**5123**		**3116**	**100.0%**	**1.27 [1.17, 1.38]**
Total events	1282		525			
Heterogeneity: Chi² = 13.04, df = 2 (P = 0.001); I² = 85%						
Test for overall effect: Z = 5.79 (P < 0.00001)						
Test for subgroup differences: Chi² = 12.77, df = 1 (P = 0.0004), I² = 92.2%						

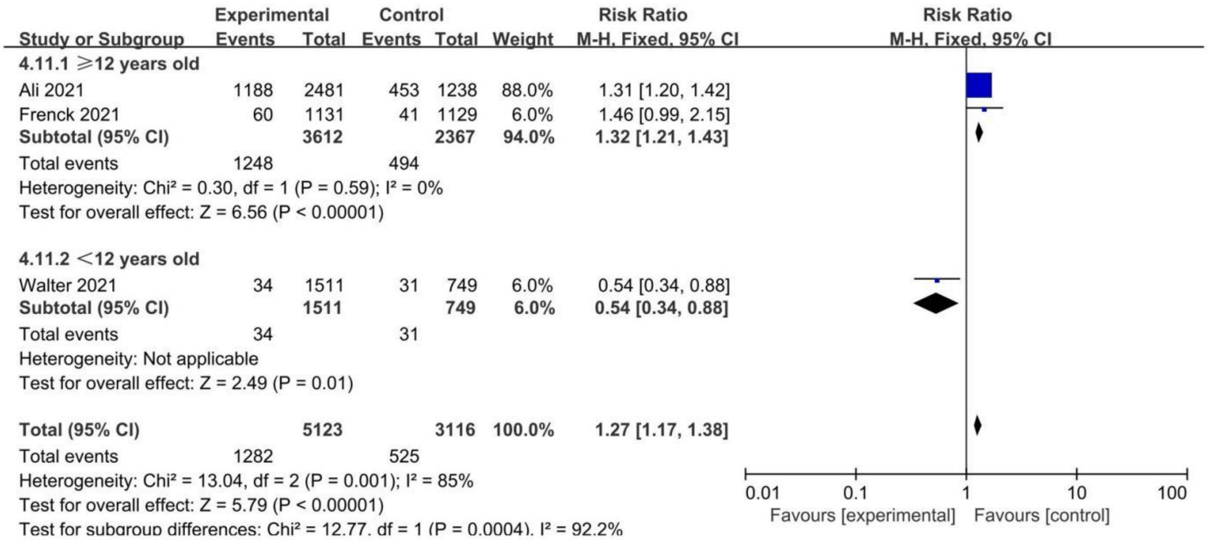

L) Fatigue after the second vaccination

Study or Subgroup	Experimental Events	Experimental Total	Control Events	Control Total	Weight	Risk Ratio M-H, Fixed, 95% CI
**4.12.1 ⩾12 years old**						
Ali 2021	1679	2478	353	1220	89.2%	2.34 [2.14, 2.57]
Frenck 2021	66	1131	25	1129	4.7%	2.64 [1.68, 4.14]
**Subtotal (95% CI)**		**3609**		**2349**	**93.9%**	**2.36 [2.15, 2.58]**
Total events	1745		378			
Heterogeneity: Chi² = 0.25, df = 1 (P = 0.62); I² = 0%						
Test for overall effect: Z = 18.58 (P < 0.00001)						
**4.12.2 <12 years old**						
Walter 2021	39	1501	24	741	6.1%	0.80 [0.49, 1.32]
**Subtotal (95% CI)**		**1501**		**741**	**6.1%**	**0.80 [0.49, 1.32]**
Total events	39		24			
Heterogeneity: Not applicable						
Test for overall effect: Z = 0.86 (P = 0.39)						
**Total (95% CI)**		**5110**		**3090**	**100.0%**	**2.26 [2.07, 2.47]**
Total events	1784		402			
Heterogeneity: Chi² = 17.44, df = 2 (P = 0.0002); I² = 89%						
Test for overall effect: Z = 18.07 (P < 0.00001)						
Test for subgroup differences: Chi² = 17.22, df = 1 (P < 0.0001), I² = 94.2%						

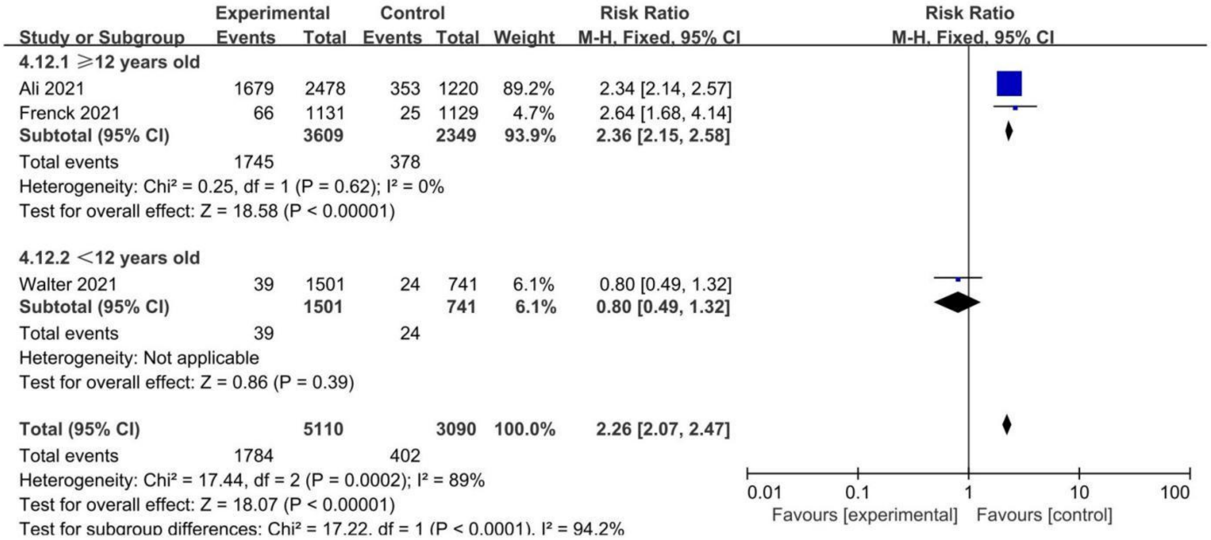

M) Myalgia after the first vaccination

Study or Subgroup	Experimental Events	Experimental Total	Control Events	Control Total	Weight	Risk Ratio M-H, Fixed, 95% CI
**4.13.1 ≥12 years old**						
Ali 2021	668	2480	205	1238	92.4%	1.63 [1.41, 1.87]
Frenck 2021	24	1131	13	1129	4.4%	1.84 [0.94, 3.60]
**Subtotal (95% CI)**		**3611**		**2367**	**96.8%**	**1.64 [1.43, 1.88]**
Total events	692		218			
Heterogeneity: Chi² = 0.13, df = 1 (P = 0.72); I² = 0%						
Test for overall effect: Z = 7.00 (P < 0.00001)						
**4.13.2 <12 years old**						
Walter 2021	9	1511	7	749	3.2%	0.64 [0.24, 1.70]
**Subtotal (95% CI)**		**1511**		**749**	**3.2%**	**0.64 [0.24, 1.70]**
Total events	9		7			
Heterogeneity: Not applicable						
Test for overall effect: Z = 0.90 (P = 0.37)						
**Total (95% CI)**		**5122**		**3116**	**100.0%**	**1.60 [1.40, 1.84]**
Total events	701		225			
Heterogeneity: Chi² = 3.58, df = 2 (P = 0.17); I² = 44%						
Test for overall effect: Z = 6.80 (P < 0.00001)						
Test for subgroup differences: Chi² = 3.46, df = 1 (P = 0.06), I² = 71.1%						

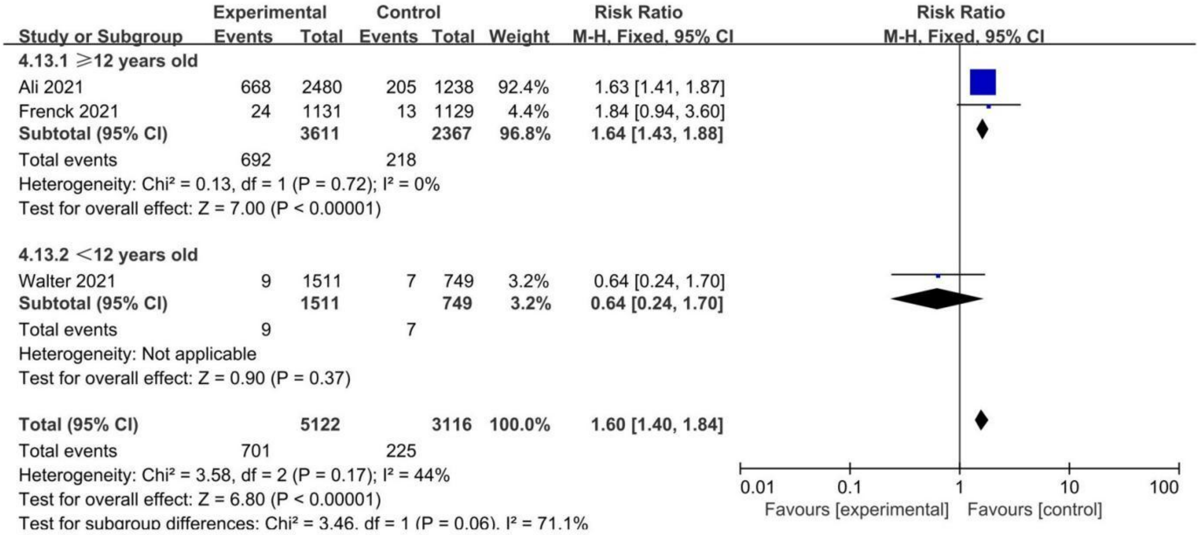

N) Myalgia after the second vaccination

Study or Subgroup	Experimental Events	Experimental Total	Control Events	Control Total	Weight	Risk Ratio M-H, Fixed, 95% CI
**4.14.1 ≥12 years old**						
Ali 2021	1154	2477	153	1220	92.2%	3.71 [3.18, 4.33]
Frenck 2021	32	1131	8	1129	3.6%	3.99 [1.85, 8.63]
**Subtotal (95% CI)**		**3608**		**2349**	**95.8%**	**3.73 [3.20, 4.33]**
Total events	1186		161			
Heterogeneity: Chi² = 0.03, df = 1 (P = 0.86); I² = 0%						
Test for overall effect: Z = 17.06 (P < 0.00001)						
**4.14.2 <12 years old**						
Walter 2021	12	1501	7	741	4.2%	0.85 [0.33, 2.14]
**Subtotal (95% CI)**		**1501**		**741**	**4.2%**	**0.85 [0.33, 2.14]**
Total events	12		7			
Heterogeneity: Not applicable						
Test for overall effect: Z = 0.35 (P = 0.72)						
**Total (95% CI)**		**5109**		**3090**	**100.0%**	**3.60 [3.11, 4.18]**
Total events	1198		168			
Heterogeneity: Chi² = 9.58, df = 2 (P = 0.008); I² = 79%						
Test for overall effect: Z = 16.94 (P < 0.00001)						
Test for subgroup differences: Chi² = 9.55, df = 1 (P = 0.002), I² = 89.5%						

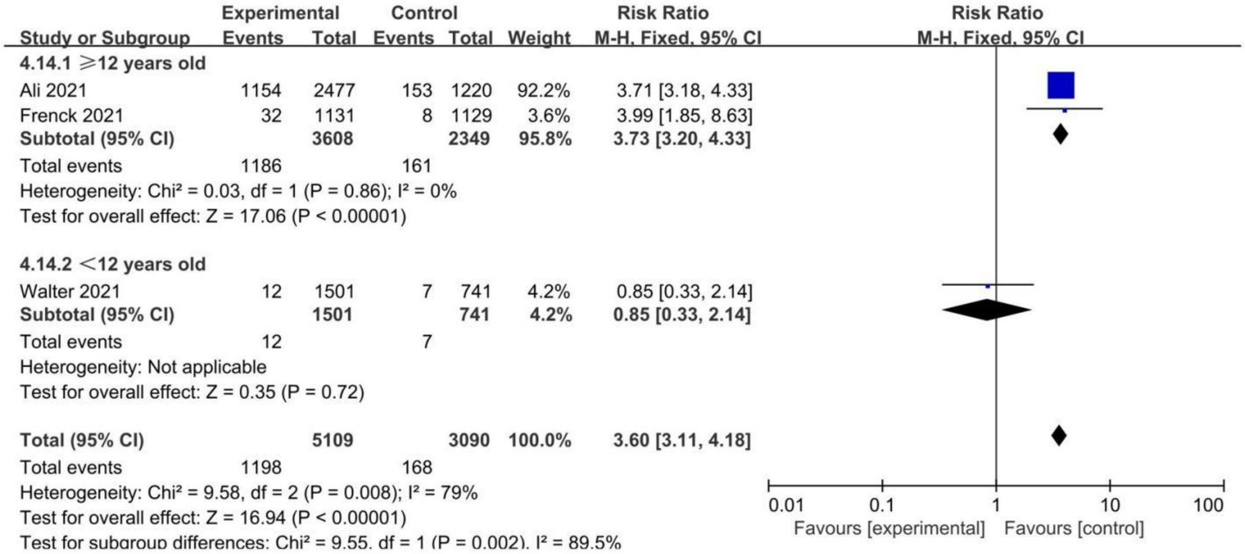

O) Arthralgia after the first vaccination

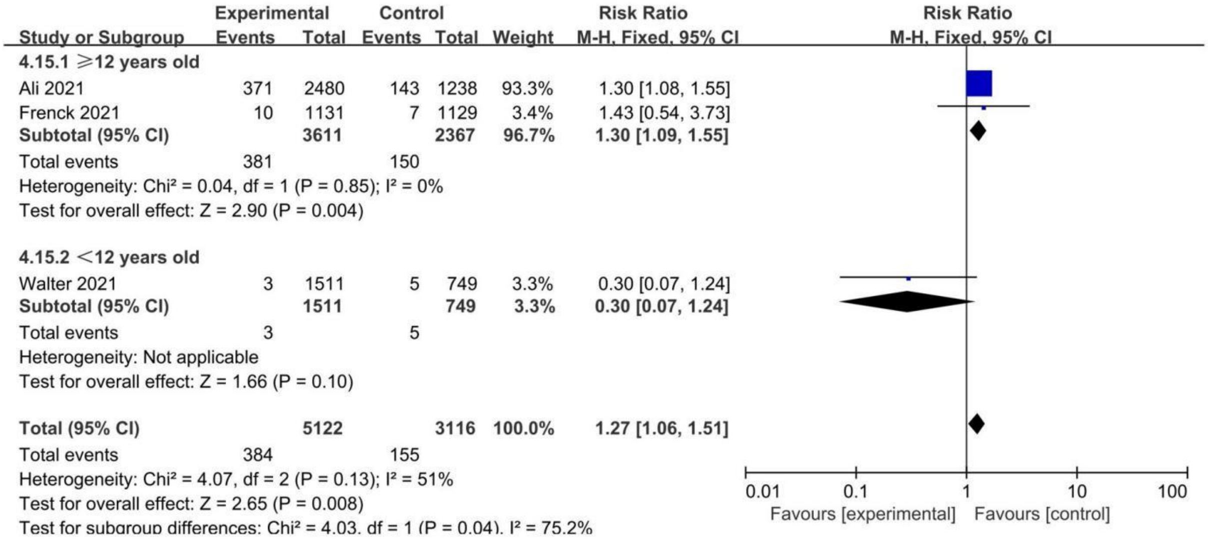

P) Arthralgia after the second vaccination

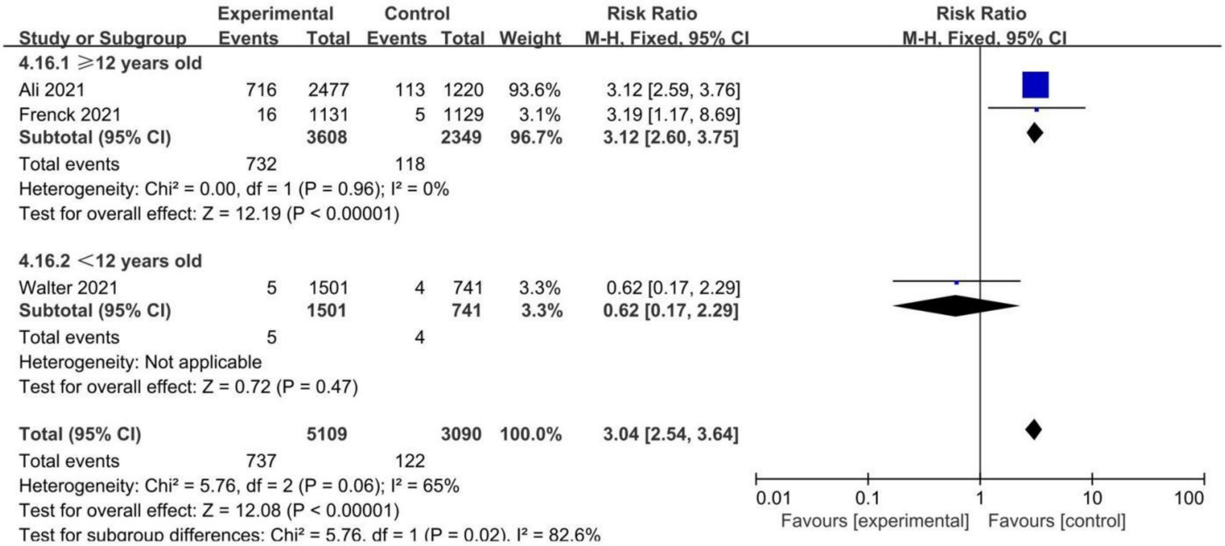

Q) Vomiting after the first vaccination

Study or Subgroup	Experimental Events	Experimental Total	Control Events	Control Total	Weight	Risk Ratio M-H, Fixed, 95% CI
**4.17.1 ≥12 years old**						
Frenck 2021	3	1131	1	1129	42.8%	2.99 [0.31, 28.75]
**Subtotal (95% CI)**		**1131**		**1129**	**42.8%**	**2.99 [0.31, 28.75]**
Total events	3		1			
Heterogeneity: Not applicable						
Test for overall effect: Z = 0.95 (P = 0.34)						
**4.17.2 <12 years old**						
Walter 2021	2	1511	1	749	57.2%	0.99 [0.09, 10.92]
**Subtotal (95% CI)**		**1511**		**749**	**57.2%**	**0.99 [0.09, 10.92]**
Total events	2		1			
Heterogeneity: Not applicable						
Test for overall effect: Z = 0.01 (P = 0.99)						
**Total (95% CI)**		**2642**		**1878**	**100.0%**	**1.85 [0.38, 9.07]**
Total events	5		2			

Heterogeneity: Chi² = 0.43, df = 1 (P = 0.51); I² = 0%
Test for overall effect: Z = 0.76 (P = 0.45)
Test for subgroup differences: Chi² = 0.43, df = 1 (P = 0.51), I² = 0%

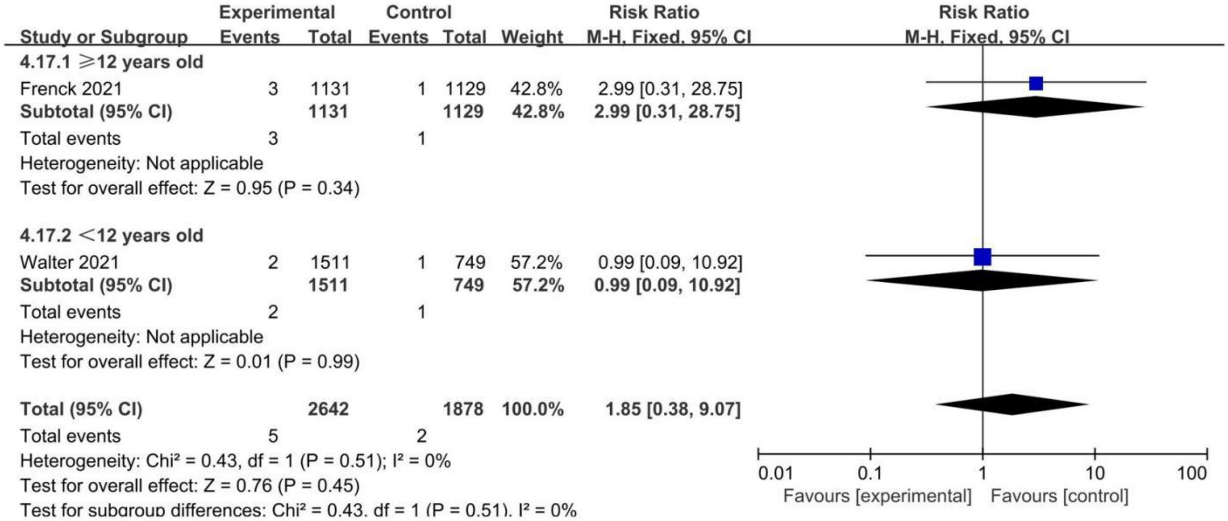

R) Vomiting after the second vaccination

Study or Subgroup	Experimental Events	Experimental Total	Control Events	Control Total	Weight	Risk Ratio M-H, Fixed, 95% CI
**4.18.1 ≥12 years old**						
Frenck 2021	3	1131	1	1129	42.8%	2.99 [0.31, 28.75]
**Subtotal (95% CI)**		**1131**		**1129**	**42.8%**	**2.99 [0.31, 28.75]**
Total events	3		1			
Heterogeneity: Not applicable						
Test for overall effect: Z = 0.95 (P = 0.34)						
**4.18.2 <12 years old**						
Walter 2021	2	1501	1	741	57.2%	0.99 [0.09, 10.87]
**Subtotal (95% CI)**		**1501**		**741**	**57.2%**	**0.99 [0.09, 10.87]**
Total events	2		1			
Heterogeneity: Not applicable						
Test for overall effect: Z = 0.01 (P = 0.99)						
**Total (95% CI)**		**2632**		**1870**	**100.0%**	**1.85 [0.38, 9.05]**
Total events	5		2			

Heterogeneity: Chi² = 0.44, df = 1 (P = 0.51); I² = 0%
Test for overall effect: Z = 0.76 (P = 0.45)
Test for subgroup differences: Chi² = 0.44, df = 1 (P = 0.51), I² = 0%

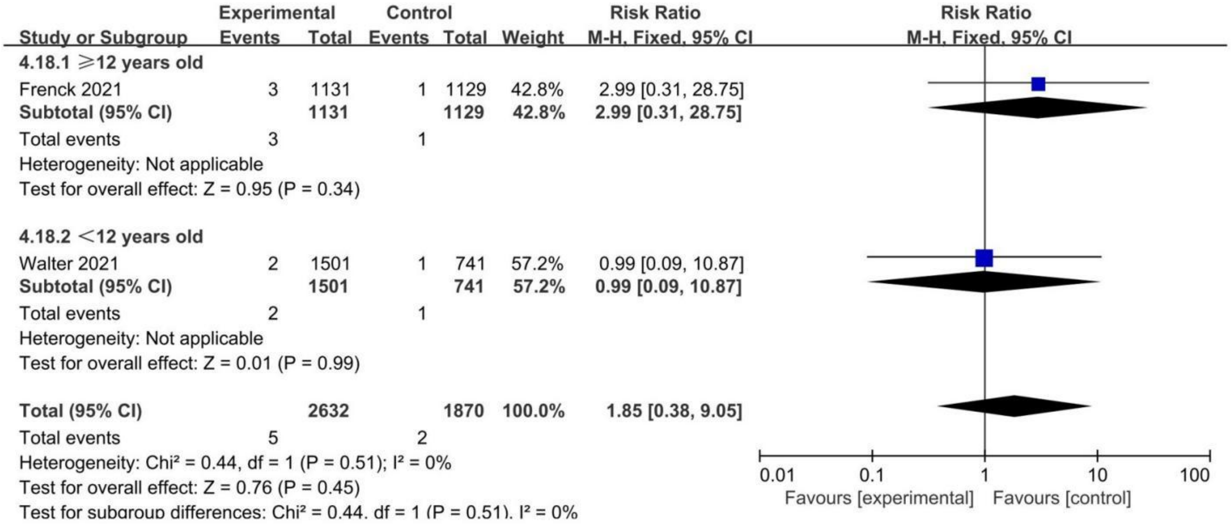

S) Diarrhea after the first vaccination

Study or Subgroup	Experimental Events	Experimental Total	Control Events	Control Total	Weight	Risk Ratio M-H, Fixed, 95% CI
**4.19.1 ⩾12 years old**						
Frenck 2021	8	1131	7	1129	56.7%	1.14 [0.42, 3.14]
**Subtotal (95% CI)**		**1131**		**1129**	**56.7%**	**1.14 [0.42, 3.14]**
Total events	8		7			
Heterogeneity: Not applicable						
Test for overall effect: Z = 0.26 (P = 0.80)						
**4.19.2 <12 years old**						
Walter 2021	6	1511	4	749	43.3%	0.74 [0.21, 2.63]
**Subtotal (95% CI)**		**1511**		**749**	**43.3%**	**0.74 [0.21, 2.63]**
Total events	6		4			
Heterogeneity: Not applicable						
Test for overall effect: Z = 0.46 (P = 0.65)						
**Total (95% CI)**		**2642**		**1878**	**100.0%**	**0.97 [0.44, 2.12]**
Total events	14		11			
Heterogeneity: Chi² = 0.27, df = 1 (P = 0.60); I² = 0%						
Test for overall effect: Z = 0.08 (P = 0.94)						
Test for subgroup differences: Chi² = 0.27, df = 1 (P = 0.60), I² = 0%						

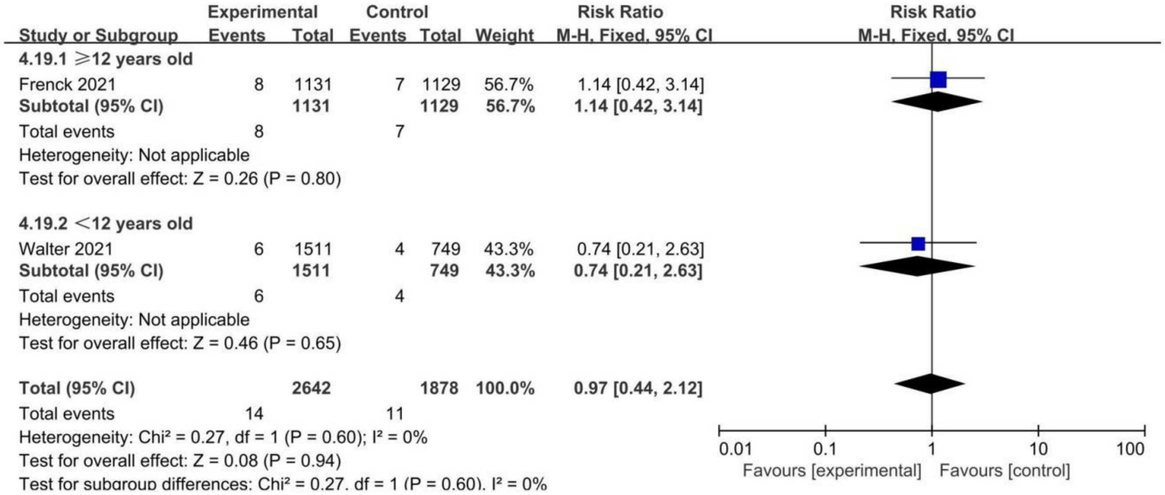

T) Diarrhea after the second vaccination

Study or Subgroup	Experimental Events	Experimental Total	Control Events	Control Total	Weight	Risk Ratio M-H, Fixed, 95% CI
**4.20.1 ⩾12 years old**						
Frenck 2021	6	1131	4	1129	30.7%	1.50 [0.42, 5.29]
**Subtotal (95% CI)**		**1131**		**1129**	**30.7%**	**1.50 [0.42, 5.29]**
Total events	6		4			
Heterogeneity: Not applicable						
Test for overall effect: Z = 0.63 (P = 0.53)						
**4.20.2 <12 years old**						
Walter 2021	5	1501	5	156	69.3%	0.10 [0.03, 0.36]
**Subtotal (95% CI)**		**1501**		**156**	**69.3%**	**0.10 [0.03, 0.36]**
Total events	5		5			
Heterogeneity: Not applicable						
Test for overall effect: Z = 3.61 (P = 0.0003)						
**Total (95% CI)**		**2632**		**1285**	**100.0%**	**0.53 [0.25, 1.13]**
Total events	11		9			
Heterogeneity: Chi² = 9.36, df = 1 (P = 0.002); I² = 89%						
Test for overall effect: Z = 1.65 (P = 0.10)						
Test for subgroup differences: Chi² = 8.81, df = 1 (P = 0.003), I² = 88.6%						

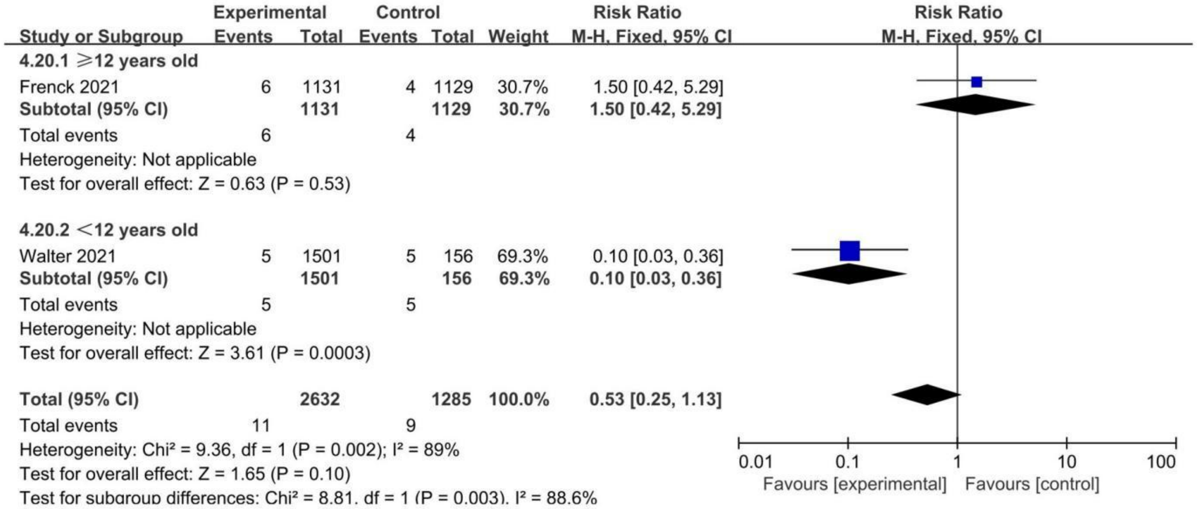

U) Chills after the first vaccination

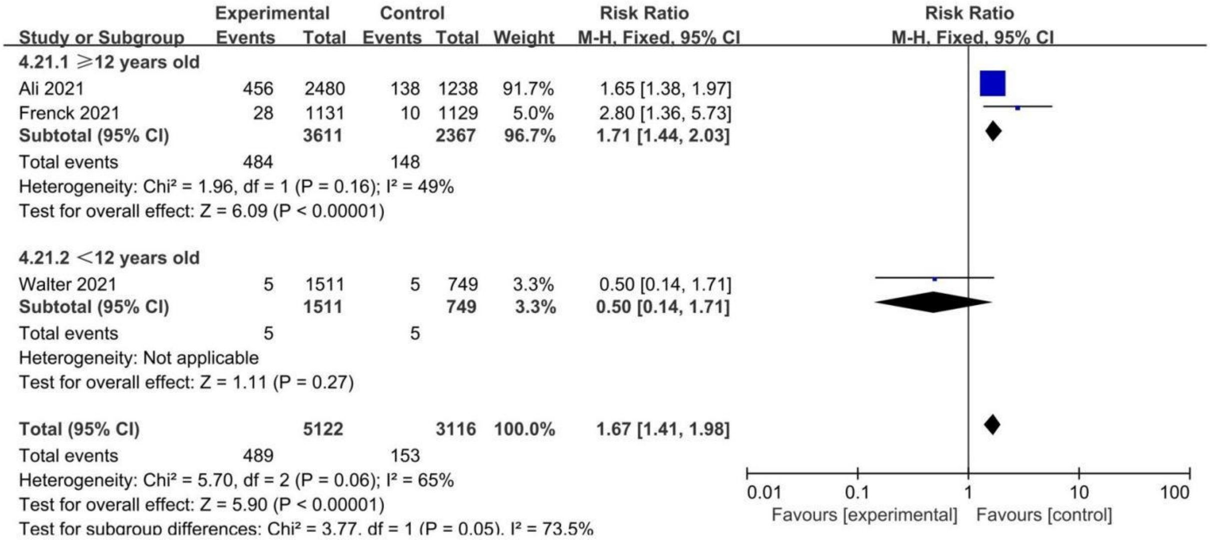

V) Chills after the second vaccination

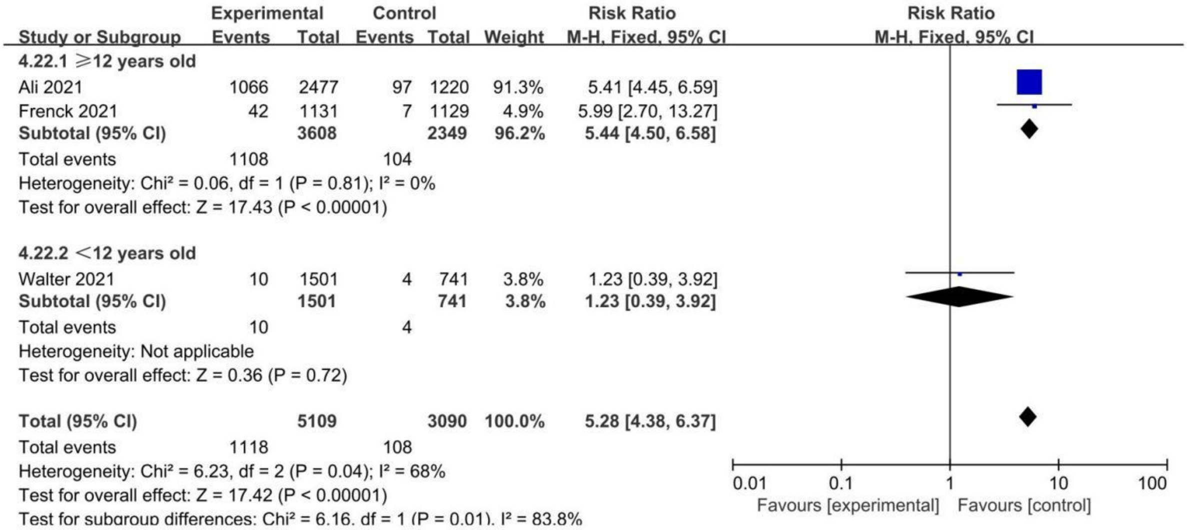

Supplementary Figure 9. Specific adverse reactions in mRNA vaccine recipients aged ≥12 years versus <12 years:

A) After dose 1

Study or Subgroup	12 - 15 years old Events	12 - 15 years old Total	5 - 11 years old Events	5 - 11 years old Total	Weight	Risk Ratio M-H, Fixed, 95% CI
**5.5.1 Local pain**						
Frenck 2021	86	1131	23	1129	7.7%	3.73 [2.37, 5.87]
Walter 2021	74	1511	31	749	13.9%	1.18 [0.79, 1.78]
**Subtotal (95% CI)**		**2642**		**1878**	**21.6%**	**2.09 [1.56, 2.81]**
Total events	160		54			
Heterogeneity: Chi² = 13.71, df = 1 (P = 0.0002); I² = 93%						
Test for overall effect: Z = 4.94 (P < 0.00001)						
**5.5.2 Erythema or redness**						
Frenck 2021	6	1131	1	1129	0.3%	5.99 [0.72, 49.67]
Walter 2021	15	1511	6	749	2.7%	1.24 [0.48, 3.18]
**Subtotal (95% CI)**		**2642**		**1878**	**3.0%**	**1.77 [0.77, 4.03]**
Total events	21		7			
Heterogeneity: Chi² = 1.82, df = 1 (P = 0.18); I² = 45%						
Test for overall effect: Z = 1.35 (P = 0.18)						
**5.5.3 Swelling**						
Frenck 2021	7	1131	1	1129	0.3%	6.99 [0.86, 56.70]
Walter 2021	10	1511	3	749	1.3%	1.65 [0.46, 5.99]
**Subtotal (95% CI)**		**2642**		**1878**	**1.7%**	**2.72 [0.95, 7.74]**
Total events	17		4			
Heterogeneity: Chi² = 1.36, df = 1 (P = 0.24); I² = 26%						
Test for overall effect: Z = 1.87 (P = 0.06)						
**5.5.4 Fever**						
Frenck 2021	10	1131	1	1129	0.3%	9.98 [1.28, 77.85]
Walter 2021	3	1511	1	749	0.4%	1.49 [0.15, 14.27]
**Subtotal (95% CI)**		**2642**		**1878**	**0.8%**	**5.12 [1.25, 21.01]**
Total events	13		2			
Heterogeneity: Chi² = 1.55, df = 1 (P = 0.21); I² = 36%						
Test for overall effect: Z = 2.27 (P = 0.02)						
**5.5.5 Headache**						
Frenck 2021	55	1131	35	1129	11.8%	1.57 [1.04, 2.38]
Walter 2021	22	1511	24	749	10.8%	0.45 [0.26, 0.80]
**Subtotal (95% CI)**		**2642**		**1878**	**22.5%**	**1.04 [0.75, 1.43]**
Total events	77		59			
Heterogeneity: Chi² = 11.81, df = 1 (P = 0.0006); I² = 92%						
Test for overall effect: Z = 0.21 (P = 0.83)						
**5.5.6 Fatigue**						
Frenck 2021	60	1131	41	1129	13.8%	1.46 [0.99, 2.15]
Walter 2021	34	1511	31	749	13.9%	0.54 [0.34, 0.88]
**Subtotal (95% CI)**		**2642**		**1878**	**27.7%**	**1.00 [0.75, 1.34]**
Total events	94		72			
Heterogeneity: Chi² = 9.88, df = 1 (P = 0.002); I² = 90%						
Test for overall effect: Z = 0.00 (P = 1.00)						
**5.5.7 Myalgia**						
Frenck 2021	24	1131	13	1129	4.4%	1.84 [0.94, 3.60]
Walter 2021	9	1511	7	749	3.1%	0.64 [0.24, 1.70]
**Subtotal (95% CI)**		**2642**		**1878**	**7.5%**	**1.34 [0.78, 2.29]**
Total events	33		20			
Heterogeneity: Chi² = 3.06, df = 1 (P = 0.08); I² = 67%						
Test for overall effect: Z = 1.06 (P = 0.29)						
**5.5.8 Arthralgia**						
Frenck 2021	10	1131	7	1129	2.4%	1.43 [0.54, 3.73]
Walter 2021	3	1511	5	749	2.2%	0.30 [0.07, 1.24]
**Subtotal (95% CI)**		**2642**		**1878**	**4.6%**	**0.87 [0.41, 1.85]**
Total events	13		12			
Heterogeneity: Chi² = 3.18, df = 1 (P = 0.07); I² = 69%						
Test for overall effect: Z = 0.35 (P = 0.73)						
**5.5.9 Vomiting**						
Frenck 2021	3	1131	1	1129	0.3%	2.99 [0.31, 28.75]
Walter 2021	2	1511	1	749	0.4%	0.99 [0.09, 10.92]
**Subtotal (95% CI)**		**2642**		**1878**	**0.8%**	**1.85 [0.38, 9.07]**
Total events	5		2			
Heterogeneity: Chi² = 0.43, df = 1 (P = 0.51); I² = 0%						
Test for overall effect: Z = 0.76 (P = 0.45)						
**5.5.10 Diarrhea**						
Frenck 2021	8	1131	7	1129	2.4%	1.14 [0.42, 3.14]
Walter 2021	6	1511	4	749	1.8%	0.74 [0.21, 2.63]
**Subtotal (95% CI)**		**2642**		**1878**	**4.1%**	**0.97 [0.44, 2.12]**
Total events	14		11			
Heterogeneity: Chi² = 0.27, df = 1 (P = 0.60); I² = 0%						
Test for overall effect: Z = 0.08 (P = 0.94)						
**5.5.11 Chills**						
Frenck 2021	28	1131	10	1129	3.4%	2.80 [1.36, 5.73]
Walter 2021	5	1511	5	749	2.2%	0.50 [0.14, 1.71]
**Subtotal (95% CI)**		**2642**		**1878**	**5.6%**	**1.87 [1.05, 3.36]**
Total events	33		15			
Heterogeneity: Chi² = 5.64, df = 1 (P = 0.02); I² = 82%						
Test for overall effect: Z = 2.11 (P = 0.03)						
**Total (95% CI)**		**29062**		**20658**	**100.0%**	**1.40 [1.21, 1.62]**
Total events	480		258			
Heterogeneity: Chi² = 72.18, df = 21 (P < 0.00001); I² = 71%						
Test for overall effect: Z = 4.57 (P < 0.00001)						
Test for subgroup differences: Chi² = 24.10, df = 10 (P = 0.007), I² = 58.5%						

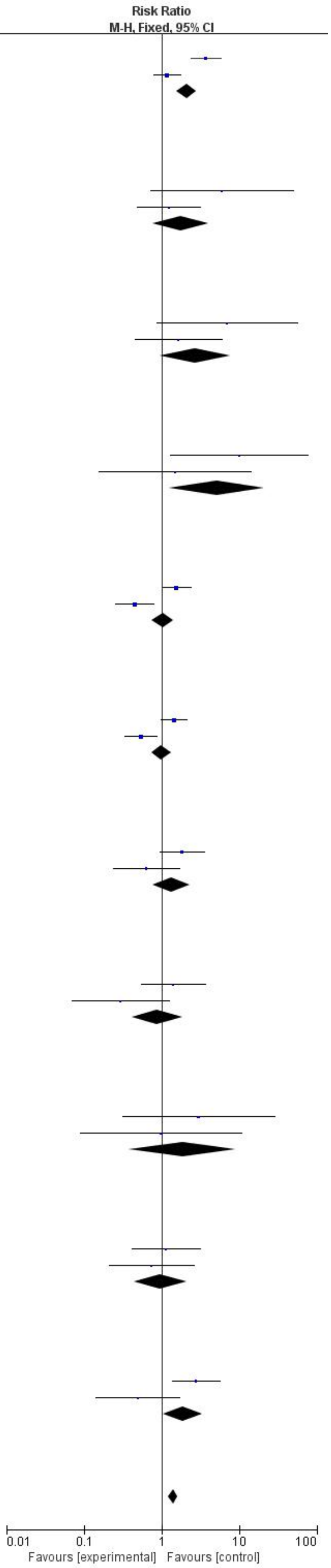

B) After dose 2

Study or Subgroup	12 - 15 years old Events	12 - 15 years old Total	5 - 11 years old Events	5 - 11 years old Total	Weight	Risk Ratio M-H, Fixed, 95% CI
**5.7.1 Local pain**						
Frenck 2021	79	1131	18	1129	7.7%	4.38 [2.64, 7.26]
Walter 2021	71	1501	29	741	16.6%	1.21 [0.79, 1.84]
**Subtotal (95% CI)**		**2632**		**1870**	**24.3%**	**2.21 [1.62, 3.02]**
Total events	150		47			
Heterogeneity: Chi² = 14.88, df = 1 (P = 0.0001); I² = 93%						
Test for overall effect: Z = 5.02 (P < 0.00001)						
**5.7.2 Erythema or redness**						
Frenck 2021	5	1131	1	1129	0.4%	4.99 [0.58, 42.65]
Walter 2021	19	1501	5	741	2.9%	1.88 [0.70, 5.00]
**Subtotal (95% CI)**		**2632**		**1870**	**3.3%**	**2.28 [0.95, 5.48]**
Total events	24		6			
Heterogeneity: Chi² = 0.66, df = 1 (P = 0.42); I² = 0%						
Test for overall effect: Z = 1.84 (P = 0.07)						
**5.7.3 Swelling**						
Frenck 2021	5	1131	1	1129	0.4%	4.99 [0.58, 42.65]
Walter 2021	15	1501	3	741	1.7%	2.47 [0.72, 8.50]
**Subtotal (95% CI)**		**2632**		**1870**	**2.1%**	**2.97 [1.03, 8.57]**
Total events	20		4			
Heterogeneity: Chi² = 0.31, df = 1 (P = 0.58); I² = 0%						
Test for overall effect: Z = 2.02 (P = 0.04)						
**5.7.4 Fever**						
Frenck 2021	20	1131	1	1129	0.4%	19.96 [2.68, 148.51]
Walter 2021	7	1501	1	741	0.6%	3.46 [0.43, 28.04]
**Subtotal (95% CI)**		**2632**		**1870**	**1.0%**	**10.52 [2.68, 41.29]**
Total events	27		2			
Heterogeneity: Chi² = 1.48, df = 1 (P = 0.22); I² = 32%						
Test for overall effect: Z = 3.37 (P = 0.0007)						
**5.7.5 Headache**						
Frenck 2021	65	1131	24	1129	10.3%	2.70 [1.71, 4.29]
Walter 2021	28	1501	19	741	10.9%	0.73 [0.41, 1.29]
**Subtotal (95% CI)**		**2632**		**1870**	**21.1%**	**1.69 [1.20, 2.38]**
Total events	93		43			
Heterogeneity: Chi² = 12.22, df = 1 (P = 0.0005); I² = 92%						
Test for overall effect: Z = 2.98 (P = 0.003)						
**5.7.6 Fatigue**						
Frenck 2021	66	1131	25	1129	10.7%	2.64 [1.68, 4.14]
Walter 2021	39	1501	24	741	13.7%	0.80 [0.49, 1.32]
**Subtotal (95% CI)**		**2632**		**1870**	**24.4%**	**1.60 [1.16, 2.22]**
Total events	105		49			
Heterogeneity: Chi² = 11.97, df = 1 (P = 0.0005); I² = 92%						
Test for overall effect: Z = 2.87 (P = 0.004)						
**5.7.7 Myalgia**						
Frenck 2021	32	1131	8	1129	3.4%	3.99 [1.85, 8.63]
Walter 2021	12	1501	7	741	4.0%	0.85 [0.33, 2.14]
**Subtotal (95% CI)**		**2632**		**1870**	**7.4%**	**2.30 [1.31, 4.01]**
Total events	44		15			
Heterogeneity: Chi² = 6.43, df = 1 (P = 0.01); I² = 84%						
Test for overall effect: Z = 2.92 (P = 0.004)						
**5.7.8 Arthralgia**						
Frenck 2021	16	1131	5	1129	2.1%	3.19 [1.17, 8.69]
Walter 2021	5	1501	4	741	2.3%	0.62 [0.17, 2.29]
**Subtotal (95% CI)**		**2632**		**1870**	**4.4%**	**1.86 [0.88, 3.92]**
Total events	21		9			
Heterogeneity: Chi² = 3.84, df = 1 (P = 0.05); I² = 74%						
Test for overall effect: Z = 1.64 (P = 0.10)						
**5.7.9 Vomiting**						
Frenck 2021	3	1131	1	1129	0.4%	2.99 [0.31, 28.75]
Walter 2021	2	1501	1	741	0.6%	0.99 [0.09, 10.87]
**Subtotal (95% CI)**		**2632**		**1870**	**1.0%**	**1.85 [0.38, 9.05]**
Total events	5		2			
Heterogeneity: Chi² = 0.44, df = 1 (P = 0.51); I² = 0%						
Test for overall effect: Z = 0.76 (P = 0.45)						
**5.7.10 Diarrhea**						
Frenck 2021	6	1131	4	1129	1.7%	1.50 [0.42, 5.29]
Walter 2021	5	1501	5	156	3.9%	0.10 [0.03, 0.36]
**Subtotal (95% CI)**		**2632**		**1285**	**5.6%**	**0.53 [0.25, 1.13]**
Total events	11		9			
Heterogeneity: Chi² = 9.36, df = 1 (P = 0.002); I² = 89%						
Test for overall effect: Z = 1.65 (P = 0.10)						
**5.7.11 Chills**						
Frenck 2021	42	1131	7	1129	3.0%	5.99 [2.70, 13.27]
Walter 2021	10	1501	4	741	2.3%	1.23 [0.39, 3.92]
**Subtotal (95% CI)**		**2632**		**1870**	**5.3%**	**3.93 [2.11, 7.33]**
Total events	52		11			
Heterogeneity: Chi² = 4.93, df = 1 (P = 0.03); I² = 80%						
Test for overall effect: Z = 4.30 (P < 0.0001)						
**Total (95% CI)**		**28952**		**19985**	**100.0%**	**2.04 [1.75, 2.38]**
Total events	552		197			
Heterogeneity: Chi² = 90.99, df = 21 (P < 0.00001); I² = 77%						
Test for overall effect: Z = 9.06 (P < 0.00001)						
Test for subgroup differences: Chi² = 25.99, df = 10 (P = 0.004), I² = 61.5%						

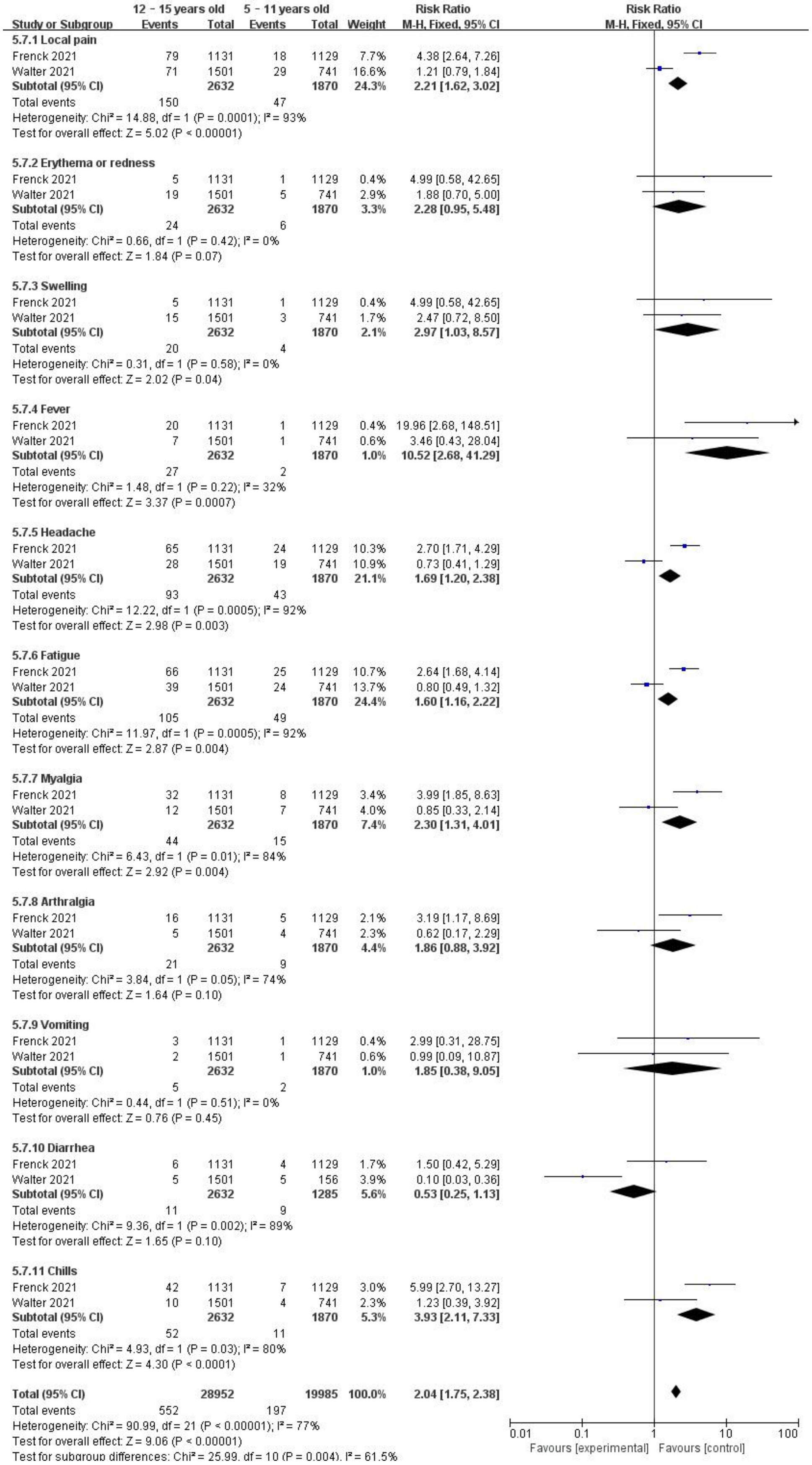

Supplementary Figure 10. Overall adverse reactions within 28 days after whole vaccination procedure in inactivated vaccine group of different ages versus control group

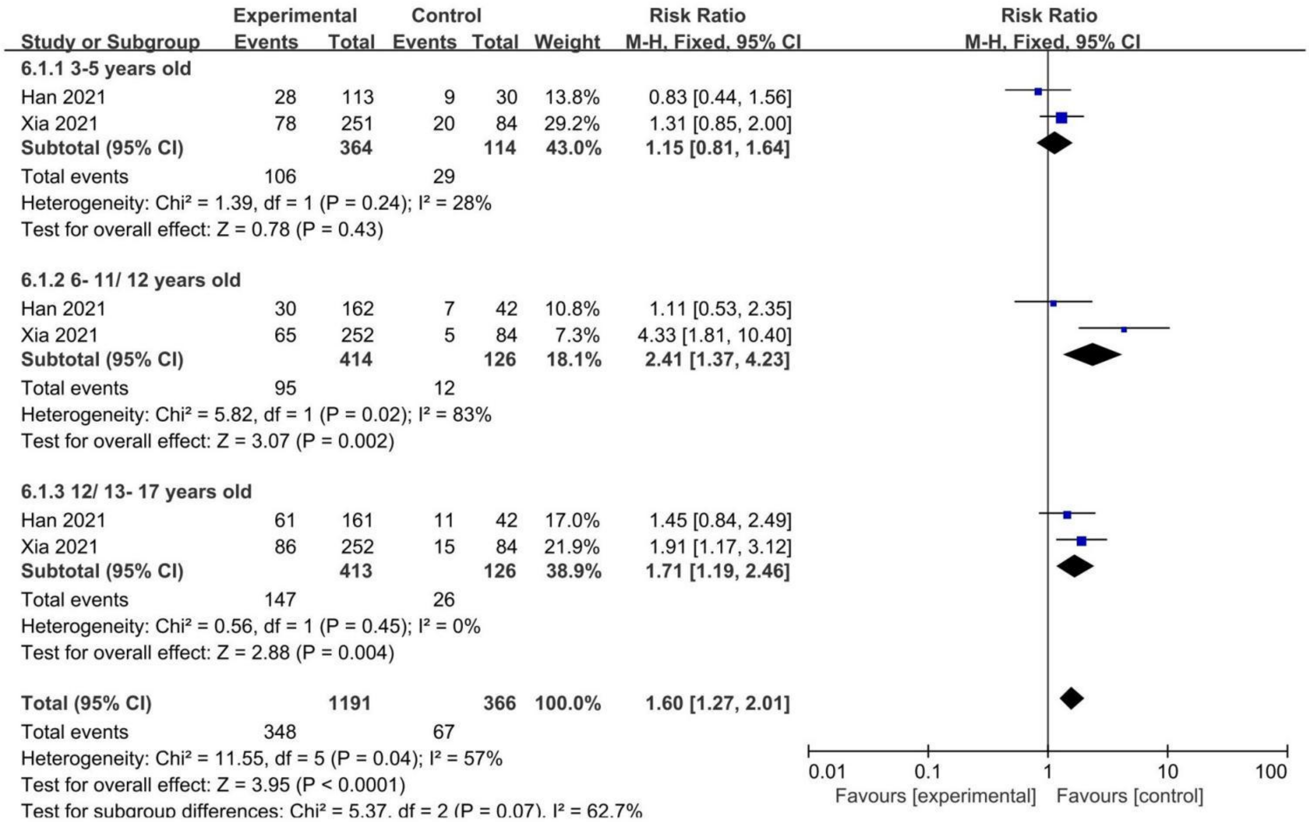

Study or Subgroup	Experimental Events	Experimental Total	Control Events	Control Total	Weight	Risk Ratio M-H, Fixed, 95% CI
**6.1.1 3-5 years old**						
Han 2021	28	113	9	30	13.8%	0.83 [0.44, 1.56]
Xia 2021	78	251	20	84	29.2%	1.31 [0.85, 2.00]
**Subtotal (95% CI)**		**364**		**114**	**43.0%**	**1.15 [0.81, 1.64]**
Total events	106		29			
Heterogeneity: Chi² = 1.39, df = 1 (P = 0.24); I² = 28%						
Test for overall effect: Z = 0.78 (P = 0.43)						
**6.1.2 6- 11/ 12 years old**						
Han 2021	30	162	7	42	10.8%	1.11 [0.53, 2.35]
Xia 2021	65	252	5	84	7.3%	4.33 [1.81, 10.40]
**Subtotal (95% CI)**		**414**		**126**	**18.1%**	**2.41 [1.37, 4.23]**
Total events	95		12			
Heterogeneity: Chi² = 5.82, df = 1 (P = 0.02); I² = 83%						
Test for overall effect: Z = 3.07 (P = 0.002)						
**6.1.3 12/ 13- 17 years old**						
Han 2021	61	161	11	42	17.0%	1.45 [0.84, 2.49]
Xia 2021	86	252	15	84	21.9%	1.91 [1.17, 3.12]
**Subtotal (95% CI)**		**413**		**126**	**38.9%**	**1.71 [1.19, 2.46]**
Total events	147		26			
Heterogeneity: Chi² = 0.56, df = 1 (P = 0.45); I² = 0%						
Test for overall effect: Z = 2.88 (P = 0.004)						
**Total (95% CI)**		**1191**		**366**	**100.0%**	**1.60 [1.27, 2.01]**
Total events	348		67			
Heterogeneity: Chi² = 11.55, df = 5 (P = 0.04); I² = 57%						
Test for overall effect: Z = 3.95 (P < 0.0001)						
Test for subgroup differences: Chi² = 5.37, df = 2 (P = 0.07), I² = 62.7%						

Supplementary Figure 11. Seroconversion rate in vaccine group versus control group: A) Pseudovirus neutralizing antibody; B) Neutralizing antibody 28 days after Dose 2; C) RBD–binding enzyme-linked immunosorbent assay antibody

A) Pseudovirus neutralizing antibody

Study or Subgroup	Experimental Events	Experimental Total	Control Events	Control Total	Weight	Risk Ratio M-H, Fixed, 95% CI
**1.4.2 28 days after Dose 1**						
Han 2021	37	53	0	16	7.8%	23.61 [1.53, 364.38]
Xia 2021	705	745	0	248	7.7%	470.96 [29.54, 7509.00]
Zhu 2021	88	100	3	50	41.2%	14.67 [4.88, 44.04]
**Subtotal (95% CI)**		**898**		**314**	**56.7%**	**77.99 [28.40, 214.14]**
Total events	830		3			
Heterogeneity: Chi² = 11.22, df = 2 (P = 0.004); I² = 82%						
Test for overall effect: Z = 8.45 (P < 0.00001)						
**1.4.3 28 days after Dose 2**						
Han 2021	413	419	0	110	8.1%	218.56 [13.76, 3472.62]
Xia 2021	749	749	0	246	7.7%	493.67 [30.97, 7870.45]
Zhu 2021	98	100	2	50	27.4%	24.50 [6.30, 95.28]
**Subtotal (95% CI)**		**1268**		**406**	**43.3%**	**144.80 [44.97, 466.24]**
Total events	1260		2			
Heterogeneity: Chi² = 7.41, df = 2 (P = 0.02); I² = 73%						
Test for overall effect: Z = 8.34 (P < 0.00001)						
**Total (95% CI)**		**2166**		**720**	**100.0%**	**106.92 [49.71, 229.97]**
Total events	2090		5			
Heterogeneity: Chi² = 20.76, df = 5 (P = 0.0009); I² = 76%						
Test for overall effect: Z = 11.96 (P < 0.00001)						
Test for subgroup differences: Chi² = 0.62, df = 1 (P = 0.43), I² = 0%						

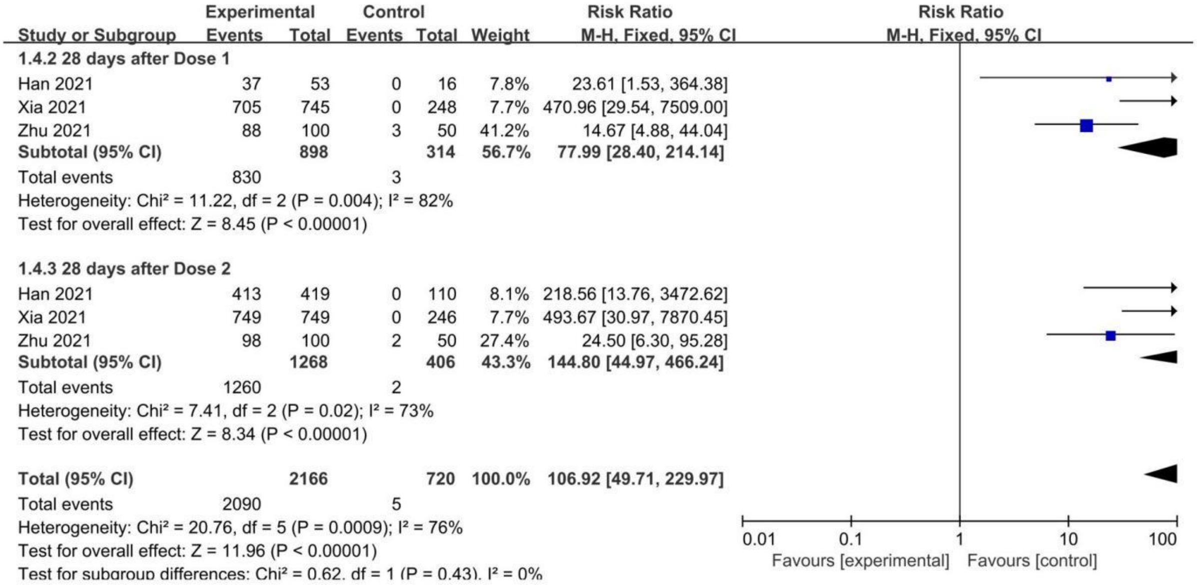

B) Neutralizing antibody 28 days after Dose 2

Study or Subgroup	Experimental Events	Experimental Total	Control Events	Control Total	Weight	Risk Ratio M-H, Fixed, 95% CI
**1.5.1 3-5 years old**						
Han 2021	109	109	0	29	17.0%	59.73 [3.82, 933.02]
Xia 2021	248	248	0	81	16.3%	163.67 [10.32, 2594.65]
**Subtotal (95% CI)**		**357**		**110**	**33.3%**	**110.57 [15.87, 770.57]**
Total events	357		0			
Heterogeneity: Chi² = 0.27, df = 1 (P = 0.60); I² = 0%						
Test for overall effect: Z = 4.75 (P < 0.00001)						
**1.5.2 6- 11/ 12 years old**						
Han 2021	154	155	0	41	17.1%	83.19 [5.29, 1308.27]
Xia 2021	250	250	0	83	16.2%	167.67 [10.57, 2658.51]
**Subtotal (95% CI)**		**405**		**124**	**33.3%**	**124.37 [17.79, 869.21]**
Total events	404		0			
Heterogeneity: Chi² = 0.13, df = 1 (P = 0.72); I² = 0%						
Test for overall effect: Z = 4.86 (P < 0.00001)						
**1.5.3 12/ 13- 17 years old**						
Han 2021	150	155	0	40	17.1%	79.11 [5.03, 1243.70]
Xia 2021	251	251	0	82	16.3%	165.67 [10.45, 2626.62]
**Subtotal (95% CI)**		**406**		**122**	**33.4%**	**121.28 [17.36, 847.06]**
Total events	401		0			
Heterogeneity: Chi² = 0.14, df = 1 (P = 0.71); I² = 0%						
Test for overall effect: Z = 4.84 (P < 0.00001)						
**Total (95% CI)**		**1168**		**356**	**100.0%**	**118.74 [38.67, 364.63]**
Total events	1162		0			
Heterogeneity: Chi² = 0.56, df = 5 (P = 0.99); I² = 0%						
Test for overall effect: Z = 8.35 (P < 0.00001)						
Test for subgroup differences: Chi² = 0.01, df = 2 (P = 1.00), I² = 0%						

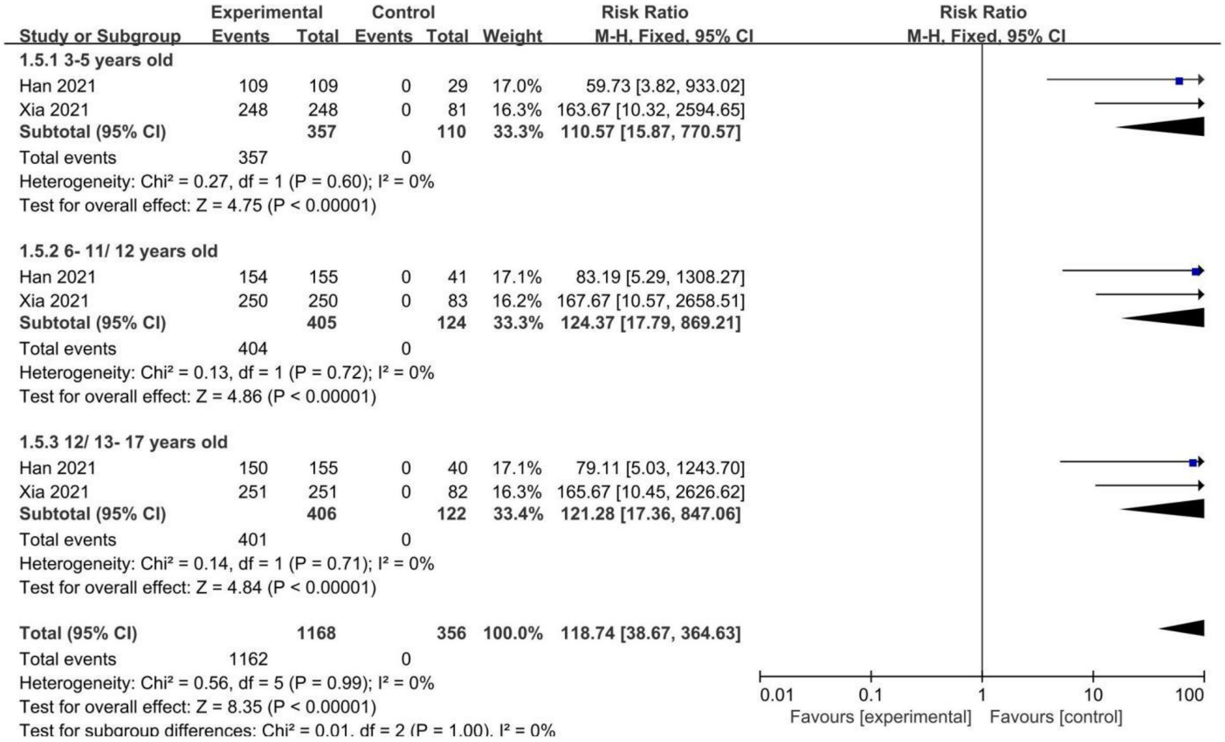

C) RBD–binding enzyme-linked immunosorbent assay antibody

Study or Subgroup	Experimental Events	Experimental Total	Control Events	Control Total	Weight	Risk Ratio M-H, Fixed, 95% CI
**1.6.1 28 days after Dose 1**						
Zhu 2021	98	100	0	50	33.3%	99.48 [6.31, 1569.12]
**Subtotal (95% CI)**		**100**		**50**	**33.3%**	**99.48 [6.31, 1569.12]**
Total events	98		0			
Heterogeneity: Not applicable						
Test for overall effect: Z = 3.27 (P = 0.001)						
**1.6.2 56 days after Dose 1 (Bfter Dose 2)**						
Zhu 2021	97	100	0	50	33.3%	98.47 [6.24, 1553.30]
**Subtotal (95% CI)**		**100**		**50**	**33.3%**	**98.47 [6.24, 1553.30]**
Total events	97		0			
Heterogeneity: Not applicable						
Test for overall effect: Z = 3.26 (P = 0.001)						
**1.6.3 28 days after Dose 2**						
Zhu 2021	100	100	0	50	33.3%	101.50 [6.44, 1600.76]
**Subtotal (95% CI)**		**100**		**50**	**33.3%**	**101.50 [6.44, 1600.76]**
Total events	100		0			
Heterogeneity: Not applicable						
Test for overall effect: Z = 3.28 (P = 0.001)						
**Total (95% CI)**		**300**		**150**	**100.0%**	**99.81 [20.30, 490.69]**
Total events	295		0			
Heterogeneity: Chi² = 0.00, df = 2 (P = 1.00); I² = 0%						
Test for overall effect: Z = 5.67 (P < 0.00001)						
Test for subgroup differences: Chi² = 0.00, df = 2 (P = 1.00), I² = 0%						

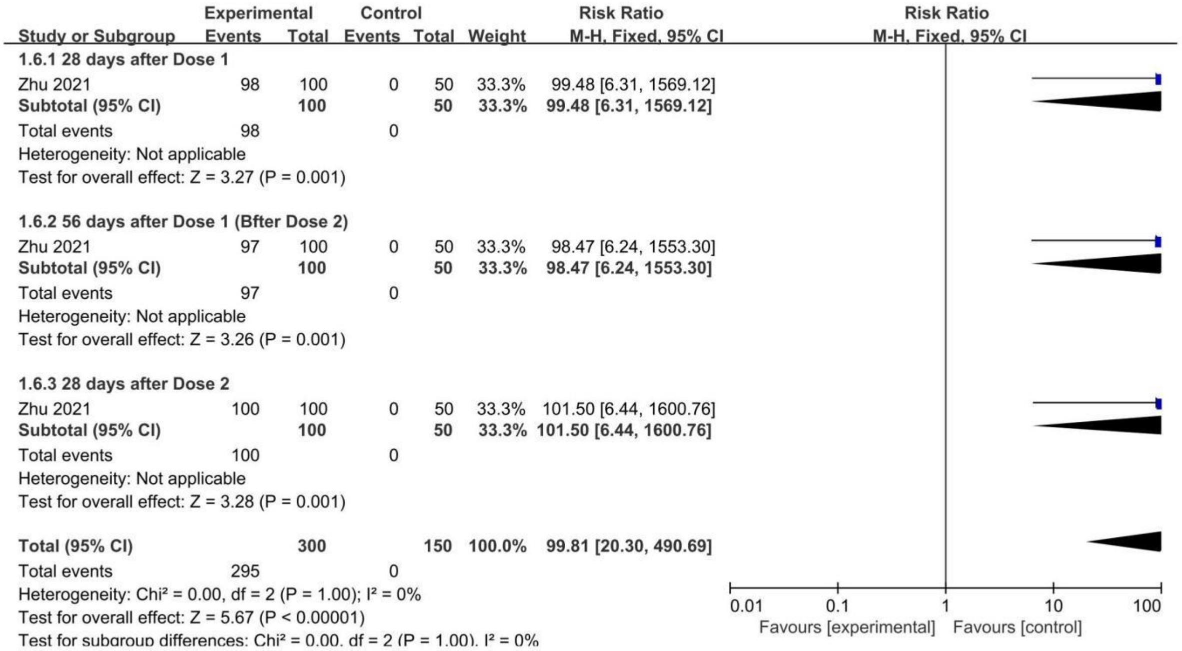

Supplementary Figure 12. COVID-19 diagnosed after vaccination in vaccine group versus control group:

A) Covid-19 after the vaccination

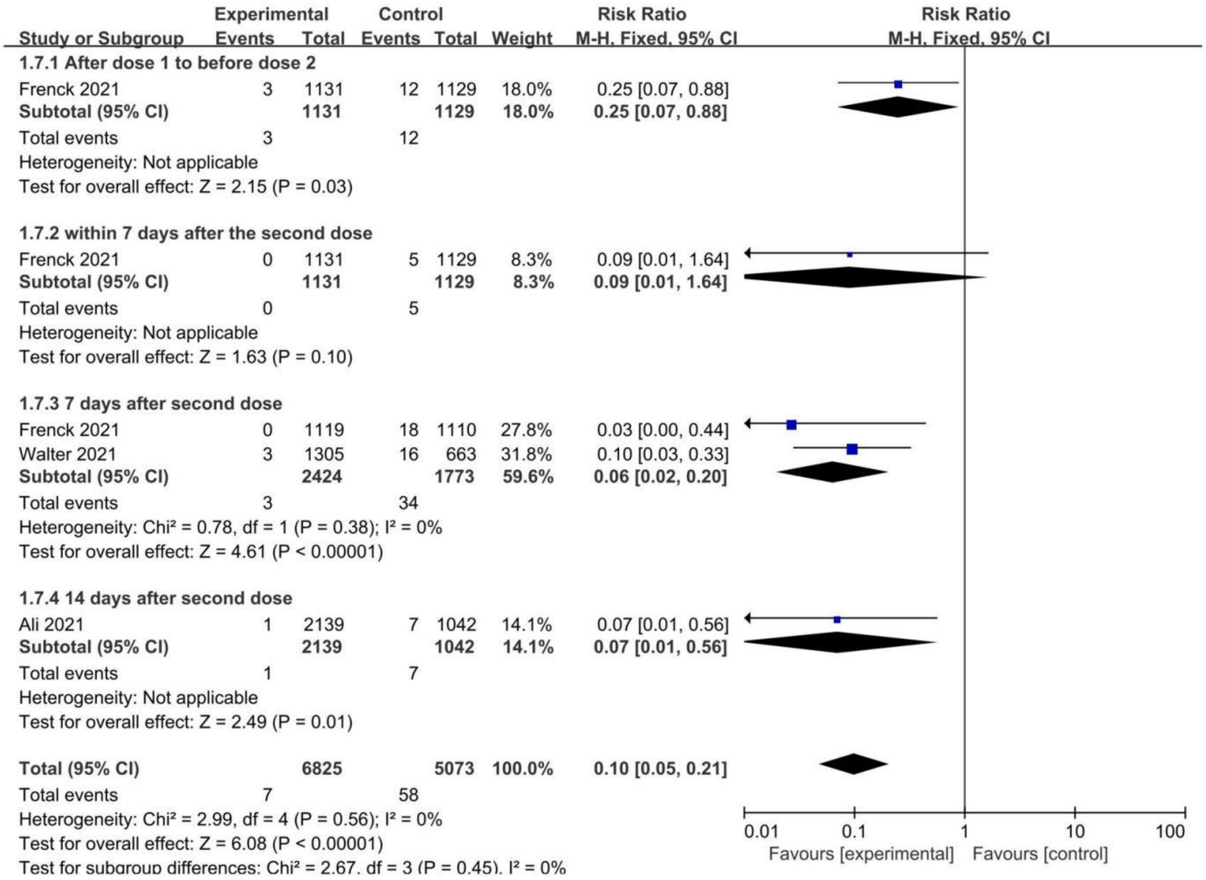

B) Covid-19 after dose 2

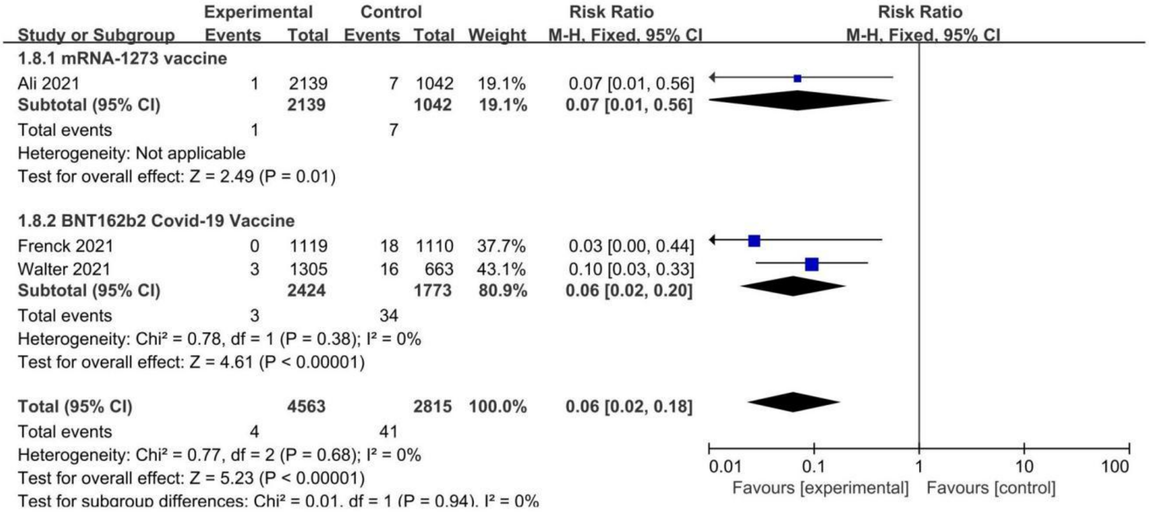

Supplement: Supplementary file 1 [file Data_Sheet_1.ZIP › Supplementary Material/Supplementary Figures (total).pdf]
