# Supplementary material for: Safety, Immunogenicity, and Efficacy of COVID-19 Vaccines in Adolescents, Children, and Infants: A Systematic Review and Meta-Analysis
Source: Front Public Health. 2022 Apr 14;10:829176. doi: 10.3389/fpubh.2022.829176 (PMC9046659; doi:10.3389/fpubh.2022.829176)
Supplement: Supplementary file 1 [file Data_Sheet_1.ZIP › Supplementary Material/Supplementary Tables.docx]

Supplementary Material

**Supplementary Table 1. Specific adverse reactions among vaccination group versus control group after dose 1 and dose 2**

|  |  | **No. of studies** | **RR (95% CI)** | **I2** | **P** |
| --- | --- | --- | --- | --- | --- |
| **After dose 1** | | | | | |
| **mRNA vaccine** | Overall | 3 | 1.81 [1.74, 1.88] | 94 | <0.05* |
|  | Local pain | 3 | 2.61 [2.43, 2.82] | 88 | <0.05* |
|  | Erythema or Redness | 3 | 12.09 [7.21, 20.28] | 92 | <0.05* |
|  | Swelling | 3 | 13.41 [8.15, 22.06] | 82 | <0.05* |
|  | Axillary Swelling | 1 | 2.85 [2.34, 3.49] | Not applicable | <0.05* |
|  | Fever | 3 | 2.94 [1.68, 5.14] | 0 | <0.05* |
|  | Headache | 3 | 1.15 [1.06, 1.24] | 84 | <0.05* |
|  | Fatigue | 3 | 1.27 [1.17, 1.38] | 85 | <0.05* |
|  | Myalgia | 3 | 1.60 [1.40, 1.84] | 44 | <0.05* |
|  | Arthralgia | 3 | 1.27 [1.06, 1.51] | 51 | <0.05* |
|  | Nausea/ vomiting | 1 | 1.28 [1.03, 1.57] | Not applicable | <0.05* |
|  | Vomiting | 2 | 1.85 [0.38, 9.07] | 0 | >0.05 |
|  | Diarrhea | 2 | 0.97 [0.44, 2.12] | 0 | >0.05 |
|  | Chills | 3 | 1.67 [1.41, 1.98] | 65 | <0.05* |
| **Inactivated vaccine** | Overall | 2 | 1.40 [1.04, 1.90] | 0 | <0.05* |
|  | Local pain | 2 | 21.53 [3.00, 154.35] | 0 | <0.05* |
|  | Redness | 1 | 1.34 [0.15, 11.89] | Not applicable | >0.05 |
|  | Induration | 2 | 1.16 [0.13, 10.46] | 0 | >0.05 |
|  | Pruritus/ Itch | 2 | 1.70 [0.20, 14.35] | 0 | >0.05 |
|  | Swelling | 2 | 0.50 [0.10, 2.42] | 22 | >0.05 |
|  | Fever | 2 | 1.54 [0.84, 2.82] | 0 | >0.05 |
|  | Cough | 2 | 0.73 [0.36, 1.45] | 0 | >0.05 |
|  | Headache | 2 | 1.04 [0.33, 3.32] | 48 | >0.05 |
|  | Fatigue | 2 | 1.11 [0.19, 6.54] | 0 | >0.05 |
|  | Myalgia | 2 | 2.09 [0.26, 16.84] | 0 | >0.05 |
|  | Nausea | 2 | 0.74 [0.18, 3.13] | 0 | >0.05 |
|  | Vomiting | 2 | 1.55 [0.27, 8.88] | 0 | >0.05 |
|  | Diarrhea | 2 | 0.46 [0.17, 1.27] | 0 | >0.05 |
|  | Constipation | 1 | 1.00 [0.04, 24.57] | Not applicable | >0.05 |
|  | Anorexia | 1 | 0.52 [0.10, 2.82] | Not applicable | >0.05 |
|  | Acute allergic reaction | 1 | 0.11 [0.00, 2.73] | Not applicable | >0.05 |
|  | Abnormal skin and mucosa | 1 | 1.84 [0.10, 35.41] | Not applicable | >0.05 |
|  | Dysphagia | 1 | 1.00 [0.04, 24.57] | Not applicable | >0.05 |
| **Vectored vaccine** | Overall | 1 | 3.87 [2.36, 6.34] | 0 | <0.05* |
|  | Local pain | 1 | 5.67 [1.83, 17.55] | Not applicable | <0.05* |
|  | Erythema or Redness | 1 | 2.52 [0.12, 51.61] | Not applicable | >0.05 |
|  | Induration | 1 | 2.52 [0.12, 51.61] | Not applicable | >0.05 |
|  | Itch | 1 | 2.52 [0.12, 51.61] | Not applicable | >0.05 |
|  | Swelling | 1 | 4.54 [0.25, 82.78] | Not applicable | >0.05 |
|  | Fever | 1 | 7.00 [1.74, 28.21] | Not applicable | <0.05* |
|  | Cough | 1 | 1.00 [0.26, 3.83] | Not applicable | >0.05 |
|  | Oropharyngeal pain | 1 | 1.50 [0.16, 14.06] | Not applicable | >0.05 |
|  | Headache | 1 | 14.64 [0.89, 240.57] | Not applicable | >0.05 |
|  | Fatigue | 1 | 2.25 [0.51, 10.02] | Not applicable | >0.05 |
|  | Myalgia | 1 | 4.54 [0.25, 82.78] | Not applicable | >0.05 |
|  | Arthralgia | 1 | 1.51 [0.06, 36.53] | Not applicable | >0.05 |
|  | Nausea | 1 | 4.54 [0.25, 82.78] | Not applicable | >0.05 |
|  | Vomiting | 1 | 2.52 [0.12, 51.61] | Not applicable | >0.05 |
|  | Diarrhea | 1 | 2.52 [0.12, 51.61] | Not applicable | >0.05 |
|  | Anorexia | 1 | 7.57 [0.44, 130.01] | Not applicable | >0.05 |
|  | Pruritus (systemic adverse reaction) | 1 | 1.51 [0.06, 36.53] | Not applicable | >0.05 |
|  | Dyspnoea | 1 | Not estimable | Not applicable | Not applicable |
| **After dose 2** | | | | | |
| **mRNA vaccine** | Overall | 3 | 3.16 [3.03, 3.30] | 91 | <0.05* |
|  | Local pain | 3 | 2.96 [2.73, 3.22] | 90 | <0.05* |
|  | Erythema or Redness | 3 | 15.02 [9.26, 24.34] | 90 | <0.05* |
|  | Swelling | 3 | 16.62 [10.11, 27.33] | 82 | <0.05* |
|  | Axillary Swelling | 1 | 4.19 [3.24, 5.41] | Not applicable | <0.05* |
|  | Fever | 3 | 12.16 [7.15, 20.66] | 0 | <0.05* |
|  | Headache | 3 | 2.26 [2.07, 2.46] | 87 | <0.05* |
|  | Fatigue | 3 | 2.26 [2.07, 2.47] | 89 | <0.05* |
|  | Myalgia | 3 | 3.60 [3.11, 4.18] | 79 | <0.05* |
|  | Arthralgia | 3 | 3.04 [2.54, 3.64] | 65 | <0.05* |
|  | Nausea/ vomiting | 1 | 2.75 [2.26, 3.34] | Not applicable | <0.05* |
|  | Vomiting | 2 | 1.85 [0.38, 9.05] | 0 | >0.05 |
|  | Diarrhea | 2 | 0.53 [0.25, 1.13] | 89 | >0.05 |
|  | Chills | 3 | 5.28 [4.38, 6.37] | 68 | <0.05* |
| **Inactivated vaccine** | Overall | 2 | 1.84 [1.20, 2.81] | 0 | <0.05* |
|  | Local pain | 2 | 6.84 [1.96, 23.90] | 0 | <0.05* |
|  | Erythema or Redness | 2 | 1.21 [0.30, 4.83] | 0 | >0.05 |
|  | Pruritus/ Itch | 2 | 1.16 [0.13, 10.41] | 0 | >0.05 |
|  | Swelling | 2 | 2.24 [0.28, 18.17] | 0 | >0.05 |
|  | Fever | 2 | 1.64 [0.64, 4.24] | 0 | >0.05 |
|  | Cough | 2 | 2.12 [0.49, 9.19] | 0 | >0.05 |
|  | Headache | 2 | 1.21 [0.20, 7.38] | 16 | >0.05 |
|  | Fatigue | 2 | 0.79 [0.03, 19.23] | Not applicable | >0.05 |
|  | Myalgia | 2 | 0.89 [0.09, 8.49] | 0 | >0.05 |
|  | Nausea | 2 | 1.75 [0.21, 14.54] | 0 | >0.05 |
|  | Vomiting | 2 | 0.89 [0.14, 5.49] | 0 | >0.05 |
|  | Diarrhea | 1 | 0.26 [0.02, 4.14] | Not applicable | >0.05 |
|  | Anorexia | 1 | 2.37 [0.13, 43.62] | Not applicable | >0.05 |
|  | Acute allergic reaction/ Hypersensitivity | 2 | 0.31 [0.05, 2.04] | 10 | >0.05 |
|  | Abnormal skin and mucosa | 1 | 0.26 [0.02, 4.14] | Not applicable | >0.05 |
|  | Dysphagia | 1 | 0.99 [0.04, 24.31] | Not applicable | >0.05 |
| **Vectored vaccine** | Overall | 1 | 4.03 [2.03, 8.01] | 0 | <0.05* |
|  | Local pain | 1 | 14.64 [0.89, 240.57] | Not applicable | >0.05 |
|  | Erythema or Redness | 1 | Not estimable | Not applicable | Not applicable |
|  | Induration | 1 | 1.51 [0.06, 36.53] | Not applicable | >0.05 |
|  | Itch | 1 | 3.53 [0.19, 67.13] | Not applicable | >0.05 |
|  | Swelling | 1 | 4.54 [0.25, 82.78] | Not applicable | >0.05 |
|  | Fever | 1 | 6.50 [0.87, 48.29] | Not applicable | >0.05 |
|  | Cough | 1 | 2.00 [0.23, 17.43] | Not applicable | >0.05 |
|  | Oropharyngeal pain | 1 | 1.51 [0.06, 36.53] | Not applicable | >0.05 |
|  | Headache | 1 | 8.58 [0.51, 145.79] | Not applicable | >0.05 |
|  | Fatigue | 1 | 6.56 [0.38, 114.24] | Not applicable | >0.05 |
|  | Myalgia | 1 | 1.51 [0.06, 36.53] | Not applicable | >0.05 |
|  | Arthralgia | 1 | 1.51 [0.06, 36.53] | Not applicable | >0.05 |
|  | Nausea | 1 | 3.53 [0.19, 67.13] | Not applicable | >0.05 |
|  | Vomiting | 1 | 2.52 [0.12, 51.61] | Not applicable | >0.05 |
|  | Diarrhea | 1 | 1.51 [0.06, 36.53] | Not applicable | >0.05 |
|  | Anorexia | 1 | 2.52 [0.12, 51.61] | Not applicable | >0.05 |
|  | Pruritus (systemic adverse reaction) | 1 | Not estimable | Not applicable | Not applicable |
|  | Dyspnoea | 1 | 1.51 [0.06, 36.53] | Not applicable | >0.05 |

* P <0.05

**Supplementary Table 2. Adverse reactions in mRNA vaccine group of different ages versus control group**

|  | No. of studies | RR (95% CI) | I2 | P |
| --- | --- | --- | --- | --- |
| Local pain after the first vaccination | 3 | 2.61 [2.43, 2.82] | 88 | <0.05* |
| ≥ 12 years old | 2 | 2.71 [2.51, 2.93] | 51 | <0.05* |
| ＜12 years old | 1 | 1.18 [0.79, 1.78] | Not applicable | >0.05 |
| Local pain after the second vaccination | 3 | 2.96 [2.73, 3.22] | 90 | <0.05* |
| ≥ 12 years old | 2 | 3.09 [2.84, 3.37] | 49 | <0.05* |
| ＜12 years old | 1 | 1.21 [0.79, 1.84] | Not applicable | >0.05 |
| Erythema or Redness after the first vaccination | 3 | 12.09 [7.21, 20.28] | 92 | <0.05* |
| ≥ 12 years old | 2 | 19.55 [10.04, 38.06] | 19 | <0.05* |
| ＜12 years old | 1 | 1.24 [0.48, 3.18] | Not applicable | >0.05 |
| Erythema or Redness after the second vaccination | 3 | 15.02 [9.26, 24.34] | 90 | <0.05* |
| ≥ 12 years old | 2 | 20.60 [11.60, 36.58] | 41 | <0.05* |
| ＜12 years old | 1 | 1.88 [0.70, 5.00] | Not applicable | >0.05 |
| Swelling after the first vaccination | 3 | 13.41 [8.15, 22.06] | 82 | <0.05* |
| ≥ 12 years old | 2 | 16.18 [9.31, 28.09] | 0 | <0.05* |
| ＜12 years old | 1 | 1.65 [0.46, 5.99] | Not applicable | >0.05 |
| Swelling after the second vaccination | 3 | 16.62 [10.11, 27.33] | 82 | <0.05* |
| ≥ 12 years old | 2 | 19.95 [11.50, 34.61] | 39 | <0.05* |
| ＜12 years old | 1 | 2.47 [0.72, 8.50] | Not applicable | >0.05 |
| Fever after the first vaccination | 3 | 2.94 [1.68, 5.14] | 0 | <0.05* |
| ≥ 12 years old | 2 | 3.05 [1.71, 5.44] | 34 | <0.05* |
| ＜12 years old | 1 | 1.49 [0.15, 14.27] | Not applicable | >0.05 |
| Fever after the second vaccination | 3 | 12.16 [7.15, 20.66] | 0 | <0.05* |
| ≥ 12 years old | 2 | 12.84 [7.41, 22.25] | 0 | <0.05* |
| ＜12 years old | 1 | 3.46 [0.43, 28.04] | Not applicable | >0.05 |
| Headache after the first vaccination | 3 | 1.15 [1.06, 1.24] | 84 | <0.05* |
| ≥ 12 years old | 2 | 1.18 [1.09, 1.28] | 50 | <0.05* |
| ＜12 years old | 1 | 0.45 [0.26, 0.80] | Not applicable | <0.05* |
| Headache after the second vaccination | 3 | 2.26 [2.07, 2.46] | 87 | <0.05* |
| ≥ 12 years old | 2 | 2.33 [2.14, 2.55] | 0 | <0.05* |
| ＜12 years old | 1 | 0.73 [0.41, 1.29] | Not applicable | >0.05 |
| Fatigue after the first vaccination | 3 | 1.27 [1.17, 1.38] | 85 | <0.05* |
| ≥ 12 years old | 2 | 1.32 [1.21, 1.43] | 0 | <0.05* |
| ＜12 years old | 1 | 0.54 [0.34, 0.88] | Not applicable | <0.05* |
| Fatigue after the second vaccination | 3 | 2.26 [2.07, 2.47] | 89 | <0.05* |
| ≥ 12 years old | 2 | 2.36 [2.15, 2.58] | 0 | <0.05* |
| ＜12 years old | 1 | 0.80 [0.49, 1.32] | Not applicable | >0.05 |
| Myalgia after the first vaccination | 3 | 1.60 [1.40, 1.84] | 44 | <0.05* |
| ≥ 12 years old | 2 | 1.64 [1.43, 1.88] | 0 | <0.05* |
| ＜12 years old | 1 | 0.64 [0.24, 1.70] | Not applicable | >0.05 |
| Myalgia after the second vaccinatio | 3 | 3.60 [3.11, 4.18] | 79 | <0.05* |
| ≥ 12 years old | 2 | 3.73 [3.20, 4.33] | 0 | <0.05* |
| ＜12 years old | 1 | 0.85 [0.33, 2.14] | Not applicable | >0.05 |
| Arthralgia after the first vaccination | 3 | 1.27 [1.06, 1.51] | 51 | <0.05* |
| ≥ 12 years old | 2 | 1.30 [1.09, 1.55] | 0 | <0.05* |
| ＜12 years old | 1 | 0.30 [0.07, 1.24] | Not applicable | >0.05 |
| Arthralgia after the second vaccination | 3 | 3.04 [2.54, 3.64] | 65 | <0.05* |
| ≥ 12 years old | 2 | 3.12 [2.60, 3.75] | 0 | <0.05* |
| ＜12 years old | 1 | 0.62 [0.17, 2.29] | Not applicable | >0.05 |
| Vomiting after the first vaccination | 2 | 1.85 [0.38, 9.07] | 0 | >0.05 |
| ≥ 12 years old | 1 | 2.99 [0.31, 28.75] | Not applicable | >0.05 |
| ＜12 years old | 1 | 0.99 [0.09, 10.92] | Not applicable | >0.05 |
| Vomiting after the second vaccination | 2 | 1.85 [0.38, 9.05] | 0 | >0.05 |
| ≥ 12 years old | 1 | 2.99 [0.31, 28.75] | Not applicable | >0.05 |
| ＜12 years old | 1 | 0.99 [0.09, 10.87] | Not applicable | >0.05 |
| Diarrhea after the first vaccination | 2 | 0.97 [0.44, 2.12] | 0 | >0.05 |
| ≥ 12 years old | 1 | 1.14 [0.42, 3.14] | Not applicable | >0.05 |
| ＜12 years old | 1 | 0.74 [0.21, 2.63] | Not applicable | >0.05 |
| Diarrhea after the second vaccination | 2 | 0.53 [0.25, 1.13] | 89 | >0.05 |
| ≥ 12 years old | 1 | 1.50 [0.42, 5.29] | Not applicable | >0.05 |
| ＜12 years old | 1 | 0.10 [0.03, 0.36] | Not applicable | <0.05* |
| Chills after the first vaccination | 3 | 1.67 [1.41, 1.98] | 65 | <0.05* |
| ≥ 12 years old | 2 | 1.71 [1.44, 2.03] | 49 | <0.05* |
| ＜12 years old | 1 | 0.50 [0.14, 1.71] | Not applicable | >0.05 |
| Chills after the second vaccination | 3 | 5.28 [4.38, 6.37] | 68 | <0.05* |
| ≥ 12 years old | 2 | 5.44 [4.50, 6.58] | 0 | <0.05* |
| ＜12 years old | 1 | 1.23 [0.39, 3.92] | Not applicable | >0.05 |

* P <0.05

| **Supplementary Table 3. Summary of findings table for the safety of COVID-19 vaccines (Vaccine versus Placebo)** | | | | | | |  |
| --- | --- | --- | --- | --- | --- | --- | --- |
| **Patient or population:** adolescents, children, and infants **Intervention:** Vaccine versus Placebo | | | | | | |  |
| **Outcomes** | **Illustrative comparative risks* (95% CI)** | | **Relative effect (95% CI)** | **No of Participants (studies)** | **Quality of the evidence (GRADE)** | **Comments** |  |
|  | Assumed risk | Corresponding risk |  |  |  |  |  |
|  | **Control** | **Vaccine versus Placebo** |  |  |  |  |  |
| **Unsolicited adverse reactions within 28 or 30 days after whole vaccination procedure - Overall** | Study population | | RR 1.21  (1.07 to 1.36) | 8804 (4 studies) | ⊕⊕⊕⊕ high | / |  |
|  | 106 per 1000 | 128 per 1000 (113 to 144) |  |  |  |  |  |
|  | Moderate | |  |  |  |  |  |
|  | 86 per 1000 | 104 per 1000 (92 to 117) |  |  |  |  |  |
| **Unsolicited adverse reactions within 28 or 30 days after whole vaccination procedure - Related to study vaccination** | Study population | | RR 1.96  (1.59 to 2.41) | 8254 (3 studies) | ⊕⊕⊕⊕ high | / |  |
|  | 35 per 1000 | 68 per 1000 (56 to 84) |  |  |  |  |  |
|  | Moderate | |  |  |  |  |  |
|  | 21 per 1000 | 41 per 1000 (33 to 51) |  |  |  |  |  |
| **Unsolicited adverse reactions within 28 or 30 days after whole vaccination procedure - Severe** | Study population | | RR 2.35  (0.78 to 7.03) | 8254 (3 studies) | ⊕⊕⊕⊕ high^1^ | / |  |
|  | 1 per 1000 | 3 per 1000 (1 to 9) |  |  |  |  |  |
|  | Moderate | |  |  |  |  |  |
|  | 1 per 1000 | 2 per 1000 (1 to 7) |  |  |  |  |  |
| **Unsolicited adverse reactions within 28 or 30 days after whole vaccination procedure - Life-threatening** | Study population | | RR 1  (0.06 to 15.94) | 8254 (3 studies) | ⊕⊕⊕⊕ high | / |  |
|  | 0 per 1000 | 0 per 1000 (0 to 5) |  |  |  |  |  |
|  | Moderate | |  |  |  |  |  |
|  | 0 per 1000 | 0 per 1000 (0 to 0) |  |  |  |  |  |
| **Total adverse reactions in vaccination group versus control group** | Study population | | RR 1.85  (1.8 to 1.89) | 30242 (4 studies) | ⊕⊕⊕⊝ moderate^2^ | / |  |
|  | 391 per 1000 | 724 per 1000 (704 to 739) |  |  |  |  |  |
|  | Moderate | |  |  |  |  |  |
|  | 86 per 1000 | 159 per 1000 (155 to 163) |  |  |  |  |  |
| **Total adverse reactions in vaccination group versus control group - Solicited adverse reactions after the first vaccination** | Study population | | RR 1.49  (1.43 to 1.55) | 4420 (3 studies) | ⊕⊕⊕⊝ moderate^2^ | / |  |
|  | 591 per 1000 | 881 per 1000 (846 to 917) |  |  |  |  |  |
|  | Moderate | |  |  |  |  |  |
|  | 160 per 1000 | 238 per 1000 (229 to 248) |  |  |  |  |  |
| **Total adverse reactions in vaccination group versus control group - Solicited local adverse reactions after the first vaccination** | Study population | | RR 2.6  (2.42 to 2.8) | 4877 (3 studies) | ⊕⊕⊕⊕ high^1^ | / |  |
|  | 299 per 1000 | 777 per 1000 (723 to 836) |  |  |  |  |  |
|  | Moderate | |  |  |  |  |  |
|  | 60 per 1000 | 156 per 1000 (145 to 168) |  |  |  |  |  |
| **Total adverse reactions in vaccination group versus control group - Solicited systemic adverse reactions after the first vaccination** | Study population | | RR 1.26  (1.19 to 1.33) | 4877 (3 studies) | ⊕⊕⊕⊝ moderate^2^ | / |  |
|  | 466 per 1000 | 587 per 1000 (554 to 619) |  |  |  |  |  |
|  | Moderate | |  |  |  |  |  |
|  | 100 per 1000 | 126 per 1000 (119 to 133) |  |  |  |  |  |
| **Total adverse reactions in vaccination group versus control group - Solicited adverse reactions after the second vaccination** | Study population | | RR 1.76  (1.67 to 1.85) | 4384 (3 studies) | ⊕⊕⊕⊝ moderate^2^ | / |  |
|  | 500 per 1000 | 879 per 1000 (834 to 924) |  |  |  |  |  |
|  | Moderate | |  |  |  |  |  |
|  | 72 per 1000 | 127 per 1000 (120 to 133) |  |  |  |  |  |
| **Total adverse reactions in vaccination group versus control group - Solicited local adverse reactions after the second vaccination** | Study population | | RR 2.89  (2.67 to 3.14) | 4847 (3 studies) | ⊕⊕⊕⊕ high^1^ | / |  |
|  | 264 per 1000 | 762 per 1000 (704 to 827) |  |  |  |  |  |
|  | Moderate | |  |  |  |  |  |
|  | 8 per 1000 | 23 per 1000 (21 to 25) |  |  |  |  |  |
| **Total adverse reactions in vaccination group versus control group - Solicited systemic adverse reactions after the second vaccination** | Study population | | RR 1.88  (1.77 to 2.01) | 4847 (3 studies) | ⊕⊕⊕⊕ high | / |  |
|  | 376 per 1000 | 707 per 1000 (666 to 756) |  |  |  |  |  |
|  | Moderate | |  |  |  |  |  |
|  | 40 per 1000 | 75 per 1000 (71 to 80) |  |  |  |  |  |
| **Total adverse reactions in vaccination group versus control group - Solicited local adverse reactions after the third vaccination** | Study population | | RR 1.86  (0.55 to 6.3) | 995 (1 study) | ⊕⊕⊕⊕ high | / |  |
|  | 12 per 1000 | 23 per 1000 (7 to 77) |  |  |  |  |  |
|  | Moderate | |  |  |  |  |  |
|  | 12 per 1000 | 22 per 1000 (7 to 76) |  |  |  |  |  |
| **Total adverse reactions in vaccination group versus control group - Solicited systemic adverse reactions after the third vaccination** | Study population | | RR 2.3  (0.69 to 7.64) | 995 (1 study) | ⊕⊕⊕⊕ high^1^ | / |  |
|  | 12 per 1000 | 28 per 1000 (8 to 93) |  |  |  |  |  |
|  | Moderate | |  |  |  |  |  |
|  | 12 per 1000 | 28 per 1000 (8 to 92) |  |  |  |  |  |
| **Total adverse reactions after dose 1 - mRNA vaccine** | Study population | | RR 1.47  (1.41 to 1.54) | 3720 (1 study) | ⊕⊕⊕⊕ high | / |  |
|  | 651 per 1000 | 957 per 1000 (918 to 1000) |  |  |  |  |  |
|  | Moderate | |  |  |  |  |  |
|  | 651 per 1000 | 957 per 1000 (918 to 1000) |  |  |  |  |  |
| **Total adverse reactions after dose 1 - Inactivated vaccine** | Study population | | RR 1.27  (0.76 to 2.13) | 550 (1 study) | ⊕⊕⊕⊕ high | / |  |
|  | 132 per 1000 | 167 per 1000 (100 to 280) |  |  |  |  |  |
|  | Moderate | |  |  |  |  |  |
|  | 132 per 1000 | 168 per 1000 (100 to 281) |  |  |  |  |  |
| **Total adverse reactions after dose 1 - Virus-vectored vaccine** | Study population | | RR 3.44  (1.78 to 6.65) | 150 (1 study) | ⊕⊕⊕⊕ high^1,3^ | / |  |
|  | 160 per 1000 | 550 per 1000 (285 to 1000) |  |  |  |  |  |
|  | Moderate | |  |  |  |  |  |
|  | 160 per 1000 | 550 per 1000 (285 to 1000) |  |  |  |  |  |
| **Total adverse reactions after dose 2 - mRNA vaccine** | Study population | | RR 1.74  (1.66 to 1.83) | 3698 (1 study) | ⊕⊕⊕⊕ high | / |  |
|  | 557 per 1000 | 970 per 1000 (925 to 1000) |  |  |  |  |  |
|  | Moderate | |  |  |  |  |  |
|  | 557 per 1000 | 969 per 1000 (925 to 1000) |  |  |  |  |  |
| **Total adverse reactions after dose 2 - Inactivated vaccine** | Study population | | RR 1.83  (0.9 to 3.72) | 536 (1 study) | ⊕⊕⊕⊕ high | / |  |
|  | 72 per 1000 | 132 per 1000 (65 to 268) |  |  |  |  |  |
|  | Moderate | |  |  |  |  |  |
|  | 72 per 1000 | 132 per 1000 (65 to 268) |  |  |  |  |  |
| **Total adverse reactions after dose 2 - Virus-vectored vaccine** | Study population | | RR 8.25  (2.06 to 33) | 150 (1 study) | ⊕⊕⊕⊕ high^1,3^ | / |  |
|  | 40 per 1000 | 330 per 1000 (82 to 1000) |  |  |  |  |  |
|  | Moderate | |  |  |  |  |  |
|  | 40 per 1000 | 330 per 1000 (82 to 1000) |  |  |  |  |  |
| **Local adverse reactions after dose 1 - mRNA vaccine** | Study population | | RR 2.56  (2.38 to 2.76) | 3720 (1 study) | ⊕⊕⊕⊕ high^1^ | / |  |
|  | 368 per 1000 | 941 per 1000 (875 to 1000) |  |  |  |  |  |
|  | Moderate | |  |  |  |  |  |
|  | 368 per 1000 | 942 per 1000 (876 to 1000) |  |  |  |  |  |
| **Local adverse reactions after dose 1 - Inactivated vaccine** | Study population | | RR 6.34  (1.54 to 26.1) | 1007 (1 study) | ⊕⊕⊕⊕ high^1^ | / |  |
|  | 8 per 1000 | 50 per 1000 (12 to 207) |  |  |  |  |  |
|  | Moderate | |  |  |  |  |  |
|  | 8 per 1000 | 51 per 1000 (12 to 209) |  |  |  |  |  |
| **Local adverse reactions after dose 1 - Virus-vectored vaccine** | Study population | | RR 6  (1.94 to 18.53) | 150 (1 study) | ⊕⊕⊕⊕ high^1,3^ | / |  |
|  | 60 per 1000 | 360 per 1000 (116 to 1000) |  |  |  |  |  |
|  | Moderate | |  |  |  |  |  |
|  | 60 per 1000 | 360 per 1000 (116 to 1000) |  |  |  |  |  |
| **Local adverse reactions after dose 2 - mRNA vaccine** | Study population | | RR 2.86  (2.64 to 3.1) | 3698 (1 study) | ⊕⊕⊕⊕ high^1^ | / |  |
|  | 326 per 1000 | 933 per 1000 (861 to 1000) |  |  |  |  |  |
|  | Moderate | |  |  |  |  |  |
|  | 326 per 1000 | 932 per 1000 (861 to 1000) |  |  |  |  |  |
| **Local adverse reactions after dose 2 - Inactivated vaccine** | Study population | | RR 4.29  (1.03 to 17.96) | 999 (1 study) | ⊕⊕⊕⊕ high^1^ | / |  |
|  | 8 per 1000 | 35 per 1000 (8 to 145) |  |  |  |  |  |
|  | Moderate | |  |  |  |  |  |
|  | 8 per 1000 | 34 per 1000 (8 to 144) |  |  |  |  |  |
| **Local adverse reactions after dose 2 - Virus-vectored vaccine** | Study population | | RR 19.69  (1.21 to 319.62) | 150 (1 study) | ⊕⊕⊕⊕ high^1,3^ | / |  |
|  | 0 per 1000 | 0 per 1000 (0 to 0) |  |  |  |  |  |
|  | Moderate | |  |  |  |  |  |
|  | 0 per 1000 | 0 per 1000 (0 to 0) |  |  |  |  |  |
| **Systemic adverse reactions after dose 1 - mRNA vaccine** | Study population | | RR 1.23  (1.17 to 1.31) | 3720 (1 study) | ⊕⊕⊕⊕ high | / |  |
|  | 555 per 1000 | 683 per 1000 (649 to 727) |  |  |  |  |  |
|  | Moderate | |  |  |  |  |  |
|  | 555 per 1000 | 683 per 1000 (649 to 727) |  |  |  |  |  |
| **Systemic adverse reactions after dose 1 - Inactivated vaccine** | Study population | | RR 1.32  (0.87 to 2) | 1007 (1 study) | ⊕⊕⊕⊕ high | / |  |
|  | 99 per 1000 | 131 per 1000 (86 to 198) |  |  |  |  |  |
|  | Moderate | |  |  |  |  |  |
|  | 99 per 1000 | 131 per 1000 (86 to 198) |  |  |  |  |  |
| **Systemic adverse reactions after dose 1 - Virus-vectored vaccine** | Study population | | RR 3.7  (1.55 to 8.83) | 150 (1 study) | ⊕⊕⊕⊕ high^1,3^ | / |  |
|  | 100 per 1000 | 370 per 1000 (155 to 883) |  |  |  |  |  |
|  | Moderate | |  |  |  |  |  |
|  | 100 per 1000 | 370 per 1000 (155 to 883) |  |  |  |  |  |
| **Systemic adverse reactions after dose 2 - mRNA vaccine** | Study population | | RR 1.87  (1.76 to 1.99) | 3698 (1 study) | ⊕⊕⊕⊕ high | / |  |
|  | 460 per 1000 | 860 per 1000 (809 to 915) |  |  |  |  |  |
|  | Moderate | |  |  |  |  |  |
|  | 460 per 1000 | 860 per 1000 (810 to 915) |  |  |  |  |  |
| **Systemic adverse reactions after dose 2 - Inactivated vaccine** | Study population | | RR 1.61  (0.76 to 3.4) | 999 (1 study) | ⊕⊕⊕⊕ high | / |  |
|  | 32 per 1000 | 52 per 1000 (25 to 110) |  |  |  |  |  |
|  | Moderate | |  |  |  |  |  |
|  | 32 per 1000 | 52 per 1000 (24 to 109) |  |  |  |  |  |
| **Systemic adverse reactions after dose 2 - Virus-vectored vaccine** | Study population | | RR 6  (1.48 to 24.38) | 150 (1 study) | ⊕⊕⊕⊕ high^1,3^ | / |  |
|  | 40 per 1000 | 240 per 1000 (59 to 975) |  |  |  |  |  |
|  | Moderate | |  |  |  |  |  |
|  | 40 per 1000 | 240 per 1000 (59 to 975) |  |  |  |  |  |
| **Overall adverse reactions within 28 days after whole vaccination procedure** | Study population | | RR 1.6  (1.27 to 2.01) | 1557 (2 studies) | ⊕⊕⊕⊝ moderate^2^ | / |  |
|  | 183 per 1000 | 293 per 1000 (232 to 368) |  |  |  |  |  |
|  | Moderate | |  |  |  |  |  |
|  | 208 per 1000 | 333 per 1000 (264 to 418) |  |  |  |  |  |
| **Overall adverse reactions within 28 days after whole vaccination procedure - 3-5 years old** | Study population | | RR 1.15  (0.81 to 1.64) | 478 (2 studies) | ⊕⊕⊕⊕ high | / |  |
|  | 254 per 1000 | 293 per 1000 (206 to 417) |  |  |  |  |  |
|  | Moderate | |  |  |  |  |  |
|  | 269 per 1000 | 309 per 1000 (218 to 441) |  |  |  |  |  |
| **Overall adverse reactions within 28 days after whole vaccination procedure - 6- 11/ 12 years old** | Study population | | RR 2.41  (1.37 to 4.23) | 540 (2 studies) | ⊕⊕⊕⊕ high^1,2^ | / |  |
|  | 95 per 1000 | 230 per 1000 (130 to 403) |  |  |  |  |  |
|  | Moderate | |  |  |  |  |  |
|  | 113 per 1000 | 272 per 1000 (155 to 478) |  |  |  |  |  |
| **Overall adverse reactions within 28 days after whole vaccination procedure - 12/ 13- 17 years old** | Study population | | RR 1.71  (1.19 to 2.46) | 539 (2 studies) | ⊕⊕⊕⊕ high | / |  |
|  | 206 per 1000 | 353 per 1000 (246 to 508) |  |  |  |  |  |
|  | Moderate | |  |  |  |  |  |
|  | 220 per 1000 | 376 per 1000 (262 to 541) |  |  |  |  |  |
| **Specific adverse reactions in mRNA vaccine group versus control group after dose 1** | Study population | | RR 1.81  (1.74 to 1.88) | 90627 (3 studies) | ⊕⊕⊕⊝ moderate^2^ | / |  |
|  | 69 per 1000 | 124 per 1000 (119 to 129) |  |  |  |  |  |
|  | Moderate | |  |  |  |  |  |
|  | 10 per 1000 | 18 per 1000 (17 to 19) |  |  |  |  |  |
| **Specific adverse reactions in mRNA vaccine group versus control group after dose 2** | Study population | | RR 3.16  (3.03 to 3.3) | 89609 (3 studies) | ⊕⊕⊕⊕ high^1,2^ | / |  |
|  | 56 per 1000 | 175 per 1000 (168 to 183) |  |  |  |  |  |
|  | Moderate | |  |  |  |  |  |
|  | 10 per 1000 | 32 per 1000 (30 to 33) |  |  |  |  |  |
| **Specific adverse reactions in inactivated vaccine group versus control group after dose 1** | Study population | | RR 1.4  (1.04 to 1.9) | 23812 (2 studies) | ⊕⊕⊕⊕ high | / |  |
|  | 7 per 1000 | 10 per 1000 (8 to 14) |  |  |  |  |  |
|  | Moderate | |  |  |  |  |  |
|  | 2 per 1000 | 3 per 1000 (2 to 4) |  |  |  |  |  |
| **Specific adverse reactions in inactivated vaccine group versus control group after dose 2** | Study population | | RR 1.84  (1.2 to 2.81) | 21027 (2 studies) | ⊕⊕⊕⊕ high | / |  |
|  | 3 per 1000 | 6 per 1000 (4 to 9) |  |  |  |  |  |
|  | Moderate | |  |  |  |  |  |
|  | 0 per 1000 | 0 per 1000 (0 to 0) |  |  |  |  |  |
| **Specific adverse reactions in vectored vaccine group versus control group after dose 1** | Study population | | RR 3.87  (2.36 to 6.34) | 2700 (1 study) | ⊕⊕⊕⊕ high^1^ | / |  |
|  | 12 per 1000 | 47 per 1000 (29 to 77) |  |  |  |  |  |
|  | Moderate | |  |  |  |  |  |
|  | 0 per 1000 | 0 per 1000 (0 to 0) |  |  |  |  |  |
| **Specific adverse reactions in vectored vaccine group versus control group after dose 2** | Study population | | RR 4.03  (2.03 to 8.01) | 2700 (1 study) | ⊕⊕⊕⊕ high^1^ | / |  |
|  | 2 per 1000 | 9 per 1000 (5 to 18) |  |  |  |  |  |
|  | Moderate | |  |  |  |  |  |
|  | 0 per 1000 | 0 per 1000 (0 to 0) |  |  |  |  |  |
| **Adverse reactions in mRNA vaccine group of different ages versus control group: Local pain after the first vaccination - ≥12 years old** | Study population | | RR 2.71  (2.51 to 2.93) | 5980 (2 studies) | ⊕⊕⊕⊕ high^1,2^ | / |  |
|  | 192 per 1000 | 520 per 1000 (481 to 562) |  |  |  |  |  |
|  | Moderate | |  |  |  |  |  |
|  | 184 per 1000 | 499 per 1000 (462 to 539) |  |  |  |  |  |
| **Adverse reactions in mRNA vaccine group of different ages versus control group: Local pain after the first vaccination - ＜12 years old** | Study population | | RR 1.18  (0.79 to 1.78) | 2260 (1 study) | ⊕⊕⊕⊕ high | / |  |
|  | 41 per 1000 | 49 per 1000 (33 to 74) |  |  |  |  |  |
|  | Moderate | |  |  |  |  |  |
|  | 41 per 1000 | 48 per 1000 (32 to 73) |  |  |  |  |  |
| **Adverse reactions in mRNA vaccine group of different ages versus control group: Local pain after the second vaccination - ≥12 years old** | Study population | | RR 3.09  (2.84 to 3.37) | 5958 (2 studies) | ⊕⊕⊕⊕ high^1^ | / |  |
|  | 165 per 1000 | 510 per 1000 (469 to 557) |  |  |  |  |  |
|  | Moderate | |  |  |  |  |  |
|  | 160 per 1000 | 494 per 1000 (454 to 539) |  |  |  |  |  |
| **Adverse reactions in mRNA vaccine group of different ages versus control group: Local pain after the second vaccination - ＜12 years old** | Study population | | RR 1.21  (0.79 to 1.84) | 2242 (1 study) | ⊕⊕⊕⊕ high | / |  |
|  | 39 per 1000 | 47 per 1000 (31 to 72) |  |  |  |  |  |
|  | Moderate | |  |  |  |  |  |
|  | 39 per 1000 | 47 per 1000 (31 to 72) |  |  |  |  |  |
| **Adverse reactions in mRNA vaccine group of different ages versus control group: Erythema or redness after the first vaccination - ≥12 years old** | Study population | | RR 19.55  (10.04 to 38.06) | 5980 (2 studies) | ⊕⊕⊕⊕ high^1^ | / |  |
|  | 4 per 1000 | 74 per 1000 (38 to 145) |  |  |  |  |  |
|  | Moderate | |  |  |  |  |  |
|  | 4 per 1000 | 78 per 1000 (40 to 152) |  |  |  |  |  |
| **Adverse reactions in mRNA vaccine group of different ages versus control group: Erythema or redness after the first vaccination - ＜12 years old** | Study population | | RR 1.24  (0.48 to 3.18) | 2260 (1 study) | ⊕⊕⊕⊕ high | / |  |
|  | 8 per 1000 | 10 per 1000 (4 to 25) |  |  |  |  |  |
|  | Moderate | |  |  |  |  |  |
|  | 8 per 1000 | 10 per 1000 (4 to 25) |  |  |  |  |  |
| **Adverse reactions in mRNA vaccine group of different ages versus control group: Erythema or Redness after the second vaccination - ≥12 years old** | Study population | | RR 20.6  (11.6 to 36.58) | 5958 (2 studies) | ⊕⊕⊕⊕ high^1^ | / |  |
|  | 5 per 1000 | 105 per 1000 (59 to 187) |  |  |  |  |  |
|  | Moderate | |  |  |  |  |  |
|  | 5 per 1000 | 103 per 1000 (58 to 183) |  |  |  |  |  |
| **Adverse reactions in mRNA vaccine group of different ages versus control group: Erythema or Redness after the second vaccination - ＜12 years old** | Study population | | RR 1.88  (0.7 to 5) | 2242 (1 study) | ⊕⊕⊕⊕ high | / |  |
|  | 7 per 1000 | 13 per 1000 (5 to 34) |  |  |  |  |  |
|  | Moderate | |  |  |  |  |  |
|  | 7 per 1000 | 13 per 1000 (5 to 35) |  |  |  |  |  |
| **Adverse reactions in mRNA vaccine group of different ages versus control group: Swelling after the first vaccination - ≥12 years old** | Study population | | RR 16.18  (9.31 to 28.09) | 5980 (2 studies) | ⊕⊕⊕⊕ high^1^ | / |  |
|  | 5 per 1000 | 89 per 1000 (51 to 154) |  |  |  |  |  |
|  | Moderate | |  |  |  |  |  |
|  | 5 per 1000 | 81 per 1000 (47 to 140) |  |  |  |  |  |
| **Adverse reactions in mRNA vaccine group of different ages versus control group: Swelling after the first vaccination - ＜12 years old** | Study population | | RR 1.65  (0.46 to 5.99) | 2260 (1 study) | ⊕⊕⊕⊕ high | / |  |
|  | 4 per 1000 | 7 per 1000 (2 to 24) |  |  |  |  |  |
|  | Moderate | |  |  |  |  |  |
|  | 4 per 1000 | 7 per 1000 (2 to 24) |  |  |  |  |  |
| **Adverse reactions in mRNA vaccine group of different ages versus control group: Swelling after the second vaccination - ≥12 years old** | Study population | | RR 19.95  (11.5 to 34.61) | 5958 (2 studies) | ⊕⊕⊕⊕ high^1^ | / |  |
|  | 6 per 1000 | 110 per 1000 (64 to 192) |  |  |  |  |  |
|  | Moderate | |  |  |  |  |  |
|  | 5 per 1000 | 100 per 1000 (58 to 173) |  |  |  |  |  |
| **Adverse reactions in mRNA vaccine group of different ages versus control group: Swelling after the second vaccination - ＜12 years old** | Study population | | RR 2.47  (0.72 to 8.5) | 2242 (1 study) | ⊕⊕⊕⊕ high^1^ | / |  |
|  | 4 per 1000 | 10 per 1000 (3 to 34) |  |  |  |  |  |
|  | Moderate | |  |  |  |  |  |
|  | 4 per 1000 | 10 per 1000 (3 to 34) |  |  |  |  |  |
| **Adverse reactions in mRNA vaccine group of different ages versus control group: Fever after the first vaccination - ≥12 years old** | Study population | | RR 3.05  (1.71 to 5.44) | 5978 (2 studies) | ⊕⊕⊕⊕ high^1^ | / |  |
|  | 5 per 1000 | 17 per 1000 (9 to 30) |  |  |  |  |  |
|  | Moderate | |  |  |  |  |  |
|  | 5 per 1000 | 15 per 1000 (9 to 27) |  |  |  |  |  |
| **Adverse reactions in mRNA vaccine group of different ages versus control group: Fever after the first vaccination - ＜12 years old** | Study population | | RR 1.49  (0.15 to 14.27) | 2260 (1 study) | ⊕⊕⊕⊕ high | / |  |
|  | 1 per 1000 | 2 per 1000 (0 to 19) |  |  |  |  |  |
|  | Moderate | |  |  |  |  |  |
|  | 1 per 1000 | 1 per 1000 (0 to 14) |  |  |  |  |  |
| **Adverse reactions in mRNA vaccine group of different ages versus control group: Fever after the second vaccination - ≥12 years old** | Study population | | RR 12.84  (7.41 to 22.25) | 5957 (2 studies) | ⊕⊕⊕⊕ high^1^ | / |  |
|  | 6 per 1000 | 71 per 1000 (41 to 123) |  |  |  |  |  |
|  | Moderate | |  |  |  |  |  |
|  | 5 per 1000 | 64 per 1000 (37 to 111) |  |  |  |  |  |
| **Adverse reactions in mRNA vaccine group of different ages versus control group: Fever after the second vaccination - ＜12 years old** | Study population | | RR 3.46  (0.43 to 28.04) | 2242 (1 study) | ⊕⊕⊕⊕ high^1^ | / |  |
|  | 1 per 1000 | 5 per 1000 (1 to 38) |  |  |  |  |  |
|  | Moderate | |  |  |  |  |  |
|  | 1 per 1000 | 3 per 1000 (0 to 28) |  |  |  |  |  |
| **Adverse reactions in mRNA vaccine group of different ages versus control group: Headache after the first vaccination - ≥12 years old** | Study population | | RR 1.18  (1.09 to 1.28) | 5978 (2 studies) | ⊕⊕⊕⊝ moderate^2^ | / |  |
|  | 216 per 1000 | 255 per 1000 (236 to 277) |  |  |  |  |  |
|  | Moderate | |  |  |  |  |  |
|  | 208 per 1000 | 245 per 1000 (227 to 266) |  |  |  |  |  |
| **Adverse reactions in mRNA vaccine group of different ages versus control group: Headache after the first vaccination - ＜12 years old** | Study population | | RR 0.45  (0.26 to 0.8) | 2260 (1 study) | ⊕⊕⊕⊕ high^4^ | / |  |
|  | 32 per 1000 | 14 per 1000 (8 to 26) |  |  |  |  |  |
|  | Moderate | |  |  |  |  |  |
|  | 32 per 1000 | 14 per 1000 (8 to 26) |  |  |  |  |  |
| **Adverse reactions in mRNA vaccine group of different ages versus control group: Headache after the second vaccination - ≥12 years old** | Study population | | RR 2.33  (2.14 to 2.55) | 5958 (2 studies) | ⊕⊕⊕⊕ high^1^ | / |  |
|  | 168 per 1000 | 391 per 1000 (359 to 428) |  |  |  |  |  |
|  | Moderate | |  |  |  |  |  |
|  | 162 per 1000 | 377 per 1000 (347 to 413) |  |  |  |  |  |
| **Adverse reactions in mRNA vaccine group of different ages versus control group: Headache after the second vaccination - ＜12 years old** | Study population | | RR 0.73  (0.41 to 1.29) | 2242 (1 study) | ⊕⊕⊕⊕ high | / |  |
|  | 26 per 1000 | 19 per 1000 (11 to 33) |  |  |  |  |  |
|  | Moderate | |  |  |  |  |  |
|  | 26 per 1000 | 19 per 1000 (11 to 34) |  |  |  |  |  |
| **Adverse reactions in mRNA vaccine group of different ages versus control group: Fatigue after the first vaccination - ≥12 years old** | Study population | | RR 1.32  (1.21 to 1.43) | 5979 (2 studies) | ⊕⊕⊕⊕ high | / |  |
|  | 209 per 1000 | 275 per 1000 (253 to 298) |  |  |  |  |  |
|  | Moderate | |  |  |  |  |  |
|  | 201 per 1000 | 265 per 1000 (243 to 287) |  |  |  |  |  |
| **Adverse reactions in mRNA vaccine group of different ages versus control group: Fatigue after the first vaccination - ＜12 years old** | Study population | | RR 0.54  (0.34 to 0.88) | 2260 (1 study) | ⊕⊕⊕⊕ high | / |  |
|  | 41 per 1000 | 22 per 1000 (14 to 36) |  |  |  |  |  |
|  | Moderate | |  |  |  |  |  |
|  | 41 per 1000 | 22 per 1000 (14 to 36) |  |  |  |  |  |
| **Adverse reactions in mRNA vaccine group of different ages versus control group: Fatigue after the second vaccination - ≥12 years old** | Study population | | RR 2.36  (2.15 to 2.58) | 5958 (2 studies) | ⊕⊕⊕⊕ high^1^ | / |  |
|  | 161 per 1000 | 380 per 1000 (346 to 415) |  |  |  |  |  |
|  | Moderate | |  |  |  |  |  |
|  | 156 per 1000 | 368 per 1000 (335 to 402) |  |  |  |  |  |
| **Adverse reactions in mRNA vaccine group of different ages versus control group: Fatigue after the second vaccination - ＜12 years old** | Study population | | RR 0.8  (0.49 to 1.32) | 2242 (1 study) | ⊕⊕⊕⊕ high | / |  |
|  | 32 per 1000 | 26 per 1000 (16 to 43) |  |  |  |  |  |
|  | Moderate | |  |  |  |  |  |
|  | 32 per 1000 | 26 per 1000 (16 to 42) |  |  |  |  |  |
| **Adverse reactions in mRNA vaccine group of different ages versus control group: Myalgia after the first vaccination - ≥12 years old** | Study population | | RR 1.64  (1.43 to 1.88) | 5978 (2 studies) | ⊕⊕⊕⊕ high | / |  |
|  | 92 per 1000 | 151 per 1000 (132 to 173) |  |  |  |  |  |
|  | Moderate | |  |  |  |  |  |
|  | 89 per 1000 | 146 per 1000 (127 to 167) |  |  |  |  |  |
| **Adverse reactions in mRNA vaccine group of different ages versus control group: Myalgia after the first vaccination - ＜12 years old** | Study population | | RR 0.64  (0.24 to 1.7) | 2260 (1 study) | ⊕⊕⊕⊕ high | / |  |
|  | 9 per 1000 | 6 per 1000 (2 to 16) |  |  |  |  |  |
|  | Moderate | |  |  |  |  |  |
|  | 9 per 1000 | 6 per 1000 (2 to 15) |  |  |  |  |  |
|  | 9 per 1000 | 32 per 1000 (28 to 38) |  |  |  |  |  |
| **Adverse reactions in mRNA vaccine group of different ages versus control group: Myalgia after the second vaccination - ≥12 years old** | Study population | | RR 3.73  (3.2 to 4.33) | 5957 (2 studies) | ⊕⊕⊕⊕ high^1^ | / |  |
|  | 69 per 1000 | 256 per 1000 (219 to 297) |  |  |  |  |  |
|  | Moderate | |  |  |  |  |  |
|  | 66 per 1000 | 246 per 1000 (211 to 286) |  |  |  |  |  |
| **Adverse reactions in mRNA vaccine group of different ages versus control group: Myalgia after the second vaccination - ＜12 years old** | Study population | | RR 0.85  (0.33 to 2.14) | 2242 (1 study) | ⊕⊕⊕⊕ high | / |  |
|  | 9 per 1000 | 8 per 1000 (3 to 20) |  |  |  |  |  |
|  | Moderate | |  |  |  |  |  |
|  | 9 per 1000 | 8 per 1000 (3 to 19) |  |  |  |  |  |
| **Adverse reactions in mRNA vaccine group of different ages versus control group: Arthralgia after the first vaccination - ≥12 years old** | Study population | | RR 1.3  (1.09 to 1.55) | 5978 (2 studies) | ⊕⊕⊕⊕ high | / |  |
|  | 63 per 1000 | 82 per 1000 (69 to 98) |  |  |  |  |  |
|  | Moderate | |  |  |  |  |  |
|  | 61 per 1000 | 79 per 1000 (66 to 95) |  |  |  |  |  |
| **Adverse reactions in mRNA vaccine group of different ages versus control group: Arthralgia after the first vaccination - ＜12 years old** | Study population | | RR 0.3  (0.07 to 1.24) | 2260 (1 study) | ⊕⊕⊕⊕ high^4^ | / |  |
|  | 7 per 1000 | 2 per 1000 (0 to 8) |  |  |  |  |  |
|  | Moderate | |  |  |  |  |  |
|  | 7 per 1000 | 2 per 1000 (0 to 9) |  |  |  |  |  |
| **Adverse reactions in mRNA vaccine group of different ages versus control group: Arthralgia after the second vaccination - ≥12 years old** | Study population | | RR 3.12  (2.6 to 3.75) | 5957 (2 studies) | ⊕⊕⊕⊕ high^1^ | / |  |
|  | 50 per 1000 | 157 per 1000 (131 to 188) |  |  |  |  |  |
|  | Moderate | |  |  |  |  |  |
|  | 49 per 1000 | 153 per 1000 (127 to 184) |  |  |  |  |  |
| **Adverse reactions in mRNA vaccine group of different ages versus control group: Arthralgia after the second vaccination - ＜12 years old** | Study population | | RR 0.62  (0.17 to 2.29) | 2242 (1 study) | ⊕⊕⊕⊕ high | / |  |
|  | 5 per 1000 | 3 per 1000 (1 to 12) |  |  |  |  |  |
|  | Moderate | |  |  |  |  |  |
|  | 5 per 1000 | 3 per 1000 (1 to 11) |  |  |  |  |  |
| **Adverse reactions in mRNA vaccine group of different ages versus control group: Vomiting after the first vaccination - ≥12 years old** | Study population | | RR 2.99  (0.31 to 28.75) | 2260 (1 study) | ⊕⊕⊕⊕ high^1^ | / |  |
|  | 1 per 1000 | 3 per 1000 (0 to 25) |  |  |  |  |  |
|  | Moderate | |  |  |  |  |  |
|  | 1 per 1000 | 3 per 1000 (0 to 29) |  |  |  |  |  |
| **Adverse reactions in mRNA vaccine group of different ages versus control group: Vomiting after the first vaccination - ＜12 years old** | Study population | | RR 0.99  (0.09 to 10.92) | 2260 (1 study) | ⊕⊕⊕⊕ high | / |  |
|  | 1 per 1000 | 1 per 1000 (0 to 15) |  |  |  |  |  |
|  | Moderate | |  |  |  |  |  |
|  | 1 per 1000 | 1 per 1000 (0 to 11) |  |  |  |  |  |
| **Adverse reactions in mRNA vaccine group of different ages versus control group: Vomiting after the second vaccination - ≥12 years old** | Study population | | RR 2.99  (0.31 to 28.75) | 2260 (1 study) | ⊕⊕⊕⊕ high^1^ | / |  |
|  | 1 per 1000 | 3 per 1000 (0 to 25) |  |  |  |  |  |
|  | Moderate | |  |  |  |  |  |
|  | 1 per 1000 | 3 per 1000 (0 to 29) |  |  |  |  |  |
| **Adverse reactions in mRNA vaccine group of different ages versus control group: Vomiting after the second vaccination - ＜12 years old** | Study population | | RR 0.99  (0.09 to 10.87) | 2242 (1 study) | ⊕⊕⊕⊕ high | / |  |
|  | 1 per 1000 | 1 per 1000 (0 to 15) |  |  |  |  |  |
|  | Moderate | |  |  |  |  |  |
|  | 1 per 1000 | 1 per 1000 (0 to 11) |  |  |  |  |  |
| **Adverse reactions in mRNA vaccine group of different ages versus control group: Diarrhea after the first vaccination - ≥12 years old** | Study population | | RR 1.14  (0.42 to 3.14) | 2260 (1 study) | ⊕⊕⊕⊕ high | / |  |
|  | 6 per 1000 | 7 per 1000 (3 to 19) |  |  |  |  |  |
|  | Moderate | |  |  |  |  |  |
|  | 6 per 1000 | 7 per 1000 (3 to 19) |  |  |  |  |  |
| **Adverse reactions in mRNA vaccine group of different ages versus control group: Diarrhea after the first vaccination - ＜12 years old** | Study population | | RR 0.74  (0.21 to 2.63) | 2260 (1 study) | ⊕⊕⊕⊕ high | / |  |
|  | 5 per 1000 | 4 per 1000 (1 to 14) |  |  |  |  |  |
|  | Moderate | |  |  |  |  |  |
|  | 5 per 1000 | 4 per 1000 (1 to 13) |  |  |  |  |  |
| **Adverse reactions in mRNA vaccine group of different ages versus control group: Diarrhea after the second vaccination - ≥12 years old** | Study population | | RR 1.5  (0.42 to 5.29) | 2260 (1 study) | ⊕⊕⊕⊕ high | / |  |
|  | 4 per 1000 | 5 per 1000 (1 to 19) |  |  |  |  |  |
|  | Moderate | |  |  |  |  |  |
|  | 4 per 1000 | 6 per 1000 (2 to 21) |  |  |  |  |  |
| **Adverse reactions in mRNA vaccine group of different ages versus control group: Diarrhea after the second vaccination - ＜12 years old** | Study population | | RR 0.1  (0.03 to 0.36) | 1657 (1 study) | ⊕⊕⊕⊕ high^4^ | / |  |
|  | 32 per 1000 | 3 per 1000 (1 to 12) |  |  |  |  |  |
|  | Moderate | |  |  |  |  |  |
|  | 32 per 1000 | 3 per 1000 (1 to 12) |  |  |  |  |  |
| **Adverse reactions in mRNA vaccine group of different ages versus control group: Chills after the first vaccination - ≥12 years old** | Study population | | RR 1.71  (1.44 to 2.03) | 5978 (2 studies) | ⊕⊕⊕⊕ high | / |  |
|  | 63 per 1000 | 107 per 1000 (90 to 127) |  |  |  |  |  |
|  | Moderate | |  |  |  |  |  |
|  | 60 per 1000 | 103 per 1000 (86 to 122) |  |  |  |  |  |
| **Adverse reactions in mRNA vaccine group of different ages versus control group: Chills after the first vaccination - ＜12 years old** | Study population | | RR 0.5  (0.14 to 1.71) | 2260 (1 study) | ⊕⊕⊕⊕ high | / |  |
|  | 7 per 1000 | 3 per 1000 (1 to 11) |  |  |  |  |  |
|  | Moderate | |  |  |  |  |  |
|  | 7 per 1000 | 3 per 1000 (1 to 12) |  |  |  |  |  |
| **Adverse reactions in mRNA vaccine group of different ages versus control group: Chills after the second vaccination - ≥12 years old** | Study population | | RR 5.44  (4.5 to 6.58) | 5957 (2 studies) | ⊕⊕⊕⊕ high^1^ | / |  |
|  | 44 per 1000 | 241 per 1000 (199 to 291) |  |  |  |  |  |
|  | Moderate | |  |  |  |  |  |
|  | 43 per 1000 | 234 per 1000 (194 to 283) |  |  |  |  |  |
| **Adverse reactions in mRNA vaccine group of different ages versus control group: Chills after the second vaccination - ＜12 years old** | Study population | | RR 1.23  (0.39 to 3.92) | 2242 (1 study) | ⊕⊕⊕⊕ high | / |  |
|  | 5 per 1000 | 7 per 1000 (2 to 21) |  |  |  |  |  |
|  | Moderate | |  |  |  |  |  |
|  | 5 per 1000 | 6 per 1000 (2 to 20) |  |  |  |  |  |
| *The basis for the assumed risk (e.g. the median control group risk across studies) is provided in footnotes. The corresponding risk (and its 95% confidence interval) is based on the assumed risk in the comparison group and the relative effect of the intervention (and its 95% CI).  CI: Confidence interval; RR: Risk ratio; | | | | | | | |
| GRADE Working Group grades of evidence High quality: Further research is very unlikely to change our confidence in the estimate of effect.  Moderate quality: Further research is likely to have an important impact on our confidence in the estimate of effect and may change the estimate. Low quality: Further research is very likely to have an important impact on our confidence in the estimate of effect and is likely to change the estimate. Very low quality: We are very uncertain about the estimate. | | | | | | | |
| ^1^ The relative effect is greater than 2.0. ^2^ The statistical test for heterogeneity showed that large variation existed in point estimates. ^3^ Limited sample size.  ^4^ The relative effect is less than 0.5. | | | | | | | |

| **Supplementary Table 4. Summary of findings table for the safety of COVID-19 vaccines (Dose 1 versus Dose 2)** | | | | | | |
| --- | --- | --- | --- | --- | --- | --- |
| **Patient or population:** adolescents, children, and infants **Intervention:** Dose 1 versus Dose 2 | | | | | | |
| **Outcomes** | **Illustrative comparative risks* (95% CI)** | | **Relative effect (95% CI)** | **No of Participants (studies)** | **Quality of the evidence (GRADE)** | **Comments** |
|  | Assumed risk | Corresponding risk |  |  |  |  |
|  | **Control** | **Dose 1 versus Dose 2** |  |  |  |  |
| **Total reactions in vaccination group after dose 1 versus after dose 2 - Total adverse reactions** | Study population | | RR 1  (0.99 to 1.02) | 6021 (3 studies) | ⊕⊕⊕⊝ moderate^1^ | / |
|  | 831 per 1000 | 831 per 1000 (822 to 847) |  |  |  |  |
|  | Moderate | |  |  |  |  |
|  | 330 per 1000 | 330 per 1000 (327 to 337) |  |  |  |  |
| **Total reactions in vaccination group after dose 1 versus after dose 2 - Local adverse reactions** | Study population | | RR 1.02  (1 to 1.04) | 6666 (3 studies) | ⊕⊕⊕⊝ moderate^1^ | / |
|  | 709 per 1000 | 723 per 1000 (709 to 737) |  |  |  |  |
|  | Moderate | |  |  |  |  |
|  | 190 per 1000 | 194 per 1000 (190 to 198) |  |  |  |  |
| **Total reactions in vaccination group after dose 1 versus after dose 2 - Systemic adverse reactions** | Study population | | RR 0.83  (0.81 to 0.86) | 6666 (3 studies) | ⊕⊕⊕⊝ moderate^1^ | / |
|  | 660 per 1000 | 548 per 1000 (535 to 568) |  |  |  |  |
|  | Moderate | |  |  |  |  |
|  | 240 per 1000 | 199 per 1000 (194 to 206) |  |  |  |  |
| *The basis for the assumed risk (e.g. the median control group risk across studies) is provided in footnotes. The corresponding risk (and its 95% confidence interval) is based on the assumed risk in the comparison group and the relative effect of the intervention (and its 95% CI).  CI: Confidence interval; RR: Risk ratio; | | | | | | |
| GRADE Working Group grades of evidence High quality: Further research is very unlikely to change our confidence in the estimate of effect.  Moderate quality: Further research is likely to have an important impact on our confidence in the estimate of effect and may change the estimate. Low quality: Further research is very likely to have an important impact on our confidence in the estimate of effect and is likely to change the estimate. Very low quality: We are very uncertain about the estimate. | | | | | | |
| ^1^ The statistical test for heterogeneity showed that large variation existed in point estimates. | | | | | | |

| **Supplementary Table 5. Summary of findings table for the safety of COVID-19 vaccines (mRNA vaccine recipients aged ≥12 years versus <12 years)** | | | | | | |
| --- | --- | --- | --- | --- | --- | --- |
| **Patient or population:** adolescents, children, and infants **Intervention:** mRNA vaccine recipients aged ≥12 years versus <12 years | | | | | | |
| **Outcomes** | **Illustrative comparative risks* (95% CI)** | | **Relative effect (95% CI)** | **No of Participants (studies)** | **Quality of the evidence (GRADE)** | **Comments** |
|  | Assumed risk | Corresponding risk |  |  |  |  |
|  | **Control** | **MRNA vaccine recipients aged ≥12 years versus <12 years** |  |  |  |  |
| **After the first vaccination** | Study population | | RR 1.4  (1.21 to 1.62) | 49720 (2 studies) | ⊕⊕⊕⊝ moderate^1^ | / |
|  | 12 per 1000 | 17 per 1000 (15 to 20) |  |  |  |  |
|  | Moderate | |  |  |  |  |
|  | 7 per 1000 | 10 per 1000 (8 to 11) |  |  |  |  |
| **After the second vaccination** | Study population | | RR 2.04  (1.75 to 2.38) | 48937 (2 studies) | ⊕⊕⊕⊕ high^1,2^ | / |
|  | 10 per 1000 | 20 per 1000 (17 to 23) |  |  |  |  |
|  | Moderate | |  |  |  |  |
|  | 6 per 1000 | 12 per 1000 (11 to 14) |  |  |  |  |
| *The basis for the assumed risk (e.g. the median control group risk across studies) is provided in footnotes. The corresponding risk (and its 95% confidence interval) is based on the assumed risk in the comparison group and the relative effect of the intervention (and its 95% CI).  CI: Confidence interval; RR: Risk ratio; | | | | | | |
| GRADE Working Group grades of evidence High quality: Further research is very unlikely to change our confidence in the estimate of effect.  Moderate quality: Further research is likely to have an important impact on our confidence in the estimate of effect and may change the estimate. Low quality: Further research is very likely to have an important impact on our confidence in the estimate of effect and is likely to change the estimate. Very low quality: We are very uncertain about the estimate. | | | | | | |
| ^1^ The statistical test for heterogeneity showed that large variation existed in point estimates. ^2^ The relative effect is greater than 2.0. | | | | | | |

| **Supplementary Table 6. Summary of findings table for the immunogenicity and efficacy of COVID-19 vaccines (Vaccine versus Placebo)** | | | | | | |
| --- | --- | --- | --- | --- | --- | --- |
| **Patient or population:** adolescents, children, and infants **Intervention:** Vaccine versus Placebo | | | | | | |
| **Outcomes** | **Illustrative comparative risks* (95% CI)** | | **Relative effect (95% CI)** | **No of Participants (studies)** | **Quality of the evidence (GRADE)** | **Comments** |
|  | Assumed risk | Corresponding risk |  |  |  |  |
|  | **Control** | **Vaccine versus Placebo** |  |  |  |  |
| **Seroconversion(Pseudovirus neutralizing antibody) - 28 days after Dose 1** | Study population | | RR 77.99  (28.4 to 214.14) | 1212 (3 studies) | ⊕⊕⊕⊕ high^1,2^ | / |
|  | 10 per 1000 | 745 per 1000 (271 to 1000) |  |  |  |  |
|  | Moderate | |  |  |  |  |
|  | 0 per 1000 | 0 per 1000 (0 to 0) |  |  |  |  |
| **Seroconversion(Pseudovirus neutralizing antibody) - 28 days after Dose 2** | Study population | | RR 144.8  (44.97 to 466.24) | 1674 (3 studies) | ⊕⊕⊕⊕ high^1,2^ | / |
|  | 5 per 1000 | 713 per 1000 (222 to 1000) |  |  |  |  |
|  | Moderate | |  |  |  |  |
|  | 0 per 1000 | 0 per 1000 (0 to 0) |  |  |  |  |
| **Seroconversion 28 days after Dose 2(Neutralizing antibody) - 3-5 years old** | Study population | | RR 110.57  (15.87 to 770.57) | 467 (2 studies) | ⊕⊕⊕⊕ high^1^ | / |
|  | 0 per 1000 | 0 per 1000 (0 to 0) |  |  |  |  |
|  | Moderate | |  |  |  |  |
|  | 0 per 1000 | 0 per 1000 (0 to 0) |  |  |  |  |
| **Seroconversion 28 days after Dose 2(Neutralizing antibody) - 6- 11/ 12 years old** | Study population | | RR 124.37  (17.79 to 869.21) | 529 (2 studies) | ⊕⊕⊕⊕ high^1^ | / |
|  | 0 per 1000 | 0 per 1000 (0 to 0) |  |  |  |  |
|  | Moderate | |  |  |  |  |
|  | 0 per 1000 | 0 per 1000 (0 to 0) |  |  |  |  |
| **Seroconversion 28 days after Dose 2(Neutralizing antibody) - 12/ 13- 17 years old** | Study population | | RR 121.28  (17.36 to 847.06) | 528 (2 studies) | ⊕⊕⊕⊕ high^1^ | / |
|  | 0 per 1000 | 0 per 1000 (0 to 0) |  |  |  |  |
|  | Moderate | |  |  |  |  |
|  | 0 per 1000 | 0 per 1000 (0 to 0) |  |  |  |  |
| **Seroconversion(RBD-binding ELISA antibody) - 28 days after Dose 1** | Study population | | RR 99.48  (6.31 to 1569.12) | 150 (1 study) | ⊕⊕⊕⊕ high^1,3^ | / |
|  | 0 per 1000 | 0 per 1000 (0 to 0) |  |  |  |  |
|  | Moderate | |  |  |  |  |
|  | 0 per 1000 | 0 per 1000 (0 to 0) |  |  |  |  |
| **Seroconversion(RBD-binding ELISA antibody) - 56 days after Dose 1 (Bfter Dose 2)** | Study population | | RR 98.47  (6.24 to 1553.3) | 150 (1 study) | ⊕⊕⊕⊕ high^1,3^ | / |
|  | 0 per 1000 | 0 per 1000 (0 to 0) |  |  |  |  |
|  | Moderate | |  |  |  |  |
|  | 0 per 1000 | 0 per 1000 (0 to 0) |  |  |  |  |
| **Seroconversion(RBD-binding ELISA antibody) - 28 days after Dose 2** | Study population | | RR 101.5  (6.44 to 1600.76) | 150 (1 study) | ⊕⊕⊕⊕ high^1,3^ | / |
|  | 0 per 1000 | 0 per 1000 (0 to 0) |  |  |  |  |
|  | Moderate | |  |  |  |  |
|  | 0 per 1000 | 0 per 1000 (0 to 0) |  |  |  |  |
| **Covid-19 after the vaccination - After dose 1 to before dose 2** | Study population | | RR 0.25  (0.07 to 0.88) | 2260 (1 study) | ⊕⊕⊕⊕ high^4^ | / |
|  | 11 per 1000 | 3 per 1000 (1 to 9) |  |  |  |  |
|  | Moderate | |  |  |  |  |
|  | 11 per 1000 | 3 per 1000 (1 to 10) |  |  |  |  |
| **Covid-19 after the vaccination - within 7 days after the second dose** | Study population | | RR 0.09  (0.01 to 1.64) | 2260 (1 study) | ⊕⊕⊕⊕ high^4^ | / |
|  | 4 per 1000 | 0 per 1000 (0 to 7) |  |  |  |  |
|  | Moderate | |  |  |  |  |
|  | 4 per 1000 | 0 per 1000 (0 to 7) |  |  |  |  |
| **Covid-19 after the vaccination - 7 days after second dose** | Study population | | RR 0.06  (0.02 to 0.2) | 4197 (2 studies) | ⊕⊕⊕⊕ high^4^ | / |
|  | 19 per 1000 | 1 per 1000 (0 to 4) |  |  |  |  |
|  | Moderate | |  |  |  |  |
|  | 20 per 1000 | 1 per 1000 (0 to 4) |  |  |  |  |
| **Covid-19 after the vaccination - 14 days after second dose** | Study population | | RR 0.07  (0.01 to 0.56) | 3181 (1 study) | ⊕⊕⊕⊕ high^4^ | / |
|  | 7 per 1000 | 0 per 1000 (0 to 4) |  |  |  |  |
|  | Moderate | |  |  |  |  |
|  | 7 per 1000 | 0 per 1000 (0 to 4) |  |  |  |  |
| **Covid-19 after dose 2** | Study population | | RR 0.06  (0.02 to 0.18) | 7378 (3 studies) | ⊕⊕⊕⊕ high^4^ | / |
|  | 15 per 1000 | 1 per 1000 (0 to 3) |  |  |  |  |
|  | Moderate | |  |  |  |  |
|  | 16 per 1000 | 1 per 1000 (0 to 3) |  |  |  |  |
| **Covid-19 after dose 2 - mRNA-1273 vaccine** | Study population | | RR 0.07  (0.01 to 0.56) | 3181 (1 study) | ⊕⊕⊕⊕ high^4^ | / |
|  | 7 per 1000 | 0 per 1000 (0 to 4) |  |  |  |  |
|  | Moderate | |  |  |  |  |
|  | 7 per 1000 | 0 per 1000 (0 to 4) |  |  |  |  |
| **Covid-19 after dose 2 - BNT162b2 Covid-19 Vaccine** | Study population | | RR 0.06  (0.02 to 0.2) | 4197 (2 studies) | ⊕⊕⊕⊕ high^4^ | / |
|  | 19 per 1000 | 1 per 1000 (0 to 4) |  |  |  |  |
|  | Moderate | |  |  |  |  |
|  | 20 per 1000 | 1 per 1000 (0 to 4) |  |  |  |  |
| *The basis for the assumed risk (e.g. the median control group risk across studies) is provided in footnotes. The corresponding risk (and its 95% confidence interval) is based on the assumed risk in the comparison group and the relative effect of the intervention (and its 95% CI).  CI: Confidence interval; RR: Risk ratio; | | | | | | |
| GRADE Working Group grades of evidence High quality: Further research is very unlikely to change our confidence in the estimate of effect.  Moderate quality: Further research is likely to have an important impact on our confidence in the estimate of effect and may change the estimate. Low quality: Further research is very likely to have an important impact on our confidence in the estimate of effect and is likely to change the estimate. Very low quality: We are very uncertain about the estimate. | | | | | | |
| ^1^ The relative effect is greater than 2.0. ^2^ The statistical test for heterogeneity showed that large variation existed in point estimates. ^3^ Limited sample size.  ^4^ The relative effect is less than 0.5. | | | | | | |
