# Supplementary figures and images for: Safety, Immunogenicity, and Efficacy of COVID-19 Vaccines in Adolescents, Children, and Infants: A Systematic Review and Meta-Analysis
Source: Front Public Health. 2022 Apr 14;10:829176. doi: 10.3389/fpubh.2022.829176 (PMC9046659; doi:10.3389/fpubh.2022.829176)

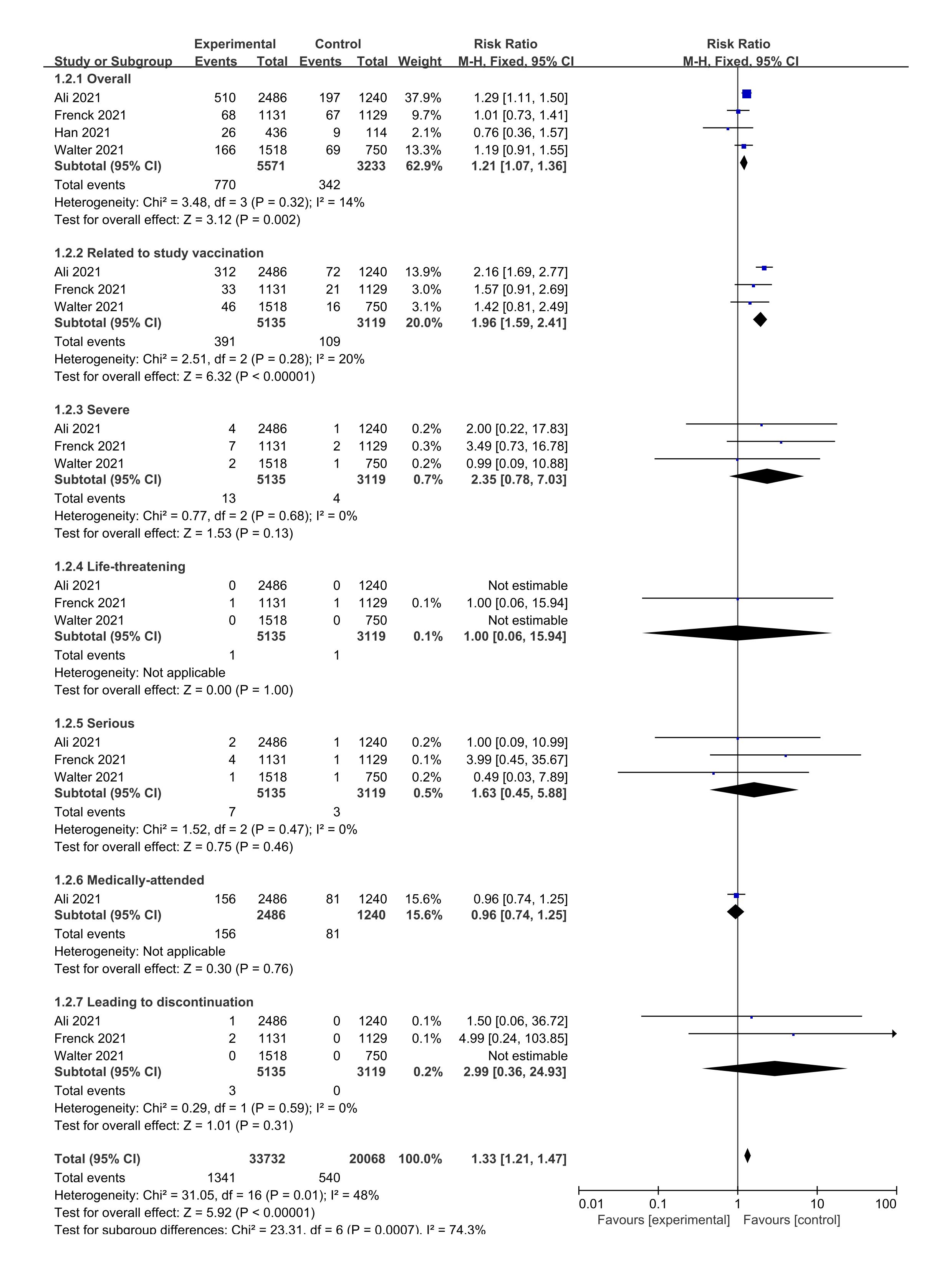

Supplement: Supplementary file 1 [file Data_Sheet_1.ZIP › Supplementary Material/Supplementary Figures (JPG)/Supplementary Figure 1. Unsolicited adverse reactions within 28 or 30 days after whole vaccination procedure.jpg]

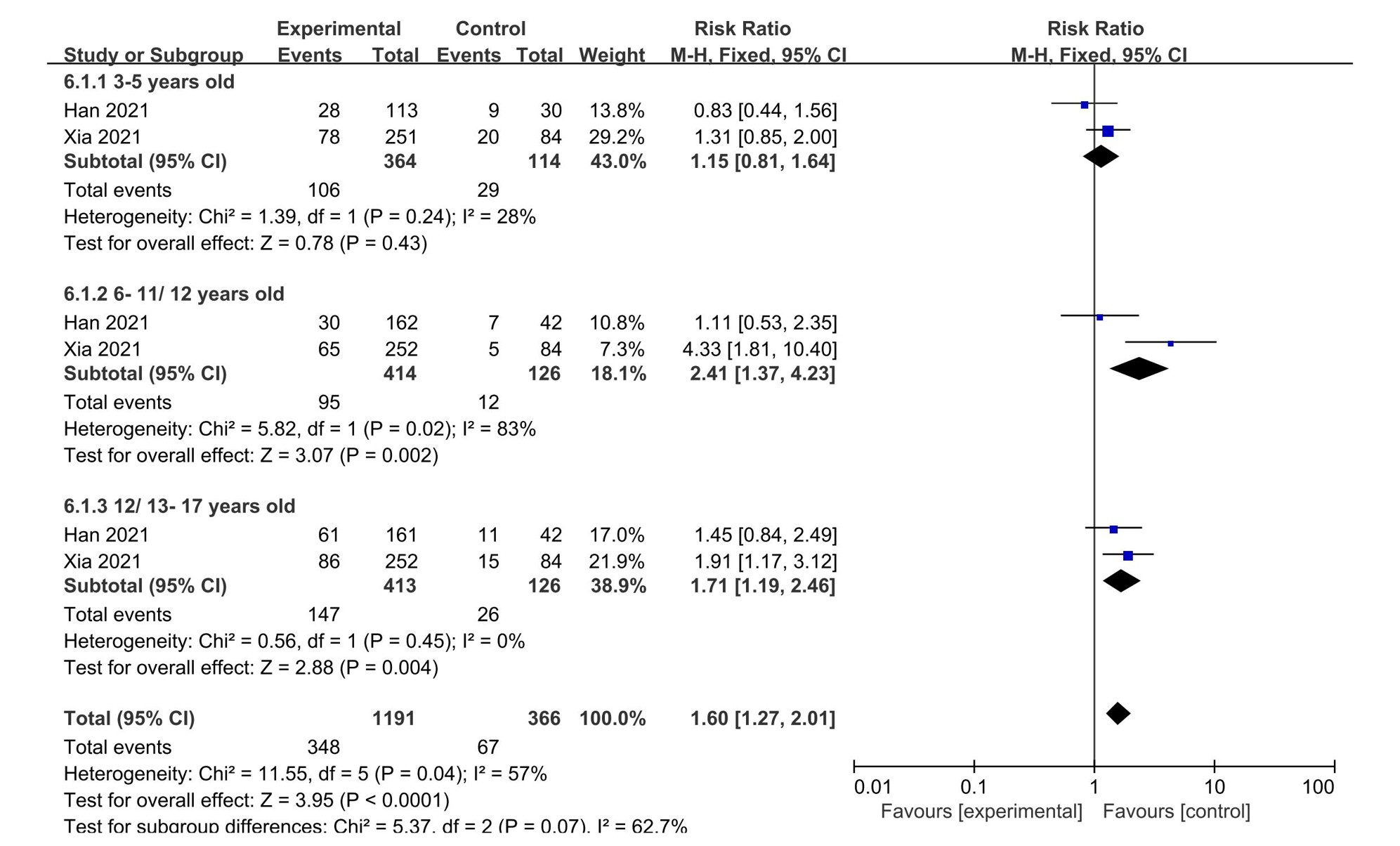

Supplement: Supplementary file 1 [file Data_Sheet_1.ZIP › Supplementary Material/Supplementary Figures (JPG)/Supplementary Figure 10. Overall adverse reactions within 28 days after whole vaccination procedure in inactivated vaccine group of different ages versus control group.jpg]

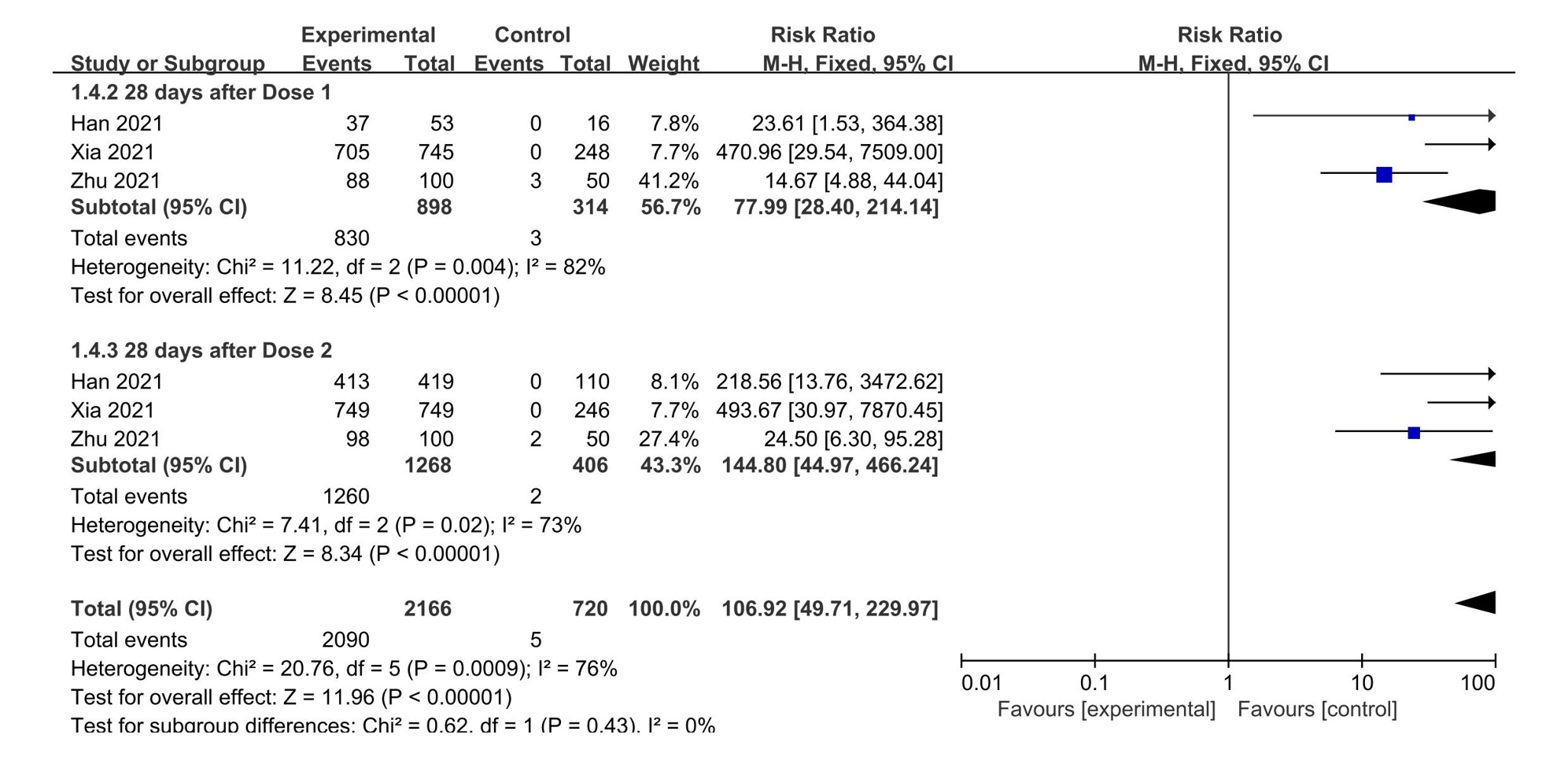

Supplement: Supplementary file 1 [file Data_Sheet_1.ZIP › Supplementary Material/Supplementary Figures (JPG)/Supplementary Figure 11. Seroconversion rate in vaccine group versus control group/(A) Pseudovirus neutralizing antibody.jpg]

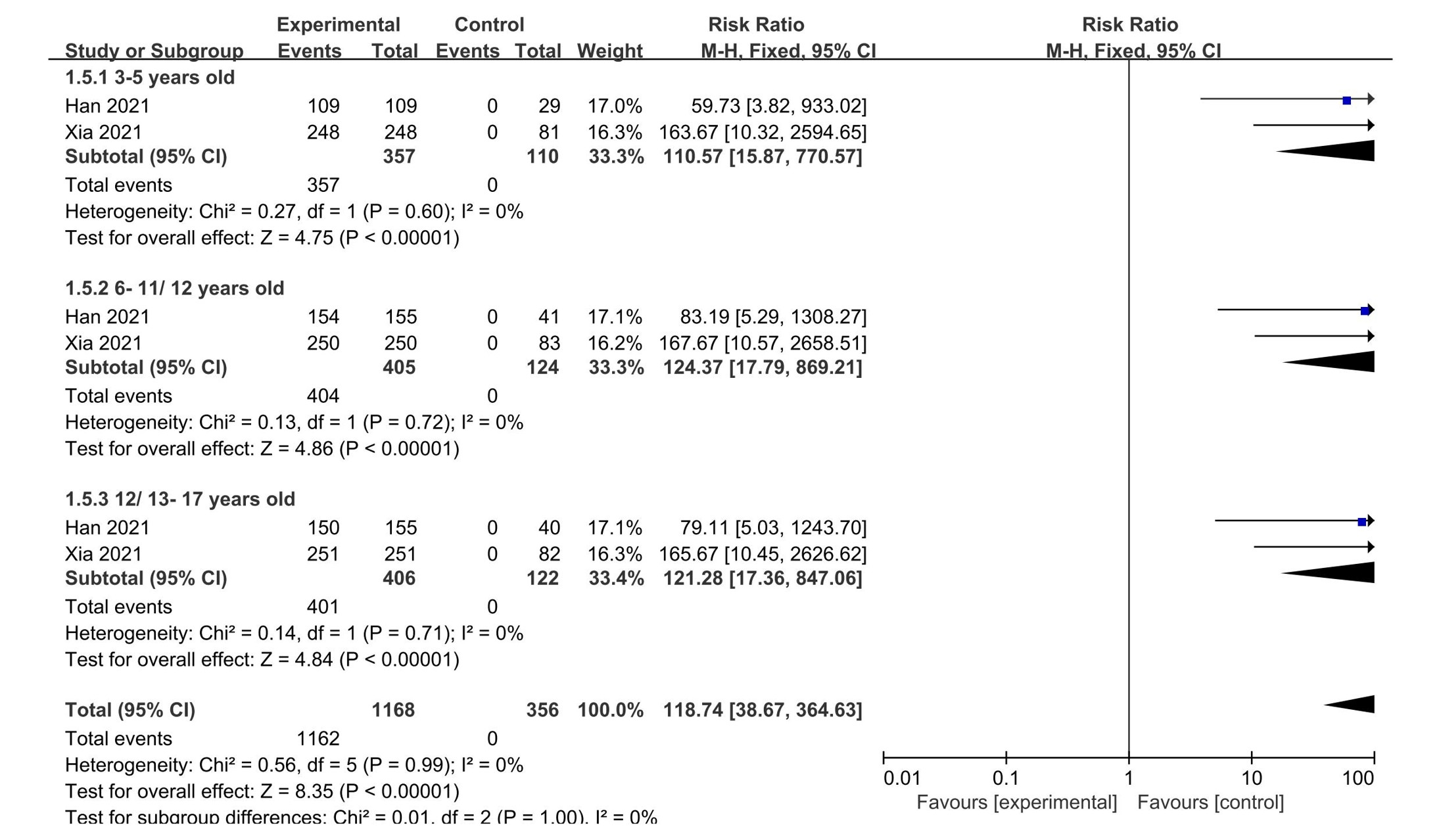

Supplement: Supplementary file 1 [file Data_Sheet_1.ZIP › Supplementary Material/Supplementary Figures (JPG)/Supplementary Figure 11. Seroconversion rate in vaccine group versus control group/(B) Neutralizing antibody 28 days after Dose 2.jpg]

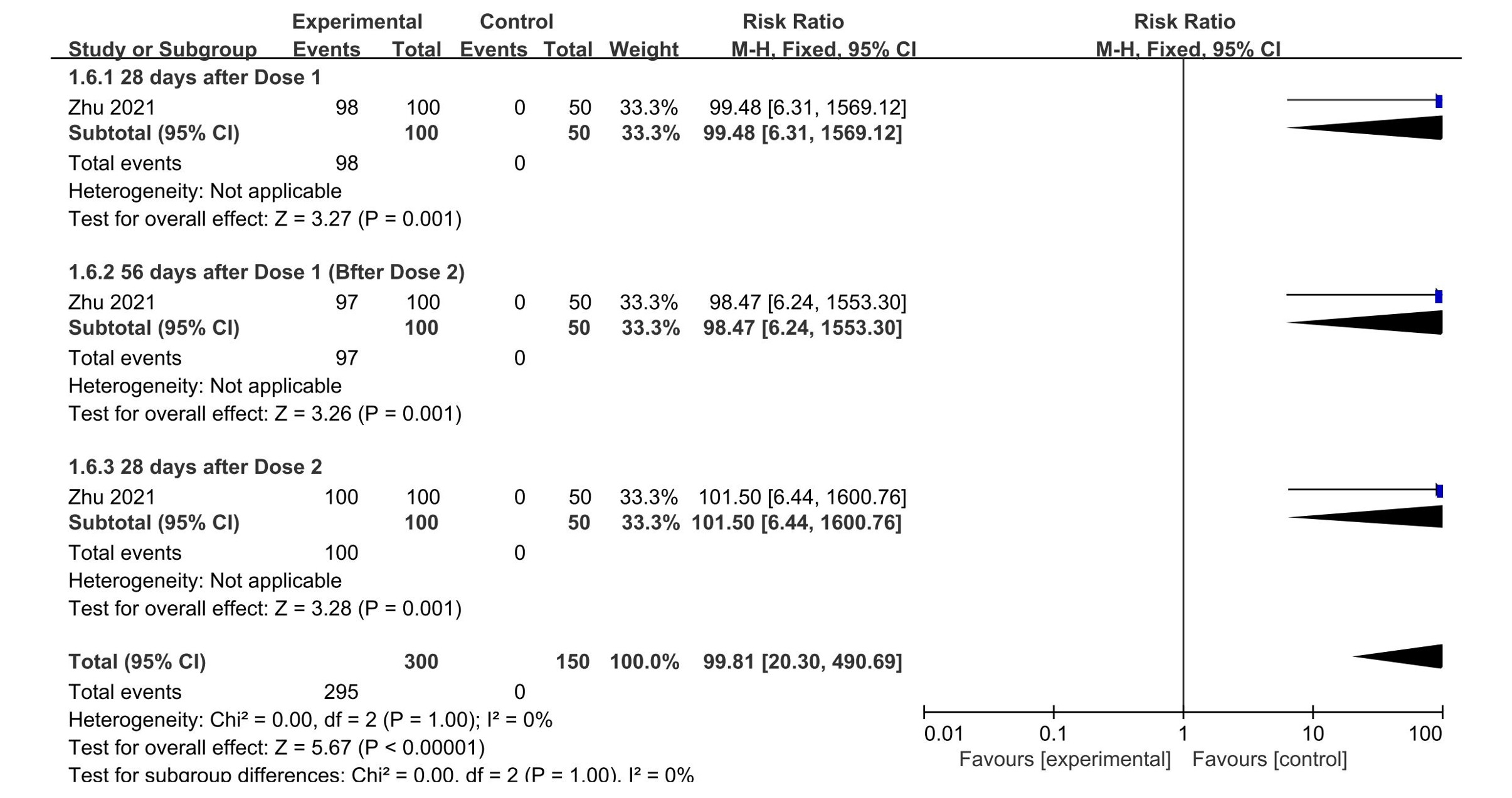

Supplement: Supplementary file 1 [file Data_Sheet_1.ZIP › Supplementary Material/Supplementary Figures (JPG)/Supplementary Figure 11. Seroconversion rate in vaccine group versus control group/(C) RBD¿Cbinding enzyme-linked immunosorbent assay antibody.jpg]

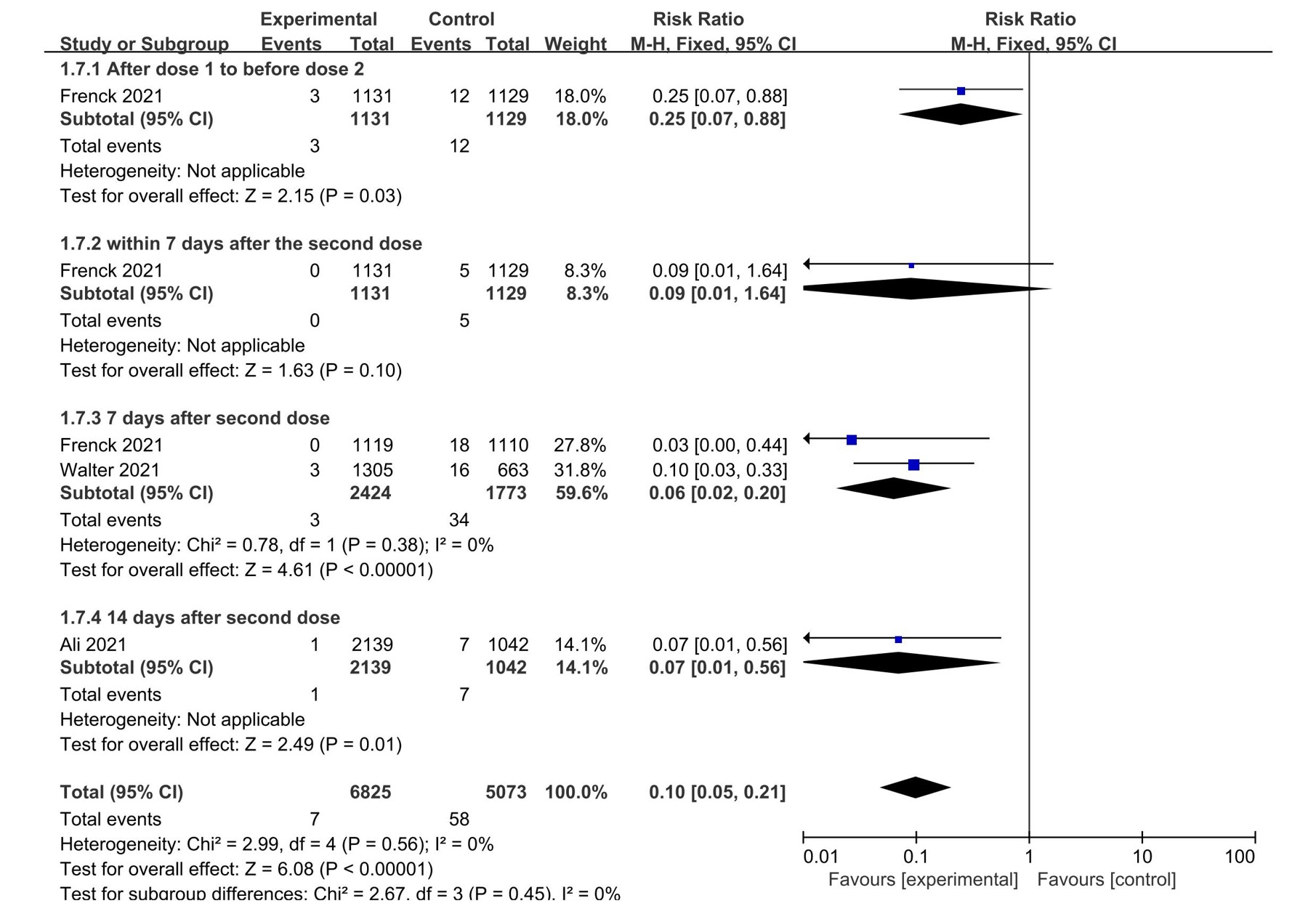

Supplement: Supplementary file 1 [file Data_Sheet_1.ZIP › Supplementary Material/Supplementary Figures (JPG)/Supplementary Figure 12. COVID-19 diagnosed after vaccination in vaccine group versus control group/(A) Covid-19 after the vaccination.jpg]

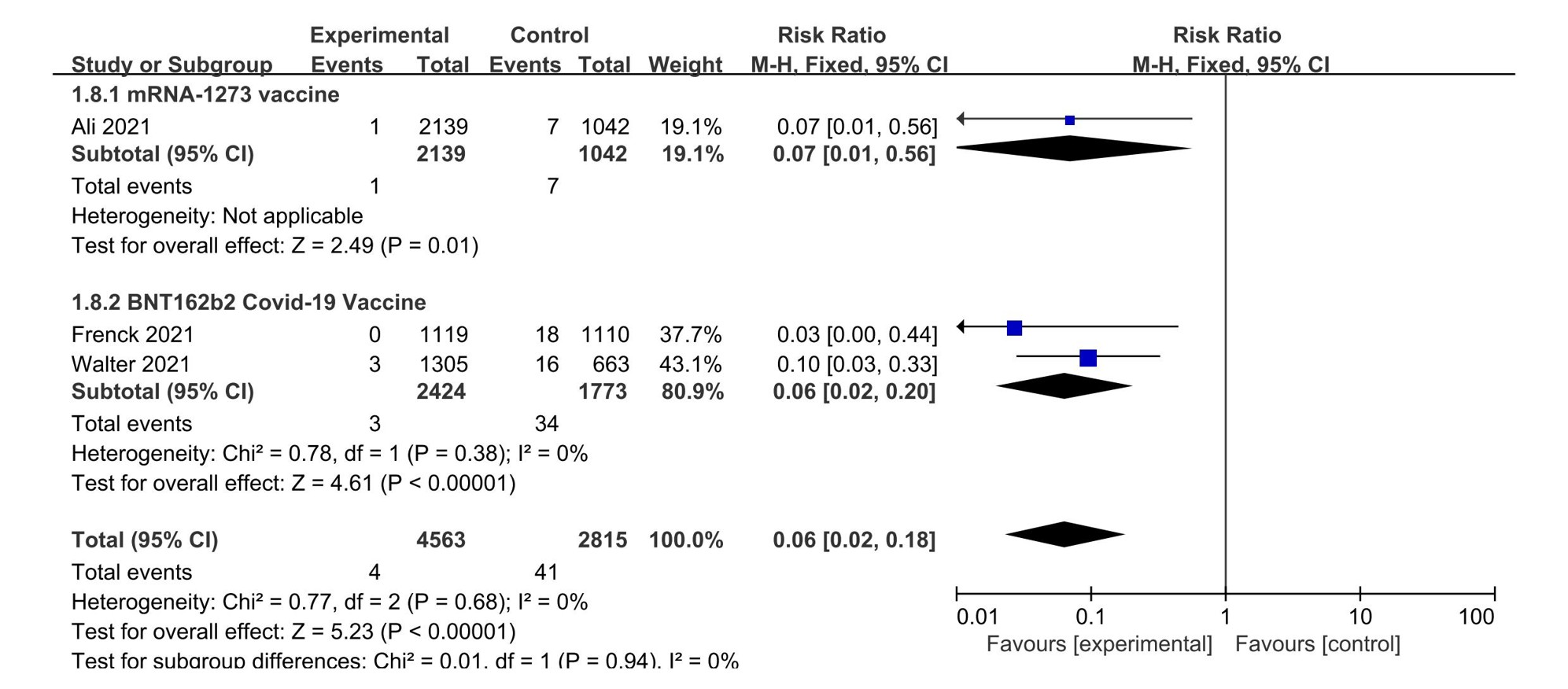

Supplement: Supplementary file 1 [file Data_Sheet_1.ZIP › Supplementary Material/Supplementary Figures (JPG)/Supplementary Figure 12. COVID-19 diagnosed after vaccination in vaccine group versus control group/(B) Covid-19 after dose 2.jpg]

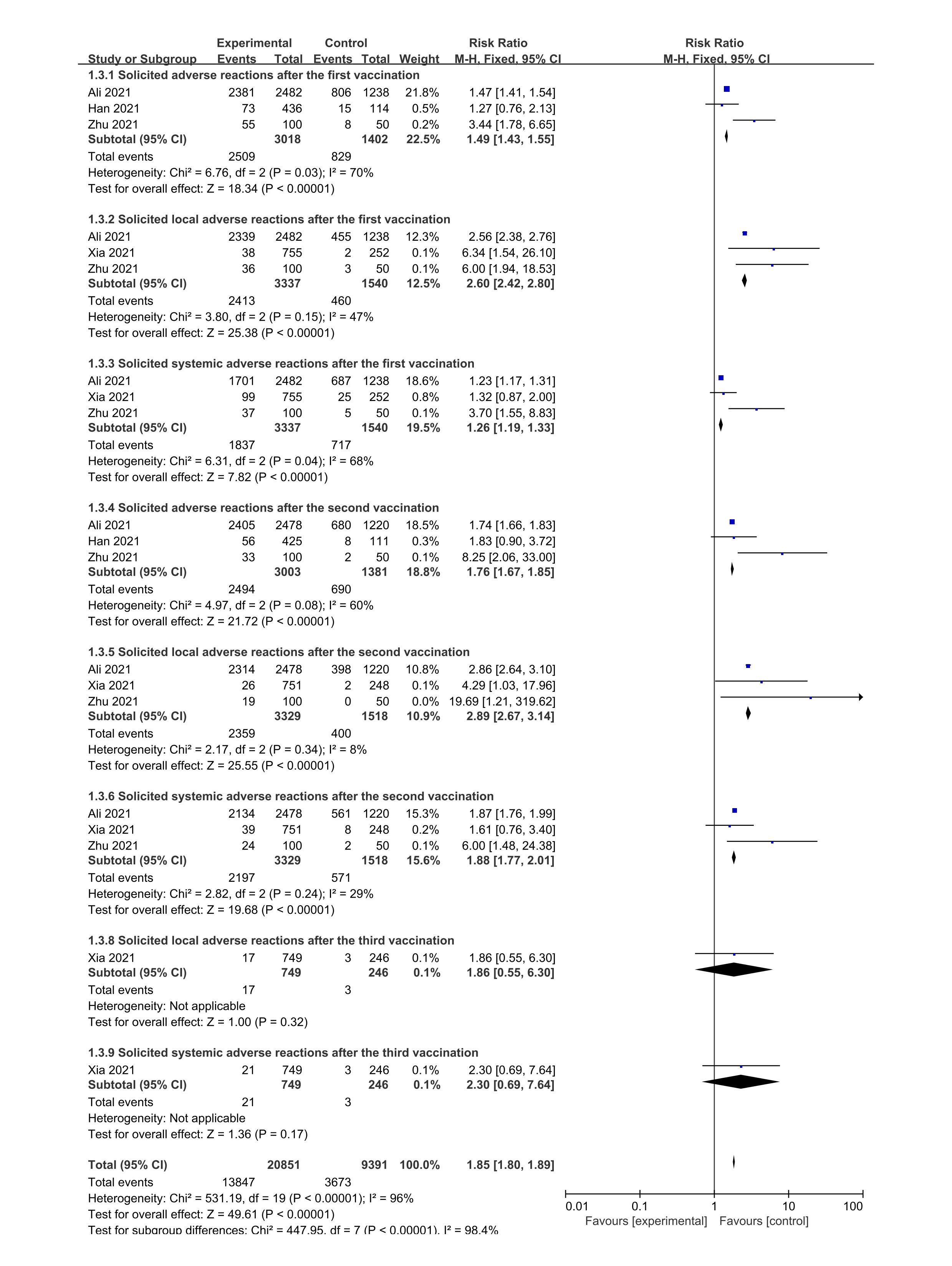

Supplement: Supplementary file 1 [file Data_Sheet_1.ZIP › Supplementary Material/Supplementary Figures (JPG)/Supplementary Figure 2. Total adverse reactions in vaccination group versus control group.jpg]

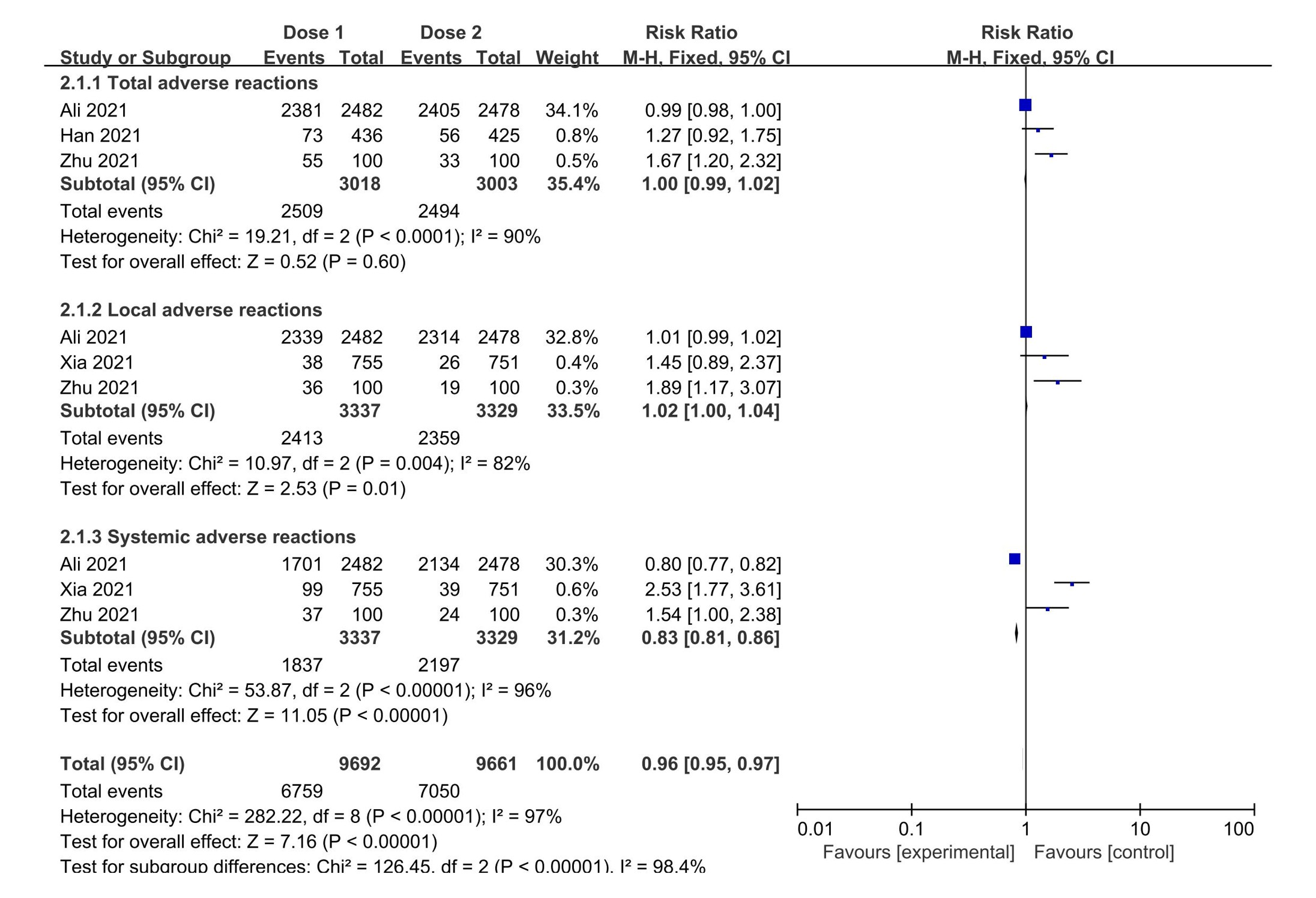

Supplement: Supplementary file 1 [file Data_Sheet_1.ZIP › Supplementary Material/Supplementary Figures (JPG)/Supplementary Figure 3. Total adverse reactions in vaccination group after dose 1 versus after dose 2.jpg]

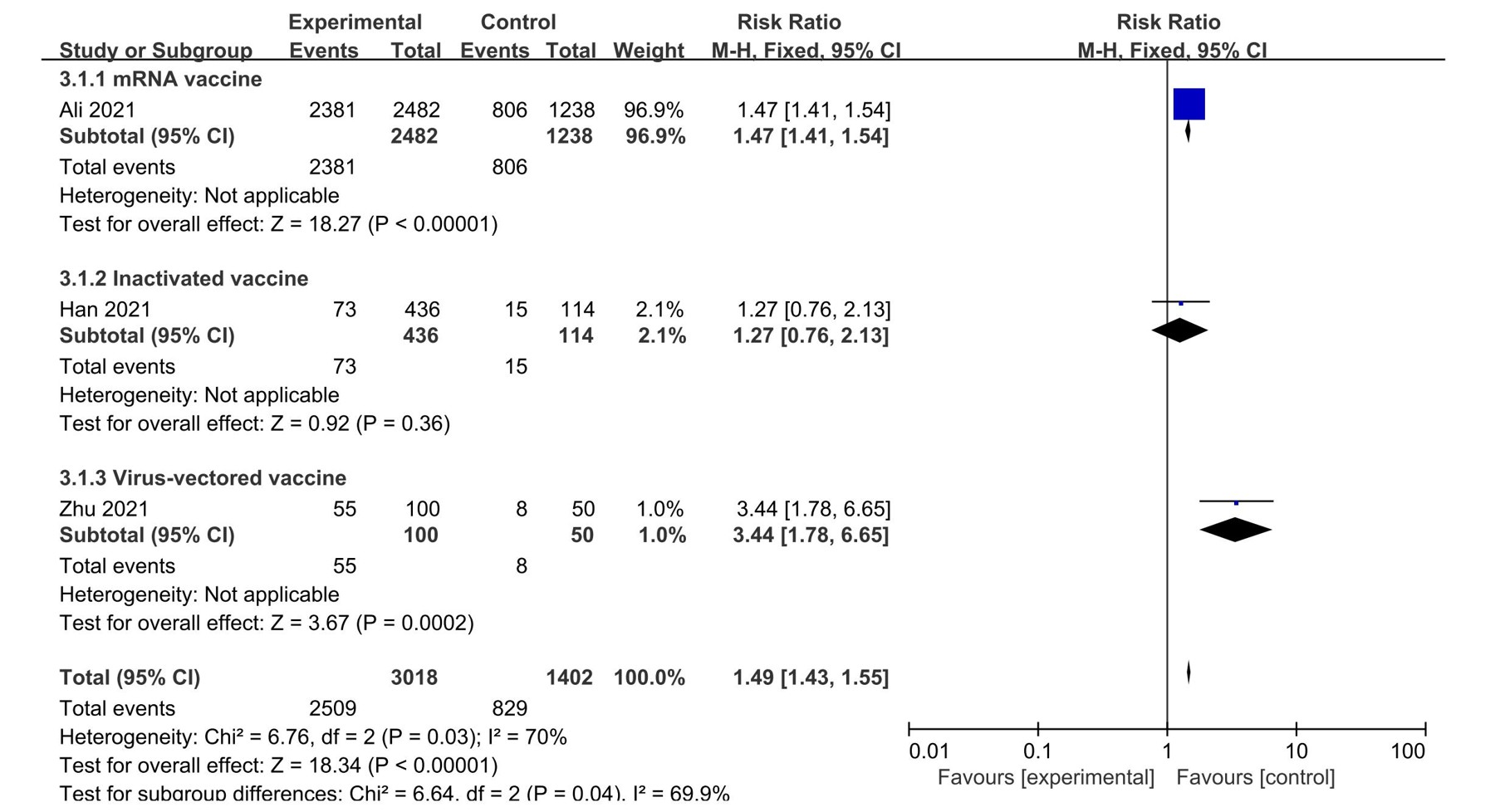

Supplement: Supplementary file 1 [file Data_Sheet_1.ZIP › Supplementary Material/Supplementary Figures (JPG)/Supplementary Figure 4. Adverse reactions among vaccination group versus control group/(A) Total adverse reactions after dose 1.jpg]

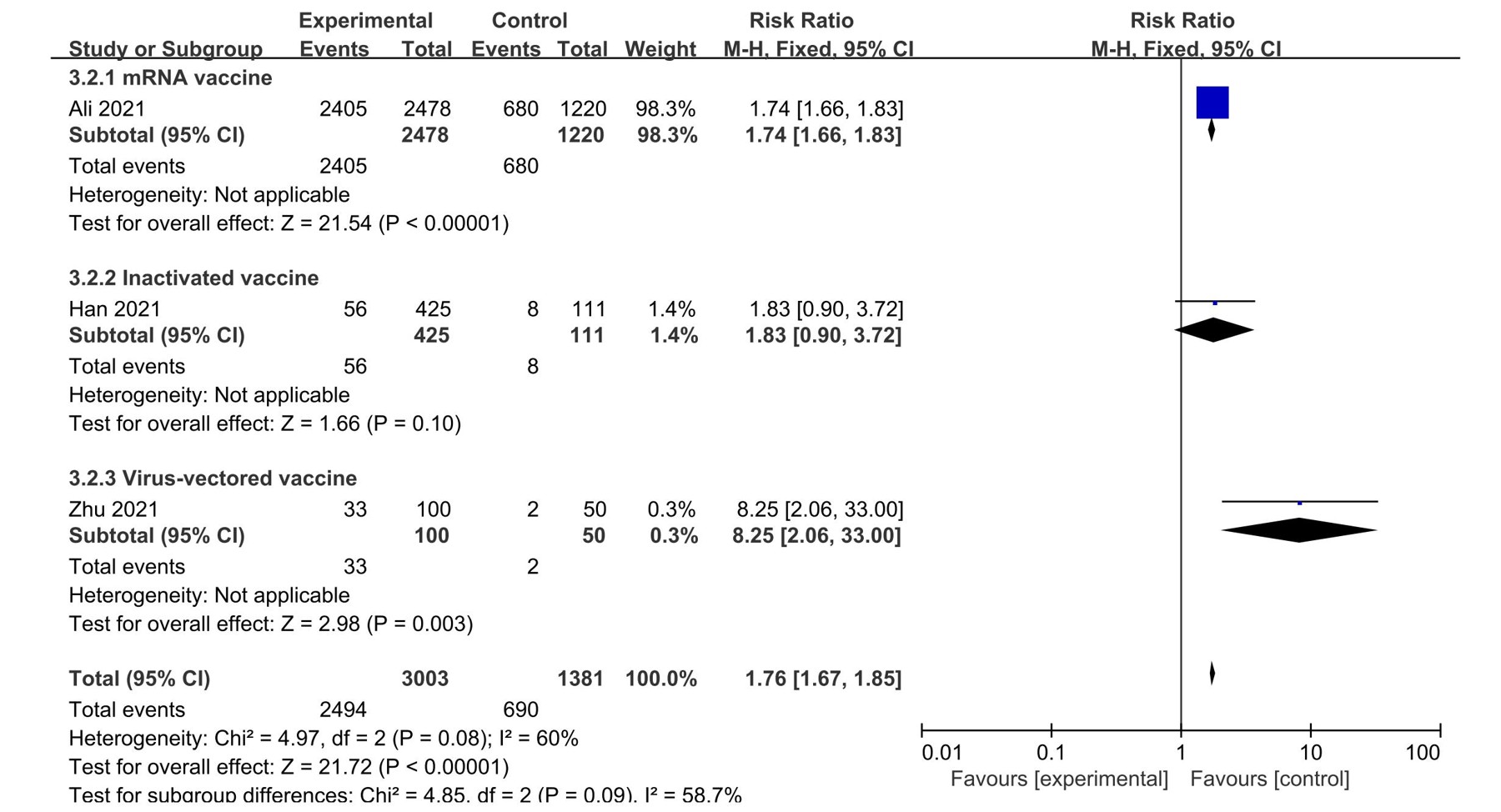

Supplement: Supplementary file 1 [file Data_Sheet_1.ZIP › Supplementary Material/Supplementary Figures (JPG)/Supplementary Figure 4. Adverse reactions among vaccination group versus control group/(B) Total adverse reactions after dose 2.jpg]

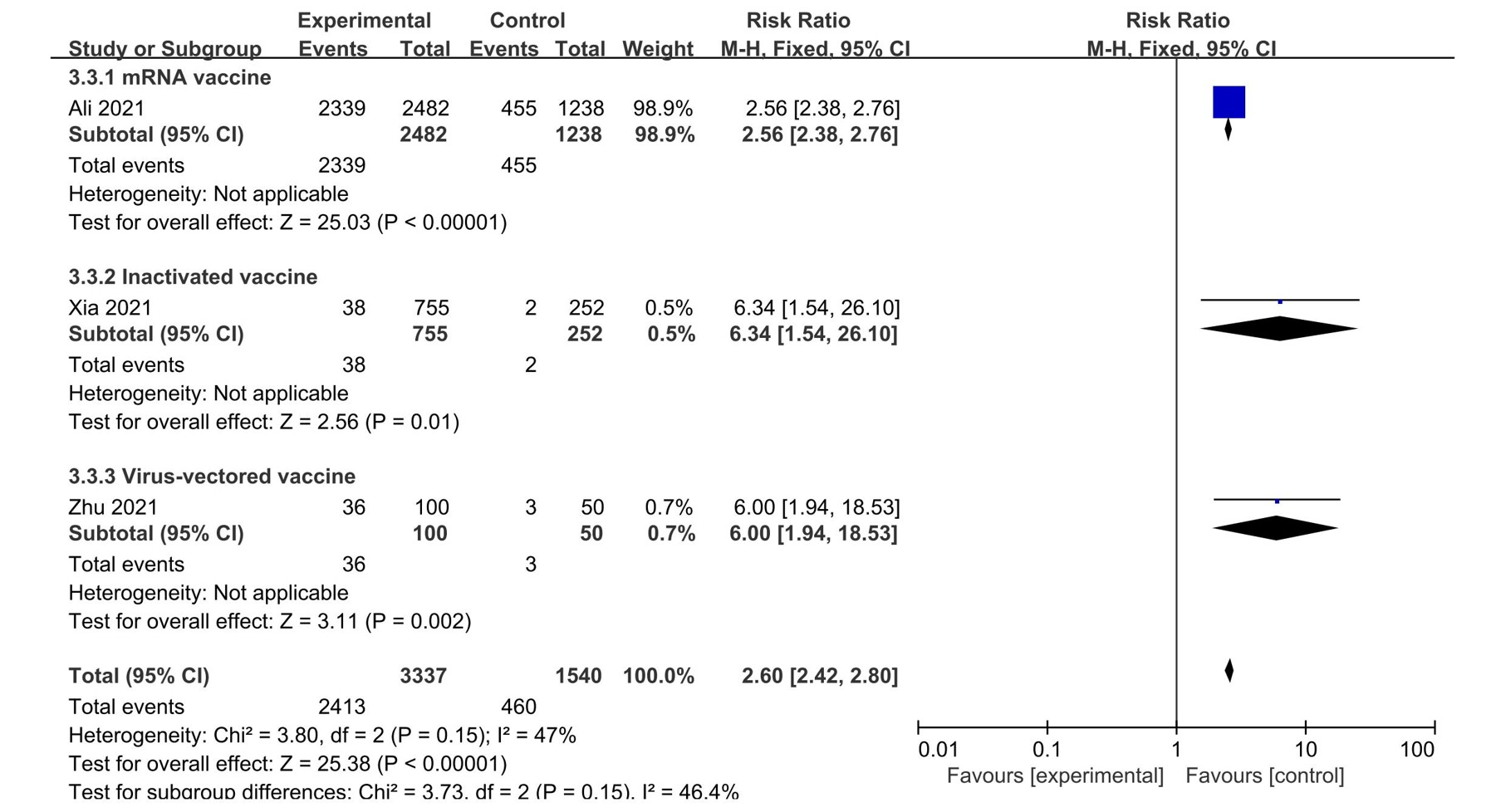

Supplement: Supplementary file 1 [file Data_Sheet_1.ZIP › Supplementary Material/Supplementary Figures (JPG)/Supplementary Figure 4. Adverse reactions among vaccination group versus control group/(C)Local adverse reactions after dose 1.jpg]

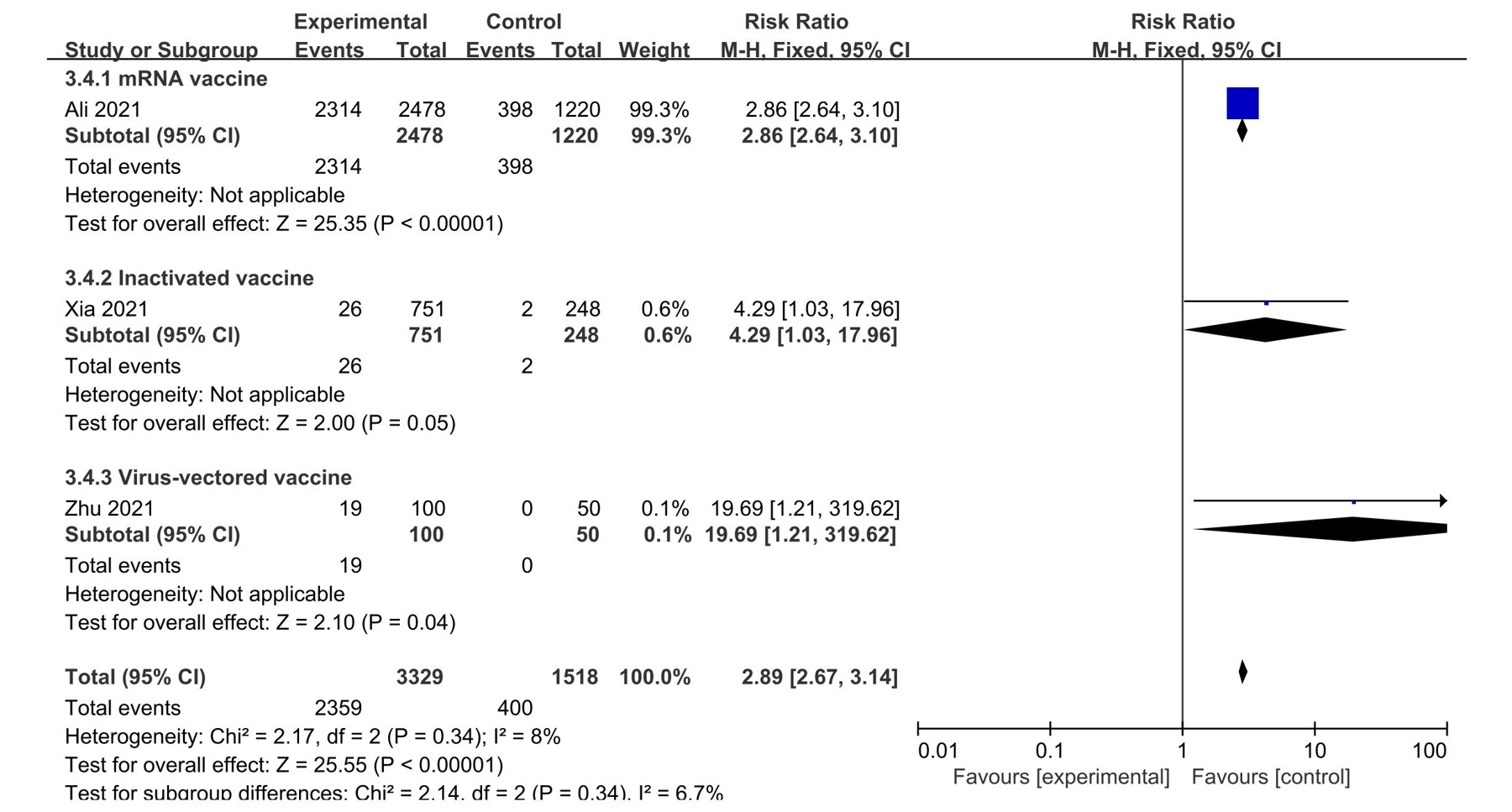

Supplement: Supplementary file 1 [file Data_Sheet_1.ZIP › Supplementary Material/Supplementary Figures (JPG)/Supplementary Figure 4. Adverse reactions among vaccination group versus control group/(D) Local adverse reactions after dose 2.jpg]

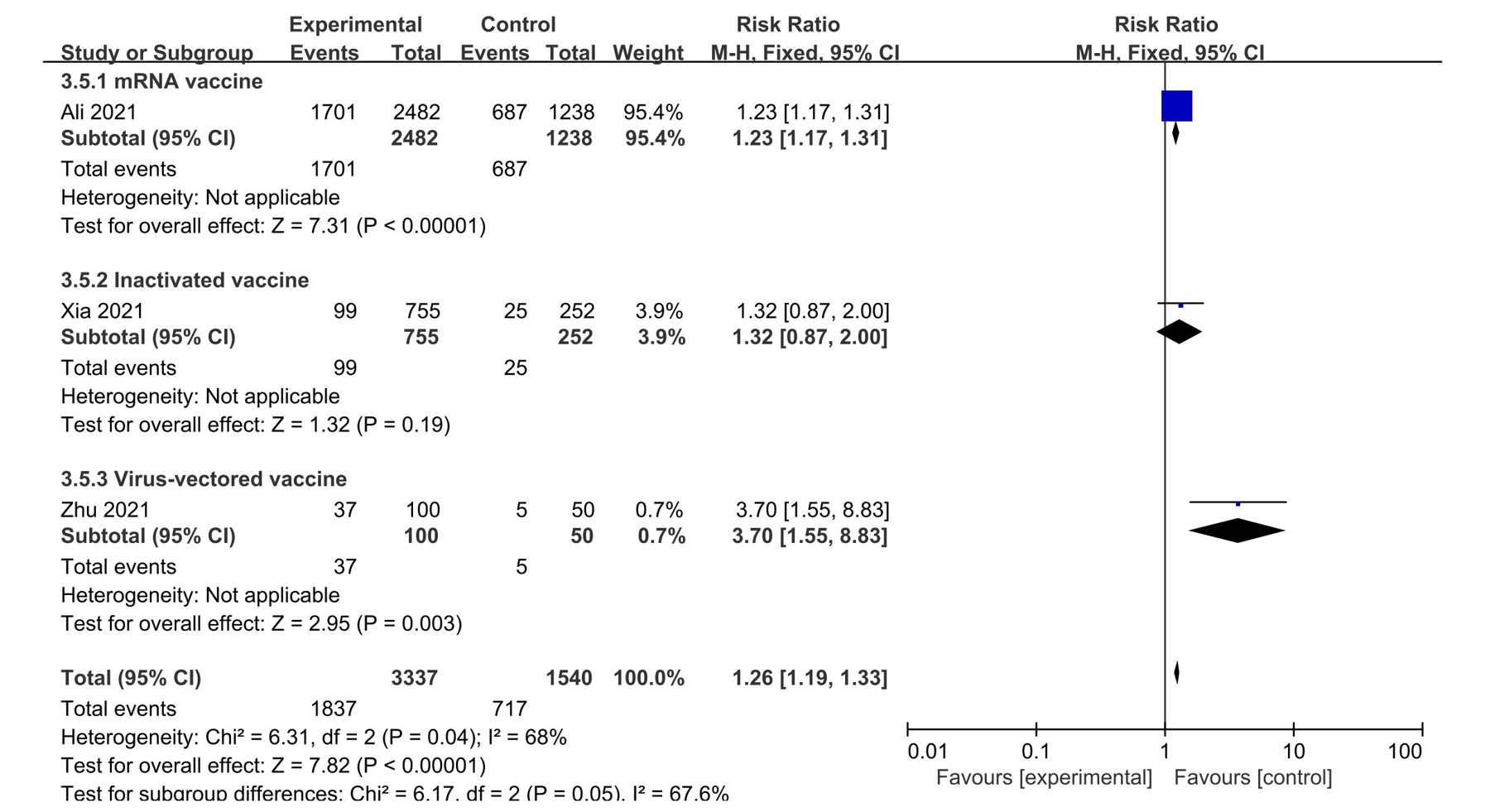

Supplement: Supplementary file 1 [file Data_Sheet_1.ZIP › Supplementary Material/Supplementary Figures (JPG)/Supplementary Figure 4. Adverse reactions among vaccination group versus control group/(E) Systemic adverse reactions after dose 1.jpg]

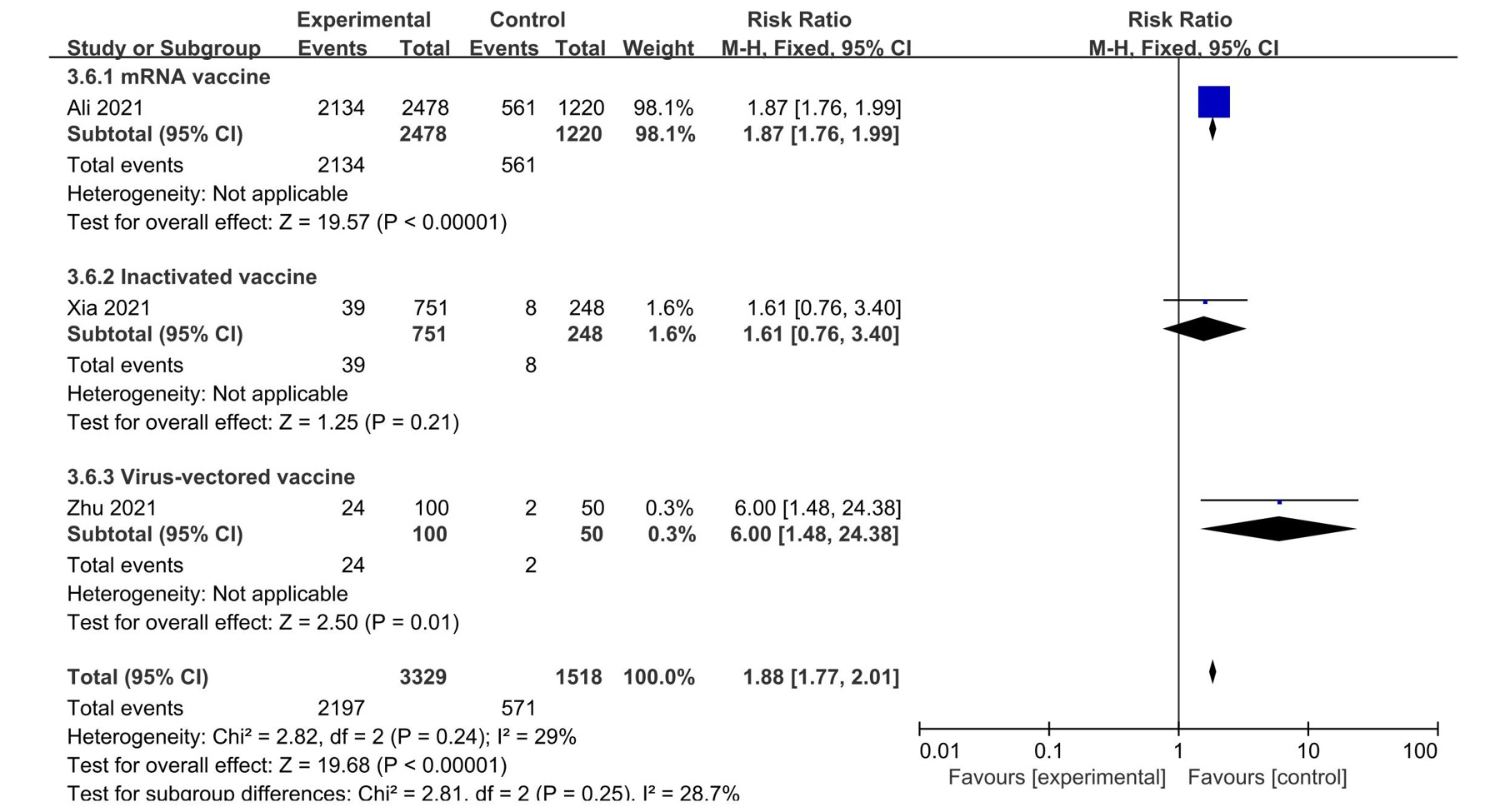

Supplement: Supplementary file 1 [file Data_Sheet_1.ZIP › Supplementary Material/Supplementary Figures (JPG)/Supplementary Figure 4. Adverse reactions among vaccination group versus control group/(F) Systemic adverse reactions after dose 2.jpg]

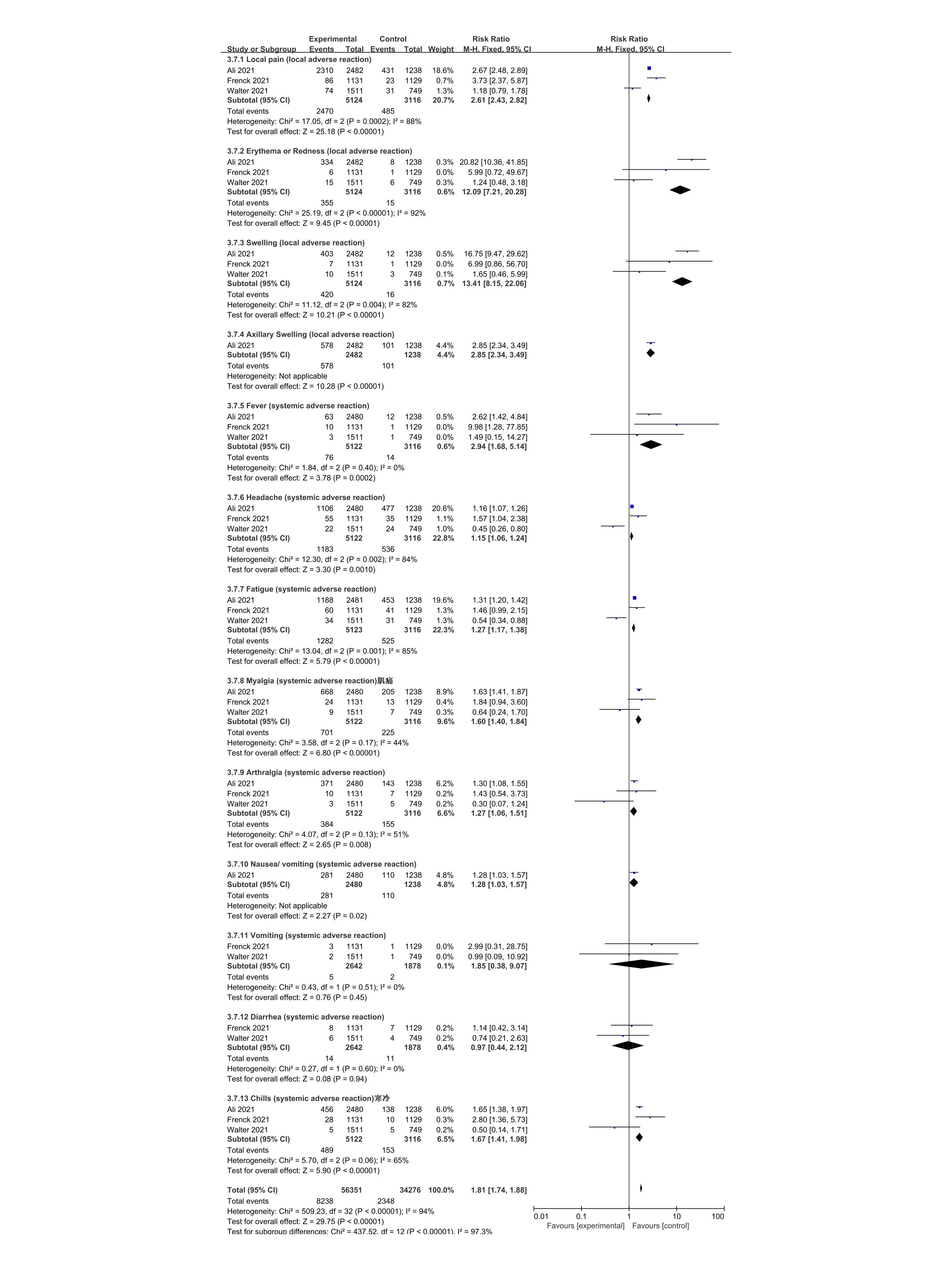

Supplement: Supplementary file 1 [file Data_Sheet_1.ZIP › Supplementary Material/Supplementary Figures (JPG)/Supplementary Figure 5. Specific adverse reactions in mRNA vaccine group versus control group/(A) After dose 1.jpg]

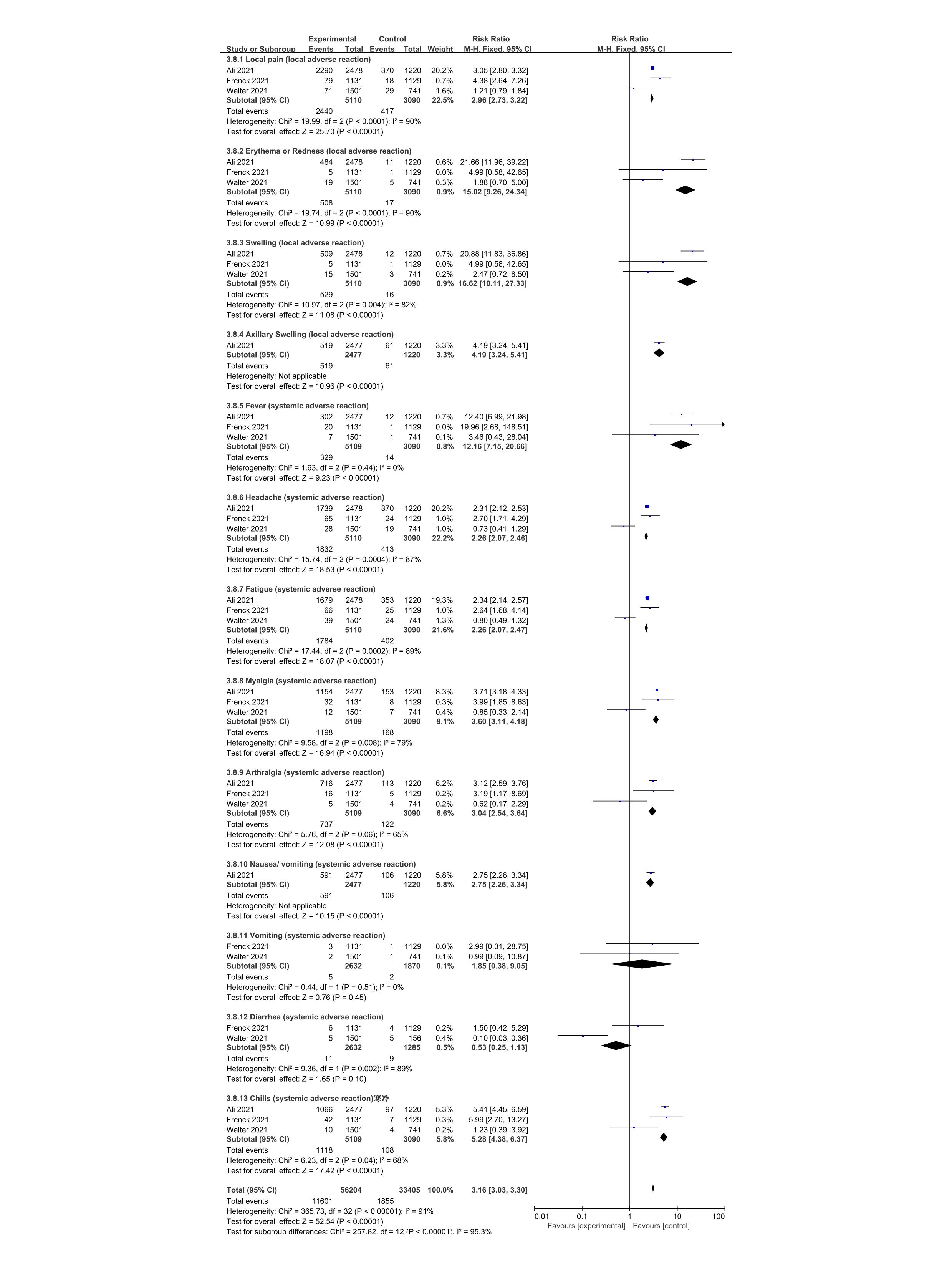

Supplement: Supplementary file 1 [file Data_Sheet_1.ZIP › Supplementary Material/Supplementary Figures (JPG)/Supplementary Figure 5. Specific adverse reactions in mRNA vaccine group versus control group/(B) After dose 2.jpg]

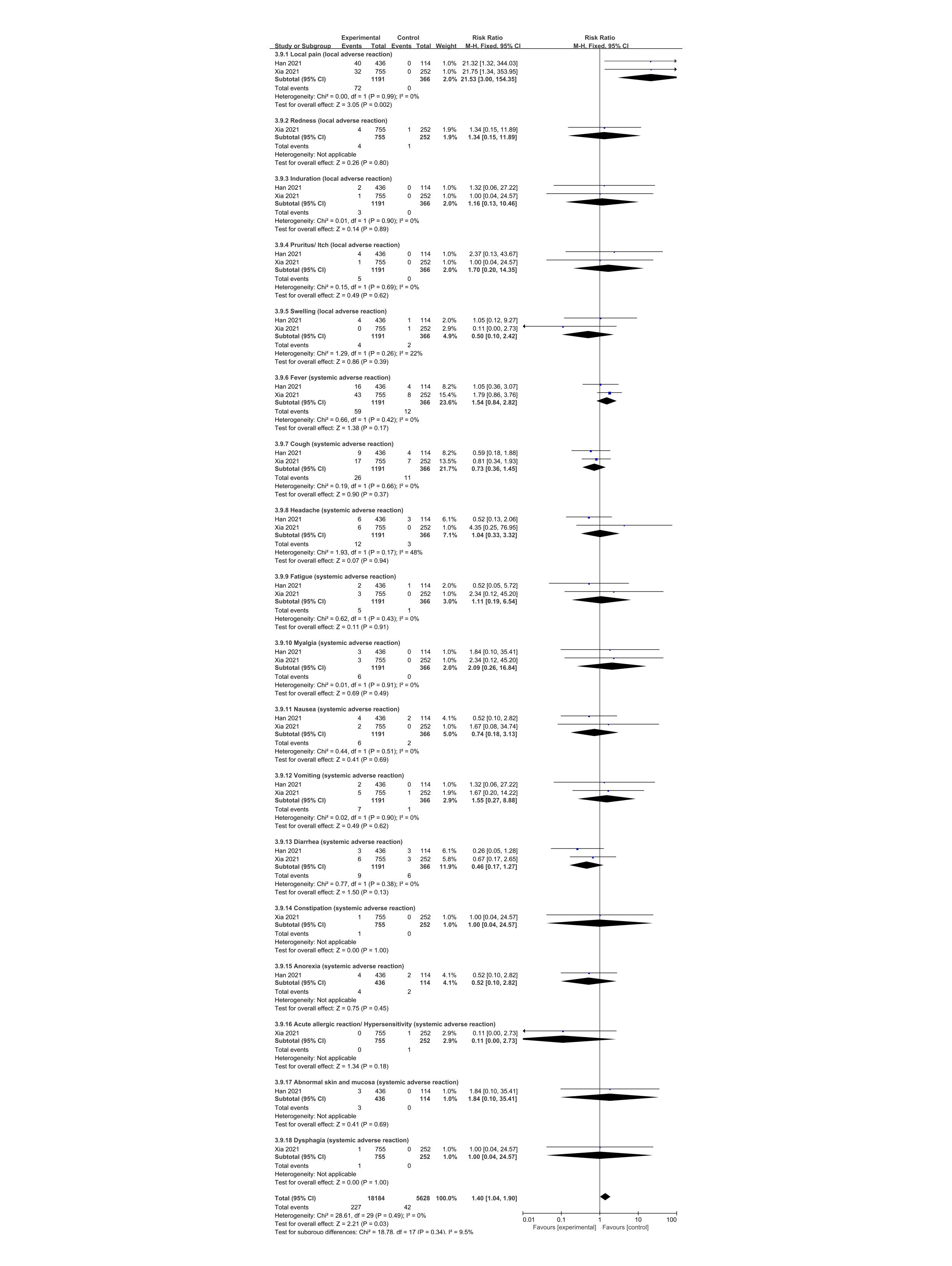

Supplement: Supplementary file 1 [file Data_Sheet_1.ZIP › Supplementary Material/Supplementary Figures (JPG)/Supplementary Figure 6. Specific adverse reactions in inactivated vaccine group versus control group/(A) After dose 1.jpg]

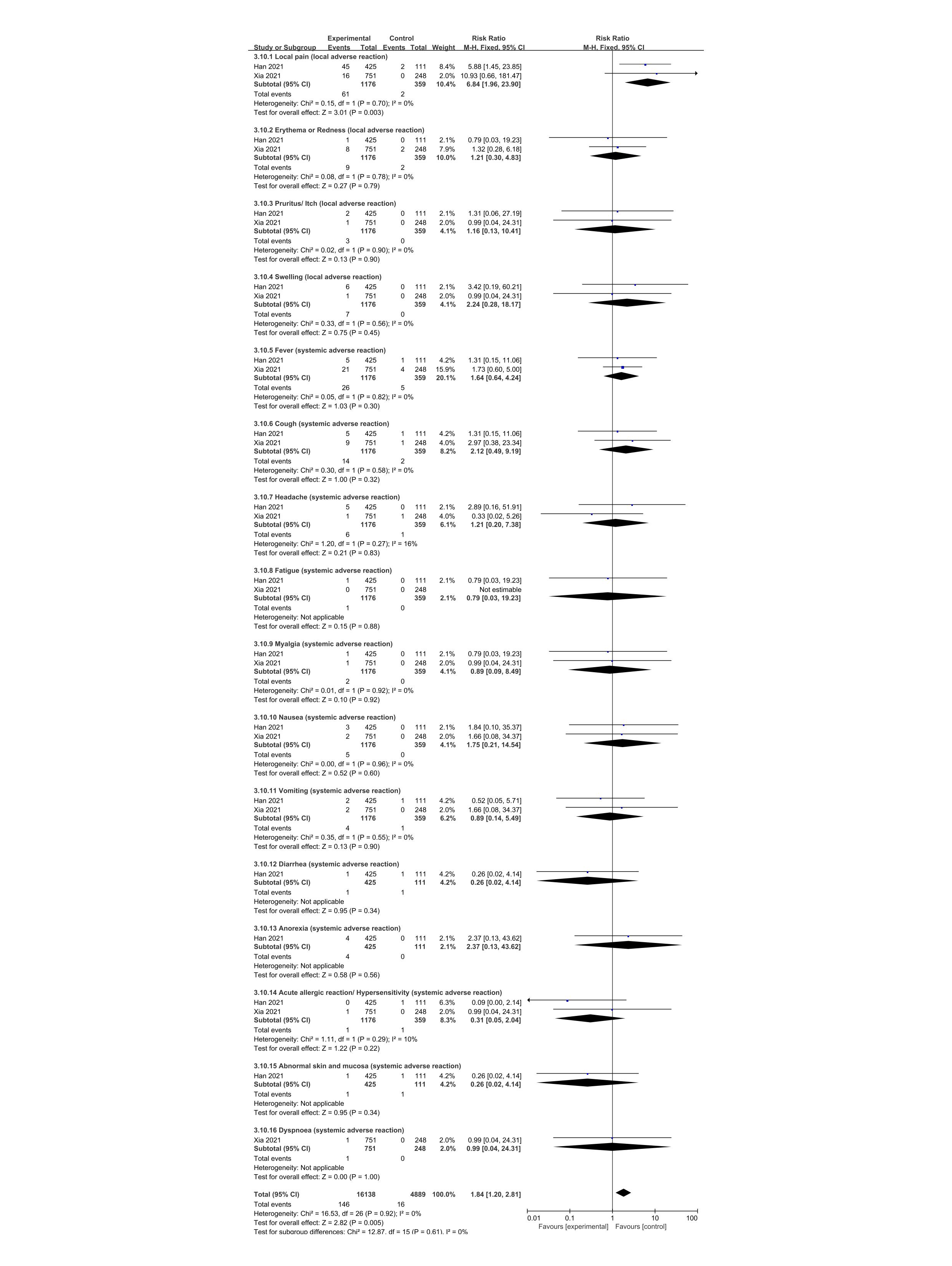

Supplement: Supplementary file 1 [file Data_Sheet_1.ZIP › Supplementary Material/Supplementary Figures (JPG)/Supplementary Figure 6. Specific adverse reactions in inactivated vaccine group versus control group/(B) After dose 2.jpg]

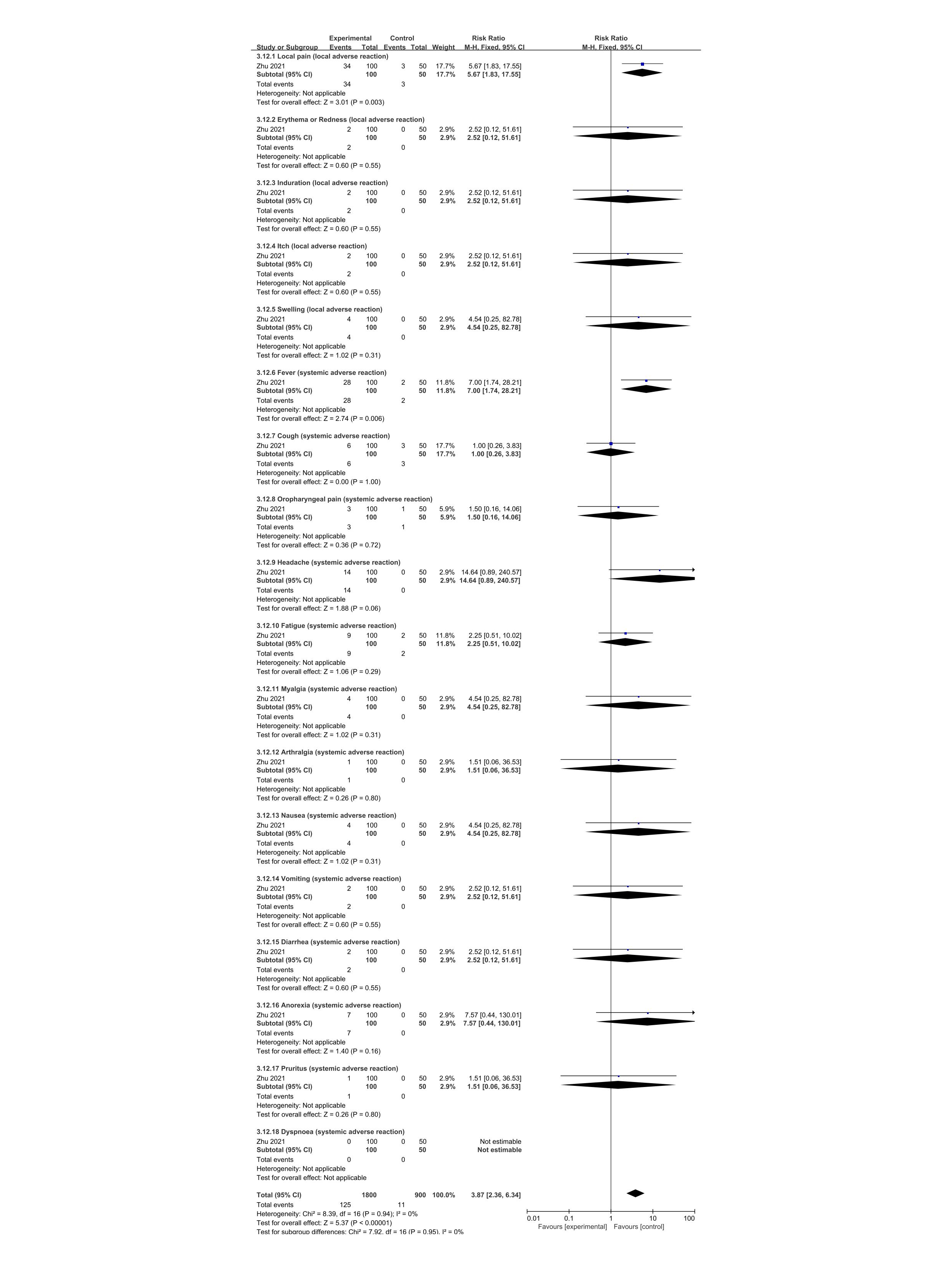

Supplement: Supplementary file 1 [file Data_Sheet_1.ZIP › Supplementary Material/Supplementary Figures (JPG)/Supplementary Figure 7. Specific adverse reactions in vectored vaccine group versus control group/(A) After dose 1.jpg]

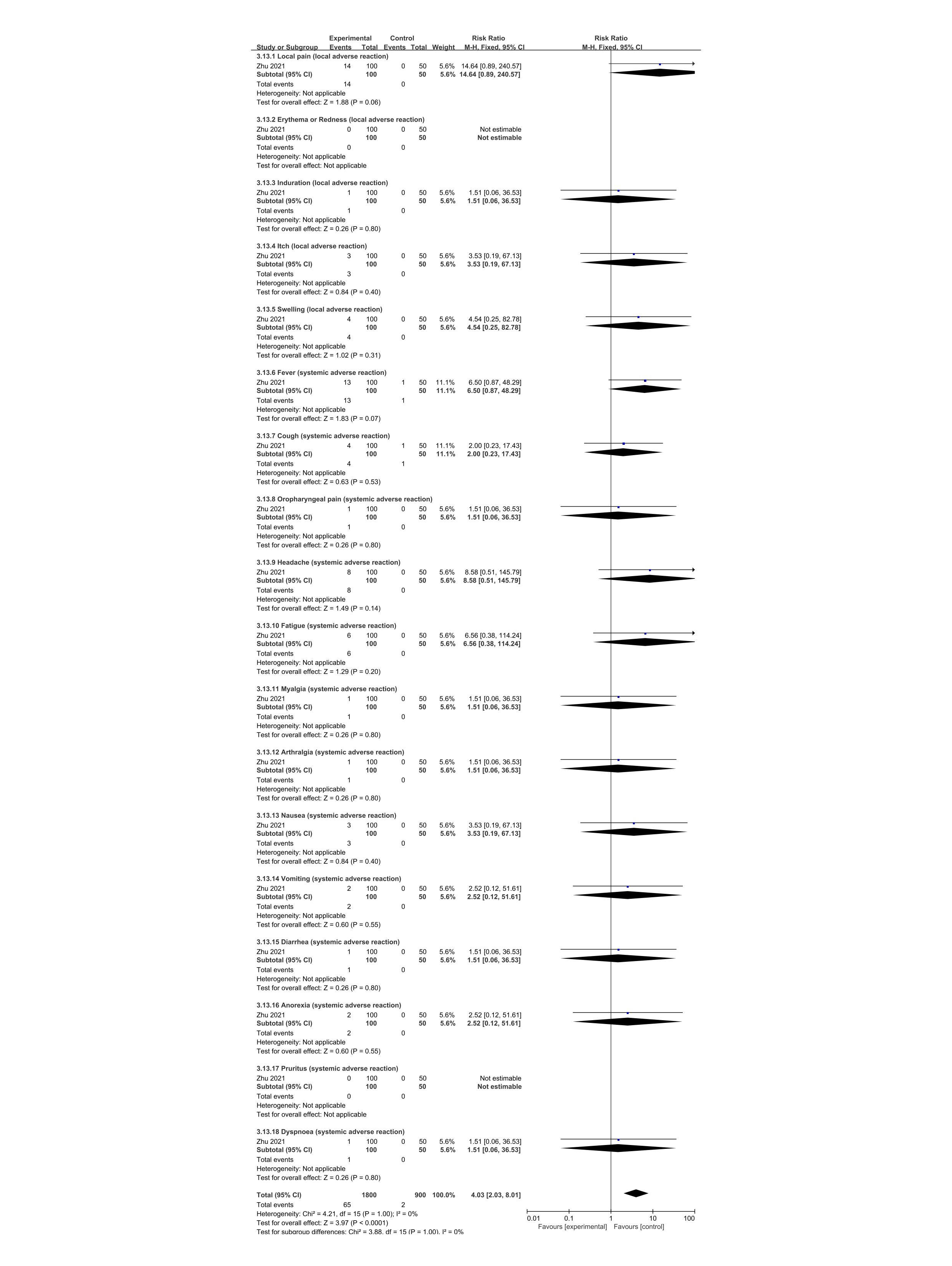

Supplement: Supplementary file 1 [file Data_Sheet_1.ZIP › Supplementary Material/Supplementary Figures (JPG)/Supplementary Figure 7. Specific adverse reactions in vectored vaccine group versus control group/(B) After dose 2.jpg]

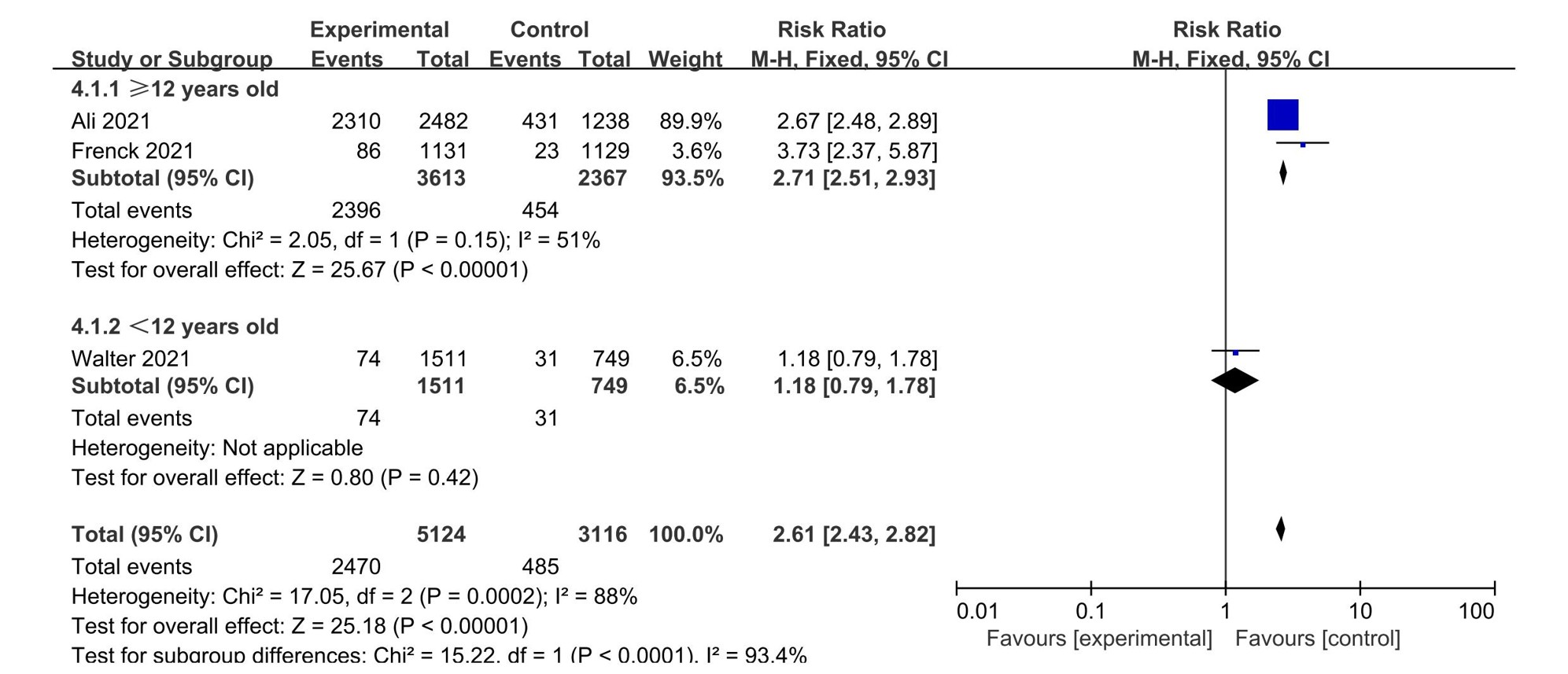

Supplement: Supplementary file 1 [file Data_Sheet_1.ZIP › Supplementary Material/Supplementary Figures (JPG)/Supplementary Figure 8. Adverse reactions in mRNA vaccine group of different ages versus control group/(A) Local pain after the first vaccination.jpg]

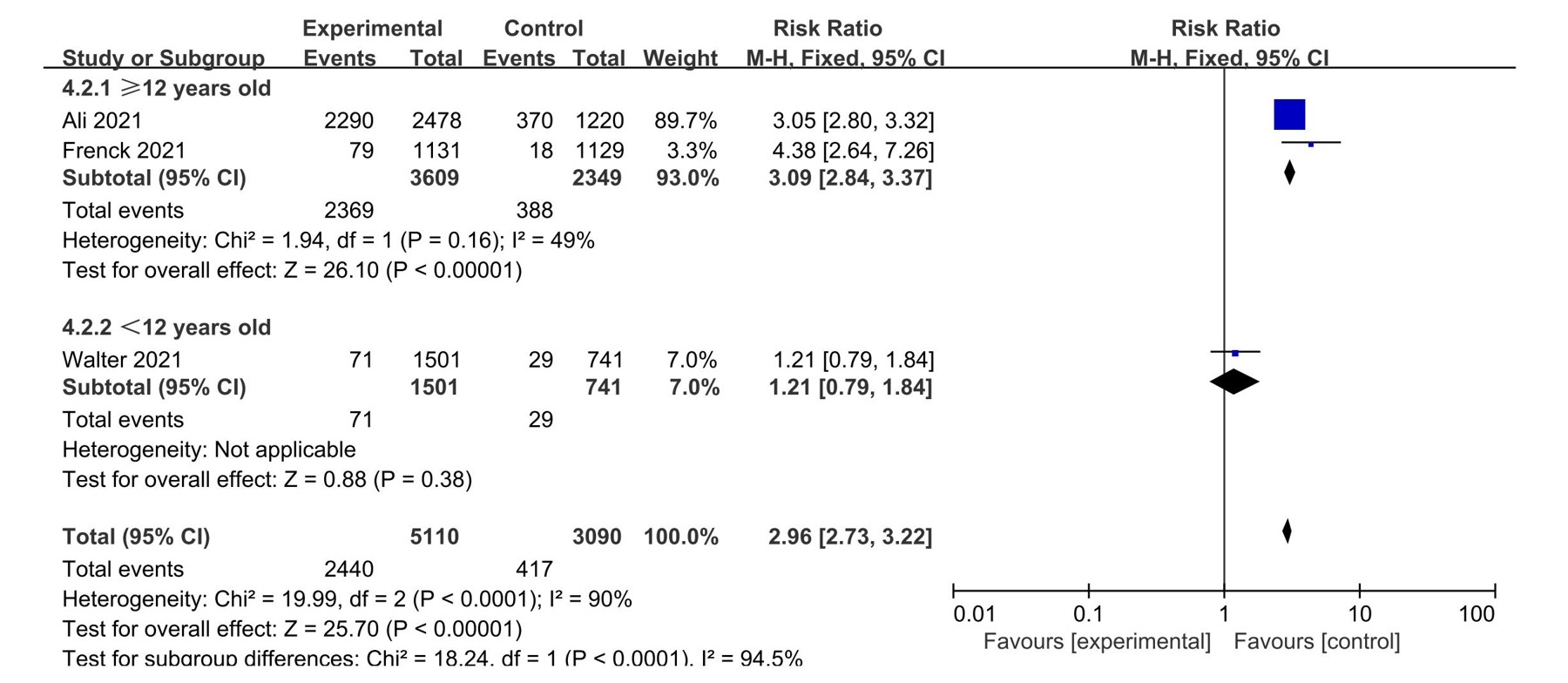

Supplement: Supplementary file 1 [file Data_Sheet_1.ZIP › Supplementary Material/Supplementary Figures (JPG)/Supplementary Figure 8. Adverse reactions in mRNA vaccine group of different ages versus control group/(B) Local pain after the second vaccination.jpg]

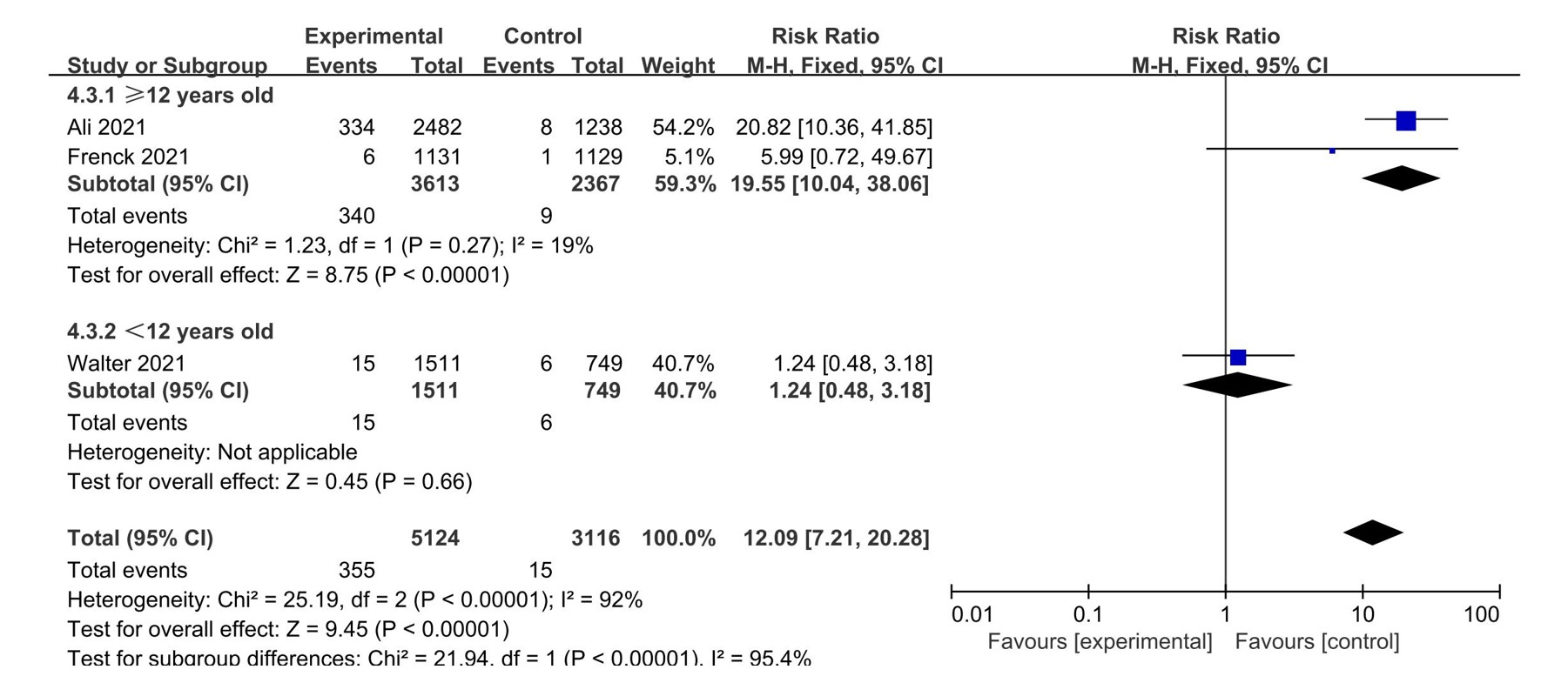

Supplement: Supplementary file 1 [file Data_Sheet_1.ZIP › Supplementary Material/Supplementary Figures (JPG)/Supplementary Figure 8. Adverse reactions in mRNA vaccine group of different ages versus control group/(C) Erythema or Redness after the first vaccination.jpg]

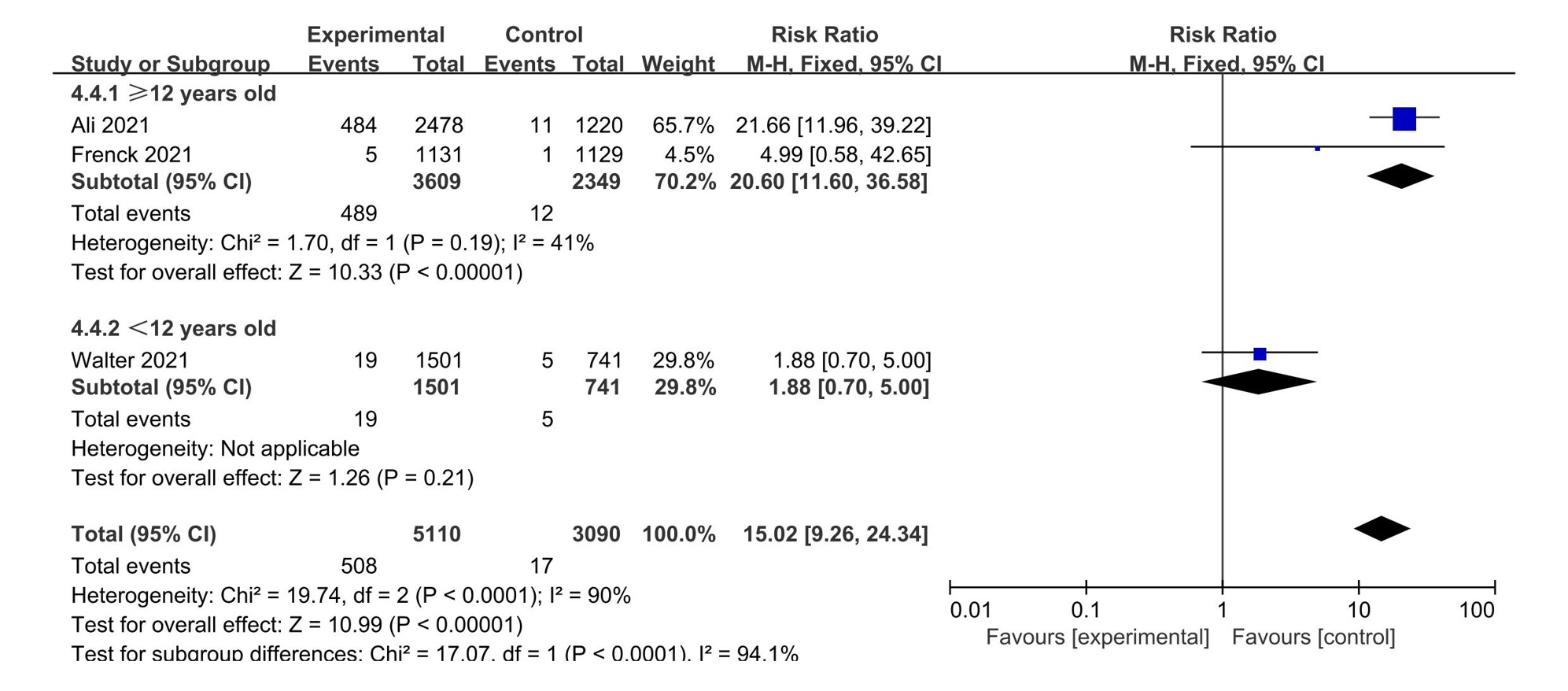

Supplement: Supplementary file 1 [file Data_Sheet_1.ZIP › Supplementary Material/Supplementary Figures (JPG)/Supplementary Figure 8. Adverse reactions in mRNA vaccine group of different ages versus control group/(D) Erythema or Redness after the second vaccination.jpg]

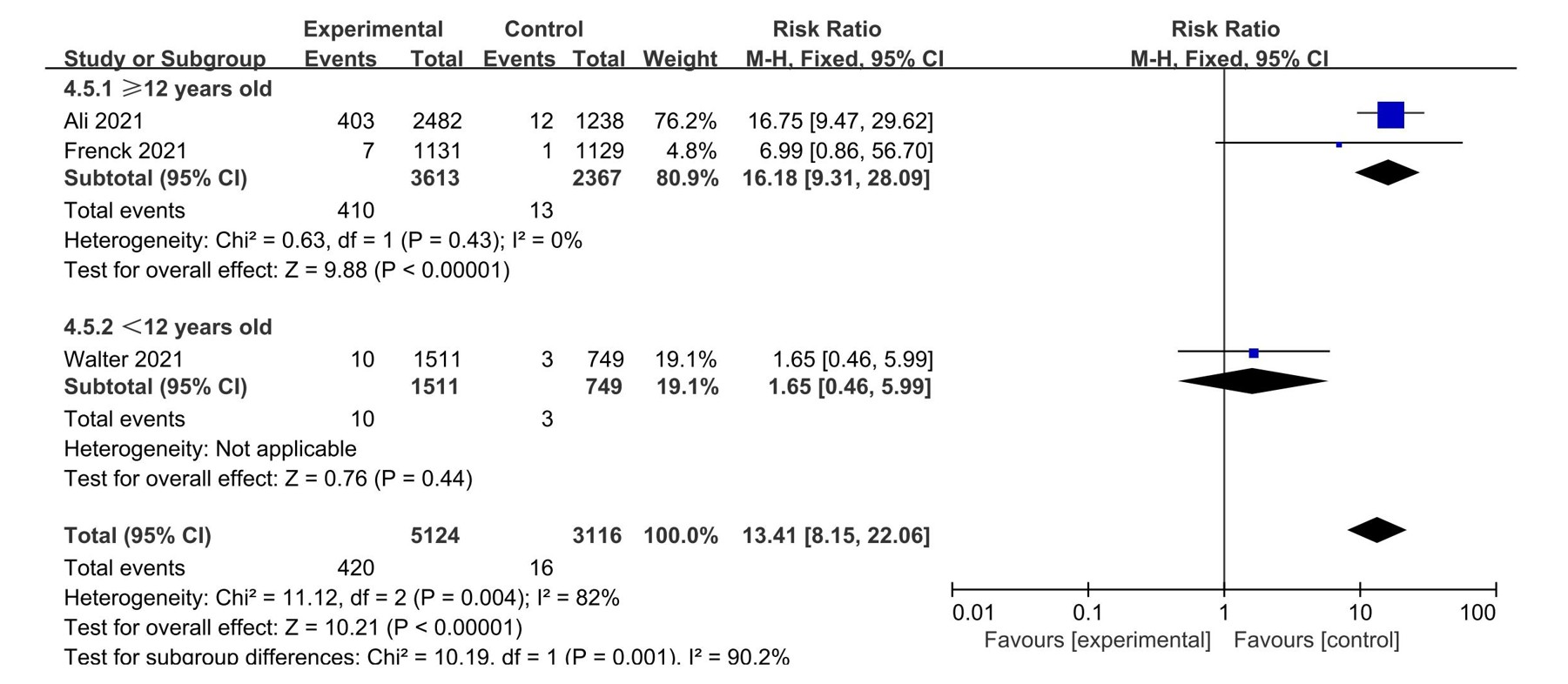

Supplement: Supplementary file 1 [file Data_Sheet_1.ZIP › Supplementary Material/Supplementary Figures (JPG)/Supplementary Figure 8. Adverse reactions in mRNA vaccine group of different ages versus control group/(E) Swelling after the first vaccination.jpg]

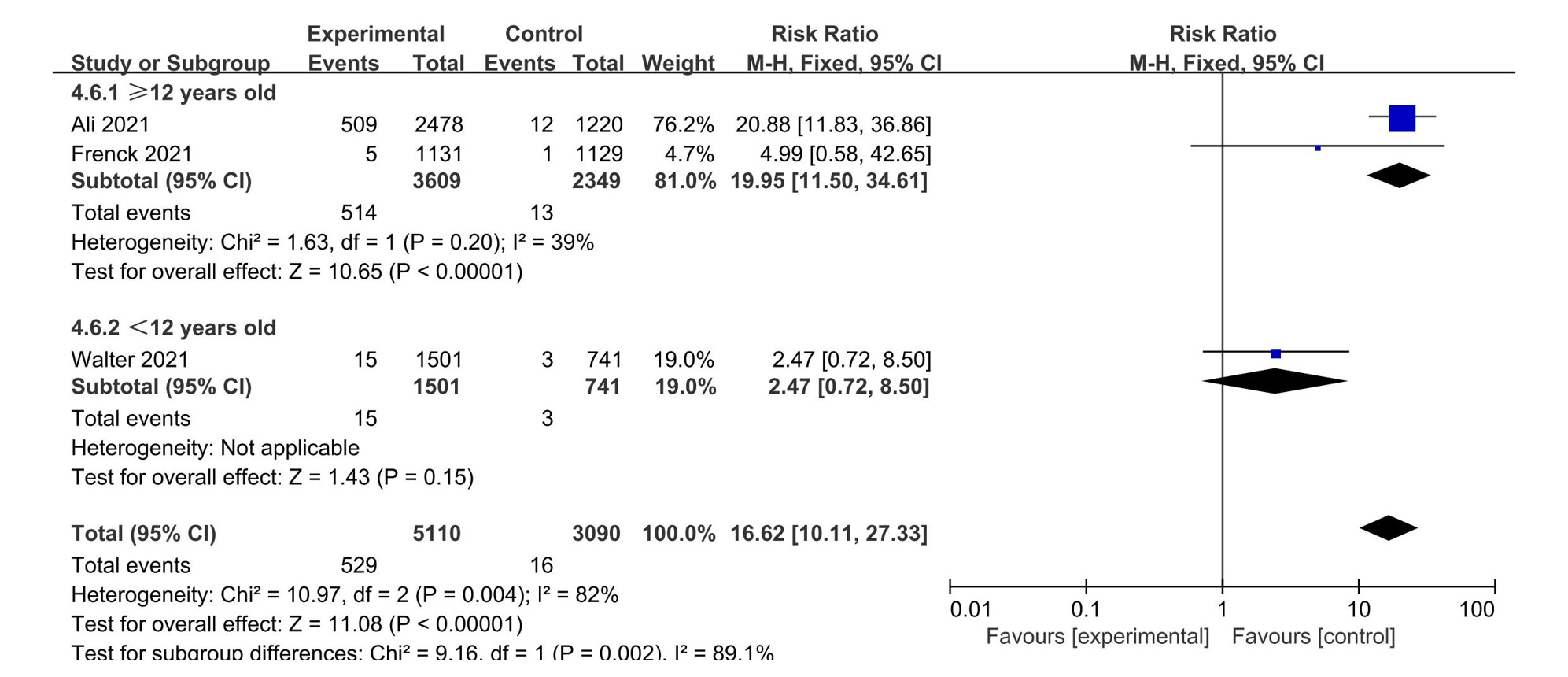

Supplement: Supplementary file 1 [file Data_Sheet_1.ZIP › Supplementary Material/Supplementary Figures (JPG)/Supplementary Figure 8. Adverse reactions in mRNA vaccine group of different ages versus control group/(F) Swelling after the second vaccination.jpg]

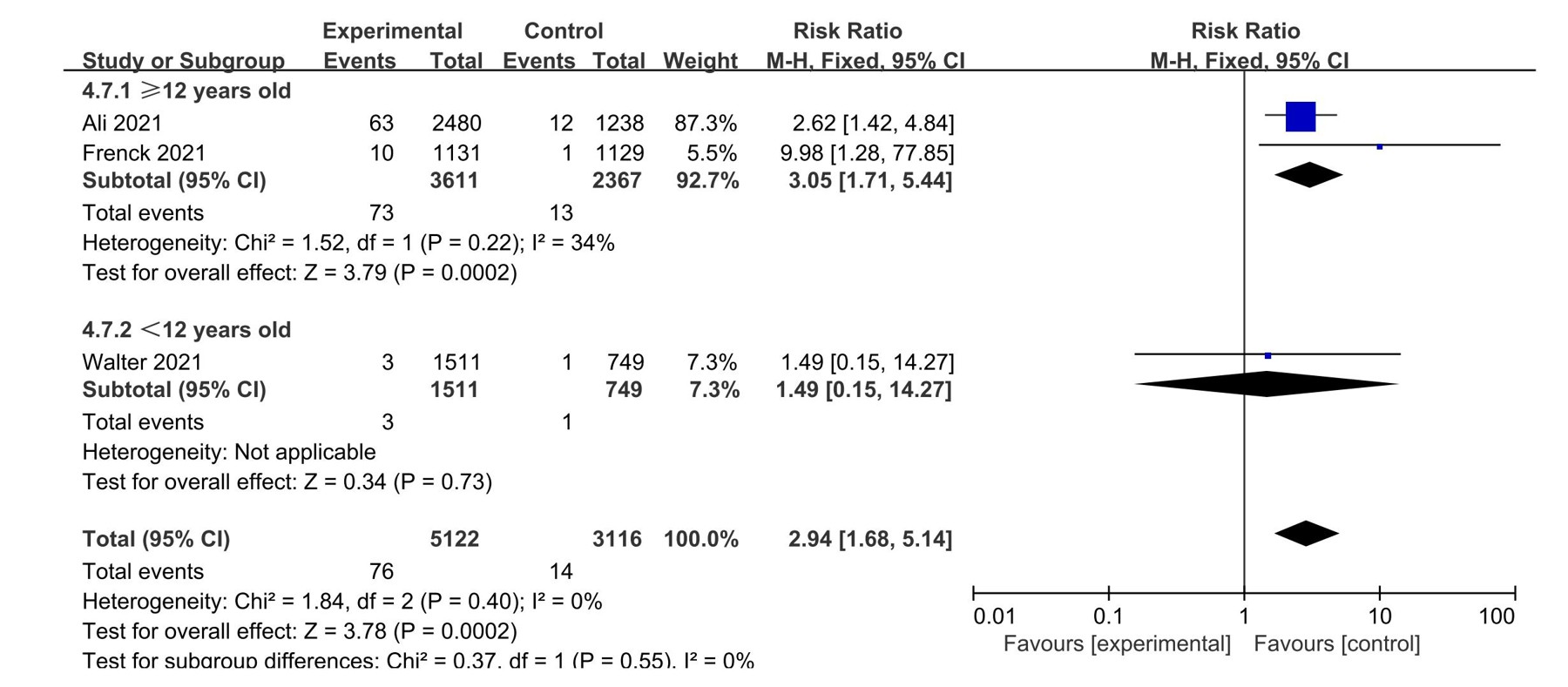

Supplement: Supplementary file 1 [file Data_Sheet_1.ZIP › Supplementary Material/Supplementary Figures (JPG)/Supplementary Figure 8. Adverse reactions in mRNA vaccine group of different ages versus control group/(G) Fever after the first vaccination.jpg]

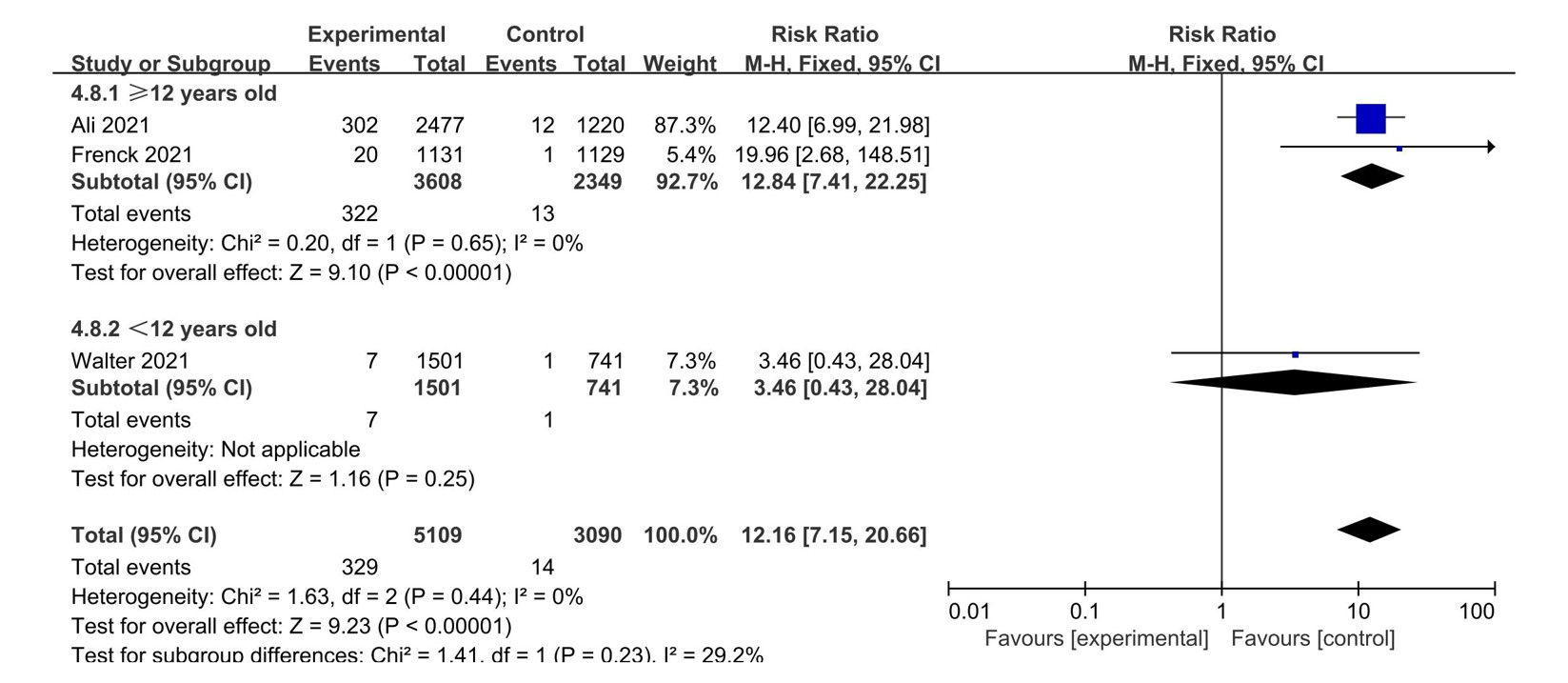

Supplement: Supplementary file 1 [file Data_Sheet_1.ZIP › Supplementary Material/Supplementary Figures (JPG)/Supplementary Figure 8. Adverse reactions in mRNA vaccine group of different ages versus control group/(H) Fever after the second vaccination.jpg]

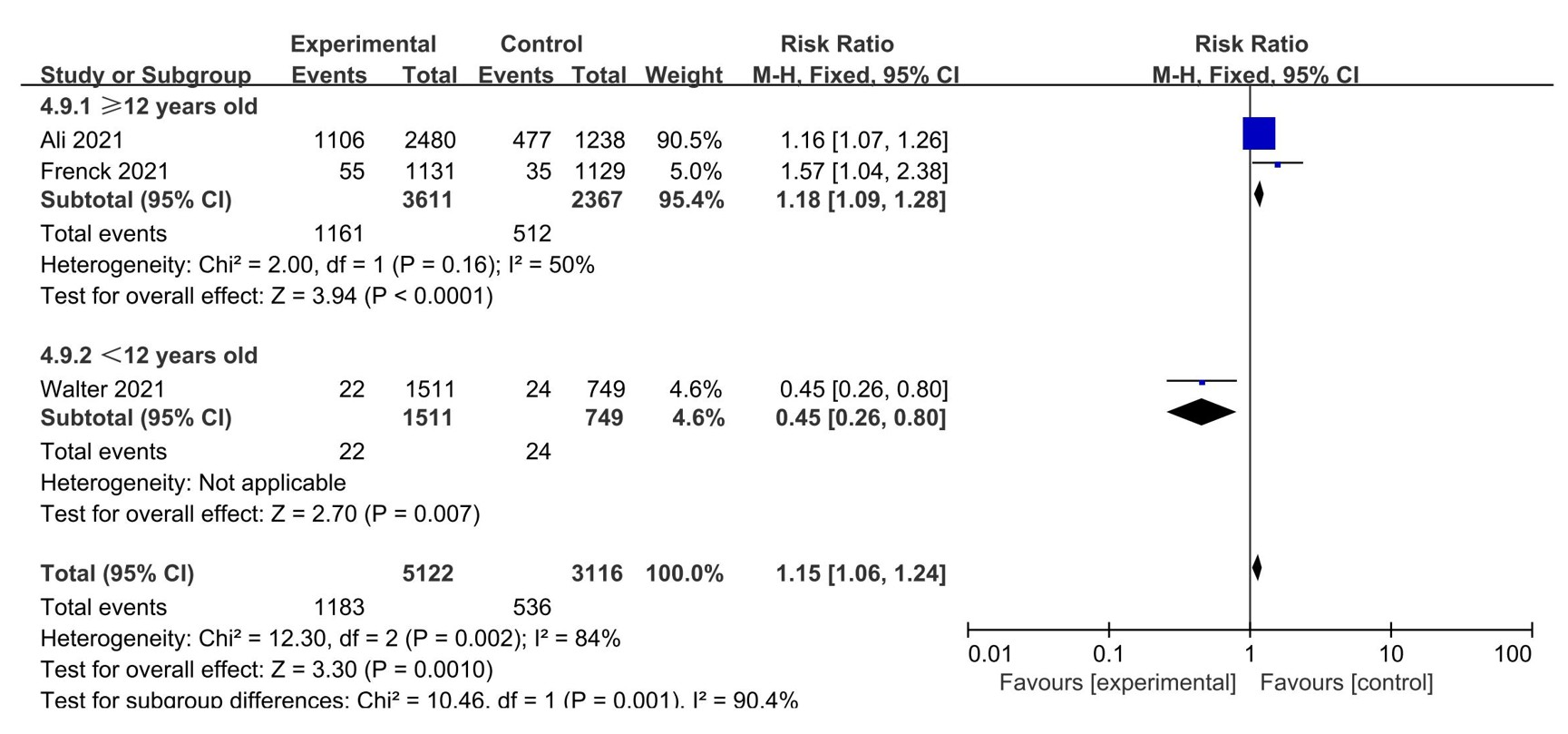

Supplement: Supplementary file 1 [file Data_Sheet_1.ZIP › Supplementary Material/Supplementary Figures (JPG)/Supplementary Figure 8. Adverse reactions in mRNA vaccine group of different ages versus control group/(I) Headache after the first vaccination.jpg]

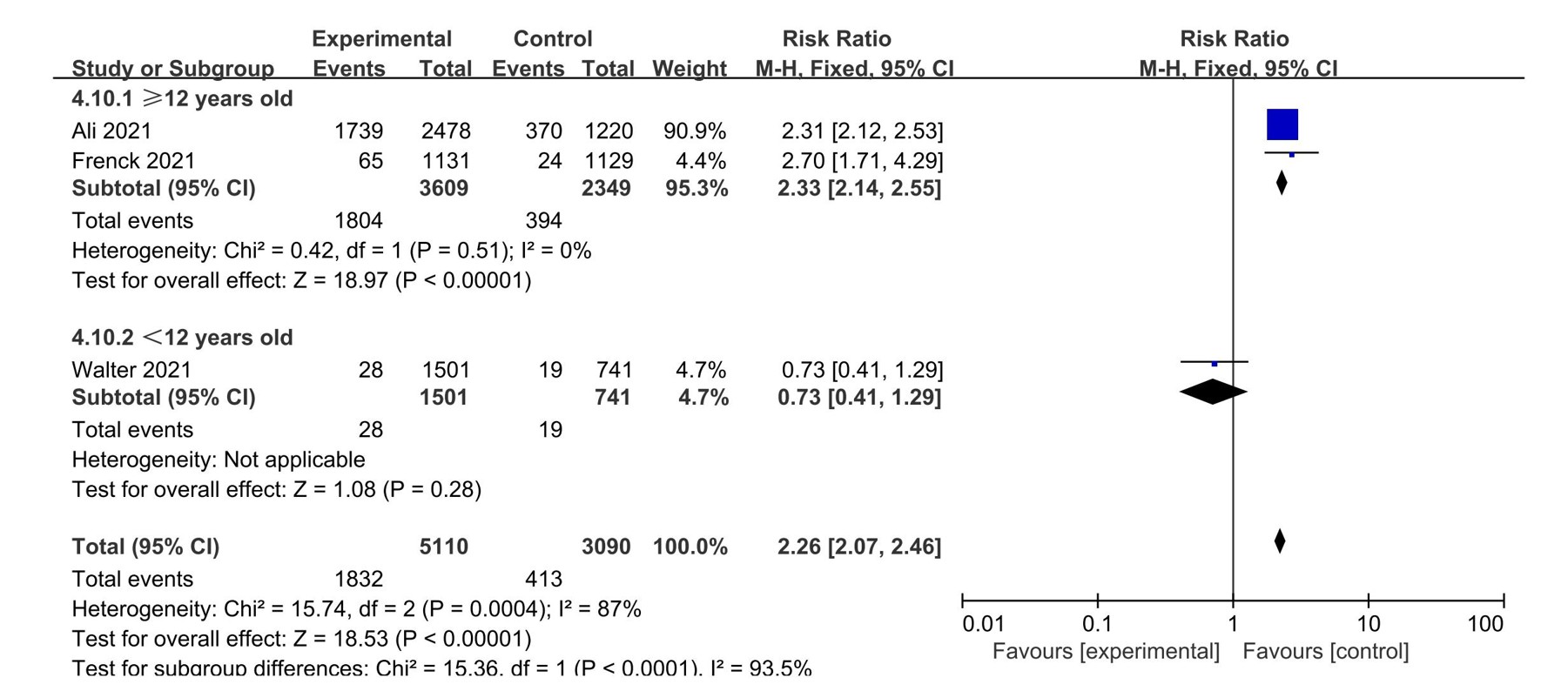

Supplement: Supplementary file 1 [file Data_Sheet_1.ZIP › Supplementary Material/Supplementary Figures (JPG)/Supplementary Figure 8. Adverse reactions in mRNA vaccine group of different ages versus control group/(J) Headache after the second vaccination.jpg]

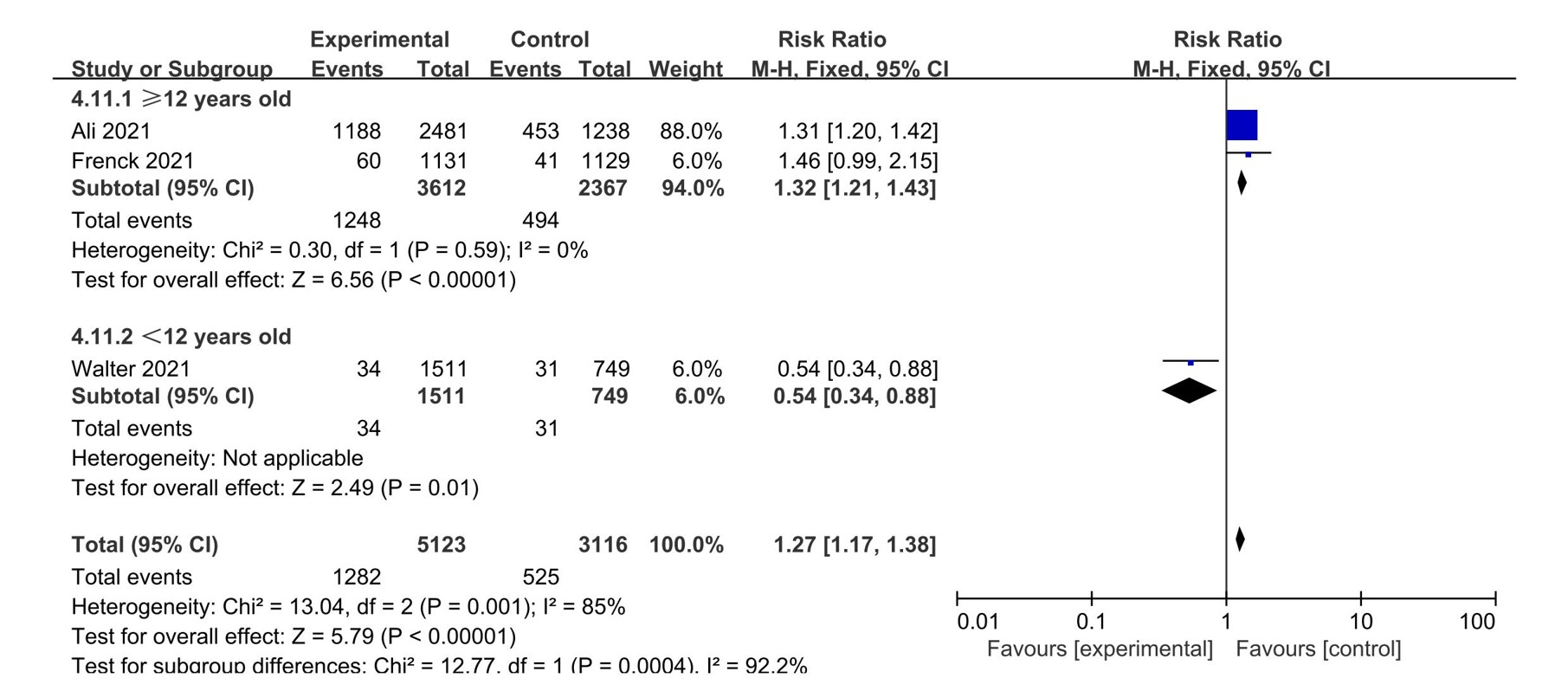

Supplement: Supplementary file 1 [file Data_Sheet_1.ZIP › Supplementary Material/Supplementary Figures (JPG)/Supplementary Figure 8. Adverse reactions in mRNA vaccine group of different ages versus control group/(K) Fatigue after the first vaccination.jpg]

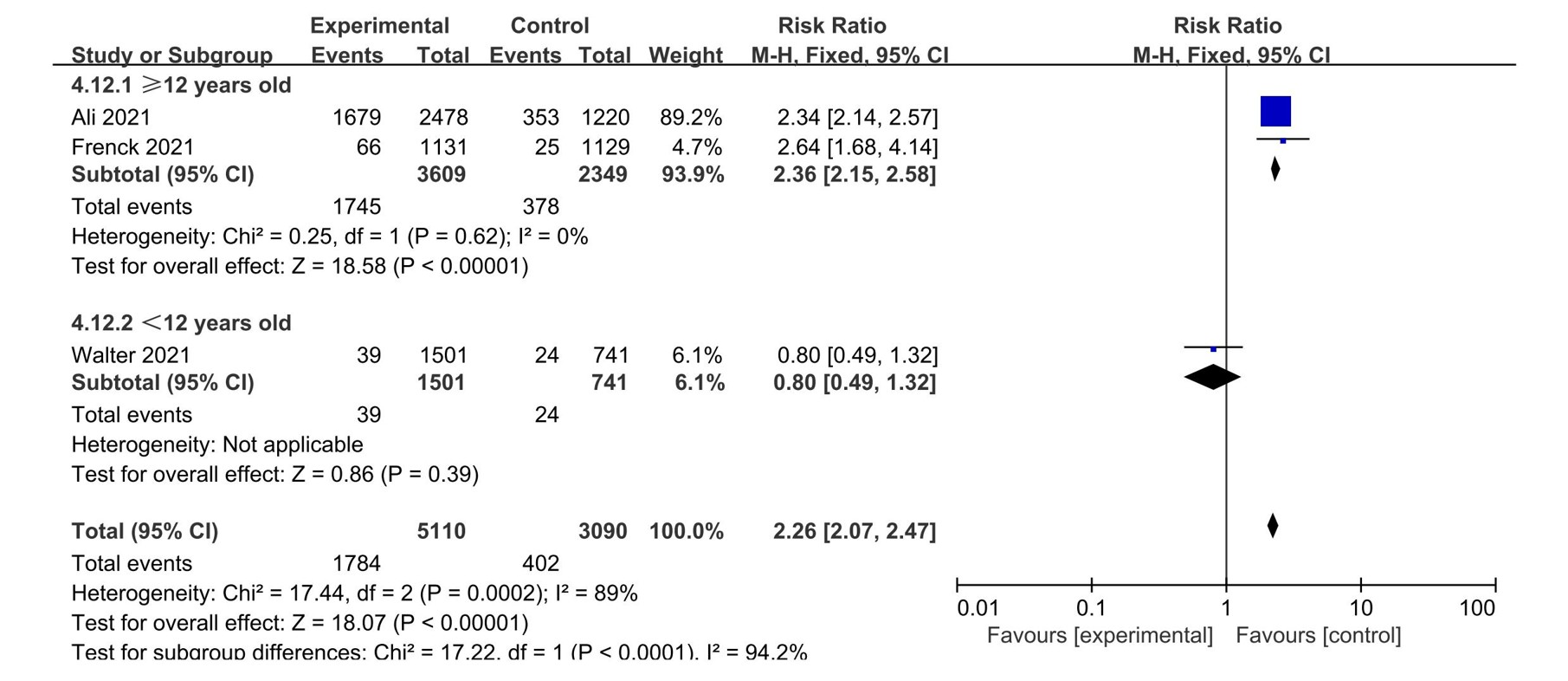

Supplement: Supplementary file 1 [file Data_Sheet_1.ZIP › Supplementary Material/Supplementary Figures (JPG)/Supplementary Figure 8. Adverse reactions in mRNA vaccine group of different ages versus control group/(L) Fatigue after the second vaccination.jpg]

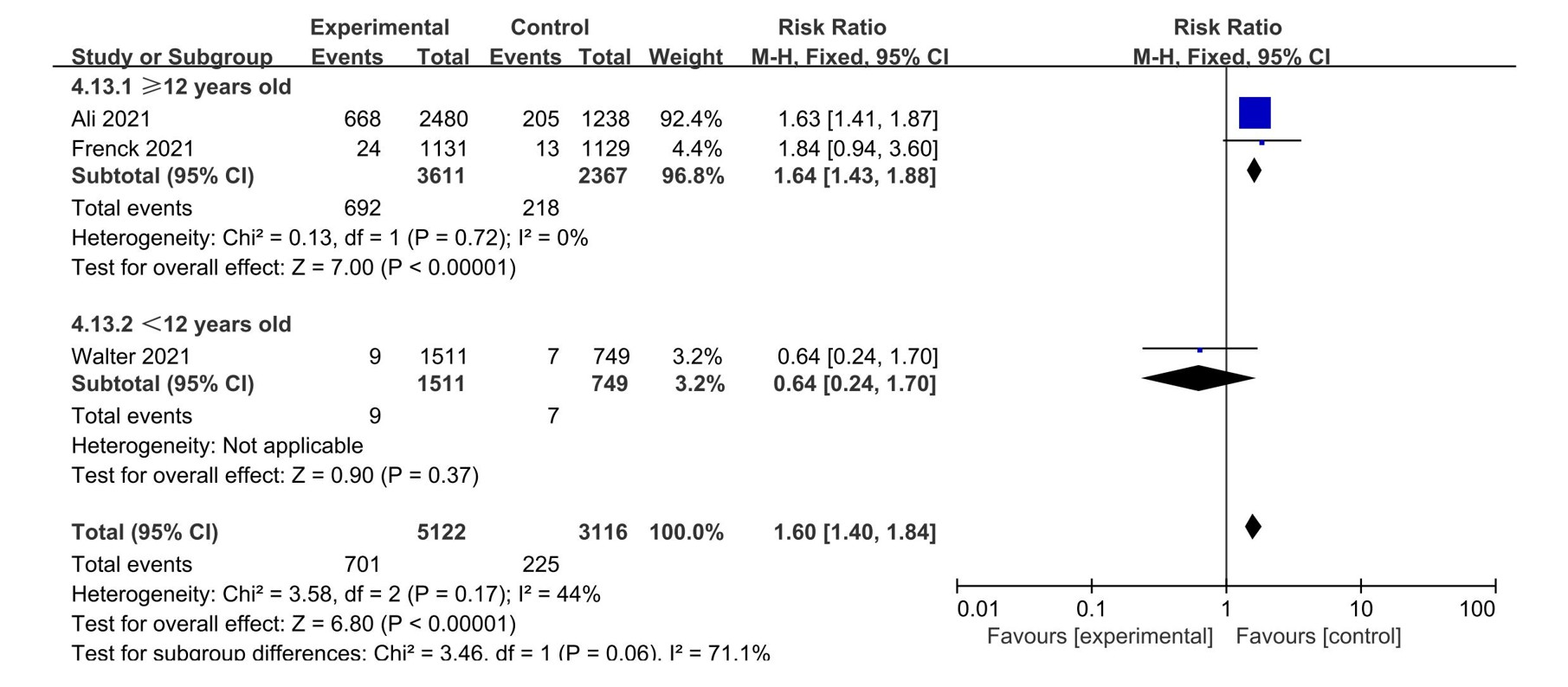

Supplement: Supplementary file 1 [file Data_Sheet_1.ZIP › Supplementary Material/Supplementary Figures (JPG)/Supplementary Figure 8. Adverse reactions in mRNA vaccine group of different ages versus control group/(M) Myalgia after the first vaccination.jpg]

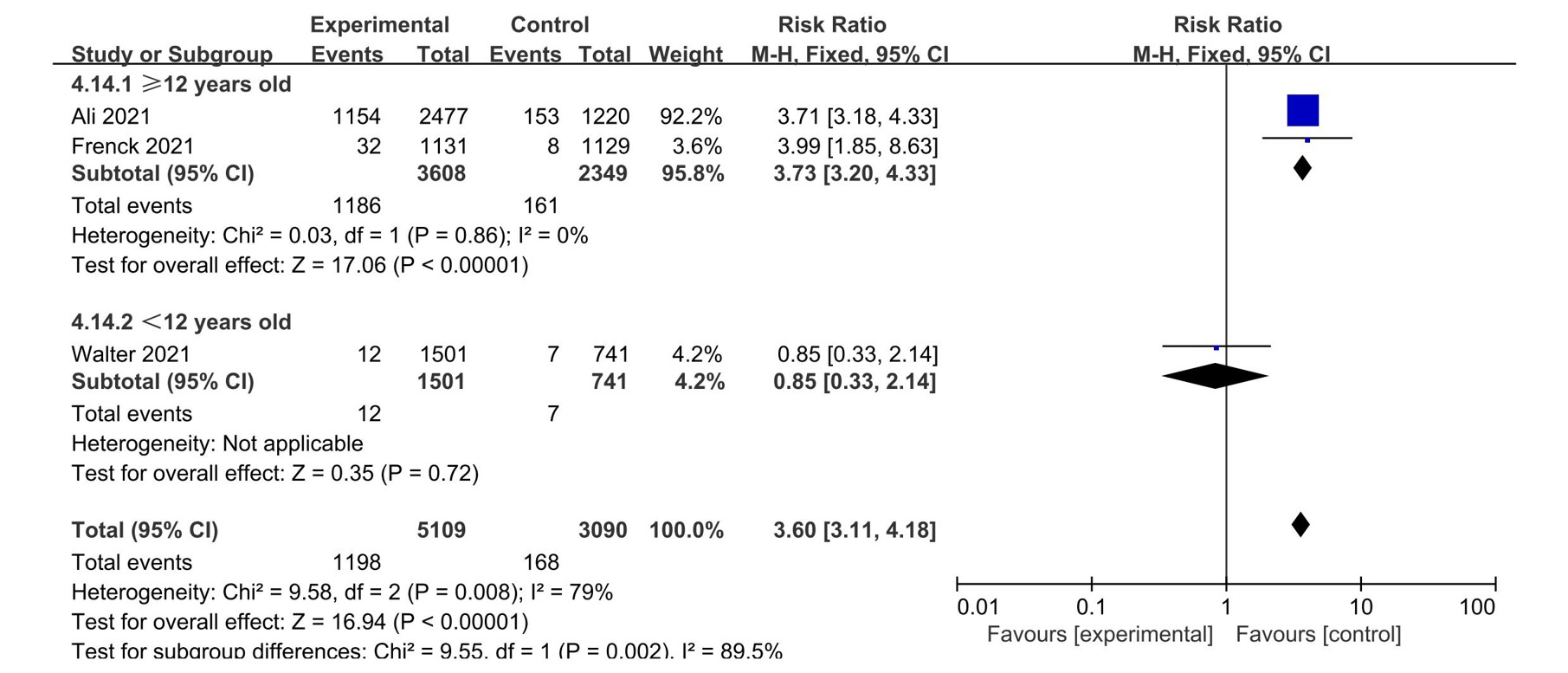

Supplement: Supplementary file 1 [file Data_Sheet_1.ZIP › Supplementary Material/Supplementary Figures (JPG)/Supplementary Figure 8. Adverse reactions in mRNA vaccine group of different ages versus control group/(N) Myalgia after the second vaccination.jpg]

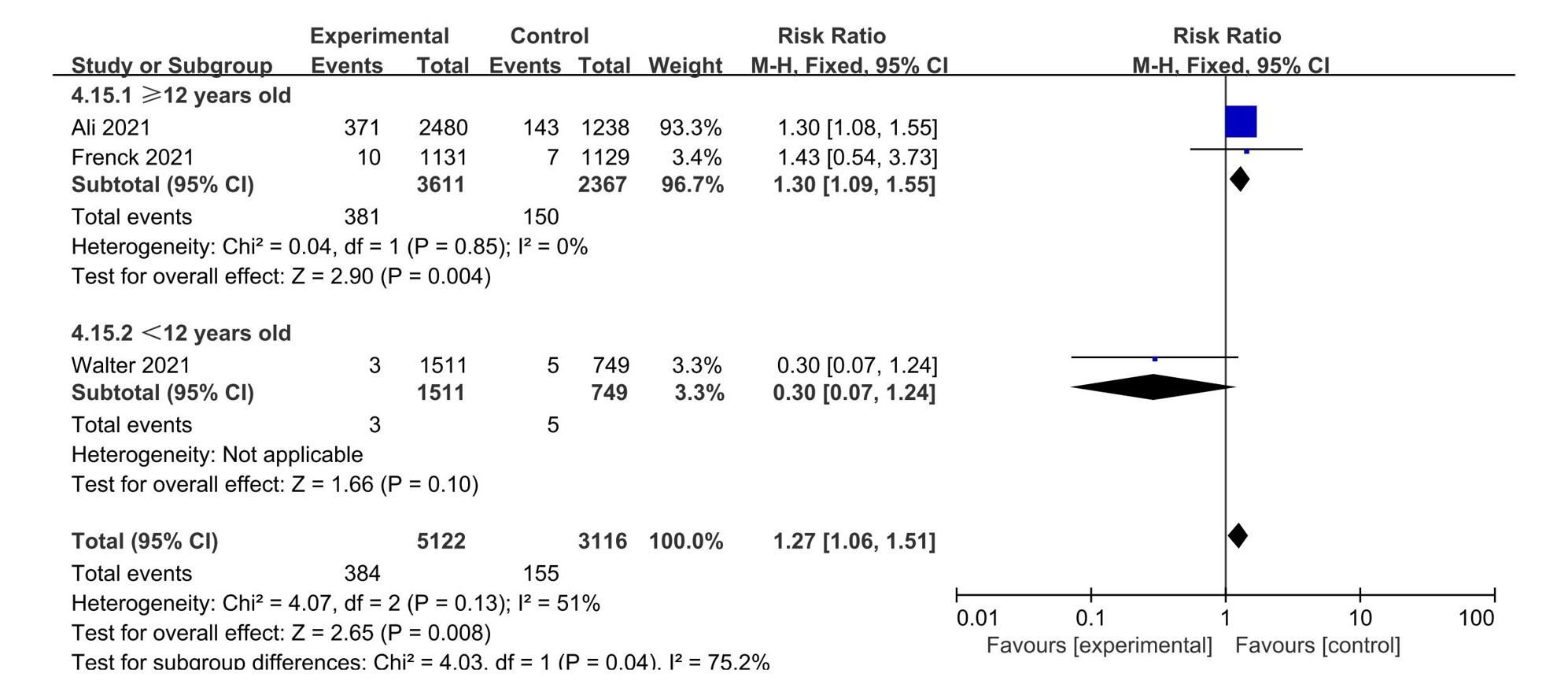

Supplement: Supplementary file 1 [file Data_Sheet_1.ZIP › Supplementary Material/Supplementary Figures (JPG)/Supplementary Figure 8. Adverse reactions in mRNA vaccine group of different ages versus control group/(O) Arthralgia after the first vaccination.jpg]

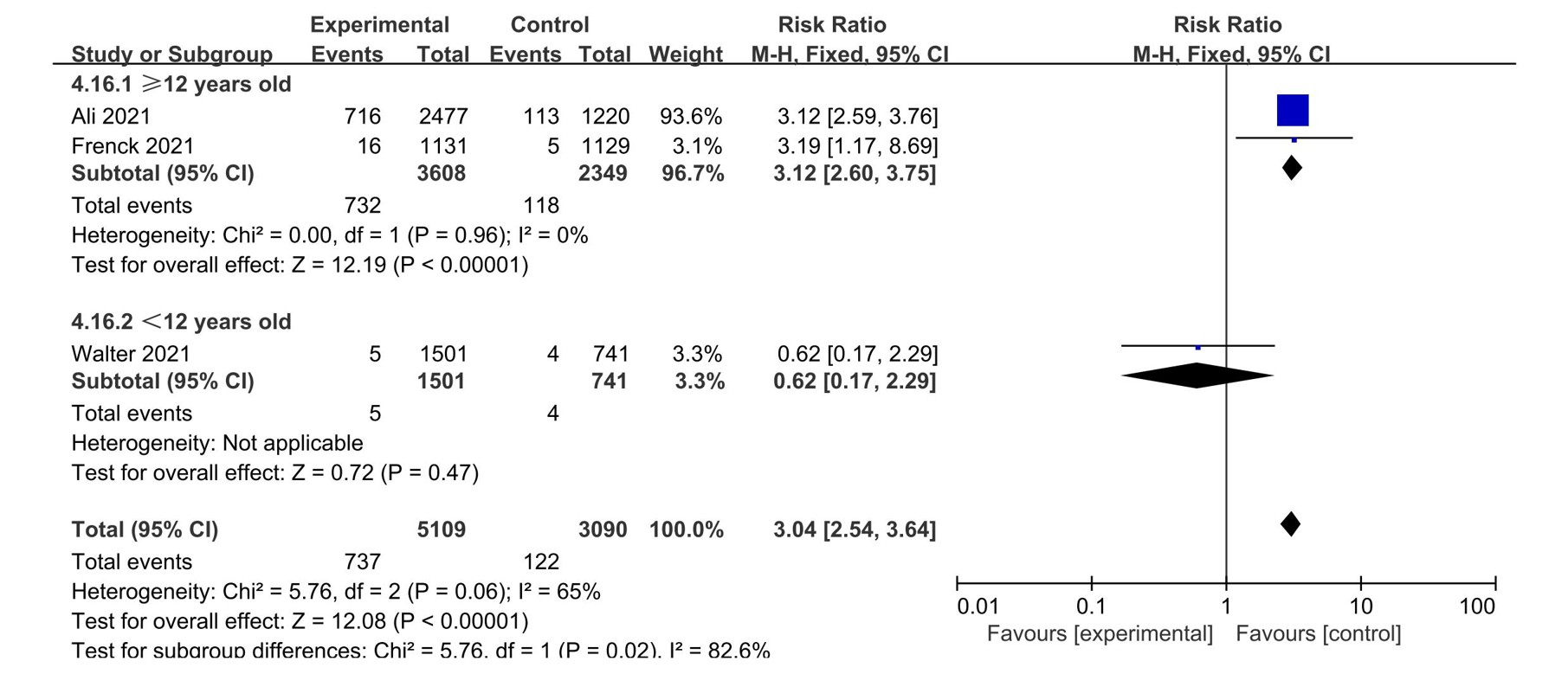

Supplement: Supplementary file 1 [file Data_Sheet_1.ZIP › Supplementary Material/Supplementary Figures (JPG)/Supplementary Figure 8. Adverse reactions in mRNA vaccine group of different ages versus control group/(P) Arthralgia after the second vaccination.jpg]

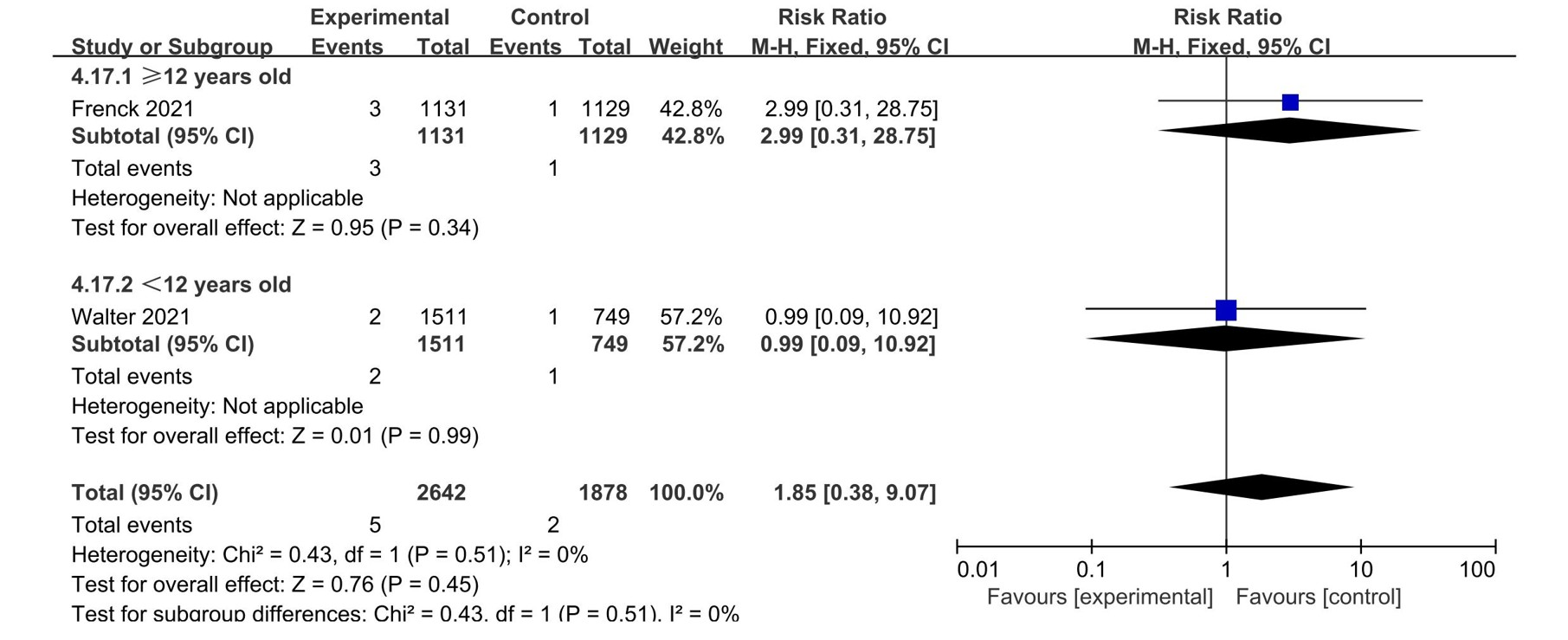

Supplement: Supplementary file 1 [file Data_Sheet_1.ZIP › Supplementary Material/Supplementary Figures (JPG)/Supplementary Figure 8. Adverse reactions in mRNA vaccine group of different ages versus control group/(Q) Vomiting after the first vaccination.jpg]

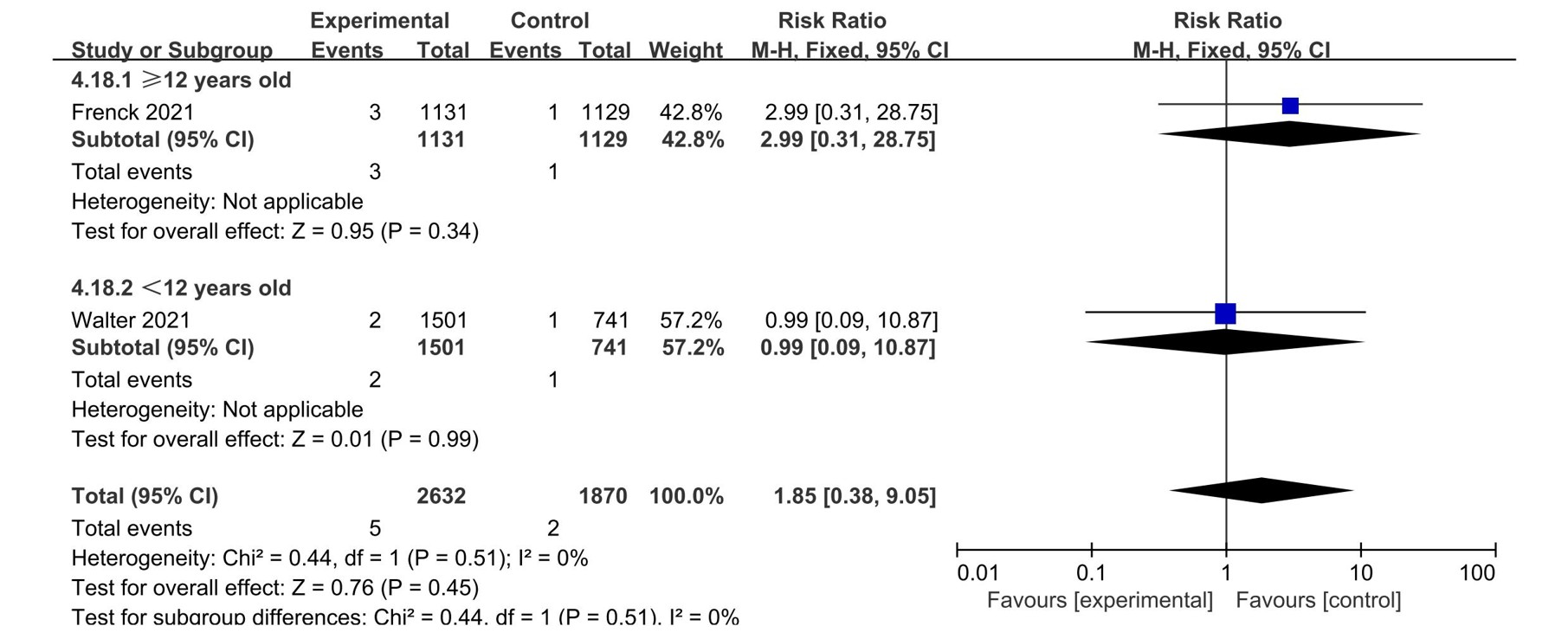

Supplement: Supplementary file 1 [file Data_Sheet_1.ZIP › Supplementary Material/Supplementary Figures (JPG)/Supplementary Figure 8. Adverse reactions in mRNA vaccine group of different ages versus control group/(R) Vomiting after the second vaccination.jpg]

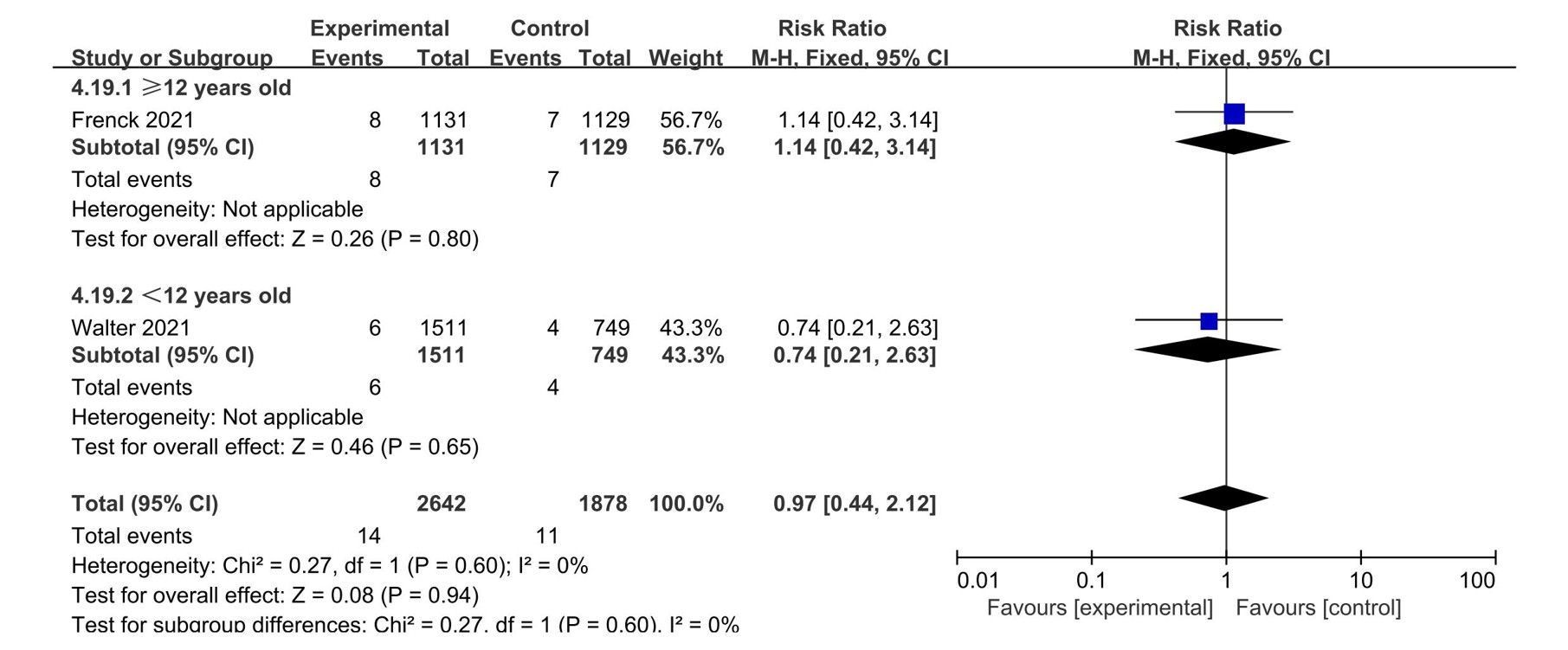

Supplement: Supplementary file 1 [file Data_Sheet_1.ZIP › Supplementary Material/Supplementary Figures (JPG)/Supplementary Figure 8. Adverse reactions in mRNA vaccine group of different ages versus control group/(S) Diarrhea after the first vaccination.jpg]

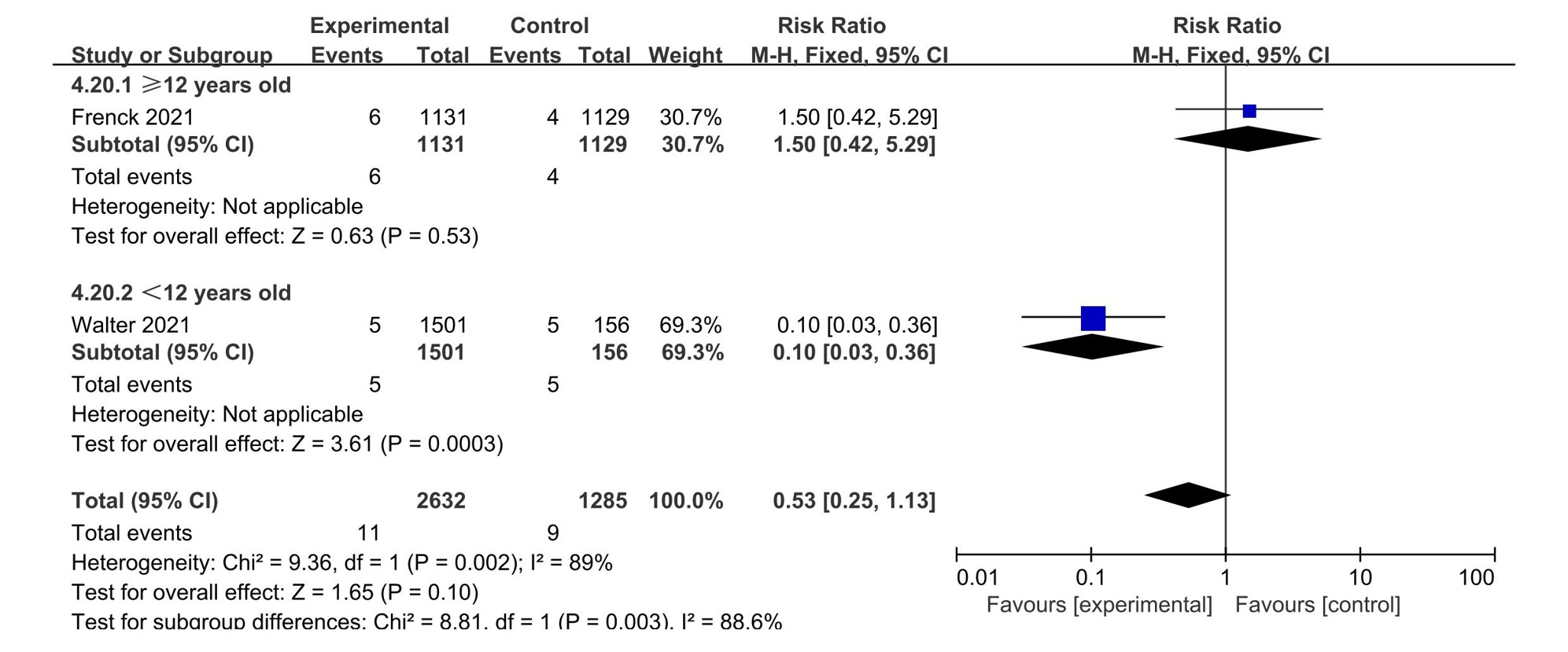

Supplement: Supplementary file 1 [file Data_Sheet_1.ZIP › Supplementary Material/Supplementary Figures (JPG)/Supplementary Figure 8. Adverse reactions in mRNA vaccine group of different ages versus control group/(T) Diarrhea after the second vaccination.jpg]

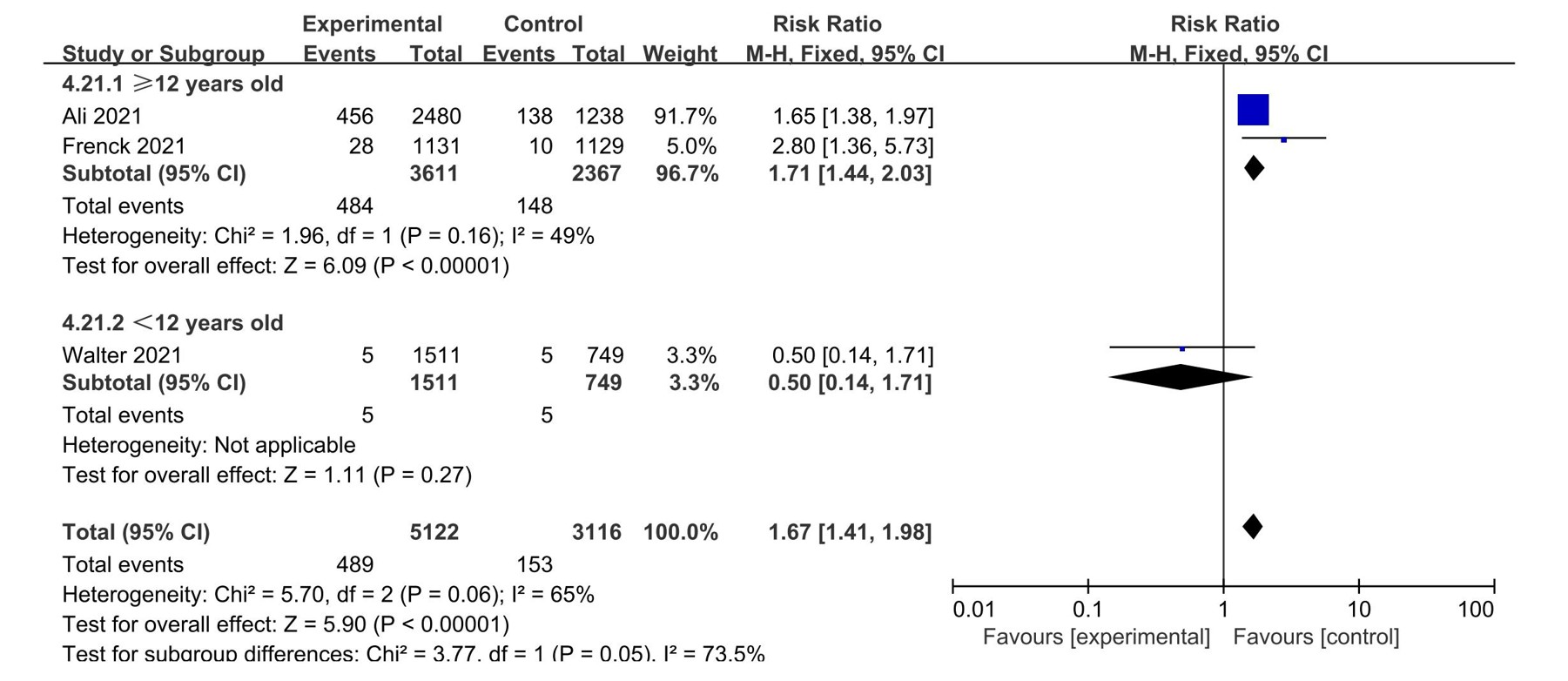

Supplement: Supplementary file 1 [file Data_Sheet_1.ZIP › Supplementary Material/Supplementary Figures (JPG)/Supplementary Figure 8. Adverse reactions in mRNA vaccine group of different ages versus control group/(U) Chills after the first vaccination.jpg]

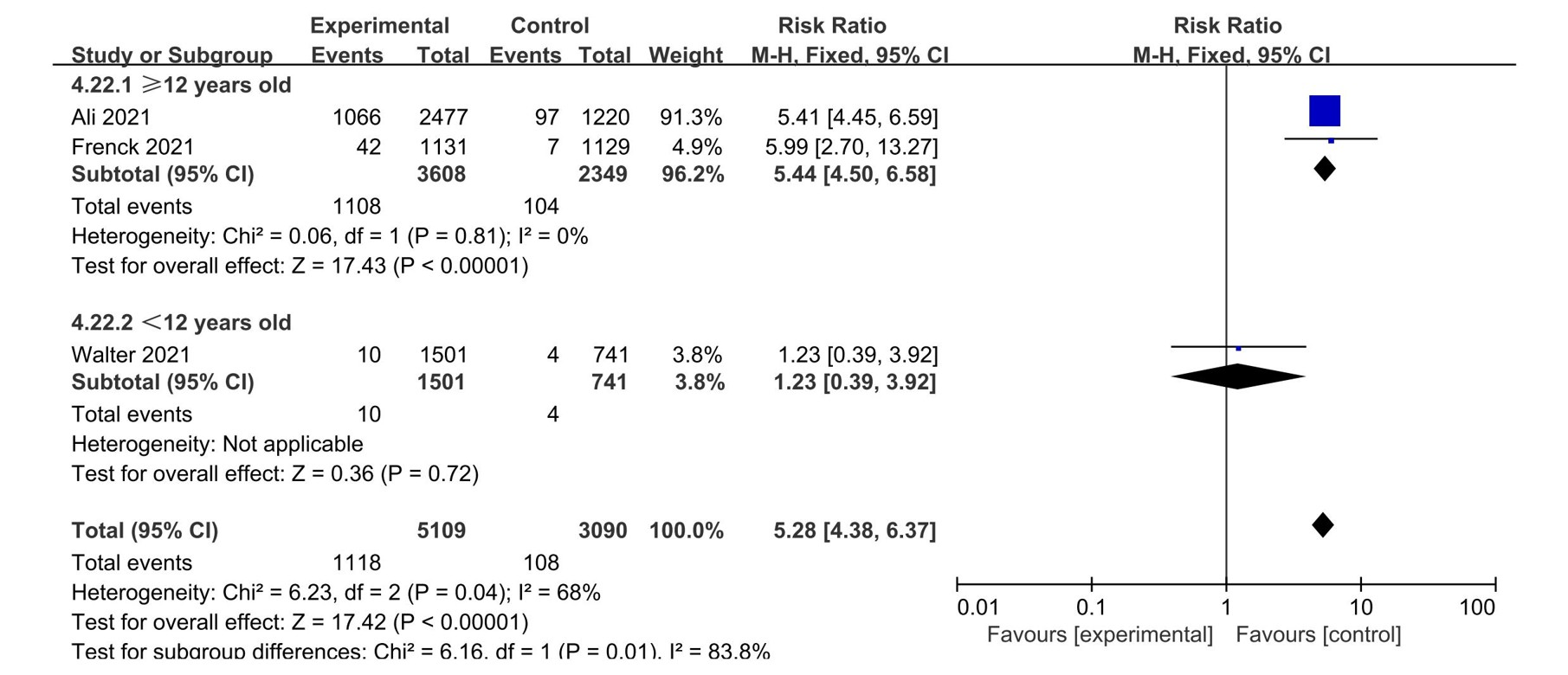

Supplement: Supplementary file 1 [file Data_Sheet_1.ZIP › Supplementary Material/Supplementary Figures (JPG)/Supplementary Figure 8. Adverse reactions in mRNA vaccine group of different ages versus control group/(V) Chills after the second vaccination.jpg]

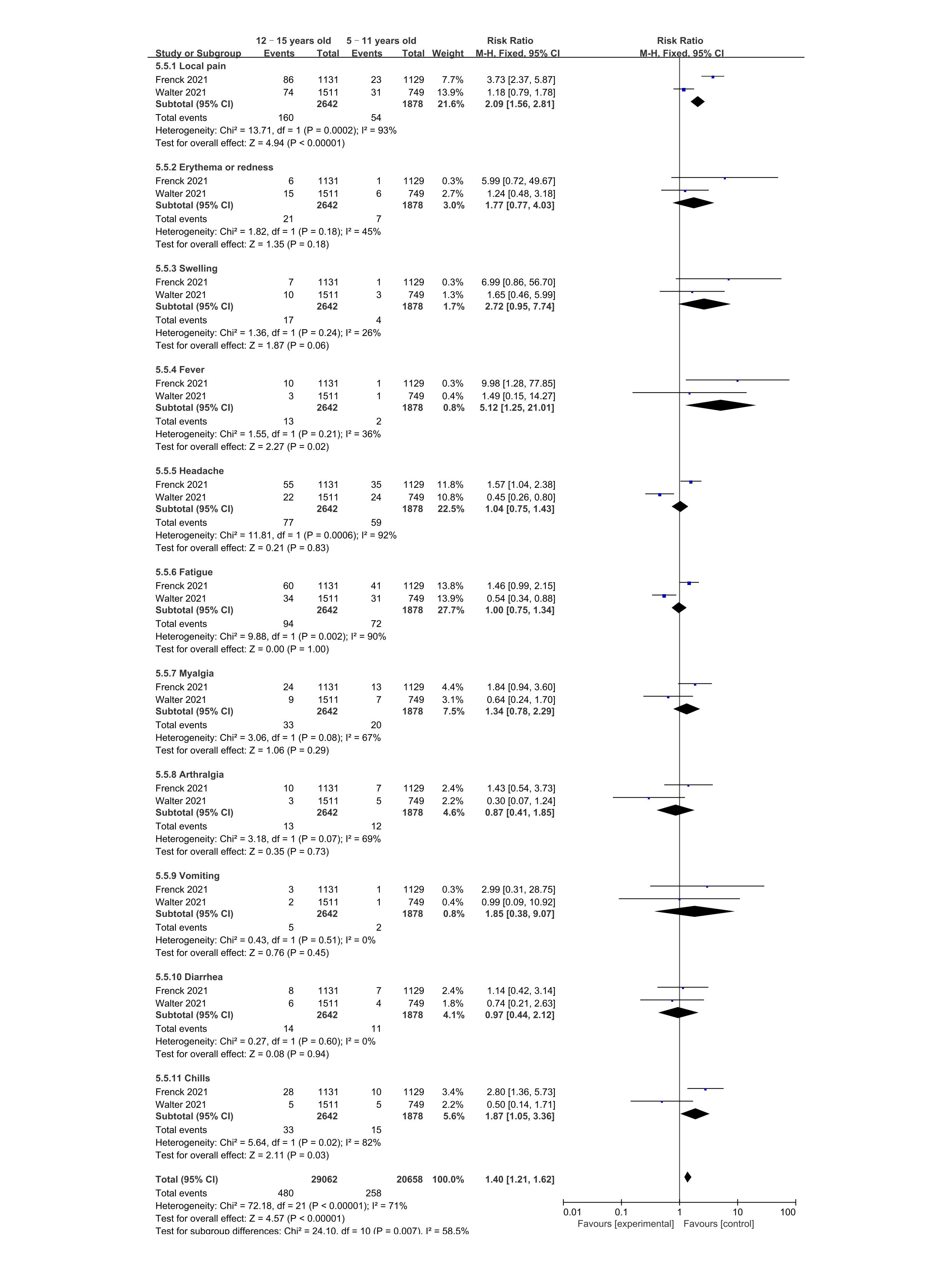

Supplement: Supplementary file 1 [file Data_Sheet_1.ZIP › Supplementary Material/Supplementary Figures (JPG)/Supplementary Figure 9. Specific adverse reactions in mRNA vaccine recipients aged í▌12 years versus 12 years/(A) After dose 1.jpg]

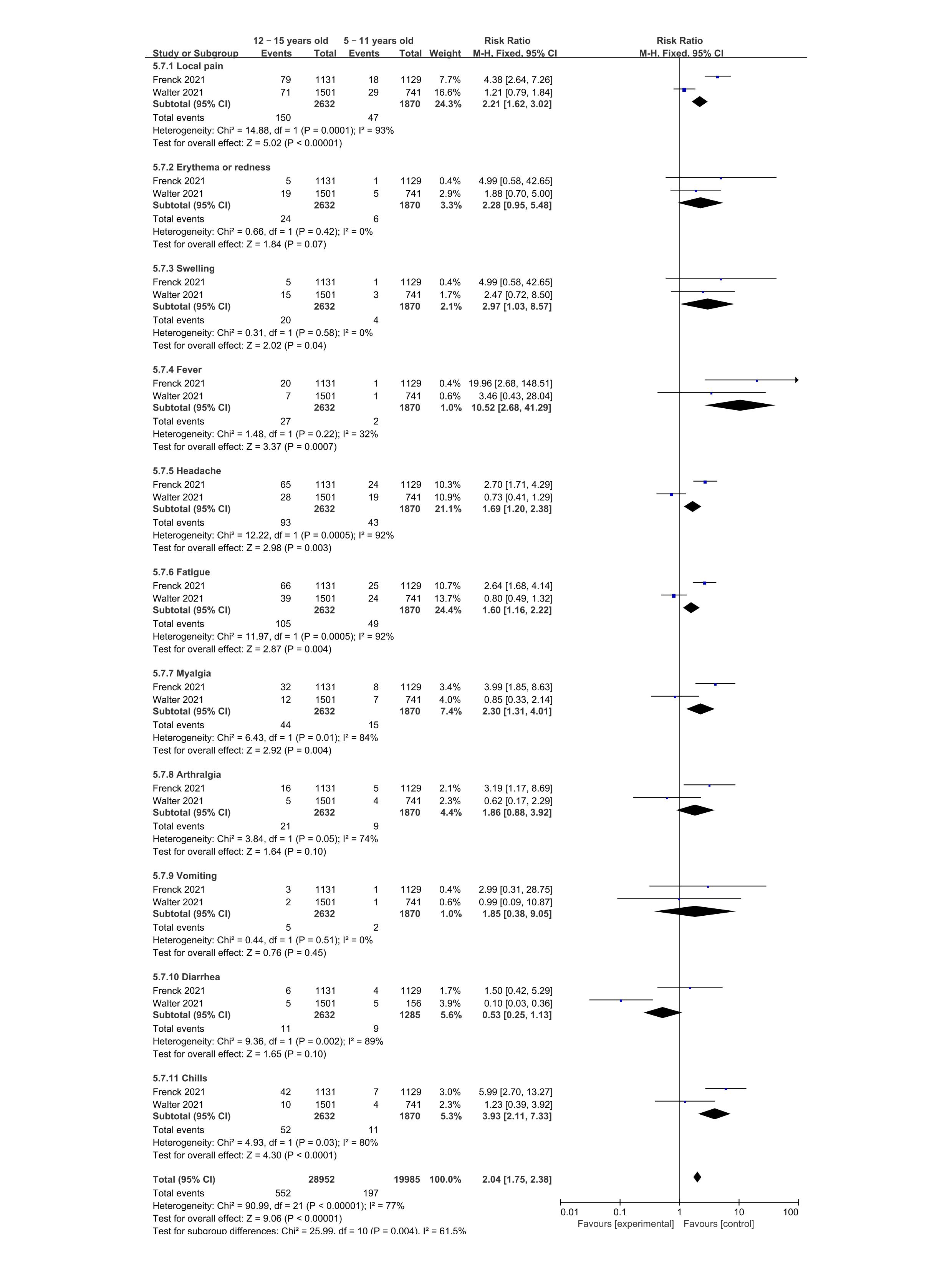

Supplement: Supplementary file 1 [file Data_Sheet_1.ZIP › Supplementary Material/Supplementary Figures (JPG)/Supplementary Figure 9. Specific adverse reactions in mRNA vaccine recipients aged í▌12 years versus 12 years/(B) After dose 2.jpg]
